# Pharmacological Interventions for Negative Symptoms in Schizophrenia: A Systematic Review of Randomised Control Trials

**DOI:** 10.3390/biomedicines13030540

**Published:** 2025-02-21

**Authors:** Lorenzo Moccia, Francesca Bardi, Maria Benedetta Anesini, Sara Barbonetti, Georgios D. Kotzalidis, Sara Rossi, Romina Caso, Flavia Grisoni, Giuseppe Mandracchia, Stella Margoni, Tommaso Callovini, Delfina Janiri, Marianna Mazza, Alessio Simonetti, Silvia Montanari, Gianna Autullo, Giovanni Camardese, Maria Pepe, Marco Di Nicola, Vassilij Di Giorgio, Fabio Conti, Gabriele Sani, on behalf of the Gemelli RePsy Study Group

**Affiliations:** 1 Department of Psychiatry, Fondazione Policlinico Universitario Agostino Gemelli IRCCS, Largo Agostino Gemelli 8, 00168 Rome, Italy; francesca.bardi97@gmail.com (F.B.); mbenedetta@hotmail.it (M.B.A.); sara.barbonetti@gmail.com (S.B.); sara.rossi1349@gmail.com (S.R.); casoromina@gmail.com (R.C.); dr.flaviagrisoni@gmail.com (F.G.); peppemandracchia@gmail.com (G.M.); stella.margoni98@gmail.com (S.M.); t.callovini@gmail.com (T.C.); delfina.janiri@unicatt.it (D.J.); marianna.mazza@policlinicogemelli.it (M.M.); alessio.simonetti@policlinicogemelli.it (A.S.); silvia.montanari.rm@gmail.com (S.M.); gianna.autullo@gmail.com (G.A.); mariapepe.992@gmail.com (M.P.); marco.dinicola@policlinicogemelli.it (M.D.N.); gabriele.sani@unicatt.it (G.S.); 2 Department of Neuroscience, Section of Psychiatry, Università Cattolica del Sacro Cuore, Largo Francesco Vito 1, 00168 Rome, Italy; 3 Menninger Department of Psychiatry and Behavioral Sciences, Baylor College of Medicine, Houston, TX 77030, USA; 4 Department of Life Science, Health, and Health Professions, Link Campus University, Via del Casale di S. Pio V, 44, 00165 Rome, Italy; 5 Istituto di Neuroscienze, Neomesia Kos Group, Via Nomentana 1362, 00137 Rome, Italy; vassilij.digiorgio@neomesia.com (V.D.G.); fabio.conti@neomesia.com (F.C.)

**Keywords:** schizophrenia, negative symptoms, pharmacological treatment, antipsychotics, antidepressants

## Abstract

**Background/Objectives:** While positive symptoms of schizophrenia are often satisfactorily controlled, negative symptoms are difficult to treat, persisting despite treatment. Different strategies have been devised to deal with this problem. We aimed to review drug treatment for negative symptoms of schizophrenia in controlled trials of marketed drugs. **Methods:** We searched the PubMed database and the resulting records’ reference lists to identify eligible trials using schizophrenia[ti] AND “negative symptom*”[ti] as a search strategy. We determined eligibility through Delphi rounds among all authors. **Results:** On 11 February 2025, we identified 1485 records on PubMed and 3 more from reference lists. Eligible were 95 records. Most studies were double-blind, randomized controlled trials, carried-out in add-on in patients stabilized with antipsychotics. Other antipsychotics were the most frequent comparators, followed by antidepressants, and recently, antioxidants are gaining importance in trials. Many trials, especially those conducted in the Western world, found no significant effects compared to placebo, while most Iranian studies were positive, although not with a strong effect size. **Conclusions:** Current research has contributed little to progress in the treatment of the negative symptoms of schizophrenia. The reason might reside in the absence of knowledge of the mechanisms whereby these symptoms are generated, which prevents us from designing possibly effective treatment strategies, and/or to the chronicity of negative symptoms, as they are the first to be established even when they do not become fully apparent.

## 1. Introduction

Schizophrenia (SCZ) was so termed by Eugen Bleuler in 1907, who introduced into the literature in 1908 [1] and further elaborated upon it in his seminal book of 1911 [2]. He considered it the heir of Emil Kraepelin’s dementia praecox [3], in turn remolded from Bénédict-Auguste Morel’s 1860 *dégénérescence* [4], in which was already embedded a strong negativity. Bleuler considered blunted affect, autism, loosening of associations of ideas, and ambivalence as fundamental (*Grundsymptomen*), while he saw catatonic behavior, delusions, and hallucinations as accessory (*akzessorischen Symptome*) [2]; this classification resembles what was later to become the positive vs. negative distinction. Gerd Huber [5] moved his formulation of the *Defektsyndrome* in the same direction, which has been translated as deficit syndrome in the English literature. Well after the introduction of neuroleptics in the treatment of SCZ (1952) [6] and the realization that their effects were carried on through inhibition of dopamine receptors (1966) [7], two British authors, Angus V.P. Mackay [8] and Timothy J. Crow [9], converged in suggesting that in SCZ, two different syndromes exist, one positive and one negative, with the former being characterized by response to dopamine receptor inhibitors the former and the latter being characterized by relative nonresponse, which also had a poorer prognosis. Separate assessment tools for positive and negative symptoms (or syndromes) were developed by Nancy Andreasen in the early and mid-1980’s [10,11] and for the combined assessment of both positive and negative syndromes of SCZ by Stanley Kay and his collaborators in 1987 [12]. Data of these scales were subjected to factor analyses that yielded more SCZ dimensions or clusters, like disorganization, reality distortion, excitement, and anxiety/depression, that were distinct from the positive and negative dimensions [13].

SCZ is a highly disabling psychiatric disorder characterized by heterogeneous clusters of symptoms. Among these, negative symptoms contribute significantly to long-term morbidity and poor functional outcomes in patients with SCZ. Negative symptoms usually involve a decrease in or lack of normal behaviors associated with motivation and interest, such as avolition, anhedonia, and asociality or with expression, such as blunted affect and alogia. Since anergia, anhedonia, avolition, and asociality are also core features of depression, the existence of depression in schizophrenia may be confused with negative symptoms [14]. However, depression in schizophrenia, despite its “negativity”, is a different type of symptom that also differs from that of depressive disorders; for this reason, the common depression assessment measures are inadequate for evaluating it; hence, depression in schizophrenia needs the employment of specifically designed scales, e.g., the Calgary Depression Rating Scale for Schizophrenia [15]. The negative symptoms of SCZ can be classified as primary, intrinsic to the disorder’s underlying pathophysiology, or secondary, arising from psychiatric or medical comorbidities, treatment side effects, or environmental factors [16,17]. Despite the prominence put on positive SCZ symptoms, like delusions and hallucinations, it is felt that the negative symptoms are more part of the pathophysiological core of the disorder since they are those that precede the full-blown outbreak of SCZ [16,18]. In spite of their unpleasantness, negative symptoms are unlikely to be recognized by the patient or his/her family members as pathological; thus, the people involved do not seek medical help, which in turn accounts for the usual long duration of untreated illness and duration of untreated psychosis [19,20,21]; these are later related to greater treatment resistance, even at the first episode of psychosis [22,23].

Despite their diversity and the challenge posed by diagnostic difficulties in differentiating them, their careful assessment, timely identification, and appropriate management are essential given the above considerations. Over half of patients with chronic SCZ exhibit at least one negative symptom, and the prevalence of persistent negative symptoms after the first psychotic episode ranges from 11% to 37% [24]. While current antipsychotic medications are generally effective in ameliorating the positive symptoms of SCZ, there are still limited options for treating negative symptoms [16]. Although in the past, some hope has been engendered by positive reports or perspectives for the use of atypical antipsychotics or dopamine agonists [25,26], current clinical practice is still unsatisfactory. In addition, negative symptoms do not undergo spontaneous improvement throughout the course of SCZ; while continuing stably with antipsychotic pharmacotherapy, they may even worsen upon antipsychotic withdrawal [27], or they may worsen with antipsychotic coadministration along other, possibly innovative drugs to deal with negative symptoms. Moreover, these new drugs can possibly have side effects of their own that reduce their effectiveness. Even with substantial advancements in the epidemiology, etiology, biology, and psychopharmacology of SCZ, negative symptoms persist as a critical unmet medical need [16].

### Objectives

Our aim was to identify the best clinical practices in treating negative symptoms in patients with SCZ. To achieve this, we searched the PubMed database for clinical trials aiming at improving negative symptoms in such patient population.

## 2. Materials and Methods

On 11 February 2025, we searched the PubMed database using schizophrenia[ti] AND “negative symptom*”[ti] as a search strategy. We used this as strategy to focus on studies actually investigating the negative symptoms of SCZ. We did not use terms like “drug treatment” or the names of specific antipsychotics or other drugs commonly used in SCZ since we could miss other treatment strategies that do not appear in the title, and we used words found in the title so as to increase the search’s specificity. Eligible were studies conducted with drugs, monotherapy, or in add-on when the background antipsychotic treatment was stable since a reasonable time and when the patients’ clinical status was unchanged but not when two (or more) drugs were concomitantly initiated since we could not disentangle the effects on negative symptoms of each drug. Studies had to focus on negative symptoms in patients with schizophrenia, schizoaffective disorder, or other schizophrenia spectrum disorders, including people with clinical high risk (ultra-high risk), such as adolescents and young adults with attenuated psychotic symptoms, brief-limited intermittent psychotic symptoms, or genetic risk/deterioration. Excluded were studies not using drug treatment (No drug); guidelines, reviews, and meta-analyses (collectively termed Review); editorials; theoretical issues with no experimentation, letters to the editor with no data and expressing the authors’ personal opinions (collectively termed Opinion); studies conducted with no control or in an open-label fashion (Open); case reports or case series (labelled as Case); animal studies (Animal); those that dealt with the subject matter but had an unfocused design (labelled as Unfocused); studies involving drugs that were not introduced in the market in the country where the study was conducted (Non-marketed); studies using the same sample or overlapping samples (labelled as Overlapping; of such studies, the best-quality study was chosen to represent the group and possibly the one with the largest sample, when the more recent study reported on a greater sample); post hoc studies reporting on already published studies (labelled Post hoc, but the individual studies were sought and included if eligible); protocols without even preliminary data (Protocol); studies not using placebo or adequate comparators (No placebo); studies on patients with different diagnoses not providing differentiated data for patients with schizophrenia spectrum disorders or using different drugs without providing figures for each drug (collectively termed Lumping); post mortem studies (Post mortem), those with a design that is not adequate to provide useful data for our purposes (Inadequate); those that were included in the results of the search due to serendipity but are otherwise unrelated to our subject matter (Unrelated); and possible studies in vitro or conducted on cell systems (termed In vitro). We also did not include articles that were retracted or removed by their authors or publishers (Retracted), while duplicates were eliminated.

To decide eligibility, all authors participated in Delphi rounds and discussed on every article. There was a particular focus on possible sources of bias in each study during each encounter, e.g., selection bias, attrition bias (drop-out rates), sponsor bias, bias due to the use of inadequate statistical methods or assessment methods, and so on. We did not exclude studies using imperfect methods but tracked every possible defect in design or reporting and report it in our Table 1, last column, i.e., Conclusion(s)/Observations. Rounds were repeated until complete consensus was reached. Not more than two were required to reach consensus.

We used the *PRISMA* statement to prepare the flowchart of our inclusion process and the checklist [28]. The risk of bias was assessed through the RoB 2 tool [29,30]. We registered the review on the OSF Registry.

## 3. Results

**Figure 1:**
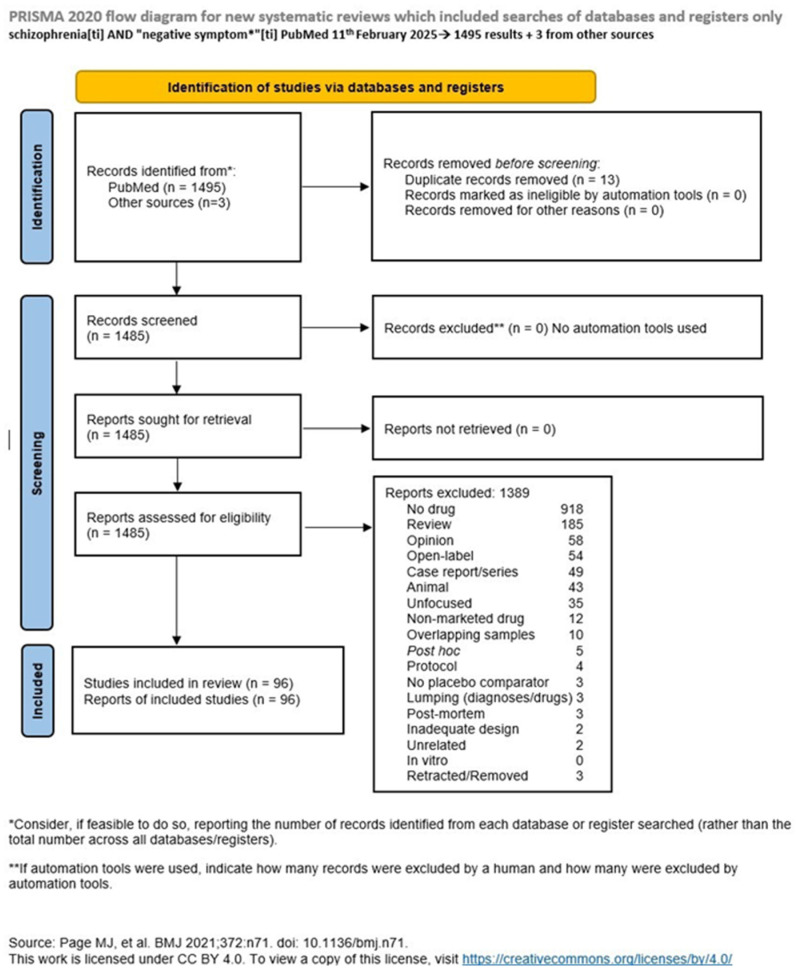
Flowchart of the systematic literature search according to PRISMA guidelines [28].

Our PubMed search, conducted on the above date and with the above strategy, identified 1495 articles, of which 94 were eligible. Another three were identified in the reference lists of the resulting articles, of which two were eligible, thus bringing the total output of eligible studies to 96. The selection process and its results, along with the reasons of exclusion for each article, are shown in the online Supplementary Materials. These results were taken into account in constructing the PRISMA flowchart, which also contains details on the reasons of exclusion, as shown in Figure 1. The first resulting article was published in June 1978, while the last went on the web on 5 February 2025. The eligible studies spanned from December 1985 to 20 January 2025, thus covering a more than 39-year range. Their summary is provided in Table 1 [31,32,33,34,35,36,37,38,39,40,41,42,43,44,45,46,47,48,49,50,51,52,53,54,55,56,57,58,59,60,61,62,63,64,65,66,67,68,69,70,71,72,73,74,75,76,77,78,79,80,81,82,83,84,85,86,87,88,89,90,91,92,93,94,95,96,97,98,99,100,101,102,103,104,105,106,107,108,109,110,111,112,113,114,115,116,117,118,119,120,121,122,123,124,125,126].

Regarding study locations, there were 25 in Iran (involving 39 sites), 19 studies in the U.S. (124 sites), 8 in China (9 sites), 7 in Israel (8 sites), 4 in the U.K. (31 sites), 5 in Italy (8 sites), 4 in Germany (4 sites), 3 in Spain (26 sites) and France (22 sites), 2 in India (2 sites), and 1 each in the Netherlands (6 sites), Australia (4 sites), Republic of Korea (1 site), South Africa (1 site), and Canada (1 site). Moreover, three were European studies conducted in various countries for a total of 181 sites, two involved European and African countries for a total of 64 sites, one was North American (U.S.–Canada; 40 sites) and another Pakistani–Brazilian (6 sites), while other three were international (one with 83 sites in two continents, one with 65 sites in five countries, and another in three continents involving 35 sites). One was U.S.–Israeli (5 sites) and another German–Austrian (30 sites). It should be stressed that while U.S.-only studies are scattered within a wider, 30-year time interval (May 1987–April 2017), in contrast, U.S.–other country collaborations started in 2003 and were still present in 2019. Iranian studies span across a 14-year interval; they started appearing in March 2008 but are increasing (the last appeared on 24 December 2024).

The eligible studies included a total of 10,287 participants, of whom 6197 were male and 2788 female; the sum of males and females is less than the total number of participants due to the fact that some studies did not specify the sex of participants. Some studies included male participants only, some because the hospitals where they recruited from were men only [94,116] and others for other reasons [32]. However, this did not account for the gender discrepancy in this review since the male-to-female prevalence ratio ranges from 1.4 [127] to 1.78 worldwide [128], whereas in our review it is 2.25, considerably higher than that found in the general population [129].

Of the included studies, seven were on minocycline (all in add-on), two were on intranasal oxytocin added to ongoing antipsychotics or in two psychotherapy sessions; another two involved a peptide analogue, DDAVP, in add-on; two were on verapamil, one in add-on and the other cross-over; overall, two studies had a crossover design, one with verapamil and the other with add-on glycine. Randomized controlled trials included 86 studies, of which one was open but randomized, and all other were non-randomized and double-blind. Almost all studied were double-blind (N = 93) save one. Of the eligible studies, 63 were placebo-controlled, and the other involved comparisons between drugs or different drug dosages. One study compared the SSRI antidepressant fluvoxamine with the tetracyclic maprotiline, three studies used the monoamine oxidase inhibitor selegiline/L-deprenyl in add-on, and four studies used the NaSSA (noradrenaline-serotonin specific antagonist) mirtazapine added on with various antipsychotics (one for each study). Haloperidol was used as an ongoing drug treatment to which mirtazapine or placebo was added and as a comparator in non-adjunctive studies in 12 comparisons: vs. risperidone and vs. clozapine three times each and once each vs. other antipsychotics like olanzapine, amisulpride, sertindole (four doses), and ziprasidone (two regimens) and vs. the calcium channel blocker verapamil. Olanzapine was used as ongoing treatment to which other drugs were added on in two studies, while it received comparisons with other antipsychotics in six studies (three vs. risperidone and one each vs. quetiapine, asenapine, and amisulpride or placebo) and in one study vs. the NaSSA antidepressant mirtazapine. Risperidone was used as a comparator in 10 studies: in the three aforementioned studies vs. risperidone and in three vs. haloperidol, plus two studies vs. cariprazine (two doses in one of them, plus vs. aripiprazole), one vs. quetiapine, and one vs. flupenthixol. Risperidone was the drug to which other study drugs were added in 26 studies, in which the study drugs were minocycline (four studies), citalopram vs. reboxetine, escitalopram, mirtazapine, duloxetine, selegiline, desmopressin, memantine, L-carnitine, *N*-acetylcysteine, citicoline, riluzole, cilostazol, simvastatin, sildenafil, granisetron, tropisetron, cerebrolysin, sarsasapogenin, pioglitazone, palmitoylethanolamide, pentoxifylline, sulforaphane (in this study, all ongoing antipsychotics were allowed, but most patients received risperidone [126]), and saffron. Other drugs used were modafinil and its R-isomer armodafinil (2 studies), the β-blocker propranolol, the antipsychotics thioridazine, pimozide and remoxipride, the antidepressants paroxetine and trazodone, the proneurosteroid pregnenolone (two studies), the selective estrogen receptor modulator raloxifene, the amino acid glycine (two studies), the GABA transaminase inhibitor D-cycloserine, the μ-opioid antagonist naltrexone, the anticonvulsant mood stabilizer carbamazepine, the acetylcholinesterase inhibitor galantamine, and the antioxidants nanocurcumin and black myrobalan.

Most studies used the PANSS [12] to rate schizophrenic psychopathology, especially the PANSS-Negative symptoms subscale (N = 88). The SANS [10] was used in 37 studies to rate negative symptomatology, while the positive symptoms-assessing SAPS [11] was used in 6 studies, always conjointly with the SANS, while only 2 used the semi-structured 16-item Negative Symptom Assessment scale (NSA-16) [130], and just 1 used the Self-Evaluation of Negative Symptoms (SNS) [131]. The Brief Psychiatric Rating Scale (BPRS) [132,133] was rated in 20 studies, while other scales included the Clinical Global Impressions (CGI) in 28 studies [134] as well as various scales to assess motor side effects. Depression was rated mainly through standard depression assessment scales like the Hamilton Depression Rating Scale (HAM-D) [135] (in 16 studies) and the Montgomery–Åsberg Depression Rating Scale (MADRS) [136] (in 3 studies), while the schizophrenia-specific scale that allows clinicians to distinguish between depression in schizophrenia and negative symptoms, the Calgary Depression Rating Scale (CDRS) [15], was used only in eight studies (involving the use of antidepressants in just two). Assessment timepoints were extremely heterogeneous.

**Table 1:** Summary of eligible studies in chronological order.

Study	Location	Population	Design	Results on Negative Symptoms	Conclusion(s)/Observations
Eccleston et al., 1985 [31]	Newcastle upon Tyne (Tyne and Wear) and Morpeth (Northumberland), England, U.K.	45 pts with chronic RDC SCZ; 11 ♀, 34 ♂. Divided in two groups: propranolol (N = 22, x^-^ age 44 ± 5.80 years; DoI x^-^ 19 ± 12.3 years) and thioridazine (N = 23, x^-^ age 46.3 ± 14.2 years, DoI x^-^ 19.8 ± 12.5 years	5-week RCT, DB study. On inclusion, pts had their AP medication discontinued. Pts were assessed on the BPRS and the NOSIE. The two groups received, respectively, 640 mg propranolol or 400 mg thioridazine in 4 doses/day.	BPRS total score: Propranolol group had a significantly higher ↓ from BL than thioridazine (*p* < 0.001) for the entire length of the study. Thioridazine group ↓ vs. BL at all time-points, of which only day 21 reached significance (*p* < 0.05). NOSIE total score: Propranolol significantly higher ↑ from BL than thioridazine (*p* < 0.001). Thioridazine was not significantly different from BL at any point.	The finding that propranolol resulted in significant BPRS and NOSIE improvement suggests that it has a role to play in the treatment of chronic SCZ. Propranolol had a significant influence on both positive and negative symptoms. In contrast, thioridazine did not show large improvements.
Price and Pascarzi, 1987 [32]	Youngstown, Ohio, U.S.	12 ♂ with SCZ (age 25 to 50)	DB, crossover study. Pts received Verapamil (80 mg × 4/day) and HAL (10–40 mg/day) × 30 days separated by 10-day plac wash-out. Each pt rated at days 0, 15, and 30 for each trial with the SANS.	Verapamil > effective than HAL on negative symptoms of SCZ (*p* < 0.01).	Calcium channel blockers may prove to be helpful in alleviating negative symptoms.
Uhr et al., 1988 [33]	La Jolla-San Diego, Stanford, Palo Alto; California, U.S.	12 ♂ with SCZ/SCAD (age 29–45) with negative symptoms	DB, PC. Verapamil (80 mg × 4/day = 320 mg/day) vs. plac × 2–6 wks (x^-^ = 4.75 wks). Negative symptoms measured weekly with the BPRS withdrawal-retardation subscale and a modified SANS subscale consisting of the five global items. Efficacy of verapamil tested with analysis of covariance controlling for BL negative symptoms.	Results from both the modified SANS score (F = 0.42; n.s.) and the BPRS withdrawal retardation subscale score (F = 0.07; n.s.) suggested that Verapamil and plac did not differ in their capacity to alleviate negative symptoms.	Verapamil did not affect negative symptoms.
van Kammen and Boronow, 1988 [34]	Bethesda, Maryland (Pittsburgh, Pennsylvania/Towson, Maryland) U.S.	17 ♂, 13 ♀ with SCZ (x^-^ age = 24 years; range = 18–35 years)	DB. Pts initially received DB plac capsules × 33 days. At 08:00 on the day of the infusion, after an overnight fast, each pt randomly received 20 mg DAmph in 0.9% saline iv or saline plac. A second infusion, using the alternate infusate, was performed identically 3–5 days later. In the second part, pts received DB pimozide (x^-^ dose 8 ± 1 mg/day) × 37 ± 2 days, adjusted for maximal clinical response. They then underwent a second DAmph infusion, this time omitting the paired plac infusion. Pts were rated on BPRS prior to each infusion and 30–45 min after	DAmph ↓ x^-^ scores on BPRS negative symptom items and depressed mood. DAmph led to improved scores, in ½–¾ of pts in emotional withdrawal (47%), motor retardation (53%), blunted affect (66%), and withdrawal/retardation cluster (77%). Pimozide pretreatment did not affect x^-^ improvement in negative symptoms following DAmph.	Negative symptoms of SCZ respond in part to iv DAmph.
Brambilla et al., 1989 [35]	Milan-Carate Brianza-Genoa, Italy	10 pts with chronic undifferentiated SCZ (6 ♂, 4 ♀), aged 28–63 (x^-^ age = 42.5 ± 10.6 years), DoI 6–31 years, illness onset 17–33 years	DB. Pts maintained on APs and first given a 20-day course of plac followed by 20 days DDAVP i.m., 4 µg. SANS, BPRS, NOSIE, and LN-RS administered to monitor negative symptomatology, behavior, and memory before study initiation, after plac, and after DDAVP administration	A significant ↓ of global scores was observed after DDAVP; the negative BPRS items showed significant post-DDAVP ↓; memory, examined by LN-RS, showed memory improvement in 5 pts and n.s. change in the group; NOSIE showed improvement of items related to social interests and manifest psychoses. Overall, 5 pts showed ↓ in negative symptoms, memory, and behavior.	DDAVP therapy induced some improvement of negative symptoms.
Silver and Nassar, 1992 [36]	Flugelman (Mazra) Psychiatric Hospital, Acco, Israel	25 inpts with chronic SCZ (♂ 20, ♀ 5; age = 40.80 ± 9.26) randomized to fluvoxamine (N = 13) or maprotiline (N = 12)	DB, PC, RCT of fluvoxamine (50–100 mg/day) vs. maprotiline (50–100 mg/day) for 6 weeks. Assessments with BPRS, SANS, SAPS, MADRS, and Neurological Rating Scale for EPS at BL and 2 wks × 6 wks	Fluvoxamine significantly improved negative symptoms compared to maprotiline (*p* < 0.05). No significant changes in positive symptoms or depressive symptoms.	Fluvoxamine is more effective than maprotiline in reducing negative symptoms in SCZ, likely due to its serotonergic action rather than its antidepressant effect.
Marder and Meibach, 1994 [37]	Multicenter study (20 sites in the U.S.)	388 inpts with DSM-III-R SCZ (♂ 340, ♀ 48; age = 37.4 ± 10.4) randomized to RISP (2, 6, 10, or 16 mg/day) or HAL 20 mg/day or plac	DB, PC, RCT of RISP (2, 6, 10, or 16 mg/day) vs. HAL 20 mg/day vs. plac for 8 weeks. Assessments with PANSS, CGI, and EPS at BL and wks 1, 2, 3, 4, 6, and 8	RISP (6 and 16 mg) significantly improved positive and negative symptoms compared to plac and was superior to HAL in improving negative symptoms. Higher doses of RISP and HAL had more EPS.	RISP (6 mg/day) is effective in treating both positive and negative symptoms of SCZ with fewer EPS compared to HAL. 6 mg/day is optimal for efficacy and safety.
Nachshoni et al., 1994 [38]	Shalvata Mental Health Center, Hod Hasharon, Israel	28 hospitalized pts with chronic, residual SCZ (♂ 14, ♀ 14; age = 46.6 ± 7.7 years; x^-^ DoI = 19.3 ± 6.5 years)	DB, PC, RCT of carbamazepine (600 mg/day) vs. plac for 7 weeks. Assessments with SANS, BPRS, HAM-D, and SAS at BL and wks 2, 4, and 7	No significant improvement in negative symptoms with carbamazepine. Minimal positive symptoms and EPS were observed.	Carbamazepine did not show a beneficial effect on negative symptoms in chronic SCZ. Different dosages and longer duration should be further tested.
Breier et al., 1994 [39]	Baltimore, MD, U.S.	39 outpts with chronic SCZ (♂ 28, ♀ 11; age = 34.5 ± 7.3) randomized to CLZ (N = 19) or HAL (N = 20)	DB, PC, RCT of CLZ (410.5 mg/day) vs. HAL (24.8 mg/day) for 10 weeks. Assessments with BPRS, SANS, and SAS for EPS at BL and wk 10	CLZ significantly improved BPRS positive symptoms and was superior to HAL for negative symptoms, particularly in nondeficit SCZ. CLZ led to minor worsening of EPS.	CLZ is superior to HAL for positive symptoms and has some effect on negative symptoms, mainly in nondeficit SCZ. Side effects were manageable.
Javitt et al., 1994 [40]	Bronx, NY, U.S.	14 ♂ pts with chronic SCZ (x^-^ age = 36.0 ± 9.7) randomized to glycine (N = 7, x^-^ age = 36.0 ± 9.7, x^-^ DoI = 15.5 ± 8.1) or plac (N = 7, x^-^ age = 38.1 ± 7.2, x^-^ DoI = 20.0 ± 6.6)	DB, PC, RCT of glycine 2–30 g/day vs. plac × 8 wks at Bronx Psychiatric Center. Assessment with PANSS, ESRS, and AIMS at BL and wks 2, 4, 6, and 8	Significant ↓ negative symptoms in glycine group compared to plac (*p* < 0.05). No significant differences in positive symptoms, ESRS, or AIMS scores.	Glycine may ↓ negative symptoms in TRS without exacerbating positive symptoms. Longer-term studies needed.
Decina et al., 1994 [41]	Guidonia, Lazio, Italy	47 pts with chronic, residual SCZ (♂ 23, ♀ 24; x^-^ age = 55.9 ± 6.3) randomized to trazodone (N = 26) or plac (N = 23)	DB, PC, RCT of trazodone up to 300 mg/day vs. plac × 6 wks at a tertiary psychiatric hospital. Assessment with BPRS, SANS at BL and days 21 and 42	Trazodone significantly but modestly ↓ withdrawal-retardation (*p* < 0.01) and affective flattening (*p* < 0.001) without affecting positive symptoms.	Trazodone mildly ↓ negative symptoms without exacerbating positive symptoms. Benefits may outweigh risks.
Boyer et al., 1995 [42]	Multicenter study (20 centers in France)	104 inpts with DSM-III SCZ (♂ 65, ♀ 39; x^-^ age = 32.5 ± 8.0) randomized to amisulpride 100 mg/day (N = 34, x^-^ age = 32.5 ± 8.2, x^-^ DoI = 118 ± 89 months) or amisulpride 300 mg/day (N = 36, x^-^ age = 34.1 ± 8.3, x^-^ DoI = 145 ± 96 months) or plac (N = 34, age = 30.7 ± 7.6, DoI = 117 ± 91 months)	DB, PC, RCT of amisulpride 100 mg/day or 300 mg/day vs. plac × 6 wks. Assessments with SANS, SAPS, and EPS at BL and wks 2, 4, and 6	Both doses of amisulpride were significantly more effective than plac in reducing SANS total scores (*p* < 0.02). No significant changes in SAPS or EPS scores.	Low-dose amisulpride can improve negative symptoms in SCZ without worsening positive symptoms. Supports dopaminergic hypofunction hypothesis.
Marchesi et al., 1995 [43]	Ancona, Marche, Italy	18 pts with chronic SCZ (13 ♂, 5 ♀, 9 inpts, 9 outpts; x^-^ age 35.0 years [range 24–56 years], x^-^ DoI 11.5 years [range 3–36] assigned to Naltrexone (N = 9, 7 ♂, 2 ♀, 4 inpts, 5 outpts; x^-^ age 35.1 years [range 27–56 years], x^-^ DoI 11.5 years [range 5–36]) or plac (N = 9, 6 ♂, 3 ♀, 5 inpts, 4 outpts; x^-^ age 35.0 years [range 24–50 years], x^-^ DoI 11.5 years [range 3–23])	DB, PC study of oral naltrexone (50 mg bid) or plac × 14 days added on stable routine AP and anxiolytic therapy; pt symptoms assessed with SAPS, SANS, BPRS, and CGI at BL and wks 1 and 2	Pts on naltrexone with or without negative symptoms and plac with or without negative symptoms ↓ BPRS-total score, while only pts on naltrexone ↓ their scores on the withdrawal-retardation and hostile suspiciousness domains from BL to wk 2. No differences on CGI scores between the two groups or within groups.	Naltrexone might help in treating negative symptoms. However, the paper is very badly written, and its statistics raise many doubts (usually significances were marginal in the face of small sample sizes). Limitations not reported.
Loo et al., 1997 [44]	Paris, France	N = 141 pts with SCZ (55% with residual type, 82% with chronic SCZ, x^-^ age 34 ± 10 years; 100 ♂, 41 ♀; x^-^ DoI 10.2 ± 8.6 years; previous treatments: neuroleptics [41%], antiparkinsonians [11%], ADs [13%], anxiolytics [15%], hypnotics [18%]) randomized to amisulpride (N = 69, x^-^ age 32 ± 10 years; 46 ♂, 23 ♀; x^-^ DoI 9.7 ± 8.4 years) or plac (N = 72, x^-^ age 36 ± 10 years; 54 ♂, 18 ♀; x^-^ DoI 10.7 ± 8.8 years)	DB, PC, RCT, multicenter study of 100 mg/day amisulpride vs. plac × 6 months at Sainte-Anne–Hôpital Cochin and other not declared sites. SANS, SAPS, CGI, GAF, WSEEPS, BARS, and AIMS at BL and endoint	Responders (≥50% ↓ from BL of SANS-total score) were significantly more in the amisulpride (42%) than in the plac group (15.5%) *p* < 0.001; amisulpride ↓SANS and CGI scores and ↑ GAF but not SAPS scores. Similar side effects between groups and no differences in akathisia or EPS.	Amisulpride is effective in the medium-term treatment of SCZ pts with predominantly negative symptoms. The study demonstrated what the sponsors wished. Two authors with sponsor’s affiliation.
Awad et al., 1997 [45]	Toronto, Ontario, Canada	205 pts with chronic SCZ (152 ♂, 53 ♀) randomized to remoxipride (N = 97, x^-^ age 37.7 years; 81% (N = 79) ♂, 19% (N = 18) ♀; x^-^ DoI 13.9 years) and HAL (N = 108, x^-^ age 37.3 years; 68% (N = 73) ♂, 32% (N = 35) ♀; x^-^ DoI 13.6 years)	Multicenter (sites not declared), RCT, DB, parallel study in Toronto, (remoxipride vs. HAL × 28 wks). Flexible dosing, remoxipride x^-^ 334.1 mg (150–600 mg) and HAL 10.44 mg (5–20 mg) during the last wk. Outcomes: PANSS-N and QoL scores	Comparable improvement in negative symptoms from BL for remoxipride and HAL (response as defined as a ≥20% ↓ from BL of PANSS-N scores, 49.4% with remoxipride and 47.6% with HAL). Similar improvements in QoL.	Use of low-dose FGA (HAL) yielded similar improvement in negative symptoms with that of an “atypical” AP (remoxipride, which is in fact a substituted benzoamide, a class swinging between FGAs and SGAs).
Speller et al., 1997 [46]	Plymouth, Devon, England and London, England, U.K.	N = 60 inpts with SCZ (x^-^ age 63 years [range 35–76]; 46 ♂, 14 ♀; x^-^ DoI 441 months, pre-trial AP dose: 200 mg/day CPZ equivalents, not receiving AP: 7, receiving pre-trial LAI: 19) randomized to amisulpride (N = 29, 20 ♂, x^-^ age 64 years [range 40–75], x^-^ DoI 452 months) or HAL (N = 31, x^-^ age 63 years [range 35–76]; 46 ♂, 14 ♀; x^-^ DoI 441 months)	DB, double-dummy two-site RCT of amisulpride vs. HAL × 1 year, 22 (76%) pts started on amisulpride had achieved or maintained a low-dose level of 100–100 mg/day, and 18 (58%) of those started on HAL were similarly receiving the estimated equivalent low dose of 3–4.5 mg/day. In the amisulpride group, 11 (38%) pts had at least one dose reduction, while the respective figure for the HAL pts was 6 (19%). However, as a result of dosage increases to tackle psychotic exacerbations, 5 amisulpride pts (18%) and 7 HAL pts (27%) were receiving higher doses of medication after one year than at BL	The low-dose regimens with amisulpride and HAL failed to produce significant ↓ in x^-^ scores in negative symptoms. However, comparing the two treatments, there was a trend for greater improvement in SANS affective flattening and avolition-apathy domains in the amisulpride group.	In chronically hospitalized inpts with SCZ characterized by persistent negative symptoms, amisulpride was a well-tolerated maintenance antipsychotic medication. The drug had only a limited effect in reducing negative symptoms, which were relatively stable, enduring phenomena in this sample, despite dose reduction.
Heresco-Levy et al., 1999 [47]	Jerusalem, Israel	22 TRS pts (12 ♂, 10 ♀; x^-^ age 38.8 ± 11.0 years), PANSS positive and negative symptom scores ≥70th percentile, based on normative data for inpts with chronic SCZ. To be eligible, pts had to have been treated with a stable, clinically determined, optimal oral dose of a conventional or atypical AP for ≥3 months	DB, PC, crossover RCT to 0.8 g/kg/day glycine add-on to fixed AP doses vs. plac × 6 wk → 2 wk adjuvant washout (wk 6–8)→crossed to opposite × 6 wk (wk 8–14). Assessment with PANSS, BPRS, SAS, and AIMS at BL (2 wk from start) to wk 14 bi-weekly	19 pts completed both glycine and plac trials. Glycine well tolerated and → ↑ glycine and serine serum levels. Glycine → significant 30 ± 16% ↓ in negative symptoms, as measured by the PANSS.	Findings support hypoglutamatergic hypotheses of SCZ.
Rosenheck et al., 1999 [48]	VA centers across U.S.	422 hospitalized DSM-III-R TRS pts with a history of ↑ hospitalization for SCZ during the previous year but <364 days randomized to CLZ vs. HAL in 15 VA centers; sex not specified	DB RCT of CLZ (100–900 mg/day, N = 205) or HAL (5–30 mg/day, N = 217) and treated × 12 months. HAL-treated pts also received benztropine mesylate (2–10 mg/day) for EPS; CLZ pts received a matching benztropine-plac. HAL pts participated in weekly blood counts as required for pts on CLZ. Symptoms assessed with PANSS at BL and 6 wk and 3, 6, 9, and 12 months after random assignment	Pts on CLZ ↑ improvement than those on HAL in negative symptoms at 3 months. CLZ had no independent effect on negative symptoms at any time after controlling for positive symptoms. No significant differences in response to CLZ between pts with high and low levels of negative symptoms at BL or between pts with and without deficit syndrome.	The greater effectiveness of CLZ as compared to conventional medications in TRS is not specific to either negative clinical symptoms or clinical subtypes defined by prominent negative symptoms or evidence of the deficit syndrome. Methods and Results reported in awkward and untidy manner.
Danion et al., 1999 [49]	Multicenter (29), multinational (France, Spain, Tunisia, and Italy)	242 inpts or outpts (154 ♂, 88 ♀; x^-^ age 34.7 ± 9.4) aged 18–60 years with DSM-III-R SCZ, residual type (295.6), of ≤20-year duration (mentally retarded pts excluded). Predominantly negative symptoms, with a total score of ≥60 on the SANS and ≤50 on the SAPS	After completion of a 4-week washout period, pts with SCZ and primary negative symptoms participated in a 12-week, multicenter, multinational DB, PC, RCT of plac (N = 83) vs. amisulpride 50 mg/day (N = 84) vs. amisulpride 100 mg/day (N = 75). They were evaluated with the SANS	Both amisulpride treatment groups showed significantly greater improvement in negative symptoms than the plac group. Positive symptom scores changed minimally during the study, so the improvement in negative symptoms was independent. The safety of amisulpride (either dose) was comparable to that of plac.	These findings confirm and extend those of earlier PC studies of low-dose amisulpride in the treatment of pts with predominantly negative symptoms of SCZ. Results of the study according to sponsor’s wishes.
Jungerman et al., 1999 [50]	Haifa, Israel	16 pts with DSM-IV SCZ (8 ♂, 8 ♀; x^-^ age 35.7 ± 6.3 years) without marked mood symptomatology assigned to selegiline vs. plac matched for demographic and clinical characteristics	DB, PC, RCT. Pts maintained on stable AP × ≥3 months and relative clinical stability but residual symptoms (PANSS-N ≥ 15). Pts randomly assigned to selegiline (5–15 mg/day × 8 wks) or plac, added on ongoing AP. Pts assessed at wks 1, 2, 3, 4, 6, and 8 at Rambam-Technion, after which selegiline/plac were discontinued. Pts continued AP for other 8 wks (assessment at wks 10, 12, and 16). Assessments with PANSS, BPRS, CGI, HAM-D, and AIMS	A statistically significant improvement was found in both treatment groups during the first 8 wks of the study, with a x^-^ 4.0 ± 3.5 ↓ and 4.0 ± 5.5 on the PANSS-N scores in the selegiline and plac groups. Improvement almost completely lost in both groups during the additional 8 wks of FU after selegiline discontinuation.	These studies found improvement of negative symptoms without significant aggravation in positive effects after selegiline addition and thus support the hypothesis that negative symptoms may be ameliorated via augmentation of dopamine activity.
Hale et al., 2000 [51]	89 sites across Europe (France, Belgium, Germany, the U.K., Switzerland, the Netherlands, Finland, Denmark, Norway, and Austria)	617 pts aged 18–65 years with DSM-III-R SCZ. 595 pts included in ITT analysis; 116 (83 ♂, 33 ♀; x^-^ age 34.2 years, range 19–66 years) randomized to 8 mg/day sertindole, 120 (84 ♂, 36 ♀; x^-^ age 34.3 years, range 18–64 years) to 16 mg/day sertindole, 121 (72 ♂, 49 ♀; x^-^ age 35.2 years, range 17–65 years) to 20 mg/day sertindole, 115 (77 ♂, 38 ♀; x^-^ age 35.0 years, range 18–59 years) to 24 mg/day sertindole, and 123 (84 ♂, 39 ♀; x^-^ age 36.5 years, range 18–65 years) to 10 mg/day HAL	Multicenter, DB, RCT. Pts randomized to sertindole 8 mg/day, 16 mg/day, 20 mg/day, 24 mg/day, or HAL 10 mg/day × 56 days. Efficacy assessed with PANSS and CGI	Sertindole 16 mg > efficacious than HAL 10 mg against negative symptoms (x^-^ ΔPANSS-N score ↓ = −9.0 for sertindole 16 mg/day vs. −6.6 for HAL 10 mg/day.	Sertindole at 16 mg/day is optimally effective in improving negative symptoms of SCZ. No additional benefits by ↑ dose > 16 mg/day.
Berk et al., 2001 [52]	Johannesburg, South Africa	30 inpts, 25 ♂, 5 ♀; x^-^ age = 29.5 ± 9.3, 27 single, 2 married, 1 divorced; 3 employed, 27 unemployed; FEP and recurrent illness with DSM-IV SCZ, normal laboratory examinations, no other psychotropic medications than benzodiazepines after washout	DB, PC, RCT of HAL 5 mg/day + Mirtazapine 30 mg/day vs. HAL 5 mg/day + plac × 6 wks at Witwatersrand University. PANSS, CGI-S and -I, HAM-D, and SAS at BL and 2, 4, and 6 wks, differences analyzed through ANCOVA, Mann–Whitney *u*-test, and Fisher’s exact test	Effects on negative symptoms: −42% reduction in negative symptoms in the mirtazapine group; PANSS-N scores at wk 6: Mirtazapine: 13.9 ± SD; Plac: 23.9 ± SD 5.6 (*p* < 0.0001).	Mirtazapine added to HAL significantly improved negative symptoms of SCZ compared to plac.
Feldman et al., 2003 [53]	35 sites in 7 European countries, South Africa, and U.S.	39 pts aged 50–65 years, 19 randomized to RISP (8 ♂, 11 ♀; x^-^ age = 57.2 ± 4.5) and 20 to OLA (14 ♂, 6 ♀, *χ*^2^, *p* = 0.079; x^-^ age = 56.9 ± 4.1). Most were outpts (71.8%), 92.3% white, 56.4% ♂, 43.6% ♀. SCZ (82.1%) or SCAD (17.9%). PANSS-N 27.7 for RISP, 26.4 for OLA	DB, parallel-group RCT of RISP 4–12 mg/day vs. OLA 10–20 mg/day × 28 wks. PANSS, SANS, CGI-S, BARS, and AIMS at BL and every wk × 8 wks → every 4 wks for wks 9–18	At 8 wks OLA ↓ PANSS-N > than RISP (−8.8 vs. −4.9, *p* = 0.032). At 28 wks: OLA continued to perform better than RISP on PANSS-N (−8.1 vs. −3.5, *p* = 0.032), SANS affective flattening (−5.2 vs. −0.6, *p* = 0.033), and SANS alogia (−3.8 vs. −0.3, *p* = 0.007).	OLA more effective in controlling negative symptoms of SCZ than RISP. Both drugs had approximately equal efficacy in controlling positive symptoms. Lead and other authors with Lilly affiliations and were stockholders.
Zoccali et al., 2004 [54]	Messina and probably Reggio Calabria, Italy	24 outpts with DSM-IV SCZ (15 ♂, 9 ♀), aged 21–53 years. Persistent negative symptoms despite adequate CLZ trial. Randomizeed to add-on mirtazapine (6 ♂, 4 ♀; x^-^ age 30.7 ± 6.5 yars), add-on plac (7 ♂, 3 ♀; x^-^ age 33.4 ± 9.0 yeaers). x^-^ DoI, mirtazapine 9.6 ± 4.9a years, pla 13.6 ± 7.2 years	DB, PC, RCT of mirtazapine 30 mg/day vs. pla × 8 wks. Pts on stable doses of CLZ (150–650 mg/day) and with HAM-D ≤ 20. BPRS, SAPS, and SANS at BL and 2, 4, and 8 wks	↓SANS total scores with mirtazapine compared to plac (*p* < 0.01) and SANS subscales: avolition/apathy (*p* < 0.05) and anhedonia/asociality (*p* < 0.05).	Mirtazapine augmentation to CLZ significantly improved negative symptoms in SCZ.
Duncan et al., 2004 [55]	New York, U.S.	22 ♂ with SCZ, x^-^ age 51.8 ± 12.0 years with prominent negative symptoms. Stabilized on FGAs × ≥ 60 days	DB, PC, parallel-group RCT of 50 mg/day of DCS vs. plac × 4 wks. Stable APs doses throughout the trial. Concomitant medications for side effects allowed if stable for 2 wks. SANS, BPRS or ATRS, SAS at BL and weekly until wk 4. SSTMST and CPT at BL and 2 and 4 wks	Both DCS and plac → negative symptom improvement over 4 wks. No significant differences between DCS and plac groups on SANS, BPRS, or ATRS scores or on cognitive tests.	DCS (50 mg/day) did not show a significant advantage over plac in improving negative symptoms. Longer studies and larger samples are required to detect small-to-medium effects.
Jockers-Scherübl et al., 2005 [56]	Berlin, Germany	29 pts with chronic SCZ (14 men, 15 women, x^-^ age 39.8 years, range 21–64 years). x^-^ DoI 10.0 years (range 2–20 years). Stable on APs × ≥6 months. 4 pts withdrew before BL assessment. Randomized to stable APs + paroxetine: 4 ♂, 7 ♀, x^-^ age: 40.0 ± 6.8 years; x^-^ DoI 9.9 ± 8.3 years. Stable APs + plac: 8 ♂, 6 ♀; x^-^ age: 40.8 ± 11.8 years, x^-^ DoI 9.9 ± 7.4 years	DB, PC, RCT of paroxetine 20 mg/day × 4 wks → 30 mg/day × 8 wks vs. plac added on unchanged AP × 12 wks at Charité, Berlin. Assessment through PANSS, HAM-D, CGI, BARS, SAS, and AIMS at BL and wks 2, 4, 6, 8, 10 and 12	Add-on paroxetine ↓ PANSS-N at 12th week: Paroxetine: 23.09 ± 5.47; plac: 25.36 ± 6.86; Paroxetine ↓ affective blunting from 5.18 ± 1.07 to 3.36 ± 0.81 vs. 4.57 ± 0.93 to 4.07 ± 1.27 plac (*p* = 0.009), impaired abstract thinking from 4.18 ± 0.98 to 3.27 ± 1.01 vs. from 2.93 ± 0.83 to 2.64 ± 1.08 plac (*p* = 0.026), and lack of spontaneity and flow of conversation from 4.73 ± 1.19 to 3.45 ± 0.69 vs. 4.14 ± 1.10 to 3.71 ± 1.14 plac (*p* = 0.018); Paroxetine ↓ CGI-S from 5 to 3 vs. plac from 5 to 4; no change in HAM-D.	Paroxetine added to antipsychotics is tolerable and effective in the treatment of primary negative symptoms of SCZ.
Bodkin et al., 2005 [57]	Belmont, MA, and Glen Oaks and Queens, NY, U.S.	67 outpts with SCZ with SANS summary score ≥ 12, with at least two global subscale scores ≥ 3; AP treatment ≥ 1 year at current dose ≥ 1 month, with any other psychotropic at a constant dose for ≥1 month. Exclusion criteria: severe positive symptoms at BL; treatment within 1 month from screening with ADs; current diagnosis of major mood disorder or SUD. Selegiline augmentation, 28 ♂, 5 ♀, x^-^ age: 38.0 ± 9.0 years; plac augmentation, 28 ♂, 6 ♀; x^-^ age: 39.9 ± 8.7 years	DB, PC, RCT 3-site 1:1 randomization to add-on oral selegiline 5 mg bid or plac × 12 wks at MacLean, Boston, MA AND Hillside and Creedmoor, NY. BL and FU assessments through SANS and BPRS at wks 1, 2, 4, 6, 8, 10, 12	Add-on Selegiline ↓ SANS total, and global Avolition-Apathy, and Anhedonia global scores. SANS total Selegiline group: BL: 16.40 ± 3.69; end-point 12.80 ± 3.71; Plac: BL: 16.20 ± 3.15; end-point: 13.90 ± 4.15.	Low-dose selegiline augmentation of antipsychotic medication improved significantly negative symptoms.
Riedel et al., 2005 [58]	Munich, Germany	44 pts with DSM-IV/ICD-10 SCZ, CGI score ≥ 4, predominantly primary PANSS negative symptoms. Outpts or partially or intermediately hospitalized inpts. Randomized to QUET, 22 (15 ♂,7 ♀, x^-^ age: 30.6 ± 10.9 years; final number = 19; 13 ♂, 6 ♀; x^-^ age = 29.2 ± 10.7 years) or RISP, 22 (12 ♂, 10 ♀; x^-^ age: 39.3 ± 12.3 years; final number = 15; 8 ♂, 7 ♀; x^-^ age = 39.6 ± 12.4 years, older, *p* = 0.012)	DB, parallel RCT 1:1 to QUET and RISP in pts with predominantly negative symptoms × 12 wks to oral QUET 50 mg on day 1, 100 mg on day 2, → daily 100 mg ↑ to 600 mg/day on day 7. Oral RISP 2 mg/day on days 1 and 2, → to 4 mg/day on days 3–5 and to 6 mg/day on days 6 and 7. Maximum doses allowed: 800 mg/day QUET and 8 mg/day RISP. FU assessment at BL and wks 1–12 weekly with PANSS, SANS, and SAS. 13 QUET pts and 12 RISP completed the study	34 of 44 pts continued treatment, 10 dropped-out (4 randomized to QUET and 6 to RISP). Besides being younger, pts on QUET were more severe on PANSS-N than pts on RISP and also on the SANS alogia and avolition/apathy at BL. By wk 12: QUET ↓ PANSS-N by –12.8 from 31.0 at BL, RISP by –4.2 from 25.7 at BL. No significant differences between QUET and RISP on PANSS subscales. QUET ↓ from BL to wk 12 SANS total (*p* < 0.001), affective nlunting (*p* < 0.001), alogia (*p* < 0.001), avolition (*p* < 0.001), anhedonia (*p* < 0.001), and disturbance of attention (*p* < 0.01) scores, whereas RISP ↓ from BL to wk 12 SANS total (*p* < 0.01), avolition (*p* < 0.01), anhedonia (*p* < 0.01), and disturbance of attention (*p* < 0.01). Both drugs improved clinical scores on all scales with no intergroup differences, but QUET improved more than RISP the SANS alogia and affective blunting subscales.	QUET showed equivalent efficacy and superior tolerability to RISP in the treatment of SCZ with predominantly negative symptoms; QUET ↓ negative symptoms more than RISP. The study was sponsored by the manufacturers of QUET. Pts in the two groups differed on several measures at BL. This may had obscured possible intergroup differences.
Olié et al., 2006 [59]	26 sites from Western Europe	123 pts with primary chronic DSM-III-R SCZ. Inclusion criteria: BL scores on PANSS-N ≥6 than PANSS-P scores. Exclusion criteria: acute exacerbation of SCZ or psychosis 12 wks before screening, history of psychosurgery, or any severe medical illness. 2 groups: 60 Ziprasidone: 41 ♂, 19 ♀, x^-^ age: 39.4 years. n = 42 completers; 63 Amisulpride, 38 ♂, 25 ♀; x^-^ age: 38.2 years. n = 50 completers. 31 pts discontinued due to low clinical response, TEAEs, treatment-unrelated AEs, or withdrawal of consent	DB, 1:1 randomized RCT × 12 wks to ziprasidone (40, 60, or 80 mg b.i.d.) or amisulpride (50 or 100 mg b.i.d.). The minimum interval between dosage titration steps was 1 week. FU assessment through PANSS, BPRS, and CGI-S at BL and wks 4, 8, and 12; CGI-I at wks 4, 8, and 12; BARS, SAS, and AIMS at BL and wks 6 and 12; and MDBS during study or within 6 days from treatment cessation	Primary efficacy variable: ↓ PANSS-N from BL in ziprasidone and amisulpride groups. Response rates for negative-symptom improvement, defined as ≥20% ↓ in PANSS-N, were similar for ziprasidone and amisulpride-treated pts. Attrition rate higher in the ziprasidone group.	Amisulpride and ziprasidone showed similar efficacy and safety. Two last authors with the affiliation of the manufacturers of ziprasidone.
Álvarez et al., 2006 [60]	21 sites in Spain	247 outpts aged 18–65 years with DSM-IV SCZ, prominent negative symptoms (SANS summary score ≥ 10), and previously treated with FGAs (OLA n = 124: 85 ♂, 39 ♀, x^-^ age 37 ± 10, SANS summary score 14.3 ± 3.1. RISP n = 123, 94 ♂, 29 ♀; x^-^ age 35.5 ± 10.6, SANS summary score 14.2 ± 3.1)	RCT, monitored, open-label, parallel, flexible-dose, 1:1 random assignment to initial OLA ≥ 10 mg/day or RISP ≥ 3 mg/day × 12 months. Primary efficacy measure: SANS summary score. Response rate defined as ≥30% ↓ on SANS summary score; SANS, CDRS, and CGI-S weekly until wk 24 → bi-montly until wk 48	At 1 year: OLA pts showed significantly > improvement than RISP pts on SANS summary (*p* = 0.015) and on affective flattening (*p* = 0.007) and avolition/apathy (*p* = 0.028) SANS subscales.	Long-term treatment with OLA associated with significantly better improvement in negative symptoms as compared with RISP in outpts with SCZ and prominent negative symptoms. Study partially funded by OLA manufacturers, who controlled data collection.
Sirota et al., 2006 [61]	Bat Yam–Tel Aviv-Yafo, Israel	40 pts with SCZ and Carpenter’s deficit syndrome (♂ 32, ♀ 8; x^-^ age = 37.20) randomized to QUET (N = 19, ♂ 15, ♀ 4; x^-^ age = 38.3 ± 12.2 years, x^-^ DoI 15.9 ± 9.1) or OLA (N = 21, ♂ 17, ♀ 4; x^-^ age = 36.2 ± 10.9 years, x^-^ DoI 13.3 ± 7.4)	DB RCT of QUET 200–800 mg/day vs. OLA 5–20 mg/day at Abarbanel Mental Health Center, Bat Yam × 12 wks. Inpts were TRS with deficit and PANSS-N > 15 and SANS > 60. Assessments with SANS, PANSS, SAS, BARS, and AIMS at BL and 1, 2, 4, 8, and 12 wks	Both QUET and OLA ↓ SANS total and subscale scores and PANSS-N, PANSS-T, and PANSS-P scores. No significant changes in SAS, BARS, and AIMS scores.	Both QUET and OLA treatments improved negative symptomatology in pts with SCZ whose negative symptoms were refractory to drug treatment, with small differences between the two drugs. Sponsored by QUET manufacturers.
Kinon et al., 2006 [62]	Multicenter (N = 26) (Georgia, New Hampshire, Maryland, Florida, Ohio, Illinois, New York, Texas, California, Connecticut, Washington DC, Alabama, and Pennsylvania), U.S.	346 pts with SCZ (♂ 228, ♀ 118) and prominent negative symptoms and social impairment allocated to OLA (N = 171; ♂/♀ = 66.7/33.3; x^-^ age = 41.67 ± 9.53 years, x^-^ DoI 17.57 ± 9.65) or QUET (N = 175; ♂/♀ = 65.1/34.9; x^-^ age = 40.45 ± 9.61 years, x^-^ DoI 17.78 ± 9.39)	DB RCT of 10–20 mg/day OLA vs. 300–700 mg/day QUET in 26 U.S. sites in the community; pts received add-on OLA or QUET × 2 wks and then tapered-off their AP treatment up to randomization and dose-optimisation. QUET and OLA were taken for further 22 wks. Assessments with SANS, PANSS, CGI, CDRS, GAF, SAS, BARS, and AIMS at BL and endpoint	OLA better than QUET on PANSS-P, CGI-S, CGI-I, and GAF; less drop-outs with OLA than with QUET. However, OLA could not supersede QUET on negative symptoms, as assessed with SANS and PANSS, although both treatments obtained score reductions.	OLA is similar to QUET on the negative symptoms of SCZ in pts with prominent negative symptoms; final conclusions: “Greater improvement in positive symptoms and a greater study completion rate may hold relevance to enhanced functional outcomes observed after OLA therapy”. Sponsor bias may be present in such conclusions. Sponsor-affiliated senior author.
Lecrubier et al., 2006 [63]	Paris, France	244 pts with DSM-IV residual, disorganized, or catatonic SCZ (166 ♂, 78 ♀) SANS summary ≥ 10 and PANSS ≤ 4 on each PANSS-P item “delusions” or “hallucinations” randomized to OLA 5 (60.0% ♂, x^-^ age = 38.1 ± 11.1; x^-^ DoI = 121 months) or 20 mg/day (74.3% ♂, x^-^ age = 36.4 ± 10.4; x^-^ DoI = 133 months) or amisulpride 150 mg/day (71.4% ♂, x^-^ age = 37.8 ± 11.6; x^-^ DoI = 148 months) or plac (64.7% ♂, x^-^ age = 38.2 ± 9.0; x^-^ DoI = 185 months; *p* = 0.002)	DB RCT randomized 2:2:2:1 to 5 mg/day OLA, 20 mg/day OLA, 150 mg/day amisulpride or plac at Hôpitaux Universitaires Pitié Salpêtrière × 6 months; assessment at BL and wks 1, 2, 3, 4, 5, 6, 10, 14, 18, 22, and 24 with SANS, PANSS, BPRS, CGI, PDS, SAS, BARS, and AIMS	Improvements did not differ between the groups on any scale; overall, pts in every group responded positively to the treatment. There were more responders in the OLA 5 mg/day than in the plac group; however, DoI was significantly ↑ in the plac group and ↓ in the two OLA groups.	Three of five authors affiliated at sponsor. However, no favourable results for the sponsor were obtained. DoI longer in the plac group. The only positive result regarding OLA 5 mg/day responder rate > plac responder rate could be attributed to ↑ DoI in the latter. In fact, more chronic SCZ is less responsive to therapeutic interventions.
Lindenmayer et al., 2007 [64]	New York, NY, U.S. (1 site)	35 pts with SCZ, Kirkpatrick’s deficit syndrome, and PANSS depression item < 4 randomized 1:1 to OLA (N = 16, 14 ♂, 2 ♀; x^-^ age = 39.02 ± 10.48) or HAL (N = 19, 19 ♂, 0 ♀; x^-^ age = 39.77 ± 9.49)	DB 1:1 RCT of 15 mg/day OLA vs. 15 mg/day HAL × 6 wks → OLA flexible vs. flexible HAL × 6 wks at Manhattan Psychiatric Center; pts on HAL took benztropine and those on OLA benztropine-plac; assessment at BL and wks 1, 2, 4, 6, 8, 10, and 12 with PANSS, CGI, HAM-D, SAS, and AIMS and neurocognitive assessment	OLA better than HAL on PANSS-N and PANSS-T, as well as on some neurocognitive measures; OLA linked to ↑ weight gain with respect to HAL.	OLA may ↓ negative symptoms and improve cognition. Sponsored by OLA manufacturers; small sample.
Ruhrmann et al., 2007 [65]	27 German and 3 Austrian centers	144 pts with ICD-10 SCZ × ≥ 2 years and ≥3 PANSS-N items scoring ≥ 4 randomized to flupentixol (N = 72; 45 ♂, 27 ♀; x^-^ age = 40.94 ± 12.84; x^-^ DoI = 11.28 ± 9.98 years) or RISP (N = 72; 45 ♂, 27 ♀; x^-^ age = 39.83 ± 11.13; x^-^ DoI = 11.50 ± 10.07 years)	DB, multicenter RCT in Germany and Austria of flupentixol 4–12 mg/day vs. RISP 2–6 mg/day × 25 wks. Assessment with PANSS, MADRS, and ESRS at BL and 1, 2, 4, 8, 12, 16, 20, and 24 wks; CGI at BL and 8, 16, and 24 wks	Both drugs improved negative symptoms with no differences; RISP more tolerable than flupentixol (more biperiden needed in the flupentixol group).	Non-inferiority study. Both drugs may be useful in treating negative symptoms.
Pierre et al., 2007 [66]	Los Angeles, CA, U.S.	20 pts with DSM-IV SCZ or SCAD, randomized to modafinil (N = 10; 10 ♂, 0 ♀; x^-^ age = 49.7 ± 6.8 years) or plac (N = 10; 9 ♂, 1 ♀; x^-^ age = 49.8 ± 7.0 years)	DB treatment with modafinil (100 mg/day, possibly titrated to 200 mg/day) or plac × 8 wks; assessment with 18-item SANS, BPRS, CGI, SAS, BARS, and AIMS at BL and wks 2, 4, 6, and 8	Modafinil did not improve SANS-assessed negative symptoms.	No effect of adjunctive modafinil on negative symptoms but may benefit other symptoms.
Buchanan et al., 2007 [67]	4 centers in the U.S. (New York, NY [2]; Baltimore, MD; Los Angeles, CA) and 1 in Israel (Jerusalem)	157 inpts and outpts with DSM-IV SCZ or SCAD and retrospective and prospective criteria for moderate-to-severe negative symptoms without marked positive, depressive, or EPS; randomized to glycine (N = 52, x^-^ age = 42.6 ± 10.8 years), DCS (N = 53, x^-^ age = 44.4 ± 10.4 years) and plac (N = 52, x^-^ age = 43.4 ± 11.4 years); sex not reported	16-week DB, double-dummy, parallel-group RCT of adjunctive glycine, DCS, or plac; assessment with SANS, BPRS, and CGI using mixed-model analysis at BL (two points 4 wks apart) and 4, 8, 12, and 16 wks	There were no significant differences in change in the SANS total score between glycine and plac subjects or DCS and plac subjects.	Neither glycine nor DCS are effective therapeutic options for treating negative symptoms. Gender not reported (but did not vary for the three groups across participating sites).
Amiri et al., 2008 [68]	Tehran, Iran (3 sites)	40 pts with chronic SCZ, in the active phase of the illness, PANSS-T ≥ 60 and PANSS-N ≥ 15; N = 20 (14 ♂, 6 ♀; x^-^ age = 32.1 ± 6.1 years) randomized to selegiline and N = 20 (15 ♂, 5 ♀; x^-^ age = 33.65 ± 7.27 years) to plac in add-on to ongoing RISP	DB, PC, RCT to RISP (N = 20) 6 mg/day plus selegiline 10 mg/day (5 mg bid) and RISP 6 mg/day plus plac (N = 20) × 8 wks; assessment with PANSS at BL and each 2 wks and ESRS at days 7, 14, 28, 42, and 56	RISP-selegiline combination showed significant superiority over RISP alone in ↓ negative symptoms.	Selegiline is a potential adjunctive treatment strategy for the negative symptoms of SCZ.
Goff et al., 2008 [69]	Boston, MA, U.S.	38 SCZ outpts treated with Aps and not CLZ (age range 18–65 years) (♂ 23, ♀ 15) were randomly assigned to plac (N = 19, ♂ 13, ♀ 6) (x^-^ age = 48.0 ± 6.66 years) or to DCS (N = 19, ♂ 10, ♀ 9) (x^-^ age = 50.1 ± 9.15 years)	DB, PC, prospective RCT, FU study. Pts randomized to oral DCS (50 mg/wk) vs. plac add-on to APs × 8 wks. Psychometric assessment at BL and 8 wks: PANSS, SANS, CGI and cognitive battery. SAFTEE once weekly	SANS total score ↓ at wk 8 in the DCS group compared to plac (*p* = 0.048). ΔSANS total score change did not correlate with age, gender, AP type (FGA or SGA), BL SANS, or BL PANSS-T.	DCS 50 mg/wk × 8 wks significantly ↓ negative symptoms compared to plac in SCZ pts treated with a variety of APs. No side effects reported.
Akhondzadeh et al., 2008 [70]	Tehran, Iran	40 SCZ inpts (age range 19–45 years) (♂ 25, ♀ 15) were randomly assigned to RIS 6 mg/day + plac (N = 20, ♂ 12, ♀ 8) (x^-^ age = 33.05 ± 6.98 years) or to RIS 6 mg/day + Ritanserin 12 mg/day (6 mg bid) oral (N = 20, ♂ 13, ♀ 7) (x^-^ age = 32.05 ± 5.96 years)	DB, PC, prospective RCT, FU study. Pts randomized to RIS 6 mg/day + plac or to RIS 6 mg/day + Ritanserin 12 mg/day (6 mg bid) × 8 wks. Psychometric assessment at BL and after 8 wks of treatment: PANSS and ESRS	Ritanserin ↓ PANSS-N from BL to wk 8 more than plac (*p* < 0.05) and was associated with less days of biperiden treatment (*p* = 0.03).	Ritanserin (6 mg bid) was a potential adjuvant treatment strategy to treat negative symptoms of SCZ. Ritanserin-treated pts showed significantly ↓ of EPS.
Marx et al., 2009 [71]	Durham, NC, U.S.	18 SCZ or SCAD outpts treated with SGAs (age range 18–65 years) (♂ 17, ♀ 1), were randomly assigned after 2-week single-blind plac lead-in to oral PREG (fixed escalating doses to 500 mg/day; N = 9, ♂ 8, ♀ 1, x^-^ age = 52.68 ± 6.31 years) or to oral plac twice daily (N = 9, ♂ 9, ♀ 0, x^-^ age = 49.43 ± 12.19 years)	DB, PC, prospective RCT, FU study. Pts randomized to PREG or plac twice daily × 8 wks. Visit 1: 2-week single-blind plac lead-in phase × 2/day (all pts). Visit 2: Randomization to PREG 50 mg × 2/day (100 mg/day) or plac × 2 wks. Visit 3: PREG 150 mg × 2/day or plac × 2 wks. Visit 4: PREG 250 mg × 2/day or plac × 2 wks. Visit Visit 5: PREG 250 mg × 2/day or plac × 2 wks. Psychiatric symptoms assessed at BL and at endpoint (Visit 6) using SANS, PANSS, CDRS, CGI-I, and CGI-S. Side effects assessed with BARS and SAS. Cognitive symptoms assessed by BACS and the MCCB	Pts randomized to PREG ↓ SANS scores compared to plac (*p* = 0.048). Pts randomized to PREG showed significantly greater improvements in the SANS affect subscale (*p* = 0.035) and a trend for the SANS alogia subscale (*p* = 0.087) in PREG.	Treatment with adjunctive PREG significantly reduced negative symptoms as assessed by SANS scores in pts with SCZ or SCAD compared with plac. No pt receiving PREG experienced serious AEs.
Iancu et al., 2010 [72]	Be’er Ya’akov, Israel	40 pts, 20 (75% ♂; x^-^ age 35.5 ± 8.7 years) with escitalopram (5 mg/day × 3 days, 10 mg/day × 4 wks and 20 mg/day thereafter to achieve maximum effect) and 20 pts (70% ♂; 38.8 ± 6.88 years) with plac × 10 wks. 2 pts withdrew consent in the first weeks; hence, 38 pts were included in the efficacy analyses (including those who withdrew due to side effects)	DB, PC, RTC. Inclusion: age 18–60 years, diagnosis of chronic SCZ, PANSS-T score of ≥50 and stable treatment with APs. Exclusion: axis I comorbidity (MDD and mania), pregnancy, lactation, impaired renal/hepatic function, and history of sensitivity to SSRIs. Pts assessed with CGI, SFS, PANSS, SANS, HAM-D, and AIMS. Side effects as reported every 2 weeks. Physical examination at BL and end of study. Vital signs × 2/month and blood and urine before entry, after 5 wks, and last wk	↓ in PANSS-N was 5% for escitalopram and 10% for plac (n.s.). No superiority for escitalopram over plac on PANSS-T (F_(6,216)_ = 1.44, n.s.), PANSS-P (F_(6,216)_ = 0.84, n.s.), PANSS-N (F_(6,216)_ = 1.22, n.s.), the PANSS-G (F_(6,216)_ = 1.45, n.s.), SANS (F_(6,216)_ = 0.36, n.s.), CGI (F_(6,216)_ = 1.18, n.s.), SFS (F_(6,216)_ = 0.78, P = n.s.), HAM-D (F_(6,216)_ = 0.31, n.s.), and AIMS (F_(6,216)_ = 1.39, n.s.).	Escitalopram does not improve negative symptoms in pts with chronic SCZ vs. plac.
Stahl et al., 2010 [73]	40 centers in the U.S. and Canada	599 pts randomized 3:3:2 to 2 flexibly-dosed ziprasidone regimens (ZSTD, 80–160 mg/day in 2 divided doses, n = 227; or ZLOW, 80–120 mg/day dosed once, n = 221) or HAL, 5–20 mg/day (n = 151); sex not reported	40-week DB RCT of ZSTD/ZLOW vs. HAL → 3-year DB extension trial. Inclusion: DSM-III-R SCZ or SCAD; no hospitalization for ≥12 wks before screening; and PANSS-N ≥ 10; PANSS for SCZ symptoms, MADRS for depression and SARS for EPS at BL, 40 wks, and 3 years	HAL took longer to remit negative symptoms. ZSTD remitted better negative symptoms than HAL during the 156-week DB extension (*p* = 0.036) independently from PANSS-P (*p* = 0.012), MADRS (*p* = 0.037), and SARS (*p* = 0.546) score mediation. Early improvement in PANSS-T (920% from BL to wk 40) predicted negative symptom remission during the 3-year extension.	The SGA ziprasidone (80–160 mg/day in two divided daily doses) seems to be better than the conventional FGA HAL during the 196-week DB study period.
Akhondzadeh et al., 2011 [74]	Tehran, Iran	40 pts with chronic SCZ (36 ♂, 4 ♀), aged 18–45 years; 20 sildenafil (75 mg/day) + RISP (6 mg/day) (18 ♂, 2 ♀, x^-^ age 34.12 ± 8.36 years); 20 plac + RISP (6 mg/day) (18 ♂, 2 ♀, x^-^ age 32.60 ± 8.33 years) × 8 wks	RCT, DB. Inclusion: DSM -IV-TR SCZ; ≥60 PANSS-T. No APs for 1 wk or LAIs ≥ 2 months prior to trial. Pts excluded if with significant organic and neurological disorders, psychotic disorders other than SCZ, use of drugs contraindicated with sildenafil, ADs ≥1 month from screening, and current major mood disorder or SUD	Both groups improved on all PANSS scores; Sildenafil add-on ↓ PANSS-N and PANSS-T more than plac add-on (*p* < 0.001).	Sildenafil may improve negative symptoms in pts with SCZ when added on RISP.
Cho et al., 2011 [75]	Bundang, Republic of Korea	20 pts (aged 21–70) with SCZ randomized to RISP + mirtazapine (N = 11; 5 ♂, 6 ♀; x^-^ age = 35.08 ± 13.58 years) or RISP + plac (N = 9; 5 ♂, 4 ♀ x^-^ age = 36.44 ± 9.57 years)	RCT, DB treatment with RISP (2–4 mg/day) and mirtazapine (15–30 mg/day) or plac × 8 wks; assessments with PANSS, SANS, and RBANS at BL and wks 2, 3, and 8	The RISP + mirtazapine group exhibited significant ↓ in SANS score at wks 2, 4, and 8 compared with plac (*p* < 0.001). The RISP + mirtazapine group exhibited a statistically significant improvement in cognitive function, including vocabulary and immediate memory, and showed AEs (x^-^ ↑ 5.83 kg weight).	Augmentation RISP with mirtazapine can effectively improve both negative and some cognitive symptoms of SCZ.
Xiao et al., 2011 [76]	Shanghai, China	80 pts (≥18 years; 39 ♂, 41 ♀) randomized to sarsasapogenin + RISP (N = 41, x^-^ age = 46.02 ± 17.23 years) or to plac + RISP (N = 39, x^-^ age = 55.21 ± 15.74 years)	DB, PC, parallel-group RCTS with sarsasapogenin (200 mg/day) or plac added-on to flexible (2–4 mg/day) RISP × 8 weeks; assessments with PANSS, WMS, mWAIS, and CGI at BL and wk 8	Sarsasapogenin + RISP group showed no significant difference in changes of PANSS, WMS, or mWAIS scores at wk 8 compared with plac + RISP. The incidence of TEAEs in pts treated with sarsasapogenin was not different from that observed in plac group.	Sarsasapogenin did not significantly augment the effects of RISP in treating cognitive deficits of pts with negative symptom-dominated SCZ and did not affect symptom severity.
Kane et al., 2012 [77]	39 study centers across the U.S.	285 pts (aged 18–65 years) randomized to three dosages of armodafinil (150 mg/day N = 71, x^-^ age = 43.7 ± 11.19, 53 ♂, 18 ♀; 200 mg/day: N = 70, x^-^ age = 43.1 ± 11.07, 57 ♂, 13 ♀; 250 mg/day: N = 72, x^-^ age = 44.4 ± 9.43 years, 50 ♂, 22 ♀) or plac (N = 72, x^-^ age = 42.4 ±10.07 years, 46 ♂, 26 ♀)	PCT, DB study with armodafinil at three dosages (150 mg; 200 mg; 250 mg) or plac × 24 weeks; assessments with PANSS negative symptoms subscale at BL and wks 1, 2, 4, 6, 8, 10, 12, 14, 16, 18, 20, 22, and 24	PANSS negative symptom subscale showed no significant difference between adjunctive armodafinil compared with plac at any timepoint (*p* ≥ 0.70 for each armodafinil group versus plac).	No evidence of benefit of adjunctive armodafinil compared with plac for the treatment of negative symptoms in pts with SCZ.
Buchanan et al., 2012 [78]	20 countries: 15 Eastern Hemisphere (72 sites in Europe, Australia, and South Africa) and 5 American (95 sites in North and South America)	949 pts (≥18 years) were randomized to asenapine (N = 485; 340 ♂, 145 ♀, age range 18–75 years, x^-^ age = 41.91 years) or OLA (N = 464; 334 ♂, 130 ♀, age range 18–80 years, x^-^ age = 41.51 years); SDs impossible to calculate	RCT, DB study with asenapine (10–20 mg/day) or OLA (10–20 mg/day) × 26 weeks; results of two studies, one in Eastern Hemisphere, the other in the Western. Assessments with NSA-16 (primary outcome), CGI, CDRS, and PANSS (secondary outcomes) at BL and wks 4, 12, and 26	Both treatments ↓ NSA-16 and PANSS-N scores, but there were no significant between-group differences on the primary outcome measure; OLA better than asenapine on the CDRS in the Eastern study. OLA better than asenapine on PANSS-P in both Eastern and Western studies at various timepoints. No differences on CGI-S or -I. Response rates (tailored on the CGI) did not differ between OLA and asenapine.	Asenapine superiority over OLA was not observed. The study was partially co-funded by both drugs’ manufacturers.
Xiao et al., 2012 [79]	Shanghai, China	101 pts (aged ≥18) with SCZ treated with RISP and randomized to cerebrolysin (N = 52; x^-^ age= 47.19 ± 18.06 years; 41 ♂, 11 ♀) or plac (N = 49; x^-^ age = 48.90 ± 14.46 years; 34 ♂, 15 ♀)	DB, PC, parallel-group study with cerebrolysin (250 mL/die) or plac × 8 weeks; assessment with PANSS at BL and wks 2, 4, and 8	Similar improvements between pts treated with cerebrolysin added to RISP and those treated with RISP alone in terms of PANSS total and negative scores at wk 8.	Augmentation with cerebrolysin 30 mL daily does *not* enhance the efficacy of RISP in treating the negative symptoms of SCZ.
Chaudhry et al., 2012 [80]	6 sites: Pakistan (1 Rawalpindi and 4 Karachi) and Brazil (São Paulo, ≈20%)	198 pts (aged 18–65 years) with a DSM-IV SSD. 198 pts eligible; 54 excluded, 144 pts randomized to minocycline (200 mg/day) + AP (41 ♂, 30 ♀; x^-^ age 25.87 ± 7.07 years) or plac + AP (45 ♂, 28 ♀; x^-^ age 26.59 ± 8.26 years)	DB, RCT, PC multicenter study of add-on minocycline vs. plac × 1 year. Inclusion: DSM -IV-TR SSD with onset ≤ 5 years, treated with AP and stabilized × 4 wks. Exclusion: major medical illness, pregnancy, hypersensitivity to minocycline, concomitant penicillin or anticoagulant therapy, and SUD diagnosis. PANSS at BL and 6 and 12 months, CGI, AIMS, cognitive battery and GAF at BL and 1 year	Improved PANSS-N in both countries; improved also PANSS-P, PANSS-G and PANSS-T in Brazil more than in Pakistan. No effect on neuropsychological cognitive testing and positive effect on CGI-S with minocycline but better AIMS improvement with plac.	Minocycline add-on TAU improved negative symptoms in both countries. No significant differences in PANSS-P and CGI.
Khodaie-Ardakani et al., 2013 [81]	2 sites: Tehran and Sanandaj, Iran	82 pts eligible; 42 excluded. 40 pts with chronic SCZ (23 ♂, 17 ♀) ages 18–50 years randomized 1:1 to Granisetron + RISP (12 ♂, 8 ♀, x^-^ age 36.7 11.3 years) and plac + RISP (11 ♂,9 ♀, 37.9 9.3 years)	DB, PC, 2-center RCT of granisetron 1 mg q 12 h vs. plac. × 8 wks. Inclusion: DSM -IV-TR SCZ during ≥2 years; treated with RISP for ≥8 wks and stabilized for ≥4 wks defined as ≥20% ↓ from BL in PANSS-T. Pts excluded if with significant organic and neurological disorders, psychotic disorders other than SCZ, lactation and pregnancy, pts with a score ≥ 14 on HAM-D, or a score of ≥4 on the PANSS depression item; PANSS and ESRS at BL and 4 and 8 wks; HAM-D at BL and wk 8	Add-on granisetron ↓ PANSS-N and PANSS-T scores than plac at endpoint (*p* < 0.001). Granisetron vs. plac → no differences in PANSS-P, PANSS-G, and HAM-D scores. ESRS score ↓ significantly in the granisetron group at 4th wk and similar ESRS score at wk 8.	Granisetron add-on to RISP improves the primary negative symptoms of pts with chronic stable SCZ; no safety issues.
Noroozian et al., 2013 [82]	2 sites: Tehran, Iran	40 pts with chronic SCZ ages 18–50 years; 20 Tropisetron + RISP (16 ♂, 4 ♀, x^-^ age 33.8 ± 7.0 years); 20 plac + RISP (15 ♂, 5 ♀, 33.7 ± 5.9 years)	PC, DB, parallel-group RCT of 10 mg/day tropisetron vs. plac add-on to RISP 6 mg/day × 8 wks at Roozbeh Psychiatric Hospital and Razi Hospital. Inclusion: DSM-IV-TR SCZ during ≥2 years; treated with RISP for ≥8 wks and stabilized for ≥4 wks defined as ≤20% ΔPANSS-T. Excluded pts with ≥14 on HAM-D or ≥4 on the depression item of PANSS. Pts excluded if with significant organic and neurological disorders, psychotic disorders other than SCZ, pregnancy, hypersensitivity to tropisetron, and ECT during last 2 wks. PANSS and ESRS at BL and wks 2, 4, 6 and 8; HAM-D at BL and wk 8	Tropisetron add-on ↓ PANSS-T scores (*p* < 0.001) and PANSS-N scores (*p* < 0.001) and PANSS-G (*p* = 0.017) but not positive subscale scores compared to plac (*p* = 0.893). No differences on ESRS or HAM-D or PANSS-P.	Tropisetron add-on to RISP improved the primary negative symptoms of pts with chronic stable SCZ. No safety concerns.
Rezaei et al., 2013 [83]	2 sites: Tehran and Sanandaj, Iran	40 pts with chronic SCZ (23 ♂, 17 ♀) ages 18–50 years; randomized 1:1 to memantine (20 mg/day) + RISP (12 ♂, 8 ♀, x^-^ age 33.5 ± 6.9); 20 plac + RISP (11 ♂, 9 ♀, x^-^ age 33.0 ± 6.9)	DB, RCT, PC parallel-group study of memantine 20 mg/day vs. plac in add-on to 6 mg/day RISP × 8 wks at Tehran University of Medical Sciences and Qods Psychiatric Teaching Hospital (Kurdistan University of Medical Sciences, Sanandaj. Inclusion: DSM-IV-TR SCZ since ≥2 years; treated with RISP for ≥8 wks and stabilized for ≥4 wks. Pts with HAM-D ≥14 or ≥4 on the PANSS depression item excluded. Exclusion criteria were organic, psychiatric, or neurological disorder; ECT in last 2 wks; hypersensitivity to memantine; pregnancy. Rating through PANSS at BL and 4 and 8 wks; ESRS and HAM-D at BL and wk 8	Memantine add-on ↓ PANSS-N and PANSS-T scores at 8 wks (*p* < 0.001). PANSS-G improved more in the memantine group then in plac (*p* = 0.008); PANSS-P, ESRS, HAM-D, and adverse events did not differ between memantine and plac.	Memantine add-on to RISP improves the primary negative symptoms of pts with SCZ. No safety concerns.
Vahia et al., 2013 [84]	San Diego, CA, U.S., and Cincinnati, OH, U.S.	187 pts (145 ♂ and 42 ♀; x^-^ age 52.54; SD impossible to calculate) with SCZ or SCAD aged ≥40 and who met study criteria for SSD (subsyndromal depression), randomized to plac + AP (N = 89, 75 ♂ and 14 ♀; x^-^ age 51.6 ± 6.4) or citalopram + AP (N = 98, 70 ♂ and 19 ♀; x^-^ age 53.4 ± 7.7)	DB, PC, RCT. Pts randomly assigned to flexible-dose citalopram (20 mg/day) or plac augmentation of their current AP medication at Intervention Research Center at UCSD and Cincinnati sites and Chillicothe VA Medical Center. → 1st week, study dose could be ↓ to 10 mg/day or ↑, based on clinical response and/or adverse events (minimum–maximum doses 10–40 mg/day) × 12 wks; assessment with HAM-D and CDRS at wks 1, 2, 3, 4, 6, 8, and 12 and PANSS at wks 1, 4, 8, and 12	Significant improvement in PANSS-N scores in the citalopram group, which was partially mediated by improvement in depressive symptoms.	In pts with SCZ/SCAD, treating depressive symptoms with citalopram appears to carry the added benefit of improving negative symptoms.
Hinkelmann et al., 2013 [85]	Hamburg, Germany	51 pts (x^-^ age = 39.78, SD impossible to calculate; 34 ♂, 17 ♀) with DSM-IV SCZ and predominant negative symptoms randomized in a double-blind design to add-on treatment with citalopram, reboxetine, or plac for 4 weeks. Pts had to be on stable antipsychotic medication for at least 2 weeks before inclusion. 16 to plac + AP (x^-^ age = 38.3 ± 8.4; 9 ♂, 7 ♀), 16 to citalopram + AP (x^-^ age = 38.5 ± 7.5; 10 ♂, 6 ♀), and 19 to reboxetine + AP (x^-^ age = 42.1 ± 13.4; 15 ♂, 4 ♀)	DB RCT, PC. Pts continued their current AP, kept stable throughout the study. Pts randomized to add-on citalopram 20 mg, reboxetine 4 mg, or plac × 4 weeks. Medication could ↑ → 1 wk to 2 capsules (citalopram 40 mg, reboxetine 8 mg, or plac 2 capsules), respectively, given orally ×2/day. Assessment on days 0 (BL), 7, 14, 21, and 28 with PANSS, HAM-D, CGI, AIMS, BARS, and SAS	The main effect of treatment (reflecting different psychopathology levels between treatment groups) and the treatment time interaction (change over time in different treatment groups) were not significant (*p* = 0.10). PANSS-N responder rates did not differ between treatment groups (W2, *p* = 0.10).	No beneficial effect of adjunctive AD treatment on negative symptoms in SCZ.
Caforio et al., 2013 [86]	Bari, Italy	Inclusion criteria: SCZ with recent psychotic exacerbation requiring hospitalization. 28 white pts with SCZ (x^-^ age = 29.3 ± 7.4; 21 ♂, 7 ♀), randomized in 2 groups: 14 (plac + OLA) and 14 (mirtazapine 30 mg/day + OLA). Treatment groups did not differ for sex, age, length of illness, drug-free period before entering the trial, or OLA dose (all *p* = 0.1)	DB, PC, RCT. Starting from the first day of hospital admission, pts enrolled and treated × 8 wks with OLA in monotherapy (x^-^ = 16.5 ± 7 mg). → 8 wks, pts randomized 1:1 for further 8 wks to 30 mg/day mirtazapine (n = 14) or plac (n = 14). OLA held at stable dose. Assessment at BL (1, 4, and 8 wks) and endpoint (wk 16) with PANSS and CDRS.	Mirtazapine was significantly more effective than plac in ↓ PANSS-N scores after 8 wks of therapy; no significant differences between mirtazapine and plac in PANSS-T, PANSS-P, PANSS-G, and CDRS scores. Adverse events did not differ between groups.	Mirtazapine significantly > effective than plac in lowering negative symptoms of SCZ.
Farokhnia et al., 2013 [87]	Tehran, Iran, and Sanandaj, Kurdistan, Iran	42 pts with SCZ randomized to NAC + RISP (9 ♂, 12 ♀; x^-^ age = 32.23 ± 6.12; DoI x^-^ = 83.23 ± 41.02 months) or plac + RISP (11 ♂, 10 ♀; x^-^ age = 33.38 ± 6.97; DoI x^-^=88.95 ± 44.66 months)	DB, PC, RCT. Pts received RISP 2 mg/day × 1 wk → 4 mg/day × 1 wk → 6 mg/day × 1 wk titrated according to needs and remained constant up to wk 8; 1:1 randomization to NAC 1000 mg/day × 1 wk → NAC 2000 mg/day × 7 wks or plac at Roozbeh, Razi, and Qods Hospitals. Assessment with PANSS at BL and wks 2, 4, 6 and 8; HAM-D at BL and wk 8; TEAEs and ESRS at wks 1, 2, 4, 6, and 8	The NAC group outperformed the plac group on the PANSS-N and PANSS-T. No differences on PANSS-G and PANSS-P, HAM-D, ESRS, and TEAEs.	The addition of NAC to ongoing AP treatment (RISP) may benefit the negative symptomatology of SCZ.
Farokhnia et al., 2014 [88]	Tehran, Iran, and Sanandaj, Kurdistan, Iran	50 pts (aged 18–50 years) with chronic DSM-IV SCZ, treated with RISP and ≥20 on the PANSS, randomized to riluzole (N = 24; 21 ♂, 4 ♀; x^-^ age= 32.20 ± 6.84 years) or plac (N = 24; 22 ♂, 3♀; x^-^ age = 33.64 ± 8.00 years)	RCT, DB, PC, with riluzole (100 mg/day) or plac × 8 wks; assessment with PANSS-N at BL and wk 8	↓ PANSS-N scores significantly higher in the riluzole-treated pts than in the plac group.	Riluzole as an adjunctive treatment to RISP is efficacious in alleviating negative schizophrenic symptoms.
Khodaie-Ardakani et al., 2014 [89]	Tehran, Iran	38 outpts (aged 18–50 years) with DSM-IV SCZ and DoI ≥ 2 years, randomized to minocycline + RISP (N = 20; 14 ♂, 6 ♀; x^-^ age= 41.05 ± 7.47 years) or plac + RISP (N = 18; 15 ♂, 3 ♀; x^-^ age = 38.95 ± 7.78 years)	RCT, DB, PC, parallel-group trial with minocycline (100 mg/day × 1 wk, → 200 mg/day) or plac × 8 wks; assessment with PANSS-N at BL and wk 8	Reduction in PANSS total scores were observed in the minocycline group compared with the plac group.	Minocycline as an adjunctive treatment to RISP is effective in alleviating negative schizophrenic symptoms.
Liu et al., 2014 [90]	Yunnan Province, China	79 pts (aged 18–49 years) with DSM-IV SCZ randomized to RISP + minocycline (N = 39; 25 ♂, 14 ♀; x^-^ age = 27.05 ±5.68 years) or RISP + plac (N = 40; 24 ♂, 16 ♀; x^-^ age = 27.70 ± 7.27 years)	RCT, DB, PC with minocycline (200 mg/day) or plac × 16 wks; assessment with SANS, PANSS, CGI, and cognitive tests at BL and wk 16	Pts receiving minocycline had greater improvements on SANS and PANSS negative subscale scores (*p* < 0.001) when compared with those receiving the plac. No difference between the seven cognitive domains except for attention (*p* = 0.044).	Minocycline as an adjunctive treatment to RISP is effective in alleviating negative schizophrenic symptoms and has a slight effect on the attention domains of pts with SCZ.
Ritsner et al., 2014 [91]	Tirat Carmel, Israel	52 pts with DSM-IV SCZ randomized to PREG (N = 25; 22 ♂, 3 ♀; x^-^ age = 26.9 ± 5.2 years) or plac (N = 27; 23 ♂, 4 ♀; x^-^ age = 27.8 ± 6.0 years)	RCT, DB, PC, with PREG (50 mg/die) or plac × 8 wks; assessment with PANSS-N and SANS at BL and wk 8	Pts treated with PREG showed a significant reduction in the PANSS-N and SANS total scores compared to plac.	Add-on PREG was found to be effective in alleviating negative SCZ symptoms.
Hosseini et al., 2014 [92]	Tehran, Kurdistan, and Hamadan, Iran	36 ♂ and 8 ♀ (age range 18–50) with chronic SCZ, PANSS total score ≥ 60 and 2-year minimum DoI. Pts randomized to DDAVP nasal spray + RISP (N =22) or plac + RISP (N =22) × 8 wks. DDAVP administered as 10 mcg/day (one spray) × 1 wk → 20 mcg/day (two sprays) × 7 wks. Plac spray similarly to DDAVP spray. RISP continued at same dose (5 or 6 mg/day)	DB RCT. Multicenter clinical study (September 2012–May 2013) Pts treated with stable RISP dose × ≥ 4 wks and partially stabilized (≤20% change on the PANSS-T in two subsequent visits)	Pts treated with DDAVP showed significantly greater improvement in negative symptoms and PANSS general and total psychopathology subscale scores compared to plac.	DDAVP nasal spray shown to be an effective and safe drug for improving negative symptoms in pts with chronic SCZ.
Usall et al., 2014 [93]	Barcelona, Madrid, Valencia, and Victoria, Spain	67 ♂ and 23 ♀ (age range 18–65) with chronic SCZ. Pts randomized to 6 months of adjunctive treatment, divided in 3 groups: (a) citalopram 30 mg/day (N = 23, 18 ♂, 5 ♀; x^-^ age = 42.47), (b) reboxetine 8 mg/day (N = 34; 27 ♂, 7 ♀; x^-^ age = 40.02) (c) plac (N = 33, 22 ♂, 11 ♀; x^-^ age = 44.15)	DB RCT. Multicenter double-blind randomized plac-controlled clinical trial (November 2008–December 2011). Pts had received stable doses of OLA or RISP × ≥60 days prior to inclusion. Psychopathology assessed at BL and wks 12 and 24 through PANSS and SANS	No significant differences on PANSS or SANS between plac and the two treatment groups.	This study does not support add-on citalopram or reboxetine with RISP or OLA for the treatment of negative symptoms in SCZ.
Mirabzadeh et al., 2014 [94]	Tehran, Iran	66 ♂ hospitalized pts (x^-^ age = 37.41 ± 8.46; range 23–57 years) with chronic SCZ and ≥45 on total BPRS scores, randomized to RISP 6 mg/day (N = 37) or to HAL 15 mg/day (N = 29)	DB RCT. 8-week RCT; 2-week AP washout. BPRS at BL and weekly up × 8 wks	RISP and HAL improved negative symptoms of SCZ. HAL was more effective at wk 2 and RISP at wk 8, but difference was not statistically significant.	RISP or HAL for negative symptoms of SCZ are similarly effective. EPS and pt clinical status should be considered; treatment not limited only to treating negative symptoms.
Ghanizadeh et al., 2014 [95]	Shiraz, Iran	43 pts with DSM-IV SCZ randomized to minocycline 200 mg/day + RISP (N = 21) or plac + RISP (N = 22)	Add-on DB, PC, RCT of minocycline 200 mg/day vs. plac, both + RISP as previously taken at Hafez Hospital, Shiraz. Assessment with SANS, PANSS, BDI, and AIMS at BL and wks 4 and 8	After 4 wks, no significant differences. At 8 wks ↓ in scores SANS and PANSS-N scores, which were significant only for the SANS (ΔSANS 12.2 ± 7.9 vs. 6.8 ± 8.6). No pt dropped out.	Minocycline could be a useful adjunctive treatment to RISP for the negative symptoms of SCZ.
Yassini et al., 2014 [96]	Yazd and Rafsanjan, Iran	40 pts (aged 18–60 years) with DSM-IV SCZ and DoI ≥ 2 years, randomized with bupropion (N = 20; 13 ♂, 7 ♀; x^-^ age = 49.58 ± 12.05 years) or plac (N = 20; 12 ♂, 8 ♀; x^-^ age = 49.00 ± 9.45 years)	RCT, DB, PC trial with bupropion (150 mg/day for 3 days, then 300 mg/day) or plac × 8 wks; assessment with SANS at BL and wk 12	No significant differences between groups on SANS scores at wk 12.	No effect of bupropion on negative symptoms.
Buchanan et al., 2015 [97]	Baltimore, MD, U.S.	60 pts with DSM-IV SCZ randomized to rasagiline (N = 31; 24♂, 7♀; x^-^ age= 46.3 ± 12.2 years) or plac (N = 29; 22♂,7♀; x^-^ age= 45.9 ± 11.1 years)	RCT, DB, PC with rasagiline (1 mg/die) or plac × 12 wks; assessment with SANS, RBANS, N-back test at BL and wks 4, 8, and 12	No significant differences in SANS and RBANS scores or N-back test performance between groups at wk 12, but there was a significant treatment × time effect for SANS total scores.	Rasagiline may be of clinical benefit for persistent negative symptoms, but further investigations are needed.
Kantrowitz et al., 2015 [98]	4 U.S. centers (3 New York, NY; 1 New Haven, CT)	44 pts of whom 35 had assessable data, with APS, randomized to D-Ser (N = 15; 8 ♂, 7 ♀; x^-^ age = 20 ± 4.9 years) or plac (N = 20; 15 ♂, 5 ♀; x^-^ age = 19 ± 3.5 years)	RCT, DB, PC with D-Ser (60 mg/kg) or plac × 16 wks at Nathan Kline Institute [NKI], Yale, Zucker Hillside Hospital, and New York State Psychiatry Institute in New York and at PRIME Clinic, Yale University; with a cognitive battery assessment and SOPS at 1, 2, 3, 4, 5, 6, 8, 10, 12, 14, and 16 wks	90% of pts on D-Ser ↓ >20% SOPS-negative vs. 45% of pts on plac (*χ*^2^ = 4.7, *p* = 0.03). D-Ser ↓ SOPS-negative by 35.7% (SD 17.8, vs. plac *p* = 0.03). One conversion to SCZ in the D-Ser group vs. 2 in the plac group (n.s.). D-Ser outperformed plac on the SOPS-negative from wk 12 to wk 16. Final scores did not differ between D-Ser and plac on other SOPS measures (positive, total, disorganization, and general).	D-Ser might help improving negative symptoms of the high-risk psychotic syndrome (prodromal symptoms of SCZ). It does not appear to act on cognition (no significant results obtained on the cognitive battery) or other SOPS dimensions.
Iranpour et al., 2016 [99]	Tehran, Iran	42 pts with DSM-IV SCZ, ≥20 score at PANSS and treated with RISP, randomized to add-on pioglitazone (N = 21; 14 ♂, 7 ♀; x^-^ age = 38 ± 9 years) or plac (N = 21; 15 ♂, 6 ♀; x^-^ age = 37 ± 8 years)	RCT, DB, PC with pioglitazone (30 mg/day) or plac × 8 wks; assessment with PANSS at BL and wks 2, 4, 6, and 8	Pts on pioglitazone showed significantly more improvement in PANSS-N scores and PANSS total scores compared with the plac group.	Negative symptoms might benefit from pioglitazone add-on therapy.
Nikbakhat et al., 2016 [100]	Tehran and Sanandaj, Iran	64 pts (aged 18–50 years) with chronic SCZ, stabilized with RISP ≥ 8 wks and clinically stable for ≥4 wks randomized 1:1 × 8 wks to RISP + duloxetine (21 ♂, 11 ♀, x^-^ age 33.94 ± 5.91 years; DoI x^-^ 8.97 ± 4.55 years) or plac (22 ♂, 10 ♀, x^-^ age 34.22 ± 5.80 years; DoI 8.94 ± 3.85 years)	8-week RCT, DB, PC parallel-group in 2 psychiatric clinics in Iran. Pts randomized to RISP 4–6 mg/day plus duloxetine 60 mg/day or RISP 4–6 mg/day + plac. Rating through PANSS at 2, 4, 6, and 8 wks	Add-on duloxetine ↓ PANSS-N compared to plac from wk 4 on; ↓ PANSS-G and PANSS-T scores compared to plac at all FU timepoints.	Duloxetine added to RISP is tolerable and efficacious in the treatment of primary negative symptoms of SCZ.
Barnes et al., 2016 [101]	15 sites, U.K.	358 pts (aged 18–65 years) diagnosed with a DSM-IV SSD, stabilized with AP for ≥3 months, with a ≥20 PANSS score with ≥3/7 items on the PANSS-N rated ≥3. 85 pts eligible; 23 excluded, 62 pts randomized 1:1 × 48 wks to citalopram (N = 30, 26 ♂, 4 ♀; x^-^ age 43.02 ± 12.3 years; DoI x^-^ 13.96 ± 10.05 years) or plac (N = 32, 22 ♂, 10 ♀; x^-^ age 45.1 ± 12.3; DoI x^-^ 18.10 ± 11.83 years)	DB, PC, parallel arm RCT; 12-month multicenter, DB, individually randomized, PC, parallel-arm RCT. Pts randomized 1:1 to oral citalopram 20 mg/day (→ ↑40 mg/day at 4 wks) or plac (1/2 capsules). FU assessments through PANSS at 12th wk and QLS score at 12th and 48th wks	Citalopram vs. plac → no differences in QLS scores at 12–48th wks and PANSS-N at 48th wk.	Although adjunctive citalopram did not improve overall negative symptoms, there was some evidence of positive effects on avolition/amotivation.
Labad et al., 2016 [102]	Barcelona, Catalunya, Spain	DB, PC, RCT; 78 ♀ pts, in postmenopausal status, diagnosed with SCZ from DSM-IV, stabilized with AP for ≥1 month, with ≥1 symptom score ≥ 4 on the PANSS-N. 8 pts retired, 70 pts started the trial, 65 agreed to DNA collection and completed pharmacogenetics. Randomized 1:1 × 24 wks to raloxifene (36 ♀, x^-^ age 62 ± 9.6) or plac (29♀, x^-^ age 61.1 ± 10.8)	An exploratory pharmacogenetic analysis of a 24-week DB, parallel, PC, RCT. Pts randomized either to raloxifene 60 mg/day or plac. Four SNPs were studied: *ESR1* rs9340799, rs2234693, and rs1801132 and *UGT1A8* rs1042597. PANSS assessments at wks 4, 12, and 24	Pts using raloxifene who were homozygous for the C-allele of the *UGT1A8* gene-related rs1042597 SNP → ↓ PANSS-N at all FU timepoints compared to *ESR1* gene–related SNPs.	The addition of raloxifene to regular AP treatment in postmenopausal women with SCZ and prominent negative symptoms is associated with general improvements in negative symptoms; genetic factors could explain some of the variability in treatment response.
Suresh Kumar et al., 2016 [103]	Kozhikode, Kerala, India	71 inpts and outpts with DSM-IV SCZ, aged 18–64 years; 36 randomized to OLA (25 ♂, 11 ♀, x^-^ age 41.5 ± 9.6 years; DoI x^-^ 157.8 ± 119.6 months); 35 to RISP (23 ♂,12 ♀, x^-^ age 39.8 ± 09.5 years; DoI x^-^ 166.2 ± 119.6 months)	RCT, DB, parallel-group comparison of RISP and OLA in SCZ at the Psychiatry Department of KMCT Medical College × 1 year. RISP (2–8 mg/day); OLA (5–20 mg/day) × 48 wks. Rating through PANSS and CGI at 3rd, 6th, 9th, and 12th months	OLA ↓ PANSS-N compared to RISP from 3rd month on; ↓ CGI severity scale at 3rd, 6rd, and 9rd months of FU.	RISP and OLA generally well tolerated and efficacious in SCZ. OLA showed a significant advantage over RISP in ↓ negative symptoms and overall clinical severity. Apparent benefit within 3 months and persisted >1 year.
Tajik-Esmaeeli et al., 2017 [104]	Tehran, Iran	66 pts with DMS-IV SCZ stabilized on RISP (56 ♂, 10 ♀; x^-^ age = 43.91 ± 8.98 years) randomized to RISP + simvastatin (N = 33) or RISP + plac (N = 33)	DB, PC, RCT. Participants received either RISP (4–6 mg/day) plus simvastatin (40 mg/day) or plac × 8 wks. PANSS rated at BL and 4 and 8 wks	Simvastatin group showed a significantly higher reduction in PANSS-N scores from BL to wk 8 compared to the plac group (x^-^ Δ: 95% CI = −1.42 [OR from −2.32 to −0.52], *p* = 0.003).	Simvastatin as adjunct therapy shows potential in reducing negative symptoms in SCZ pts stabilized on RISP, warranting further research.
Németh et al., 2017 [105]	66 centers in Europe	460 pts with DSM-IV SCZ, randomized to cariprazine (N = 230; 124 ♂, 106 ♀; x^-^ age = 40.2 ± 10.5 years) or RISP (N = 230; 140 ♂, 90 ♀; x^-^ age = 40.7 ± 11.2 years)	DB RCT with cariprazine (4.2 ± 0.6 mg/day) or RISP (3.8 ± 0.4 mg/day) × 26 wks; assessment with PANSS-N at BL and wk 26	Cariprazine led to a bigger improvement in PANSS-N scores compared to RISP.	Cariprazine is a potential treatment strategy for the negative symptoms of SCZ. Study sponsored by cariprazine manufacturers.
Rezaei et al., 2017 [106]	2 sites: Tehran and Sanandaj, Iran	84 outpts 18–50 years with DSM-IV-TR SCZ ≥ 2-year DoI, clinically stable × 4 wks, treated with same RISP dose × 8 wks. Exclusion criteria: ECT in last 3 months, abnormal bleeding, PANSS Depression item ≥ 4; HAM-D score ≥ 14, IQ < 70. Cilostazol+ RISP (42; ♂ 37, ♀ 5, x^-^ age 37.40 ± 7.80 years), RISP +plac (42; ♂ 37, ♀ 5, x^-^ age 36.19 ± 7.18 years)	RCT, DB, PC, parallel-group at Tehran University of Medical Sciences and Qods Psychiatric Teaching Hospital (Kurdistan University of Medical Sciences, Sanandaj; Cilostazol tablet 50 mg bid *+* RISP 2 mg tid vs. plac + RISP 2 mg tid × 8 wks. PANSS at BL and at wks 2, 4, 6, and 8	↓ PANNS-N and PANNS-T scores significantly ↓ with Cilostazol + RISP compared to plac + RISP. ↓ PANNS-P and PANNS-G scores not statistically different between the two groups.	The addition of Cilostazol, a reversible selective PDE3 inhibitor, which is an antiplatelet and antithrombotic agent, can improve negative symptoms in SCZ. Larger and longer and dose-finding studies needed to investigate long-term safety and efficacy.
Buchanan et al., 2017 [107]	Baltimore, Maryland, U.S.	56 inpts or outpts, aged 18–65 years with clinically stable DSM-IV-TR SCZ or SCAD (same APs for 2 months and same dose for 1 month), SANS ≥ 20, BPRS-P score ≤ 16, BPRS Anxiety/Depression factor score ≤ 14. Randomized to OT + plac (16 pts, ♂ 14, ♀ 2, x^-^ age 47.4 ± 11.2 years) or galantamine+ plac (20 pts, ♂ 14, ♀ 6, x^-^ age 45.8 ± 12.4 years) or plac (20 pts; ♂ 17, ♀ 3, x^-^ age 42.2 ± 11.7 years)	RCT, PC, DB, double-dummy. Pts assessed for eligibility for 4 wks, → randomized to i.n. OT (24 IU bid) + plac, i.n. plac + galantamine (12 mg bid) or i.n. plac + oral plac × 6 wks at at Maryland Psychiatric Research Center and Spring Grove Hospital Center. Rating through SANS, BPRS-T, BPRS-P, CDRS, and CGI-S at BL and wks 2, 4, and 6; adverse events as reported	OT, galantamine, and plac did not differ for ↓ of SANS, BPRS-P, BPRS-T, CDRS, CGI-S scores; treatment × time (wk) interaction not significant for galantamine vs. OT, galantamine vs. plac, and OT vs. plac for all scales and cognition; more enuresis with OT.	Galantamine, OT, and plac did not differ on their effects on negative symptoms of SCZ.
Ghajar et al., 2018 [108]	Tehran and Sanandaj, Iran	66 outpts 18–60 years with SCZ DSM-5 with DoI ≥ 2 years, clinically stable × 4 wks, treated with same RISP dose × 8 wks; exclusion criteria: ECT in last 2 months, Depression item of the PANSS ≥ 4, HAM-D ≥ 14, IQ < 70, serious medical, neurological, or other psychiatric disorders. Randomized to Citicoline + RISP (33 pts, ♂ 31, ♀ 2, x^-^ age 45.36 ± 11.63 years) or plac + RISP (33 pts, ♂ 28, ♀ 5, x^-^ age 48.85 ± 10.65 years)	RCT, DB, PC, parallel-group; pts randomized to oral Citicoline tablet 2.500 mg/day in bid or RISP 6 mg/day × 8 wks in two sites, Roozbeh-Tehran and Qods-Sanandaj Hospitals. Assessment at BL and at wks 2, 4, 6, and 8 with PANSS	Statistically significant ↓ of PANNS-N, PANSS-T, and PANSS-G scores in the Citicoline + RISP group compared to plac + RISP but no differences in PANNS-P.	Citicoline as add-on therapy to RISP showed good tolerability and significant beneficial effects on the negative symptoms of pts with chronic stable SCZ.
Liu et al., 2018 [109]	Kunming, China	55 pts (aged 18–40 years) with SCZ, stabilized with stable dose of RISP for at least 4 weeks; disease duration ≤ 5 years. A total of 79 SCZ pts were screened, and 63 were enrolled. 55 pts completed week16 assessments. 27 minocycline +RISP (11 ♂, 16 ♀, x^-^ age 26.7 ± 5.5 years; DoI x^-^ 19.0 ± 12.3 months), 28 plac + RISP (12 ♂, 16 ♀, x^-^ age 28.9 ± 7.0 years; DoI x^-^ 30.2 ± 14.5 months)	16-week, randomized DB trial in two hospitals in China. Pts were randomly assigned to receive minocycline (200 mg/day) or plac. Rating through SANS and PANSS at baseline and week 16	The minocycline group had significant ↓ in SANS total sore and PANSS-N score at week 16 compared to the plac group.	Minocycline added on RISP does benefit negative symptoms of SCZ.
Zhang et al., 2018 [110]	Guangzhou, China	75 pts (aged 18–45 years) with SCZ. AP-free × ≥ 2 wks before study entry; 25 minocycline high dose (13 ♂, 12 ♀, x^-^ age 33.24 ± 6.48 years; DoI x^-^ 5.98 ± 1.78 years), 25 minocycline low dose (12 ♂, 13 ♀, x^-^ age 33.04 ± 7.78 years; DoI x^-^ 6.28 ± 1.82 years) and 25 plac (13 ♂, 12 ♀, x^-^ age 33.68 ± 11.32 years; DoI x^-^ 6.27± 1.71 years). 57 pts completed the 3-month treatment: 18 on minocycline high dose, 20 minocycline low dose and 19 plac. x^-^ RISP daily dose also did not differ (x^-^ = 4.38 ± 0.52 mg/day for minocycline high dose, x^-^ = 4.40 ± 0.51 mg/day for minocycline low dose and x^-^ = 4.39 ± 0.53 mg/day for plac. 30 HCs (♂/♀ = 14/16), with x^-^ age = 32.07 ± 4.65 years were recruited	3-month RCT, DB trial in a hospital in China. Subjects were assigned low-dose (100 mg per day) or high-dose minocycline (200 mg per day starting at 100 mg during week 1 and 100 mg twice daily from week 2) or plac combined with RISP 3 mg to 6 mg/day. The dose of RISP was gradually increased to 3 mg/day in the first week and then was adjusted from 3 mg to 6 mg/day. Lorazepam for insomnia and trihexyphenidyl hydrochloride for EPS. Rating through SANS and PANSS at baseline and at months 1, 2, and 3 or at the end of treatment if a patient dropped out	Subjects receiving high-dose minocycline had greater improvements on the SANS total scores and PANSS negative subscale scores.	SCZ pts showed a significant improvement in negative symptoms with the addition of minocycline to RISP.
Ding et al., 2018 [111]	Shandong, China	91 pts (aged 18–60 years) and 29 HCs (15 ♂, 14 ♀, x^-^ age 41.8 ± 8.5 years). 62 pts with TRS and persistent negative symptoms, hospitalization × >6 months and stable symptoms. DoI ≥5 years. Pts were being treated with combination APs (OLA + RISP or RISP + ARI) or single antipsychotics (OLA or RISP). 62 pts randomized to escitalopram (15 ♂, 13 ♀, x^-^ age 42.4 ± 12.7 years; DoI x^-^ 18.821 ± 9.611 years) or plac (12 ♂, 14 ♀, x^-^ age 49.7 ± 9.4 years; DoI x^-^ 22.77 ± 10.041 years) × 8 wks	8-week RCT, DB in a mental health center in China. Rating through PANSS	After 8 wks, ↓ in PANSS-N and affective subscore were greater in the escitalopram group compared to plac.	Escitalopram augmentation improves negative symptoms.
Ghajar et al., 2018 [112]	Tehran, Iran	63 pts (aged 18–60 years) with chronic SCZ, stabilized with RISP (up to 6 mg/day during the course of the trial). Pts with sleep problems received 1 mg lorazepam every night for the first week of the trial. DoI ≥ 2 years. 31 pts assigned to RISP + L-Carn, 30 completed trial (28 ♂, 2 ♀, x^-^ age 43.67 ± 8.78 years; DoI x^-^ 21.67 ± 11.35; RISP dose 4.38 ± 0.46) and 32 to RISP + plac, 30 completed trial (26 ♂, 4 ♀, x^-^ age 45.97 ± 9.30 years; DoI x^-^ 21.03 ± 11.0; RISP dose 4.40 ± 0.40)	8-week RCT, DB at Roozbeh Hospital, Tehran, Iran. Pts randomized to to L-Carn (2 gr/day in two divided doses) or plac. Rating through PANSS at T0 and at 2nd, 4th, 6th, and 8th wks	L-Carn improved PANSS-N and PANSS-T scores.	L-Carn add-on therapy can reduce the negative symptoms of pts with SCZ.
Deakin et al., 2018 [113]	12 centers in the U.K.	207 pts with chronic SCZ on stable AP treatment for ≥4 wks prior to study initiation and predominantly negative symptoms recruited from 12 U.K. National Health Service trusts. Pts randomized to minocycline (n = 104, 77 ♂, 27 ♀, x^-^ age 25.5 ± 5.2 years) or plac (n = 103, 73 ♂, 30 ♀, x^-^ age 25.7 ± 5.1 years). DoI ≥ 5 years	RCT, DB trial. Pts randomly assigned to receive minocycline (200 mg/day × 2 wks → 300 mg/day for the remainder of the 12-month study period) or to receive plac. Rating through PANSS and CDSS across FUs at months 2, 6, 9, and 12	Compared with plac, the addition of minocycline had no effect on ratings of negative symptoms.	Minocycline does not benefit negative SCZ symptoms.
Earley et al., 2019 [114]	65 centers across the U.S., India, Russia, Ukraine, and Malaysia	317 pts with DSM-IV-TR SCZ since ≥1 year, both sexes, aged 18–60 years. Inclusion criteria included CGI-S score ≥ 4, PANSS 80–120, and ≥4 on at least 2 of PANSS-P delusions, hallucinatory behavior, suspiciousness/persecution, or conceptual disorganization. Pts assigned to plac (n = 79, 46 ♂, 33 ♀) cariprazine 1.5–3 mg/d (n = 94, 58 ♂, 36 ♀), cariprazine 4.5–6 mg/day (n = 66, 41 ♂, 25 ♀), RISP 4.0 mg/day (n = 34, 24 ♂, 10 ♀), or ARI 10.0 mg/day (n = 44, 30 ♂, 14 ♀)	2 randomized, DB, PC, and active-controlled cariprazine studies in pts with acute SCZ were pooled. Changes from BL to wk 6 in PANSS-N were assessed in the following treatment groups: plac (n = 79), cariprazine 1.5–3 (n = 94) and 4.5–6 mg/day (n = 66), RISP 4 mg/day (n = 34), or ARI 10 mg/day (n = 44)	Significant differences vs. plac for cariprazine (1.5–3 mg/day, *p* = 0.0179; 4.5–6 mg/day, *p* = 0.0002) and RISP (*p* = 0.0149) but not ARI (*p* = 0.3265) and vs. ARI for cariprazine 4.5–6 mg/day (*p* = 0.0197). After adjusting for positive symptom changes, differences vs. plac remained significant for cariprazine (1.5–3 mg/day, *p* = 0.0322; 4.5–6 mg/day, *p* = 0.0038) but not for RISP (*p* = 0.2204). PANSS-N response (≥20% ↓ from BL rates) significantly ↑ with cariprazine (1.5–3 mg/day = 54.3%, *p* = 0.0194; 4.5–6 mg/day = 69.7%, *p* = 0.0001) compared to plac (35.4%).	In pts with acute SCZ and moderate/severe negative symptoms, cariprazine was associated with significantly greater improvement in negative symptoms compared with plac and ARI, warranting further exploration of the efficacy of cariprazine on negative symptoms.
Samaei et al., 2020 [115]	Tehran, Iran, 2 sites	52 pts aged 18–60 DSM-5 SCZ, DoI ≥ 2 years. Participants with PANSS ≥ 15 and clinically stable and on a stable dose of RISP for the last 8 wks prior to the beginning of the study. Clinical stability was considered as ≤20% change in the total score of PANSS on 2 consecutive ratings. Pts assigned to resveratrol (n = 26, 16 ♂ 10 ♀, x^-^ age 34.73 ± 7.03) or plac (n = 26, 15 ♂ 11 ♀, x^-^ age 33.08 ± 5.48)	DB, PC, RCT of add-on 200 mg/day resveratrol or matched plac to stable RISP dose × 8 wks. Pts at Roozbeh and Razi Hospitals, Tehran, assessed with PANSS, ESRS, and HAM-D over the trial period. Primary outcome = Δ PANSS-P and Δ PANSS-N from BL to wk 8	52 pts completed the trial (26 in each arm). BL characteristics of both groups were statistically similar (*p* > 0.05). Despite statistical similarity of positive symptoms across time (Greenhouse–Geisser corrected: *F* = 1.76, df = 1.88, *p* = 0.18), the resveratrol group showed greater improvement in PANSS-N, PANSS-G, and PANSS-T (Greenhouse–Geisser corrected: *F* = 12.25, df = 2.04, *p* < 0.001; *F* = 5.42, df = 1.56, *p* = 0.011; F = 7.64, df = 1.48, *p* = 0.003). HAM-D scores and Δ scores, ESRS score, and frequency of other complications did not differ significantly between resveratrol and plac.	Adding resveratrol to RISP can ↑ efficacy on negative symptoms while ↓ EPS.
Hosseininasab et al., 2021 [116]	Sari, Iran	56 ♂ inpts aged 18–60 years with chronic SCZ and predominantly negative symptoms, clinically stable on currently stable AP received × ≥2 years, 29 assigned to nanocurcumin (x^-^ age = 49.3 ± 11.7) and 29 to plac (x^-^ age = 47.8 ± 12.1, n.s., *p* = 0.6). Smokers were 22 on nanocurcumin (75.9%) vs. 21 on plac (72.4%; n.s., *p* = 0.5). Pts allowed to receive lorazepam or biperiden during the study	DB, PC trial, 1:1 randomized to nanocurcumin soft gel capsule (160 mg/d) and plac. Pts in the intervention group received nanocurcumin soft gel capsule, with an initial dose of 80 mg/day (1 capsule) × 2 days, increased to 160 mg/day (2 capsules in 2 divided doses) on day 3 and continued until the end of the study along with their AP regimen × 16 wks. Pts in the plac group received the same identical soft gel capsule along with their AP regimen × 16 wks. Assessment through PANSS, CGI-S and -I, CDRS, SAS, and BARS. Primary outcome Δ between nanocurcumin and plac in PANSS-T and subscale scores	Both nanocurcumin and plac improved on all measures from BL to wk 16 but on CDRS, SAS, and BARS. The nanocurcumin group improved significantly more than the plac group on the PANSS-N from BL at wks 8 (*p* = 0.03) and 16 (*p* = 0.04) and on the PANSS-G and PANSS-T scores at wks 8, 12, and 16 (*p* = 0.009-<0.001). The nanocurcumin group improved more than plac on both CGI-S and CGI-I (*p* < 0.001).	Nanocurcumin as an add-on may improve negative symptoms and other symptoms of SCZ without posing safety problems.
Banazadeh et al., 2022 [117]	Kerman, Iran	48 hospitalized pts (36 ♂ [75%], 12 ♀ [25%]) with chronic SCZ, aged 18–65 years (x^-^ age = 45.02 ± 10.30 years) with symptoms and medications unchanged during the last two months assigned to black myrobalan (N = 23, x^-^ age = 44.65 ± 9.19 years) or to plac (N = 25, x^-^ age = 45.36 ± 11.41 years; difference n.s., *p* = 0813). Other participant characteristics at BL (marital status, education, number of hospitalizations, length of current hospital stay, DoI, and concomitant medications) did not differ between the groups	DB, PC, RCT at Golestan Salamat sanatorium; pts randomized 1:1 to black myrobalan (six capsules/day 500 mg powder with 10% w/w sweet almond oil divided between morning and evening) or to plac (six capsules/day starch divided between morning and evening) × 4 wks. Assessment at BL and wk 4 through PANSS and SCIP	SCIP scores ↑ (*p* = 0.004) and PANSS-N ↓ (*p* = 0.017) significantly more at wk 4 in the black myrobalan than in the plac group. Anxiety and the PANSS excitatory component improved more in the black myrobalan than in the plac group (*p* = 0.003).	Black myrobalan could improve cognitive impairments, negative symptoms, and excitement/activity symptoms in pts with chronic SCZ.
Bugarski-Kirola et al., 2022 [118]	83 sites (18 in North America, 65 in Europe)	403 stable outpts with SCZ aged 18–55 years with predominant negative symptoms randomized to pimavanserin (n = 201; 131 [65%] ♂; 187 [93%] white; x^-^ age = 37.7 ± 9.4 years) or plac (n = 202; 137 [68%] ♂, 186 [92%] white; x^-^ age = 36.7 ± 9.2 years), of whom 400 were included in the efficacy analysis (199 in the treatment, 201 in the plac group)	DB, PC, RCT ADVANCE study, phase 2:1 randomized × 26 wks to add-on pimavanserin 20 mg/day adjusted to 10 or 34 mg/day or plac daily, added to ongoing AP. Assessment through Marder’s Negative PANSS factor and NSA-16	The change in total NSA-16 score from BL to wk 26 was significantly improved with pimavanserin (least squares mean −10.4 [SE 0.67]) vs. plac (least squares mean −8.5 [0.67]; *p* = 0.043; effect size: 0.211, modest).	Stable pts with predominant negative symptoms of SCZ showed ↓ negative symptoms after treatment with pimavanserin.
Li et al., 2022 [119]	Tianjin, China	65 randomly selected 18–65-year-old inpts with SCZ on a stable SGA dose of a single drug × ≥ 1 month 70 pts screened, 65 eligible randomized to berberine (n = 35) and plac (n = 30). Analyzed 32 berberine pts (12 ♂ [37.5%]; 20 ♀; x^-^ age = 43.41 ± 10.77) and 27 plac (6 ♂ [22.2%], 21 ♀; x^-^ age = 40.52 ± 11.48; Student’s *t* = 0.996, *p* = 0.324, n.s. for age, χ^2^ = 0.262, n.s.). BL demographic/clinical characteristics (DoI, EQdose, BMI, HOMA-IR, educational level, marital status, and AP dose) did not differ between groups	DB, PC, RCT of berberine hydrochloride (900 mg/day) or plac as add-on to AP × 8 wks. Assessment at screening, BL and wk 4 and wk 8 with PANSS. Primary outcome: differences in PANSS-N; secondary: differences in PANSS-P, PANSS-G, and PANSS-T	From the baseline to the 8th week, berberine treatment significantly ↓ PANSS-N (*F* = 18.981; *p* < 0.001). Treatment × time effect significantly better for berberine compared to plac from BL to wk 8 for PANSS-N (F = 6.722; *p* = 0.004). At wk 4, berberine vs. plac *F* = 3.213; *p* = 0.083, n.s.; at wk 8, berberine vs. plac *F* = 5.83; *p* = 0.022 for PANSS-N. At wk 8, berberine vs. plac *F* = 7.219; *p* = 0.002 for PANSS-T, no significant effects for PANSS-P and PANSS-G.	Pts taking berberine 900 mg/day had a significant improvement in negative symptoms of SCZ than pts taking plac.Berberine may improve negative symptoms through anti-inflammatory effects. In the berberine group, the change of CRP concentration positively correlated with changes on PANSS-N within 8 wks (*r* = 0.56; *p* = 0.002).
Neill et al., 2022 [120]	Australian cities of Melbourne, Brisbane, Adelaide, and Sydney	85 pts with DSM-5 SCZ or SCAD, aged 18–65 years, on a stable dose of CLZ for ≥6 months who, despite adequate dosing (serum level of >350 µg/L), continued to experience residual symptoms, defined as either a score of >4 on two or more PANSS-N or a total PANSS score ≥ 60. 42 randomized to NAC (28 ♂, 14 ♀; x^-^ age 39.83 ± 9.19 years) and 43 to plac (33 ♂, 10 ♀; x^-^ age 39.65 ± 9.41 years)	DB, multicenter, PC, RCT of pts on CLZ with enduring psychotic symptoms to investigate the efficacy of adjunctive NAC (2 g/day) for negative symptoms, cognition and QoL. Efficacy assessed at 8, 24, and 52 weeks. The primary outcome was the PANSS-N	NAC did not significantly improve negative symptoms (*p* = 0.62) at any timepoint over a 1-year period. No differences in reported side effects between the groups (*p* = 0.26).	NAC did not significantly improve SCZ negative symptoms in treatment-resistant pts taking CLZ.
Salehi et al., 2022 [121]	Tehran, Iran	60 pts with DSM-5 SCZ, aged 18–60–years and DoI ≥ 2 years, ≥15 on PANSS-N at BL. 60 met criteria and randomized to RISP + PEA (n = 30) or RISP + plac (n = 30). Included in the final analysis were 25 PEA + RISP (23 ♂, 2 ♀; x^-^ age 33.76 ± 6.93 years) and 25 to plac + RISP (21 ♂, 4 ♀; x^-^ age 36.80 ± 9.60 years) who showed sufficient treatment adherence	DB, PC, RCT of pts with SCZ randomized 1:1 to PEA 600 mg × 2/day +RISP and plac + RISP × 8 wks. Efficacy and safety assessed at BL and wk 4 and 8 with PANSS, HAM-D, and ESRS. Stable RISP doses. Primary outcome Δ PANSS-N during the trial period	Significant effect of time × treatment interaction on negative symptoms (*p* = 0.012) in the PEA add-on group. ESRS and adverse events not different between PEA and plac (*p* > 0.05).	Adjunctive therapy with PEA and RISP alleviates SCZ-related primary negative symptoms safely.
Tharoor et al., 2023 [122]	Chennai, Tamil Nadu, India	100 pts aged 18–45–years with ICD-10 SCZ and predominant negative symptoms (SANS ≥ 60 at BL) randomized 1:1 to add-on L-Carn (N = 50; 35 ♂, 15 ♀; x^-^ age 32.1 ± 7.4 years) and add-on plac (N = 50; 31 ♂, 19 ♀; x^-^ age 31 ± 5.8 years)	DB, PC, parallel, 6-month RCT. Inpts and outpts from SCZ Research Foundation, Chennai, India. L-Carn 400 mg/day × 3 months → 800 mg/day × another 3 months; SANS, SAPS, and CGI-S at BL and 1 month, 3 months, and 6 months; NIMHANS at 6 months	No significant change in both groups in SANS and CGI scores; no differential effect on SANS subdomains. L-Carn better than plac on attention.	L-Carn add-on provides no clinical benefit compared to plac in pts with stable SCZ and predominant symptoms.
Zierhut et al., 2024 [123]	Berlin, Germany	41 individuals with SSD aged 18–65 years; 22 randomized to OT (16 ♂, 6 ♀, x^-^ age = 40.53 ± 10.30); 19 to plac (15 ♂, 4 ♀, x^-^ age 43.11 ± 11.65)	DB, PC, RCT at Charité. Participants on MBGT randomly allocated to add-on OT or plac. OT was administered as nasal spray (24 I.U. Syntocinon^®^) or matching plac at “T0” and “T2” (unspecified timing but 1 wk apart). Pre- and post-intervention negative symptoms assessed with the SNS	OT compared to plac ↓ avolition and diminished emotional range among negative symptoms and negative affect, while it ↑ positive affect. OT did not affect empathy, while plac ↑ it.	OT combined with MGBT showed some positive results on negative symptoms. Tables do not elucidate results.
Mazhar et al., 2024 [124]	Tehran, Iran	56 pts with SCZ randomized to saffron + RISP (N = 28) or plac + RISP (N = 28); groups similar for BL characteristics	DB, PC, RCT at Roozbeh Psychiatric Hospital, Tehran, pts randomized to saffron 15 mg q 12 h or plac in add-on to RISP × 8 wks. PANSS and HAM-D at BL and 4 and 8 wks	Time × Treatment interaction effects on PANSS-N (=0.137), PANSS-G (=0.193), and PANSS-T (=0.113). Significantly >PANSS ↓ in saffron group at wk 4 (Cohen’s *d* = 0.922 for PANSS-N, 0.898 for PANSS-G, and 0.759 for PANSS-T) and at wk 8 (Cohen’s *d* = 0.850 for PANSS-N, 1.047 for PANSS-G, and 0.705 for PANSS-T). Saffron better than plac at endpoint (*p* = 0.003). HAM-D scores did not differ between saffron and plac.	Saffron better than plac in ↓ negative symptoms of SCZ when added on RISP. Difference not due to ↓ in depression. Safron shown well tolerated and safe.
Shamabadi et al., 2024 [125]	Tehran, Iran (2 sites)	69 outpts age 18–60 years with SCZ since ≥2 years, PANSS-N > 14 and HAM-D < 14 randomized to pentoxyfilline 800 mg/day (N = 35, 20 ♂ [57.1%], 15 ♀ [42.9%]; x^-^ age = 36.63 ± 6.94) or plac (N = 34, 18 ♂ [52.9%], 16 ♀ [47.1%]; x^-^ age = 36.21 ± 7.09) added on 4–6 mg/day RISP and clinically stable × > 2 wks	DB, PC, RCT at Roozbeh and Razi Psychiatric Hospitals, outpt facilities, Tehran; oupts randomized 1:1 to pentoxifylline 400 mg q 12 h + RISP or plac + RISP × 8 wks. Assessment at BL and 4 and 8 wks through PANSS and HAM-D and ESRS at BL and 8 wks. Response was ≥25% ↓ in PANSS-N	Significant time effect for both pentoxifylline + RISP and plac + RISP on ↓ of PANSS-N and time×treatment interaction, with pentoxifylline better than plac on PANSS-N at wks 4 and 8. Responders were 13 pts on pentoxifylline + RISP and 7 on plac + RISP at wk 4 (n.s.) and 29 pts on pentoxyfilline + RISP and 19 on plac + RISP at wk 8 (82.9% vs. 55.9%; *p* = 0.019). Significant time effect for both pentoxifylline + RISP and plac + RISP on ↓ of PANSS-P and PANSS-G but no time × treatment interaction (pentoxifylline and plac did not differ). No differences between pentoxifylline and plac on HAM-D and ESRS.	Pentoxifylline could be a useful addition to ongoing drug treatment to pts with SCZ stabilized on RISP.
Huang et al., 2025 [126]	Hunan, China	77 pts with SCZ lasting ≤10 years, PANSS-N ≥ 20, and one or two APs to keep constant throughout, randomized 2:1 to add-on sulforaphane (N = 53; 28 ♂, 25 ♀; x^-^ age = 23.75 ± 6.21) or plac (N = 24; 12 ♂, 12 ♀; x^-^ age = 23.68 ± 6.32)	DB, PC, RCT at 2nd Xiangya Hospital of Central South University, Hunan, randomized to 2 tablets/day sulforaphane (24 mg/day) or plac × 24 wks. Outcomes measured at BL and 12 and 24 wks: PANSS-N (primary), PANSS negative factor, CGI, and TESS	Sulforaphane was followed by stronger ↓ in PANSS-N (*p* < 0.001) and PANSS negative factor scores (*p* < 0.002) compared to plac at wk 24. Results could not be explained by ↓ in depressive or cognitive symptoms. The two groups did not differ on TESS scores or EPS.	High-dose sulforaphane added on ongoing AP drug treatment has a large effect size on negative symptoms of SCZ (Cohen’s *d* = 0.86).

Abbreviations: AD(s), antidepressant drug(s); AEs, adverse events; AIMS, Abnormal Involuntary Movement Scale; AP(s), antipsychotic drug(s); APS, attenuated psychotic symptoms (prodromal SCZ); ARI, aripiprazole; ATRS, Abrams and Taylor rating scale; BACS, Brief Assessment of Cognition in Schizophrenia; BARS, Barnes Akathisia Rating Scale; bid, bis in die, twice daily; BDI, Beck Depression Inventory; BL, baseline; BPRS, Brief Psychiatric Rating Scale; CDRS, Calgary Depression Rating Scale; CGI, Clinical Global Impressions, -S, severity, -I, improvement; CLZ, clozapine; CPT, Continuous Performance Test; CPZ, chlorpromazine; CRP, C-reactive protein; DAmph, dextro-amphetamine; DB, double-blind; DCS, D-cycloserine; DD, double-dummy; DDAVP, desmopressin, deamino D-arginine vasopressin; DoI, duration of illness; D-Ser, Dextro-serine; DSM-III-R, Diagnostic and Statistical Manual of Mental Disorders, 3rd edition-Revised; DSM-IV, Diagnostic and Statistical Manual of Mental Disorders, 4th edition; DSM-5, Diagnostic and Statistical Manual of Mental Disorders, 5th edition; ECT, ElectroConvulsive Therapy; EPS, extra-pyramidal symptoms; ER, extended-release; *ESR1*, Estrogen Receptor 1 gene; ESRS, Extrapyramidal Symptoms Rating Scale; FEP, first-episode psychosis; FGA(s), first-generation antipsychotic(s), neuroleptic(s), typical antipsychotic(s); FU, follow-up; GAF, global assessment of functioning; HAL, haloperidol; HAM-D, Hamilton Depression Rating Scale; HC(s), healthy control(s); ICD-10, International Classification of Disorder-Tenth Edition; i.m., intramuscular; i.n., intranasal; inpts, inpatients; IQ, intelligence quotient; ITT, intention-to-treat analysis, population; I.U., international units; iv, intravenous; LAI(s), long-acting injectable antipsychotics; L-Carn, L-carnosine; LN-RS, Luria–Nebraska Rating Scale; LSM, least square means; MADRS, Montgomery–Åsberg Depression Rating Scale; MBGT, mindfulness-based group therapy; MCCB, MATRICS Consensus Cognitive Battery; MDBS, Movement Disorder Burden Score; MDD, Major Depressive Disorder; NAC, *N*-acetylcysteine; NIMHANS, National Institute for Mental Health and Neurosciences cognitive battery; NOSIE, Nurses’ Observation Scale for Inpatient Evaluation; n.s., not significant; NSA-16, 16-item Negative Symptom Assessment scale; OLA, olanzapine; OS, olanzapine–sertraline combination; OT, oxytocin; outpts, outpatients; PALI, Paliperidone; PANSS, Positive And Negative Syndrome Scale; PANSS-G, PANSS general symptom subscale; PANSS-N, PANSS negative subscale; PANSS-P, PANSS positive subscale; PANSS-T, PANSS total score; PC, placebo controlled; PDE3, phosphodiesterase III; PDS, Psychotic Depression Scale; PEA, Palmitoylethanolamide; plac, placebo; PREG, pregnenolone; PSP, Personal and Social Performance Scale; pt(s), patient(s); QoL, quality-of-life; QUET, quetiapine; RCT, randomized control trial; RDC, Research Diagnostic Criteria; RISP, risperidone; SAFTEE, Systematic Assessment for Treatment-Emergent Side Effects; SANS, Scale for Assessment of Negative Symptoms; SAPS, Scale for Assessment of Positive Symptoms; SAS, Simpson–Angus Scale for Extrapyramidal Symptoms; SCAD, schizoaffective disorder; SCIP, Screen for Cognitive Impairments in Psychiatry; SCZ, schizophrenia; SD, standard deviation; SE, standard error; SFS, Social Functioning Scale; SGA(s), second-generation antipsychotic(s), atypical antipsychotic(s); SNP(s), single nucleotide polymorphism(s); SNS, Self-Evaluation of Negative Symptoms; SOPS, Scale Of Prodromal Symptoms/Scale Of Psychotic-Risk Syndrome; SSD, schizophrenia spectrum disorders; SSTMST, Sternberg Short Term Memory Scanning Test; SUD, substance use disorder; TEAEs, treatment-emergent adverse events; TESS, treatment-emergent side-effect scale; tid, tris in diem, thrice daily; TRS, treatment-resistant schizophrenia; *UGT1A8*, Uridine 5′-diphospho-glucuronosyltransferase 1A8 gene; U.S., United States of America; VA, Veteran Administration; wk(s), week(s); WSEEPS, Webster scale for the evaluation of extrapyramidal symptoms; W-SOHO, Worldwide Schizophrenia Outpatient Health Outcomes study; x^-^, mean; ZLOW, ziprasidone 60–120 mg/day in a single dose; ZSTD, ziprasidone 80–160 mg/day divided in two daily doses; Δ, delta, change; ≈, about equal, not different; ×, multiple, for, per; ±, standard deviation; ♀, female; ♂, male; ↓, decrease, reduced, diminished, low; ↑, increased, augmented, high; →, induced, resulted in, followed.

## 4. Discussion

In this review, we identified several studies testing the effects of marketed drugs on the negative symptoms of SCZ. The study designs were, at the beginning, comparisons between drugs in monotherapy, involving one antipsychotic vs. another antipsychotic or vs. an antidepressant or other psychoactive drugs or placebo, but with time, add-on designs came to prevail, with various drugs and phytopharmacology/antioxidant agents and glutamatergic receptor modulators vs. placebo or another drug added on a standard antipsychotic that was taken for some time, ensuring patient stabilization. There is no net tendency of these trials as related to their anti-negative symptom effects; most of them reported positive effects, and few trials failed, and these tended to be conducted in the Western world, while the effects of most studies conducted in Iran and China were promising.

A case in point is represented by the antibiotic minocycline. This drug received trials for negative symptoms due to its antioxidant/neuroprotective effects [137] and the ability to attenuate NMDA blockade-induced hyperlocomotion and pre-pulse inhibition [138] in animal paradigms of neurodegeneration and psychosis. These animal models are not specific for the negative symptoms of SCZ; nevertheless, this drug has been the one most studied as an adjunct to stable antipsychotic treatment for the treatment of negative symptoms, represented here in seven eligible studies. Minocycline was studied as an add-on to stable antipsychotic doses starting from 2012 onward. The first was a mixed Pakistani–Brazilian study [80] showing positive results on negative symptoms in both countries. It was followed by three studies in 2014 and three in 2018. Two of them were in Iran, one positive [89] and the other moderately positive [95]; three were conducted in China [90,109,110], and were all positive; the one conducted in the U.K. was negative [113]. Since then, there have been no other trials on minocycline. It is known that differential effects in trials are affected by placebo effects, which in turn depend on cultural/ethnic factors [139]. It is supported that patient responses differ among different countries based on common patient beliefs in various countries; for example, patients in the U.S. are likely to respond better to more innovative treatments, while in China, Austria, and the U.K., where the public is more conservative and traditionalist, patients tend to respond better to standard, older drugs [140]. This may reflect the fact that observational learning affects and induces placebo effects [141]. One study identified differences in placebo responses in similar trials conducted in Russia, India, and the U.S., with U.S. patients responding better to placebo than patients in India and Russia [142]. Considering that the increasing placebo response in clinical trials applies also to SCZ [143] and that most recent trials involve traditional and not-so-innovative drugs, it is possible that in the Western world, the drug–placebo gap is closing, and most drug trials are declared as having failed due to this fact. An example of this is that lumateperone, which showed good efficacy in SCZ [144], had a modest effect size [145] largely attributable to a large placebo effect in that study [146]. It is essential to combine efforts and involve investigators from all relevant stakeholders to identify the underlying issues and address the inconsistencies in the data.

Most trials involved the addition of antipsychotics other those that the patients were already taking or comparing one vs. another (N = 24). After decades of conventional antipsychotics that did not resolve the negative-symptom-unresponsiveness problem, the advent of SGAs has generated new hopes and prompted trials initially involving SGAs vs. FGAs (risperidone, clozapine, remoxipride, olanzapine, and ziprasidone, each vs. haloperidol, in the hope to find positive results for the new drugs) and subsequently SGAs vs. each other (olanzapine vs. risperidone, quetiapine vs. risperidone, olanzapine vs. quetiapine, asenapine vs. olanzapine, and cariprazine vs. risperidone or vs. aripiprazole); these latter studies were conducted with the economic support of pharmaceutical companies and tended to report results favorable to the drug of the sponsor [147,148]. All drugs obtained improvement of negative symptoms but of small magnitude and similar overall for all comparators (Table 1). The one antipsychotic that received more trials among antipsychotics (N = 6) was the substituted benzo amide amisulpride, which is a highly specific inhibitor of D_2/3_ dopamine receptors [149], with some antagonistic activity towards 5-HT_7_ and 5-HT_1B_ serotonin receptors [150] and a quite strong binding affinity for the γ-hydroxybutyrate receptor [151]. The rationale for its use in negative SCZ resides in the fact that the negative symptoms are believed to depend on lower dopaminergic activity in the prefrontal cortex (PFC), while positive symptoms depend on higher dopaminergic activity in the limbic system (hippocampus, amygdala, ventral striatum/nucleus accumbens, and septum) [152]. Antagonists have preferential activity for autoreceptors; hence, amisulpride at low doses would block D_2_ autoreceptors, thus increasing synaptic dopamine release [153]. This in turn would reduce the negative symptoms of SCZ. The same principle is exploited by another class of SGAs, D_2/3_ partial dopamine agonists like aripiprazole, brexpiprazole, and cariprazine; these drugs would activate dopaminergic transmission in the PFC due to the partial agonism of these post-synaptic receptors and affect glutamatergic transmission [154,155,156], thus mimicking the effect of preferential autoreceptor activation. One reasoning was that low doses of classical antipsychotics that are used in maintenance would lessen the grip of higher doses needed during acute exacerbations to address positive symptoms on negative symptoms, which are believed to worsen by protracted high-dose antipsychotic treatment [49]; however, despite a trend for improvement with lower dose-specific anti-D_2_ treatment, patients were as likely to receive a low as a high dose after one year [46]. Amisulpride has received six trials in all, and almost all were moderately positive (vs. low-dose haloperidol [46]) or positive (vs. placebo [42,44,49]), with one vs. ziprasidone showing similar response rates for the two antipsychotics [59]; only in one [63] was it not differentiated from low-dose olanzapine or placebo. Taken together, these data suggest that the policy of low-dose antipsychotic maintenance for chronic SCZ first proposed by Baldessarini et al. [157] is still valid. What remains intriguing is that despite the positive literature, the low-dose amisulpride strategy is little adopted by clinicians, underscoring a potential gap between research findings and clinical practice. The dopamine D_2/3_ partial agonist approach has been suggested specifically for negative symptoms of SCZ [158], but as of now, only cariprazine has received two trials vs. risperidone at one single dose [105], with the other two doses of cariprazine vs. risperidone and vs. aripiprazole [114]; they were both positive for cariprazine, but the studies were supported by its manufacturers. Brexpiprazole has still to receive its maiden trial.

The very first study to compare a neuroleptic with a non-dopaminergic drug was conducted in the north of England [31] and involved a comparison between the piperidine phenothiazine, thioridazine, a D_1_/D_2_ blocker with significant anti-5-HT_2A_ and α_1_ adrenolytic activity, and a β_1_/β_2_-blocker [159,160] with some 5-HT_1A/1B_ serotonin receptor binding affinity [161,162], namely propranolol. The latter showed significantly greater activity against the negative symptoms than the former, which showed some activity only in a narrow time interval. This study could not respond to the serotonin vs. noradrenaline question of the pathophysiology of negative symptoms that was later dealt with by selective serotonin transporter vs. noradrenaline transporter inhibitor comparisons, which were similarly inconclusive [36,93]. It is noteworthy that this drug and other β-blockers have not received trials on the negative symptoms of SCZ since the mid-1980’s. Another hypertensive that received trials on the negative symptoms of SCZ was verapamil, a phenylalkylamine calcium channel blocker, which was tested in two early studies [32,33] and was found positive in the first and negative in the second and since then abandoned. The voltage-gated sodium channel blocker carbamazepine, which is used as an anti-seizure and mood stabilizing agent, was not superior to placebo and had no effects against negative symptoms [38]. Another channel blocker that was trialed for negative symptoms is the benzothiazole tetrodotoxin-sensitive sodium channel blocker riluzole, which is used in amyotrophic lateral sclerosis [163]; it showed positive results in one Iranian study [88], but this was not replicated, although this drug was shown to decrease anterior cingulate cortex (ACC) glutamate-plus-glutamine levels (which were correlated with negative symptoms) and strengthen ACC-anterior PFC functional connectivity in patients with treatment-resistant SCZ [164]. The mechanism of action of riluzole is still not clarified, so we cannot speculate regarding its possible interference with the negative symptoms of SCZ.

Antidepressant drugs and monoamine oxidase B inhibitors (MAOB-Is) (which are used as antiparkinsonian agents) received 20 trials (4 MAOB-Is). The reasoning behind the use of antidepressants is that negative symptoms resemble depression, but depression in SCZ differs from negative symptoms to the point that a specific assessment scale was crafted by a Canadian early-psychosis group [15]. However, just two studies on antidepressants have employed this scale in assessing depression. All others used the standard Hamilton Depression and Montgomery–Åsberg Depression Rating Scales, which are not able to tell depression from negative symptoms. Many studies were negative (N = 8). The first trial involving a comparison of antidepressants was that of Silver and Nassar [36]; it compared fluvoxamine with maprotiline, using the drugs as neurochemical probes. The study posited that if the two drugs exhibited equivalent efficacy, it would indicate the involvement of both serotonergic and noradrenergic mechanisms in the negative symptoms of SCZ. Should fluvoxamine demonstrate superior efficacy, serotonergic mechanisms would be considered predominant. Conversely, if maprotiline proved more effective, noradrenergic pathways would be implicated. The study found fluvoxamine to be able to lower negative symptoms and maprotiline ineffective in this regard, concluding that serotonergic mechanisms are important in the pathogenesis of negative symptoms. The same rationale was adopted in a citalopram vs. reboxetine vs. placebo study [93], which, however, found no effect of all antidepressants used vs. placebo. Paroxetine vs. placebo was positive in one study and trazodone vs. placebo (weakly positive) in another, while reboxetine (two studies) and bupropion obtained negative results (i.e., no evidence of efficacy). Escitalopram and citalopram were tested in six studies overall, of which just two (one for the racemic and one for the S-(+)-enantiomer) were positive; all others were failed trials. In contrast, mirtazapine, which has been used in four studies (one of which used the CDRS to rate depression) showed consistently positive results [52,54,75,86].

Antiparkinsonian MAOB-Is underwent four trials: three for selegiline [50,57,68], with all positive, and one for rasagiline [97], which was tendentially positive but inconclusive (study performed in the U.S.).

Another numerous group is that of antioxidants/anti-inflammatory drugs (N = 11 studies); this chemically heterogenous group includes statins, thiazolidinediones, alkaloids, and many plant extracts; most studies were recent, many conducted in Iran and China, and yielded generally positive results. A recent meta-analysis confirmed positive effects on negative symptoms for sarcosine and *N*-acetylcysteine, although the bulk of the studies they examined were determined to have poor quality [165]. Among other drug categories, those studies using neuropeptide analogues (oxytocin and vasopressin) had very atypical designs and produced negative or doubtful results. Seven studies used amino acids with generally negative or inconsistent results. The one that received more trials was D-cycloserine, an NMDA glutamate receptor partial agonist acting near the glycine site [166]. This drug received three trials in the U.S.; it did not supersede the placebo in one study [55] and was similarly ineffective to another amino acid, glycine, in another study [67], while it outperformed the placebo in a last study [69]. After 2008, it received no further trials in SCZ for the treatment of negative symptoms. Quite curiously, a later Iranian study tested the NMDA non-competitive antagonist memantine [167] in patients with SCZ and negative symptoms and reported encouraging results [83]. These authors did not provide a sensible rationale for using this drug despite the recognizing controversial results obtained up to their experiment. More than eleven years after, their results have not been replicated.

Stimulant drugs have received three trials, one each for dextro-amphetamine [34], modafinil [66], and armodafinil (the R-isomer of modafinil) [77], and produced doubtful to disappointing results. Steroids (pregnenolone [71,91] and raloxifene [102]) and phosphodiesterase inhibitors, whether 3 [106] or 5 [74] or non-selective [126], obtained promising results (but not Lu AF11167, an experimental phosphodiesterase 10A inhibitor-dopamine D_1_ and D_2_ receptor-modulator, which did not differentiate from placebo in one study [168]), as did serotonin 5-HT_2A_ [70,118] and 5-HT_3_ antagonists [81,82] and the cholinergic drug citicoline [108] (interestingly, xanomeline-trospium, a muscarinic M_4_ agonist [169], is the first non-dopaminergic drug after many years to obtain FDA approval for SCZ [170], and clozapine, one of the most effective antipsychotics, is also a muscarinic M_4_ agonist [171,172]) but not the cholinesterase inhibitor galantamine [107]. While citicoline is believed to act through muscarinic receptors [173], it may also act through nicotinic α7 receptors [174] and antioxidant mechanisms [175]. The studies involved for each drug and for each drug class are too few to allow us to draw sound conclusions about the participation of glutamate, serotonin, and acetylcholine receptors in the pathophysiology of the negative symptoms of SCZ. Finally, the μ-opioid receptor blocker naltrexone was used in one study [43] based on the observation that intracerebral administration of opioid peptides induced catatonia-like symptoms in animals [176]. Results were slightly positive, but the instruments used to assess psychopathology were not appropriate for evaluating negative symptoms. A recent and well-performed study casts doubt on the possible direct involvement of μ-opioid receptors in the pathophysiology of the negative symptoms of SCZ [177].

The first-performed studies started with small patient numbers. The first 1985 study included 45 patients [31], but all too often, during 1987–1999, samples of less than 20, often between 10 and 20, were used. With the new millennium, samples increased, as multicenter studies were conducted involving many countries. Overall, ten studies were carried out with samples numbering between 10 and 20; the majority of studies was conducted on samples numbering between 22 and 99 (N = 60), and 25 studies were carried out on 100 patients or more, ranging from 100 [122] to 617 [51]. The increase in the samples through the years was not paralleled by a better quality of the studies. It is often said that including various populations from diverse sites increases the generalizability of results; this would be true if investigators were obliged to declare their inter-site differences, but this rarely occurs in recent times, and sponsors and journals are happy with declaration of conflicts of interests. Intra-study variability is becoming a major problem in contemporary science, limiting generalizability and misleading conclusions, but is not considered a concern. Often, studies focus on efficacy but skip effectiveness, as few real-world studies are conducted on this issue. A search of PubMed on 11 February 2025 using a “negative symptoms”[ti] AND schizophrenia[ti] AND real-world[ti] strategy produced eight papers, of which only one was on pharmacotherapy and was a review plus a summary of a real-world study [178]. The effectiveness of a study is its efficacy plus its clinical acceptability and applicability; the latter include safety and tolerability and must take into account side effects. These are usually reported but seldom focused upon in efficacy studies.

It is well known that FGAs are often accompanied by motor disturbances, including parkinsonism, tardive dyskinesia, akathisia, and dystonias, but some of them also develop while being treated with SGAs; for example, akathisia quite frequently occurs with aripiprazole, while parkinsonism may develop while on risperidone or paliperidone. Side effects were carefully monitored in many of the studies included in this systematic review [39,44,47,50,55,59,61,62,63,64,72,75,76,80,81,82,83,115,116,120], but the greatest focus was on movement disorders. Given that the comparators were antipsychotics, or the studies were add-on trials, it is reasonable that these disorders were primarily focused upon. However, all the classes used here to overcome the limitations of the already-used antipsychotics that were proven to be ineffective have side effects of their own that could limit their effectiveness, even in clinical trials of efficacy.

The adverse events associated with the use of the various drug classes reviewed here were not specifically sought in the studies included herein. Antioxidants, for example, while generally reducing adverse events induced by oxidative stress and other treatments, either pharmacological or biological [179,180], may trigger adverse endocrine effects [181] or increased sperm decondensation [182]. Among channel blockers, verapamil [183], carbamazepine [184], and riluzole [185] have different and non-overlapping side effects. The phosphodiesterase inhibitors sildenafil and cilostazol display different side-effect profiles, with sildenafil more likely to be associated with sexual side effects [186] and cilostazol with cardiovascular ones [187]. Antidepressant drugs share both side effects and diversity [188,189,190] also on the basis of factors intrinsic to individual patients [191]. Some of these side effects may develop after long-term treatment; these long-term side effects may be absent in the duration of a single-drug trial; thus, we may obtain false pictures with time-limited trials.

Hypotheses on the pathogenesis of negative symptoms were generated in the course of years, paralleling current zeitgeists and the results of the clinical studies of each period. In the early 1990’s, the focus was on dopaminergic mechanisms [192], matching the proposals of Timothy Crow [9] and Angus Mackay [8], while in the first decade of the 21st century, following the observations and theories of Herbert Meltzer based on the clinical effects of clozapine [193] and the development of increasingly sophisticated neuroimaging methods, hypotheses involving serotonin–dopamine interactions [194] and right temporo-prefrontal reductions of glucose metabolism [195] were put forth. In the second decade of the millennium, the involvement of other neurotransmitters (GABA-glutamate imbalance, endocannabinoids, and nicotinic cholinergic) and neuropeptides (oxytocin) was proposed, based on results from animal paradigms [196,197]. Another observation regarded the negative correlation between triglycerides and the negative syndrome and the positive correlation between high-density lipoprotein cholesterol and the negative syndrome [198], but this also led to no formulation regarding the possible pathophysiological mechanisms of the negative syndrome. The fragmentation of negative symptoms into primary persistent and deficit syndrome-related (which are also persistent) [199] and secondary negative symptoms [200] led to no new hypotheses but to newly recognized psychopathological entities. A new hypothesis was advanced that intertwined negative symptoms with neuroinflammation, histone deacetylase (HDAC), and oxidative stress, pointing to the possible efficacy of the glutathione enhancer sulforaphane in negative symptoms [201,202]. Its property to block HDAC, although fascinating due to the obvious hint to epigenetics and the involvement of this predominantly nuclear enzyme in the pathophysiology of SCZ [203,204,205,206], is not supported by clinical evidence with valproate, a known HDAC inhibitor [207], which was shown to do little to negative symptoms of SCZ [208,209,210]. Although sulforaphane received trials in SCZ, it was not found to affect negative symptoms in an American [211] and a Chinese double-blind study [212], while it was found to be promising in an open Chinese study [213] (interestingly, the reduction in PANSS-negative scores paralleled the increase in the antioxidant superoxide dismutase) and in a recent double-blind study included in this review [126] but, nevertheless, paved the way to the experimentation of antioxidant molecules, which now dominate the negative-symptom scene.

During the third decade of the 21st century, investigators converged on the idea that negative symptoms can be divided in their symptom subdomains, and each should be explored individually. Asociality, avolition, and anhedonia were grouped to form an apathy/reduced motivation cluster (experiential), while blunted affect and alogia form the reduced expression cluster [214,215,216]. The apathy cluster is supposed to be associated with reduced function of the orbitofrontal, dorsolateral PFC, dorsal and ventral striatum/n. accumbens, and ACC, while the reduced expression cluster would be linked to reduced ventrolateral PFC and abnormal transmission in the amygdala [217]; this subdivision of negative symptoms would have treatment implications according to recent evidence [214,215]. A recent review proposed specific mechanisms for each negative symptom domain, i.e., hyperactivation in the amygdala, hypoactivation in the PFC, and deficit in mirror neuron function in blunted affect; impaired basal ganglia/accumbens in alogia; impaired reward mechanisms (nucleus accumbens/basal ganglia) in anhedonia (dopamine imbalance); cortico-striatal hypoactivation in avolition motivation (dopamine imbalance); and defective neuropeptide function (the affiliation neurohormone oxytocin) in the hypothalamus/posterior pituitary in asociality [218]. However, the therapeutic implications remain largely unchanged whether the symptom clusters are considered as unified entities or analyzed individually. Similar indications have been suggested, yet these have not been substantiated in clinical practice (for example, oxytocin [107,123]).

One interesting mechanism that has been proposed is the double 5-HT_2A_/σ_2_ antagonism; while 5-HT_2A_ antagonism has been advocated to explain the differential effects of SGAs vs. FGAs and the lack of the classical neuroleptic side effects of the former [219] (the evidence is by far unconvincing [220], and the neurochemical mechanisms speak against it [221]), σ_2_ antagonism has not been so much focused upon, although haloperidol is known to bind this site [221,222]. Sigma sites were formerly believed to be opioid receptors [223], but now it is realized that they are chaperones tied to the reticular endoplasmic membrane [224]. The σ_2_ chaperone has been shown to be involved in autophagy [225] and neurodegenerative diseases, while evidence for its involvement in SCZ is still to be ruled out [226]. The only known antipsychotic that has some sigma-binding activity is quetiapine, and its binding to these sites, namely σ_1_ or σ_2_, is believed to mediate its “antidepressant” effects in mice [227], though its binding affinity is rather weak [228]. Recently, the drug industry developed a σ_2_/α_1A_ adrenoceptor/5-HT_2A_ antagonist with brain-derived neurotrophic factor-stimulating activity [229], first called MIN-101 and then roluperidone (2-[[1-[2-(4-Fluorophenyl)-2-oxoethyl]piperidin-4-yl]methyl]-3H-isoindol-1-one), which has received trials since 2015. The drug was reported in a 2017 paper to benefit the negative symptoms of SCZ of 244 patients in a study conducted in six European countries (36 sites) by a group of Israeli investigators [230]. Subsequently, positive cognitive effects were reported along with negative symptom improvement in a subgroup of these same patients by American investigators [231]. Another paper reporting positive effects on negative symptoms [232] was one focusing on adding to the validation of the Brief Negative Symptom Scale and dealt with a subsample of the 2015 sample [232,233]. The two studies reported on two doses of roluperidone, 32 mg/day and 64 mg/day for 12 weeks, which both fared better than the placebo. Subsequently, there were reports and re-analyses of the same sample focusing on outcomes like personal and social adjustment [234] and reduced emotional experience and reduced emotional expression, and both were improved [235]. Patients from the same sample underwent network analysis that identified avolition as “the most central domain for the successful treatment of negative symptoms” [236], a result that was confirmed on an enlarged 60-site, 496-patient sample from eight European countries, Israel, and the U.S. [237]. In a 61-site, 513-patient study recruiting during December 2017–February 2020 and conducted in eight European countries, Israel, and the U.S. with the same design, only the 64 mg/day dosage reduced significantly negative symptoms compared to the placebo and displayed more responders, confirming the superior effect size compared to roluperidone 32 mg/day [238]. The long-term observation of both the 2015 and 2017–2020 samples confirmed the efficacy and safety of the drug in an open-label extension study, which recorded a low re-exacerbation rate [239]. In all these studies, roluperidone was administered as monotherapy. These encouraging results led the pharmaceutical company to submit an application to the FDA for a new drug [240] but received a negative response, requiring further studies and results with co-administration of roluperidone with other antipsychotics [241,242].

*Limitations*. Study heterogeneity, lack of sufficiently justified rationales of most studies, lack of consistence in the times used to treat the patients (extending from 2 weeks to 12 months), sponsors’ conditioning of results, and lack of sufficient numbers of studies on a specific drug are a few of the limitations affecting our review. Other might include the use of unsuitable rating scales to assess what investigators were wishing to measure and also the exclusion of non-marketed drugs, thus lacking possible innovative proposals that could offer a clue as to the pathophysiology of the negative symptoms of SCZ. Combining the search for markers of negative SCZ, both chemical and neuroimaging, may offer a viable way to solve the problem.

## 5. Conclusions

Considering all the available evidence, numerous promising approaches have been developed for treating the negative symptoms of SCZ—symptoms traditionally regarded as the most resistant to resolution. Yet, the pressing question remains: why has no approach proven effective in real-world applications, leaving this area an ongoing unmet need? Despite frequent claims of progress, the overall picture highlights the need for more robust and consistent findings.

We propose that the negative symptoms of SCZ, being more subtle and often preceding the onset of the disorder, have a longer window to disrupt the body’s homeostasis during development. This allows them to establish deeper and more resilient roots, making them harder to eradicate. While there are intriguing theories, such as the neurodevelopmental hypothesis, we still lack a clear understanding of why and how these symptoms emerge. However, this gap in knowledge alone does not explain their resistance to treatment, as we similarly lack full understanding of the mechanisms behind positive symptoms, yet we manage to control those more effectively. For now, we have no definitive answer. What we can advocate for is continued rigorous evidence collection for every drug trialed. Beyond the common refrain of “more studies are needed”, we must acknowledge the difficulty of this task and remain committed to exploring new avenues, guided by the belief that serendipity may ultimately lead us to breakthroughs.

## Data Availability

All used data are contained in the text or in the Supplementary Materials.

## Supplementary Materials

The following supporting information can be downloaded at https://www.mdpi.com/article/10.3390/biomedicines13030540/s1. Table S1: Studies emerging from our search and eligibility, with main reason for exclusion for each study; Table S2: Drugs used in eligible studies of drug treatment of the negative symptoms of schizophrenia; Table S3. PRISMA Checklist.

## Abbreviations

The following abbreviations are used in this manuscript:

BPRS	Brief Psychiatric Rating Scale
CDRS	Calgary Depression Rating Scale
CGI (-I, -S)	Clinical Global Impressions (-Improvement, -Severity)
DDAVP	Arginine vasopressin, desmopressin
FGA	First-generation antipsychotic
GABA	γ-Aminobutyric acid
HAM-D	Hamilton Depression Rating Scale
MADRS	Montgomery–Åsberg Depression Rating Scale
MAOB-I	Mono amine oxidase B inhibitor
NaSSA	Noradrenaline-serotonin specific antagonist
NSA-16	16-item Negative Symptom Assessment scale
PANSS	Positive And Negative Syndrome Scale
PRISMA	Preferred Reporting Items for Systematic Reviews and Meta-Analyses
RoB 2	Cochrane Risk-of-Bias tool, 2nd version
SANS	Scale for the Assessment of Negative Symptoms
SAPS	Scale for the Assessment of Positive Symptoms
SCZ	Schizophrenia
SGA	Second-generation antipsychotic
SSRI	Selective serotonin re-uptake inhibitor
U.K.	United Kingdom of Great Britain and Northern Ireland
U.S.	United States of America
