# Supplementary material for: Pharmacological Interventions for Negative Symptoms in Schizophrenia: A Systematic Review of Randomised Control Trials
Source: Biomedicines. 2025 Feb 21;13(3):540. doi: 10.3390/biomedicines13030540 (PMC11940281; doi:10.3390/biomedicines13030540)
Supplement: Supplementary file 1 [file biomedicines-13-00540-s001.zip › biomedicines-3471940-supplementary.pdf]

Supplementary Table S1. Studies emerging from our search and eligibility, with main reason for exclusion for each study. Search strategy: schizophrenia[ti] AND "negative symptom*"[ti] PubMed 11.2.2025 → 1495 results

1	King DJ. Dopamine agonists for negative symptoms in schizophrenia. Br J Clin Pharmacol. 1978;6(6):541-2. doi: 10.1111/j.1365-2125.1978.tb00882.x.	Open
2	Andreasen NC, Olsen SA, Dennert JW, Smith MR. Ventricular enlargement in schizophrenia: relationship to positive and negative symptoms. Am J Psychiatry. 1982;139(3):297-302. doi: 10.1176/ajp.139.3.297.	No drug
3	Andreasen NC. Negative symptoms in schizophrenia. Definition and reliability. Arch Gen Psychiatry. 1982;39(7):784-8. doi: 10.1001/archpsyc.1982.04290070020005.	No drug
4	Ferrier IN, Roberts GW, Crow TJ, Johnstone EC, Owens DG, Lee YC, O'Shaughnessy D, Adrian TE, Polak JM, Bloom SR. Reduced cholecystokinin-like and somatostatin-like immunoreactivity in limbic lobe is associated with negative symptoms in schizophrenia. Life Sci. 1983;33(5):475-82. doi: 10.1016/0024-3205(83)90797-x.	*Post-mortem*
5	Allen HA. Do positive symptom and negative symptom subtypes of schizophrenia show qualitative differences in language production? Psychol Med. 1983;13(4):787-97. doi: 10.1017/s0033291700051497.	No drug
6	Pearlson GD, Garbacz DJ, Breakey WR, Ahn HS, DePaulo JR. Lateral ventricular enlargement associated with persistent unemployment and negative symptoms in both schizophrenia and bipolar disorder. Psychiatry Res. 1984;12(1):1-9. doi: 10.1016/0165-1781(84)90133-1.	No drug
7	Chaturvedi SK, Gopinath PS, Mathai PJ, Michael A. Negative symptoms and negative schizophrenia. Indian J Psychiatry. 1984;26(3):200-5. PMID: 21965985; PMCID: PMC3011238.	No drug
8	Rosen WG, Mohs RC, Johns CA, Small NS, Kendler KS, Horvath TB, Davis KL. Positive and negative symptoms in schizophrenia. Psychiatry Res. 1984;13(4):277-84. doi: 10.1016/0165-1781(84)90075-1.	No drug
9	Pogue-Geile MF, Harrow M. Negative symptoms in schizophrenia: their longitudinal course and prognostic importance. Schizophr Bull. 1985;11(3):427-39. doi: 10.1093/schbul/11.3.427.	No drug
10	Zubin J. Negative symptoms: are they indigenous to schizophrenia? Schizophr Bull. 1985;11(3):461-70. doi: 10.1093/schbul/11.3.461.	Review
11	Rosen J, Silk KR, Rice HE, Smith CB. Platelet alpha-2-adrenergic dysfunction in negative symptom schizophrenia: a preliminary study. Biol Psychiatry. 1985;20(5):539-45. doi: 10.1016/0006-3223(85)90025-3.	No drug
12	Chaturvedi SK, Rao GP, Mathai PJ, Sarmukaddam S, Gopinath PS. Negative symptoms in schizophrenia and depression. Indian J Psychiatry. 1985;27(3):237-41.	No drug
13	Green M, Walker E. Neuropsychological performance and positive and negative symptoms in schizophrenia. J Abnorm Psychol. 1985;94(4):460-9. doi: 10.1037//0021-843x.94.4.460.	No drug
14	Eccleston D, Fairbairn AF, Hassanyeh F, McClelland HA, Stephens DA. The effect of propranolol and thioridazine on positive and negative symptoms of schizophrenia. Br J Psychiatry. 1985;147:623-30. doi: 10.1192/bjp.147.6.623.	**Included**
15	Wolkowitz OM, Pickar D, Doran AR, Breier A, Tarell J, Paul SM. Combination alprazolam-neuroleptic treatment of the positive and negative symptoms of schizophrenia. Am J Psychiatry. 1986;143(1):85-7. doi: 10.1176/ajp.143.1.85.	Case
16	Walker E, Harvey P. Positive and negative symptoms in schizophrenia: attentional performance correlates. Psychopathology. 1986;19(6):294-302. doi: 10.1159/000284452.	No drug
17	Green M, Walker E. Attentional performance in positive- and negative-symptom schizophrenia. J Nerv Ment Dis. 1986;174(4):208-13. doi: 10.1097/00005053-198604000-00003.	No drug
18	Waddington JL, Youssef HA. An unusual cluster of tardive dyskinesia in schizophrenia: association with cognitive dysfunction and negative symptoms. Am J Psychiatry. 1986;143(9):1162-5. doi: 10.1176/ajp.143.9.1162.	No drug
19	Kota SK, Kulhara P, Joseph S, Nagpal RS. Inter-rater reliability of the scale for assessment of negative symptoms in schizophrenia. Indian J Psychiatry. 1986;28(4):349-50.	No drug
20	Waddington JL, Youssef HA. Late onset involuntary movements in chronic schizophrenia: relationship of 'tardive' dyskinesia to intellectual impairment and negative symptoms. Br J Psychiatry. 1986;149:616-20. doi: 10.1192/bjp.149.5.616.	No drug
21	Meltzer HY, Sommers AA, Luchins DJ. The effect of neuroleptics and other psychotropic drugs on negative symptoms in schizophrenia. J Clin Psychopharmacol. 1986;6(6):329-38.	Review
22	Bucci L. The negative symptoms of schizophrenia and the monoamine oxidase inhibitors. Psychopharmacology (Berl). 1987;91(1):104-8. doi: 10.1007/BF00690936.	No placebo comparator
23	Whiteford HA, Riney SJ, Csernansky JG. Distinguishing depressive and negative symptoms in chronic schizophrenia. Psychopathology. 1987;20(5-6):234-6. doi: 10.1159/000284506.	No drug
24	Fisch RZ. Trihexyphenidyl abuse: therapeutic implications for negative symptoms of schizophrenia? Acta Psychiatr Scand. 1987;75(1):91-4. doi: 10.1111/j.1600-0447.1987.tb02757.x.	Case
25	Fleischhacker WW, Barnas C, Stuppäck C, Unterweger B, Hinterhuber H. Zotepine in the treatment of negative symptoms in chronic schizophrenia. Pharmacopsychiatry. 1987;20(1 Spec No):58-60. doi: 10.1055/s-2007-1017131.	Open
26	Petit M, Zann M, Lesieur P, Colonna L. The effect of sulpiride on negative symptoms of schizophrenia. Br J Psychiatry. 1987;150:270-1. doi: 10.1192/bjp.150.2.270a.	No placebo comparator
27	Singh MM, Kay SR, Opler LA. Anticholinergic-neuroleptic antagonism in terms of positive and negative symptoms of schizophrenia: implications for psychobiological subtyping. Psychol Med. 1987;17(1):39-48. doi: 10.1017/s0033291700012964.	Unfocused
28	Kanofsky JD, Kay SR, Lindenmayer JP, Opler LA. Catatonic schizophrenia and negative symptoms. Can J Psychiatry. 1987;32(2):162.	No drug
29	Kulhara P, Chadda R. A study of negative symptoms in schizophrenia and depression. Compr Psychiatry. 1987;28(3):229-35. doi: 10.1016/0010-440x(87)90029-0.	No drug
30	Frith CD. The positive and negative symptoms of schizophrenia reflect impairments in the perception and initiation of action. Psychol Med. 1987;17(3):631-48. doi: 10.1017/s0033291700025873.	No drug
31	Thomas P, King K, Fraser WI. Positive and negative symptoms of schizophrenia and linguistic performance. Acta Psychiatr Scand. 1987;76(2):144-51. doi: 10.1111/j.1600-0447.1987.tb02877.x.	No drug
32	Waddington JL, Youssef HA, Dolphin C, Kinsella A. Cognitive dysfunction, negative symptoms, and tardive dyskinesia in schizophrenia. Their association in relation to topography of involuntary movements and criterion of their abnormality. Arch Gen Psychiatry. 1987 Oct;44(10):907-12. doi: 10.1001/archpsyc.1987.01800220077011.	No drug
33	Cohen JD, Van Putten T, Marder S, Berger PA, Stahl SM. The efficacy of piquindone, a new atypical neuroleptic, in the treatment of the positive and negative symptoms of schizophrenia. J Clin Psychopharmacol. 1987;7(5):324-9.	Non-marketed
34	Barnas C, Stuppaeck C, Unterweger B, Hinterhuber H, Fleischhacker WW. Treatment of negative symptoms in schizophrenia with zotepine. J Clin Psychopharmacol. 1987;7(5):370-1. doi: 10.1097/00004714-198710000-00032.	Open
35	Price WA, Pascarzi GA. Use of verapamil to treat negative symptoms in schizophrenia. J Clin Psychopharmacol. 1987;7(5):357. doi: 10.1097/00004714-198710000-00020.	**Included**
36	Uhr SB, Jackson K, Berger PA, Csernansky JG. Effects of verapamil administration on negative symptoms of chronic schizophrenia. Psychiatry Res. 1988;23(3):351-2. doi: 10.1016/0165-1781(88)90027-3.	**Included**
37	van Kammen DP, Boronow JJ. Dextro-amphetamine diminishes negative symptoms in schizophrenia. Int Clin Psychopharmacol. 1988;3(2):111-21. doi: 10.1097/00004850-198804000-00002.	**Included**
38	Keilp JG, Sweeney JA, Jacobsen P, Solomon C, St Louis L, Deck M, Frances A, Mann JJ. Cognitive impairment in schizophrenia: specific relations to ventricular size and negative symptomatology. Biol Psychiatry. 1988;24(1):47-55. doi: 10.1016/0006-3223(88)90120-5.	No drugs

39	Walker E, Lewine RJ. The positive/negative symptom distinction in schizophrenia. Validity and etiological relevance. Schizophr Res. 1988;1(5):315-28. doi: 10.1016/0920-9964(88)90045-x.	No drugs
40	Berry T. L-dopa for the negative symptoms of schizophrenia. Am J Psychiatry. 1988;145(9):1180-1. doi: 10.1176/ajp.145.9.1180b.	Case
41	Brambilla F, Bondiolotti GP, Maggioni M, Sciascia A, Grillo W, Sanna F, Latina A, Picotti GB. Vasopressin (DDAVP) therapy in chronic schizophrenia: effects on negative symptoms and memory. Neuropsychobiology. 1989;20(3):113-9. doi: 10.1159/000118483.	**Included**
42	Yamagami S, Soejima K. Effect of maprotiline combined with conventional neuroleptics against negative symptoms of chronic schizophrenia. Drugs Exp Clin Res. 1989;15(4):171-6.	Open
43	Meltzer HY, Zureick J. Negative symptoms in schizophrenia: a target for new drug development. Psychopharmacol Ser. 1989;7:68-77. doi: 10.1007/978-3-642-74430-3_8.	Opinion
44	Alphs LD, Summerfelt A, Lann H, Muller RJ. The negative symptom assessment: a new instrument to assess negative symptoms of schizophrenia. Psychopharmacol Bull. 1989;25(2):159-63.	No drug
45	Glick ID, Jacobs M, Lieberman J, Simpson G, Schooler NR. Prediction of short term outcome in schizophrenia: depressive symptoms, negative symptoms, and extrapyramidal signs. Treatment Strategies in Schizophrenia Collaborative Study Group. Psychopharmacol Bull. 1989;25(3):344-7.	Unfocused
46	de Leon J, Wilson WH, Simpson GM. Measurement of negative symptoms in schizophrenia. Psychiatr Dev. 1989;7(3):211-34.	No drug
47	Bouricius JK. Negative symptoms and emotions in schizophrenia. Schizophr Bull. 1989;15(2):201-8. doi: 10.1093/schbul/15.2.201.	No drug
48	Lenzenweger MF, Dworkin RH, Wethington E. Models of positive and negative symptoms in schizophrenia: an empirical evaluation of latent structures. J Abnorm Psychol. 1989 Feb;98(1):62-70. doi: 10.1037//0021-843x.98.1.62. PMID: 2708643.	No drug
49	Den Boer JA, Westenberg HG, Verhoeven WM. Symptômes négatifs et schizophrénie. Une revue succincte des données cliniques et pharmacothérapeutiques [Negative symptoms and schizophrenia. A concise review of clinical and pharmacotherapeutic data]. Encéphale. 1989;15(2):273-82. French.	Review
50	Tandon R, Shipley JE, Eiser AS, Greden JF. Association between abnormal REM sleep and negative symptoms in schizophrenia. Psychiatry Res. 1989;27(3):359-61. doi: 10.1016/0165-1781(89)90151-0.	No drug
51	Tandon R, Silk KR, Greden JF, Goodson J, Hariharan M, Meador-Woodruff JH, Kronfol Z. Positive and negative symptoms in schizophrenia and the Dexamethasone Suppression Test. Biol Psychiatry. 1989;25(6):788-92. doi: 10.1016/0006-3223(89)90251-5.	No drug
52	Montague LR, Tantam D, Newby D, Thomas P, Ring N. The incidence of negative symptoms in early schizophrenia, mania and other psychoses. Acta Psychiatr Scand. 1989;79(6):613-8. doi: 10.1111/j.1600-0447.1989.tb10310.x.	No drug
53	McCarley RW, Faux SF, Shenton M, LeMay M, Cane M, Ballinger R, Duffy FH. CT abnormalities in schizophrenia. A preliminary study of their correlations with P300/P200 electrophysiological features and positive/negative symptoms. Arch Gen Psychiatry. 1989;46(8):698-708. doi: 10.1001/archpsyc.1989.01810080028004.	No drug
54	Chadda RK, Jain BK. Depression and negative symptoms in schizophrenia. Am J Psychiatry. 1989;146(8):1083. doi: 10.1176/ajp.146.8.1083.	No drug
55	Ellenbroek BA, Willemen AP, Cools AR. Are antagonists of dopamine D1 receptors drugs that attenuate both positive and negative symptoms of schizophrenia? A pilot study in Java monkeys. Neuropsychopharmacology. 1989;2(3):191-9. doi: 10.1016/0893-133x(89)90022-5.	Animal
56	Berner P, Küfferle B, Friedmann A, Grünberger J, Saletu B. Traitement des symptômes déficitaires de la schizophrénie par les neuroleptiques [Treatment of negative symptoms in schizophrenia with neuroleptics]. Encéphale. 1989;15(5):457-63. French.	Review
57	Jackson HJ, Minas IH, Burgess PM, Joshua SD, Charisiou J, Campbell IM. Negative symptoms and social skills performance in schizophrenia. Schizophr Res. 1989;2(6):457-63. doi: 10.1016/0920-9964(89)90014-5.	No drug
58	Pfefferbaum A, Ford JM, White PM, Roth WT. P3 in schizophrenia is affected by stimulus modality, response requirements, medication status, and negative symptoms. Arch Gen Psychiatry. 1989;46(11):1035-44. doi: 10.1001/archpsyc.1989.01810110077011.	No drug
59	Barnes TR, Liddle PF, Curson DA, Patel M. Negative symptoms, tardive dyskinesia and depression in chronic schizophrenia. Br J Psychiatry Suppl. 1989;(7):99-103.	No drug
60	Negative symptoms in schizophrenia. Symposium. London, 17-18 September 1987. Proceedings. Br J Psychiatry. 1989;155(Suppl 7):1-135.	Duplicate= 61,62,63
61	Lindenmayer JP, Kay SR. Depression, affect and negative symptoms in schizophrenia. Br J Psychiatry. 1989;155(Suppl 7):108-14.	No drug
62	Pogue-Geile MF. The prognostic significance of negative symptoms in schizophrenia. Br J Psychiatry. 1989;155(Suppl 7):123-7.	No drug
63	Sass H. The historical evolution of the concept of negative symptoms in schizophrenia. Br J Psychiatry. 1989;155(Suppl 7):26-31; discussion 37-40.	No drug
64	Whiteford HA, Peabody CA. The differential diagnosis of negative symptoms in chronic schizophrenia. Aust N Z J Psychiatry. 1989;23(4):491-6. doi: 10.3109/00048678909062616.	No drug
65	McGauley GA, Aldridge CR, Fahy TA, Eastment C. The dexamethasone suppression test and negative symptoms of schizophrenia. Acta Psychiatr Scand. 1989;80(6):548-53. doi: 10.1111/j.1600-0447.1989.tb03024.x.	No drug
66	Pickar D, Litman RE, Konicki PE, Wolkowitz OM, Breier A. Neurochemical and neural mechanisms of positive and negative symptoms in schizophrenia. Mod Probl Pharmacopsychiatry. 1990;24:124-51. doi: 10.1159/000418015.	Review
67	Kay SR. Positive-negative symptom assessment in schizophrenia: psychometric issues and scale comparison. Psychiatr Q. 1990;61(3):163-78. doi: 10.1007/BF01064966.	No drug
68	Marks RC, Luchins DJ. Relationship between brain imaging findings in schizophrenia and psychopathology. A review of the literature relating to positive and negative symptoms. Mod Probl Pharmacopsychiatry. 1990;24:89-123.	Review
69	Schizophrenia: positive and negative symptoms and syndromes. Mod Probl Pharmacopsychiatry. 1990;24:1-200.	Duplicate= 68,70
70	McGlashan TH, Heinssen RK, Fenton WS. Psychosocial treatment of negative symptoms in schizophrenia. Mod Probl Pharmacopsychiatry. 1990;24:175-200. doi: 10.1159/000418017.	No drug
71	van Kammen DP, Peters J, Yao J, van Kammen WB, Neylan T, Shaw D, Linnoila M. Norepinephrine in acute exacerbations of chronic schizophrenia. Negative symptoms revisited. Arch Gen Psychiatry. 1990;47(2):161-8. doi: 10.1001/archpsyc.1990.01810140061009.	No drug
72	Schwarzkopf SB, Lamberti JS, Jiminez M, Kane CF, Henricks M, Nasrallah HA. Visual evoked potential correlates of positive/negative symptoms in schizophrenia. Biol Psychiatry. 1990;27(4):400-10. doi: 10.1016/0006-3223(90)90550-l.	No drug
73	Adler LE, Waldo MC, Tatcher A, Cawthra E, Baker N, Freedman R. Lack of relationship of auditory gating defects to negative symptoms in schizophrenia. Schizophr Res. 1990;3(2):131-8. doi: 10.1016/0920-9964(90)90046-a.	No drug
74	Newcomer JW, Faustman WO, Yeh W, Csernansky JG. Distinguishing depression and negative symptoms in unmedicated patients with schizophrenia. Psychiatry Res. 1990;31(3):243-50. doi: 10.1016/0165-1781(90)90093-k.	No drug
75	Mizuki Y, Kajimura N, Imai T, Suetsugi M, Kai S, Kaneyuki H, Yamada M. Effects of mianserin on negative symptoms in schizophrenia. Int Clin Psychopharmacol. 1990;5(2):83-95. doi: 10.1097/00004850-199004000-00002.	Open
76	Serban G, George A, Siegel S, DeLeon M, Gaffney M. Computed tomography scans and negative symptoms in schizophrenia: chronic schizophrenics with negative symptoms and nonenlarged lateral ventricles. Acta Psychiatr Scand. 1990;81(5):441-7. doi: 10.1111/j.1600-0447.1990.tb05478.x.	No drug
77	Morrison RL, Bellack AS, Wixted JT, Mueser KT. Positive and negative symptoms in schizophrenia. A cluster-analytic approach. J Nerv Ment Dis. 1990;178(6):377-84. doi: 10.1097/00005053-199006000-00006.	No drug

78	Addington D, Addington J. Depression dexamethasone nonsuppression and negative symptoms in schizophrenia. Can J Psychiatry. 1990;35(5):430-3. doi: 10.1177/070674379003500512.	No drug
79	Tandon R, Goldman RS, Goodson J, Greden JF. Mutability and relationship between positive and negative symptoms during neuroleptic treatment in schizophrenia. Biol Psychiatry. 1990;27(12):1323-6. doi: 10.1016/0006-3223(90)90502-s.	No drug
80	Andreasen NC, Flaum M, Swayze VW 2nd, Tyrrell G, Arndt S. Positive and negative symptoms in schizophrenia. A critical reappraisal. Arch Gen Psychiatry. 1990;47(7):615-21. doi: 10.1001/archpsyc.1990.01810190015002.	No drug
81	Duff V, Olders H. Negative symptoms in schizophrenia. Can J Psychiatry. 1990;35(6):570-1. doi: 10.1177/070674379003500629.	No drug
82	Dworkin RH, Green SR, Small NE, Warner ML, Cornblatt BA, Erlenmeyer-Kimling L. Positive and negative symptoms and social competence in adolescents at risk for schizophrenia and affective disorder. Am J Psychiatry. 1990;147(9):1234-6. doi: 10.1176/ajp.147.9.1234.	No drug
83	Kelly P, Rennie C, Gordon E, Anderson J, Howson A, Meares R. Smooth pursuit eye tracking dysfunction and negative symptoms in schizophrenia. Psychiatry Res. 1990;34(1):89-97. doi: 10.1016/0165-1781(90)90060-i.	No drug
84	Rybakowski J, Linka M. Wpøyw leczenia neuroleptycznego na objawy pozytywne i negatywne schizofrenii oraz wyniki testu deksametazonowego [Effect of neuroleptic treatment on positive and negative symptoms of schizophrenia and the results of the dexamethasone test]. Psychiatr Pol. 1991;25(1):1-6. Polish.	Lumping
85	Goldman RS, Tandon R, Liberzon I, Goodson J, Greden JF. Stability of positive and negative symptom constructs during neuroleptic treatment in schizophrenia. Psychopathology. 1991;24(4):247-52. doi: 10.1159/000284721.	Unfocused
86	Mueser KT, Douglas MS, Bellack AS, Morrison RL. Assessment of enduring deficit and negative symptom subtypes in schizophrenia. Schizophr Bull. 1991;17(4):565-82. doi: 10.1093/schbul/17.4.565.	No drug
87	Schwartz F, Munich RL, Carr A, Bartuch E, Lesser B, Rescigno D, Viegener B. Negative symptoms and reaction time in schizophrenia. J Psychiatr Res. 1991;25(3):131-40. doi: 10.1016/0022-3956(91)90006-v.	No drug
88	Tandon R, Shipley JE, Greden JF, Mann NA, Eisner WH, Goodson JA. Muscarinic cholinergic hyperactivity in schizophrenia. Relationship to positive and negative symptoms. Schizophr Res. 1991;4(1):23-30. doi: 10.1016/0920-9964(91)90006-d.	Open
89	Nakazawa T. Some aspects of negative symptoms of the residual schizophrenia. Yakubutsu Seishin Kodo. 1991;11(1):39-41.	No drug
90	Keefe RS, Lobel DS, Mohs RC, Silverman JM, Harvey PD, Davidson M, Losonczy MF, Davis KL. Diagnostic issues in chronic schizophrenia: kraepelinian schizophrenia, undifferentiated schizophrenia, and state-independent negative symptoms. Schizophr Res. 1991;4(2):71-9. doi: 10.1016/0920-9964(91)90026-n.	No drug
91	Poole JH, Maricq HR, Alson E, Willerman L. Negative symptoms in schizophrenia and nailfold plexus visibility. Biol Psychiatry. 1991;29(8):757-73. doi: 10.1016/0006-3223(91)90195-r.	No drug
92	Addington J, Addington D. Positive and negative symptoms of schizophrenia. Their course and relationship over time. Schizophr Res. 1991;5(1):51-9. doi: 10.1016/0920-9964(91)90053-t.	No drug
93	Williamson P, Pelz D, Merskey H, Morrison S, Conlon P. Correlation of negative symptoms in schizophrenia with frontal lobe parameters on magnetic resonance imaging. Br J Psychiatry. 1991;159:130-4. doi: 10.1192/bjp.159.1.130.	No drug
94	Tandon R, Greden JF. Negative symptoms of schizophrenia: the need for conceptual clarity. Biol Psychiatry. 1991;30(4):321-5. doi: 10.1016/0006-3223(91)90287-v.	No drug
95	Basińska M, Rybakowski J. Test Rorschacha a objawy pozytywne i negatywne schizofrenii [Rorschach test and the positive and negative symptoms of schizophrenia]. Psychiatr Pol. 1991;25(5):16-22. Polish.	No drug
96	Rybakowski J, Linka M, Matkowski K, Kanarkowski R. Test hamowania deksametazonem, a objawy pozytywne i negatywne schizofrenii [Dexamethasone suppression test and the positive and negative symptoms of schizophrenia]. Psychiatr Pol. 1991;25(5):9-15. Polish.	No drug
97	Dworkin RH, Bernstein G, Kaplansky LM, Lipsitz JD, Rinaldi A, Slater SL, Cornblatt BA, Erlenmeyer-Kimling L. Social competence and positive and negative symptoms: a longitudinal study of children and adolescents at risk for schizophrenia and affective disorder. Am J Psychiatry. 1991;148(9):1182-8. doi: 10.1176/ajp.148.9.1182.	No drug
98	Chadda RK. Negative symptoms in schizophrenia. Am J Psychiatry. 1991;148(9):1275-6. doi: 10.1176/ajp.148.9.aj14891275.	No drug
99	Addington J, Addington D, Maticka-Tyndale E. Cognitive functioning and positive and negative symptoms in schizophrenia. Schizophr Res. 1991;5(2):123-34. doi: 10.1016/0920-9964(91)90039-t.	No drug
100	Peralta V, Cuesta MJ, de Leon J. Premorbid personality and positive and negative symptoms in schizophrenia. Acta Psychiatr Scand. 1991;84(4):336-9. doi: 10.1111/j.1600-0447.1991.tb03156.x.	No drug
101	Ring N, Tantam D, Montague L, Morris J. Negative symptoms in chronic schizophrenia. Relationship to duration of illness. Br J Psychiatry. 1991;159:495-9. doi: 10.1192/bjp.159.4.495.	No drug
102	Fenton WS, McGlashan TH. Natural history of schizophrenia subtypes. II. Positive and negative symptoms and long-term course. Arch Gen Psychiatry. 1991;48(11):978-86. doi: 10.1001/archpsyc.1991.01810350018003.	No drug
103	Lee S, Leung H. Negative symptoms of schizophrenia. Br J Psychiatry. 1991;159:728-9. doi: 10.1192/bjp.159.5.728.	No drug
104	Ring N, Tantam D, Montague L, Newby D, Black D, Morris J. Gender differences in the incidence of definite schizophrenia and atypical psychosis—focus on negative symptoms of schizophrenia. Acta Psychiatr Scand. 1991;84(6):489-96. doi: 10.1111/j.1600-0447.1991.tb03182.x.	No drug
105	Goldman RS, Tandon R, Liberzon I, Greden JF. Measurement of depression and negative symptoms in schizophrenia. Psychopathology. 1992;25(1):49-56. doi: 10.1159/000284753.	No drug
106	Lynch MR. Schizophrenia and the D1 receptor: focus on negative symptoms. Prog Neuropsychopharmacol Biol Psychiatry. 1992;16(6):797-832. doi: 10.1016/0278-5846(92)90102-k.	Review
107	Tandon R, DeQuardo JR, Goodson J, Mann NA, Greden JF. Effect of anticholinergics on positive and negative symptoms in schizophrenia. Psychopharmacol Bull. 1992;28(3):297-302.	Review
108	Martinot JL, Paillere-Martinot ML, Loc'h C, Aubin F, Dao-Castellana MH, Deslauriers AG, Syrota A, Lecrubier Y. Récepteurs dopaminergiques centraux et syndrome négatif de la schizophrénie [Central dopaminergic receptors and negative symptoms in schizophrenia]. Ann Méd Psychol (Paris). 1992;150(2-3):127-30. French.	Review
109	Michael A, Mathai PJ, Chaturvedi SK, Gopinath PS. Stability of negative symptoms of schizophrenia. Br J Psychiatry. 1992;160:281. doi: 10.1192/bjp.160.2.281a.	No drug
110	Suzuki E, Kanba S, Nibuya M, Koshikawa H, Nakaki T, Yagi G. Plasma homovanillic acid, plasma anti-D1 and -D2 dopamine-receptor activity, and negative symptoms in chronically mediated schizophrenia. Biol Psychiatry. 1992;31(4):357-64. doi: 10.1016/0006-3223(92)90229-s.	No drug
111	Tandon R, Shipley JE, Taylor S, Greden JF, Eiser A, DeQuardo J, Goodson J. Electroencephalographic sleep abnormalities in schizophrenia. Relationship to positive/negative symptoms and prior neuroleptic treatment. Arch Gen Psychiatry. 1992;49(3):185-94. doi: 10.1001/archpsyc.1992.01820030017003.	No drug
112	de Leon J, Simpson GM, Peralta V. Positive and negative symptoms in schizophrenia: where are the data? Biol Psychiatry. 1992;31(5):431-4. doi: 10.1016/0006-3223(92)90256-y.	No drug
113	Fenton WS, McGlashan TH. Testing systems for assessment of negative symptoms in schizophrenia. Arch Gen Psychiatry. 1992;49(3):179-84. doi: 10.1001/archpsyc.1992.01820030011002.	No drug
114	Perlick D, Mattis S, Stastny P, Silverstein B. Negative symptoms are related to both frontal and nonfrontal neuropsychological measures in chronic schizophrenia. Arch Gen Psychiatry. 1992;49(3):245-6. doi: 10.1001/archpsyc.1992.01820030077010.	No drug
115	Mortimer A, McKenna PJ. Negative symptoms of schizophrenia. Practitioner. 1992;236(1512):255-60.	Opinion

116	Silver H, Nassar A. Fluvoxamine improves negative symptoms in treated chronic schizophrenia: an add-on double-blind, placebo-controlled study. Biol Psychiatry. 1992;31(7):698-704. doi: 10.1016/0006-3223(92)90279-9.	**Included**
117	Perenyi A, Goswami U, Frecska E, Arató M, Bela A. L-deprenyl in treating negative symptoms of schizophrenia. Psychiatry Res. 1992;42(2):189-91. doi: 10.1016/0165-1781(92)90082-e.	Open
118	Marneros A, Andreasen NC. Positive und Negative Symptomatik der Schizophrenie [Positive and negative symptoms of schizophrenia]. Nervenarzt. 1992;63(5):262-70. German.	No drug
119	Catapano F, Maj M, Grimaldi F, Ventra C, Kemali D. Efficacy of low *vs*. high doses of levosulpiride on negative symptoms of schizophrenia. Pharmacopsychiatry. 1992;25(3):166.	Opinion
120	Bermanzohn PC, Siris SG. Akinesia: a syndrome common to parkinsonism, retarded depression, and negative symptoms of schizophrenia. Compr Psychiatry. 1992;33(4):221-32. doi: 10.1016/0010-440x(92)90045-r.	No drug
121	Serban G, Siegel S, Gaffney M. Response of negative symptoms of schizophrenia to neuroleptic treatment. J Clin Psychiatry. 1992;53(7):229-34.	Open
122	Unai Y. Positive and negative symptoms in schizophrenia: application of Jacksonism. Keio J Med. 1992;41(3):168-76. doi: 10.2302/kjm.41.168.	No drug
123	Husted JA, Beiser M, Iacono WG. Negative symptoms and the early course of schizophrenia. Psychiatry Res. 1992;43(3):215-22. doi: 10.1016/0165-1781(92)90054-7.	No drug
124	Dworkin RH, Opler LA. Simple schizophrenia, negative symptoms, and prefrontal hypodopaminergia. Am J Psychiatry. 1992;149(9):1284-5. doi: 10.1176/ajp.149.9.aj14991284.	No drug
125	Kelley ME, Gilbertson M, Mouton A, van Kammen DP. Deterioration in premorbid functioning in schizophrenia: a developmental model of negative symptoms in drug-free patients. Am J Psychiatry. 1992;149(11):1543-8. doi: 10.1176/ajp.149.11.1543.	No drug
126	Baron M, Gruen RS, Romo-Gruen JM. Positive and negative symptoms. Relation to familial transmission of schizophrenia. Br J Psychiatry. 1992;161:610-4. doi: 10.1192/bjp.161.5.610.	No drug
127	Keefe RS, Harvey PD, Lenzenweger MF, Davidson M, Apter SH, Schmeidler J, Mohs RC, Davis KL. Empirical assessment of the factorial structure of clinical symptoms in schizophrenia: negative symptoms. Psychiatry Res. 1992;44(2):153-65. doi: 10.1016/0165-1781(92)90049-9.	No drug
128	McLaren S, Cookson JC, Silverstone T. Positive and negative symptoms, depression and social disability in chronic schizophrenia: a comparative trial of bromperidol and fluphenazine decanoates. Int Clin Psychopharmacol. 1992;7(2):67-72.	Unfocused
129	Wolkin A, Sanfilipo M, Wolf AP, Angrist B, Brodie JD, Rotrosen J. Negative symptoms and hypofrontality in chronic schizophrenia. Arch Gen Psychiatry. 1992;49(12):959-65. doi: 10.1001/archpsyc.1992.01820120047007.	No drug
130	Kane JM, Dauphinais D, Barnes TR, Adler LA, Rifkin A. Assessing negative symptoms and extrapyramidal symptoms in schizophrenia: workshop report. Psychopharmacol Bull. 1993;29(1):45-9.	No drug
131	Raskin A, Pelchat R, Sood R, Alphs LD, Levine J. Negative symptom assessment of chronic schizophrenia patients. Schizophr Bull. 1993;19(3):627-35. doi: 10.1093/schbul/19.3.627.	No drug
132	Kuruvilla A, Srikrishna G, Peedicayil J, Kuruvilla K, Kanagasabapathy AS. A study on serum prolactin levels in schizophrenia: correlation with positive and negative symptoms. Int Clin Psychopharmacol. 1993;8(3):177-9. doi: 10.1097/00004850-199300830-00007.	No drug
133	Ohmori T, Koyama T, Inoue T, Matsubara S, Yamashita I. B-HT 920, a dopamine D2 agonist, in the treatment of negative symptoms of chronic schizophrenia. Biol Psychiatry. 1993;33(10):687-93. doi: 10.1016/0006-3223(93)90117-v.	Open
134	Nachshoni T, Levy A, Levin Y, Neumann M. [Negative symptoms in schizophrenia]. Harefuah. 1993;124(11):692-5. Hebrew.	Review
135	Sonobe N. Eye movements, verbal material recall and negative symptoms in chronic schizophrenia. Int J Psychophysiol. 1993;15(1):73-8. doi: 10.1016/0167-8760(93)90098-a.	No drug
136	Rosse RB, Kleinberg AW, Deutsch SI. Positive and negative symptoms of schizophrenia as predictors of length of military service: a retrospective study. Mil Med. 1993;158(8):529-33.	No drug
137	Poole JH, Maricq HR, Willerman L. Negative symptoms of schizophrenia and plexus visibility: a replication with medicated and unmedicated patients. Biol Psychiatry. 1993;34(6):414-6. doi: 10.1016/0006-3223(93)90187-i.	No drug
138	Duinkerke SJ, Botter PA, Jansen AA, van Dongen PA, van Haaften AJ, Boom AJ, van Laarhoven JH, Busard HL. Ritanserin, a selective 5-HT2/1C antagonist, and negative symptoms in schizophrenia. A placebo-controlled double-blind trial. Br J Psychiatry. 1993;163:451-5. doi: 10.1192/bjp.163.4.451.	Non-marketed
139	Tandon R, Ribeiro SC, DeQuardo JR, Goldman RS, Goodson J, Greden JF. Covariance of positive and negative symptoms during neuroleptic treatment in schizophrenia: a replication. Biol Psychiatry. 1993;34(7):495-7. doi: 10.1016/0006-3223(93)90242-6.	Open
140	Rupniak NM, Iversen SD. Cognitive impairment in schizophrenia: how experimental models using nonhuman primates may assist improved drug therapy for negative symptoms. Neuropsychologia. 1993;31(10):1133-46. doi: 10.1016/0028-3932(93)90037-z.	Animal
141	Risby ED, Jewart RD, Lewine RR, Risch SC, Stipetic M, McDaniel JS, Caudle J. An association between increased concentrations of cerebrospinal fluid dopamine sulfate and higher negative symptom scores in patients with schizophrenia and schizoaffective disorder. Biol Psychiatry. 1993;34(9):661-4. doi: 10.1016/0006-3223(93)90160-f.	No drug
142	Malla AK, Norman RM, Williamson P. Stability of positive and negative symptoms in schizophrenia. Can J Psychiatry. 1993;38(9):617-21. doi: 10.1177/070674379303800910.	No drug
143	Liddle PF, Barnes TR, Speller J, Kibel D. Negative symptoms as a risk factor for tardive dyskinesia in schizophrenia. Br J Psychiatry. 1993;163:776-80. doi: 10.1192/bjp.163.6.776.	No drug
144	Bassett AS, Collins EJ, Nuttall SE, Honer WG. Positive and negative symptoms in families with schizophrenia. Schizophr Res. 1993;11(1):9-19. doi: 10.1016/0920-9964(93)90033-f.	No drug
145	Garyfallos G, Lavrentiadis G, Amoutzias D, Monas K, Manos N. Negative symptoms of schizophrenia and the dexamethasone suppression test Acta Psychiatr Scand. 1993;88(6):425-8. doi: 10.1111/j.1600-0447.1993.tb03485.x.	No drug
146	Rao ML, Möller HJ. Biochemical findings of negative symptoms in schizophrenia and their putative relevance to pharmacologic treatment. A review. Neuropsychobiology. 1994;30(4):160-72. doi: 10.1159/000119156.	Review
147	Andreasen NC, Nopoulos P, Schultz S, Miller D, Gupta S, Swayze V, Flaum M. Positive and negative symptoms of schizophrenia: past, present, and future. Acta Psychiatr Scand. 1994;90(Suppl. 384):51-9. doi: 10.1111/j.1600-0447.1994.tb05891.x.	No drug
148	Nachshoni T, Levin Y, Levy A, Kritz A, Neumann M. A double-blind trial of carbamazepine in negative symptom schizophrenia. Biol Psychiatry. 1994;35(1):22-6. doi: 10.1016/0006-3223(94)91162-2.	**Included**
149	Schooler NR. Deficit symptoms in schizophrenia: negative symptoms versus neuroleptic-induced deficits. Acta Psychiatr Scand. 1994;89(Suppl. 380):21-6. doi: 10.1111/j.1600-0447.1994.tb05827.x.	No drug
150	Palao DJ, Arauxo A, Brunet M, Marquez M, Bernardo M, Ferrer J, Gonzalez- Monclus E. Positive versus negative symptoms in schizophrenia: response to haloperidol. Prog Neuropsychopharmacol Biol Psychiatry. 1994;18(1):155-64. doi: 10.1016/0278-5846(94)90032-9.	Inadequate
151	Martinot JL, Paillère-Martinot ML, Loc'h C, Lecrubier Y, Dao-Castellana MH, Aubin F, Allilaire JF, Mazoyer B, Mazière B, Syrota A. Central D2 receptors and negative symptoms of schizophrenia. Br J Psychiatry. 1994;164(1):27-34. doi: 10.1192/bjp.164.1.27.	No drug
152	Breier A, Buchanan RW, Kirkpatrick B, Davis OR, Irish D, Summerfelt A, Carpenter WT Jr. Effects of clozapine on positive and negative symptoms in outpatients with schizophrenia. Am J Psychiatry. 1994;151(1):20-6. doi: 10.1176/ajp.151.1.20.	**Included**
153	Harris MJ, McAdams LA, Heaton SC. Negative symptoms in late-life schizophrenia. Am J Geriatr Psychiatry. 1994;2(1):9-20. doi: 10.1097/00019442-199400210-00004.	No drug

154	Lysaker P, Bell M, Beam-Goulet J, Milstein R. Relationship of positive and negative symptoms to cocaine abuse in schizophrenia. J Nerv Ment Dis. 1994;182(2):109-12. doi: 10.1097/00005053-199402000-00008.	No drug
155	Kuniyoshi M, Nakamura J, Miura C, Inanaga K. Effectiveness of concomitant setiptiline maleate (Tecipul®) on negative symptoms of schizophrenia. Prog Neuropsychopharmacol Biol Psychiatry. 1994;18(2):339-46. doi: 10.1016/0278-5846(94)90065-5.	Open
156	Eikmeier G, Lodemann E. PINV--topography and primary negative symptoms in chronic schizophrenia. Pharmacopsychiatry. 1994;27(2):63-4. doi: 10.1055/s-2007-1014278.	No drug
157	Roff JD, Knight RA. Birth complications and subsequent negative symptoms in schizophrenia. Psychol Rep. 1994;74(2):635-41. doi: 10.2466/pr0.1994.74.2.635.	No drug
158	Schooler NR. Negative symptoms in schizophrenia: assessment of the effect of risperidone. J Clin Psychiatry. 1994;55(Suppl):22-8.	Opinion
159	Möller H-J, van Praag HM, Aufdembrinke B, Bailey P, Barnes TR, Beck J, Bentsen H, Eich FX, Farrow L, Fleischhacker WW, et al. Negative symptoms in schizophrenia: considerations for clinical trials. Working group on negative symptoms in schizophrenia. Psychopharmacology (Berl). 1994;115(1-2):221-8. doi: 10.1007/BF02244775.	Review
160	Klosterkötter J, Albers M, Steinmeyer EM, Hensen A, Sass H. Positive oder negative Symptome. Was ist brauchbarer für die Diagnose Schizophrenie? [Positive or negative symptoms. Which are more reliable in the diagnosis of schizophrenia?]. Nervenarzt. 1994;65(7):444-53. German.	No drug
161	Brekke JS, DeBonis JA, Graham JW. A latent structure analysis of the positive and negative symptoms in schizophrenia. Compr Psychiatry. 1994;35(4):252-9. doi: 10.1016/0010-440x(94)90016-7.	No drug
162	Miller DD, Flaum M, Arndt S, Fleming F, Andreasen NC. Effect of antipsychotic withdrawal on negative symptoms in schizophrenia. Neuropsychopharmacology. 1994;11(1):11-20. doi: 10.1038/npp.1994.31.	No drug
163	Javitt DC, Zylberman I, Zukin SR, Heresco-Levy U, Lindenmayer JP. Amelioration of negative symptoms in schizophrenia by glycine. Am J Psychiatry. 1994;151(8):1234-6. doi: 10.1176/ajp.151.8.1234.	**Included**
164	Igata M, Ohta M, Hayashida Y, Abe K. Missing peaks in auditory brainstem responses and negative symptoms in schizophrenia. Jpn J Psychiatry Neurol. 1994;48(3):571-8. doi: 10.1111/j.1440-1819.1994.tb03016.x.	No drug
165	Roy MA, DeVriendt X. Symptômes positifs et négatifs de la schizophrénie: une mise à jour [Positive and negative symptoms in schizophrenia: a current overview]. Can J Psychiatry. 1994;39(7):407-14. French. doi: 10.1177/070674379403900704.	Review
166	Castellon SA, Asarnow RF, Goldstein MJ, Marder SR. Persisting negative symptoms and information-processing deficits in schizophrenia: implications for subtyping. Psychiatry Res. 1994;54(1):59-69. doi: 10.1016/0165-1781(94)90065-5.	No drug
167	Martyns-Yellowe IS. The positive and negative symptoms of schizophrenia: patterns of response to depot neuroleptic treatment. West Afr J Med. 1994;13(4):200-3.	Open
168	Bermanzohn PC, Siris SG. Battle against therapeutic nihilism in caring for patients who exhibit the negative symptoms of schizophrenia (NSS). Compr Psychiatry. 1994;35(6):478. doi: 10.1016/0010-440x(94)90232-1.	No drug
169	Shioiri T, Kato T, Inubushi T, Murashita J, Takahashi S. Correlations of phosphomonoesters measured by phosphorus-31 magnetic resonance spectroscopy in the frontal lobes and negative symptoms in schizophrenia. Psychiatry Res. 1994;55(4):223-35. doi: 10.1016/0165-1781(95)91247-B.	No drug
170	Decina P, Mukherjee S, Bocola V, Saraceni F, Hadjichristos C, Scapicchio P. Adjunctive trazodone in the treatment of negative symptoms of schizophrenia. Hosp Community Psychiatry. 1994;45(12):1220-3. doi: 10.1176/ps.45.12.1220.	**Included**
171	Gerbaldo H, Cassady S, Helisch A. Negative symptoms and the course of positive symptoms in deficit schizophrenia. Psychopathology. 1995;28(3):121-6. doi: 10.1159/000284910.	No drug
172	Peralta V, Cuesta MJ, de Leon J. Positive and negative symptoms/syndromes in schizophrenia: reliability and validity of different diagnostic systems. Psychol Med. 1995;25(1):43-50. doi: 10.1017/s0033291700028075.	No drug
173	Crow TJ. Brain changes and negative symptoms in schizophrenia. Psychopathology. 1995;28(1):18-21. doi: 10.1159/000284895.	No drug
174	Mundt C, Barnett W, Witt G. The core of negative symptoms in schizophrenia: affect or cognitive deficiency? Psychopathology. 1995;28(1):46-54. doi: 10.1159/000284899.	No drug
175	Hwu HG, Tan H, Chen CC, Yeh LL. Negative symptoms at discharge and outcome in schizophrenia. Br J Psychiatry. 1995;166(1):61-7. doi: 10.1192/bjp.166.1.61.	No drug
176	Boyer P, Lecrubier Y, Puech AJ, Dewailly J, Aubin F. Treatment of negative symptoms in schizophrenia with amisulpride. Br J Psychiatry. 1995;166(1):68-72. doi: 10.1192/bjp.166.1.68.	**Included**
177	Eaton WW, Thara R, Federman B, Melton B, Liang KY. Structure and course of positive and negative symptoms in schizophrenia. Arch Gen Psychiatry. 1995;52(2):127-34. doi: 10.1001/archpsyc.1995.03950140045005.	No drug
178	Slaghuis WL, Bakker VJ. Forward and backward visual masking of contour by light in positive- and negative-symptom schizophrenia. J Abnorm Psychol. 1995;104(1):41-54. doi: 10.1037//0021-843x.104.1.41.	No drug
179	Lysaker P, Bell M. Negative symptoms and vocational impairment in schizophrenia: repeated measurements of work performance over six months. Acta Psychiatr Scand. 1995;91(3):205-8. doi: 10.1111/j.1600-0447.1995.tb09768.x.	No drug
180	Lysaker PH, Bell MD, Bioty SM, Zito WS. The frequency of associations between positive and negative symptoms and dysphoria in schizophrenia. Compr Psychiatry. 1995;36(2):113-7. doi: 10.1016/s0010-440x(95)90105-1.	No drug
181	Ellason JW, Ross CA. Positive and negative symptoms in dissociative identity disorder and schizophrenia: a comparative analysis. J Nerv Ment Dis. 1995;183(4):236-41. doi: 10.1097/00005053-199504000-00009.	No drug
182	Lagomarsino AJ. Síntomas positivos y negativos de la esquizofrenia [Positive and negative symptoms of schizophrenia]. Acta Psiquiatr Psicol Am Lat. 1995;41(2):141-7. Spanish.	Review
183	Miller AL, Mahurin RK, Velligan DI, Maas JW. Negative symptoms of schizophrenia: where do we go from here? Biol Psychiatry. 1995;37(10):691-3. doi: 10.1016/0006-3223(95)00021-8.	Opinion
184	Dollfus S, Langlois S, Assouly-Besse F, Petit M. Symptômes dépressifs et symptômes négatifs au cours des schizophrénies [Depressive symptoms and negative symptoms during schizophrenia]. Encéphale. 1995;21(Spec No 3):23-7. French.	Review
185	Jalenques I. Approche psychopharmacologique des symptômes négatifs dans les schizophrénies [Pharmacologic approach to negative symptoms in schizophrenia]. Encéphale. 1995;21(Spec No 3):35-40. French.	Review
186	Waddington JL, Youssef HA, Kinsella A. Sequential cross-sectional and 10-year prospective study of severe negative symptoms in relation to duration of initially untreated psychosis in chronic schizophrenia. Psychol Med. 1995;25(4):849-57. doi: 10.1017/s0033291700035108.	No drug
187	Bustillo JR, Kirkpatrick B, Buchanan RW. Neuroleptic treatment and negative symptoms in deficit and nondeficit schizophrenia. Biol Psychiatry. 1995;38(1):64-7. doi: 10.1016/0006-3223(95)00144-6.	Open
188	Dosiak M. Schizofrenia--jedna czy dwie psychozy? Objawy pozytywne i negatywne [Schizophrenia: one or two psychoses? Positive and negative symptoms]. Psychiatr Pol. 1995;29(4):455-64. Polish.	No drug
189	Gerbaldo H, Fickinger MP, Wetzel H, Helisch A, Philipp M, Benkert O. Primary enduring negative symptoms in schizophrenia and major depression. J Psychiatr Res. 1995;29(4):297-302. doi: 10.1016/0022-3956(95)00013-u.	No drug
190	Goff DC, Tsai G, Manoach DS, Coyle JT. Dose-finding trial of D-cycloserine added to neuroleptics for negative symptoms in schizophrenia. Am J Psychiatry. 1995;152(8):1213-5. doi: 10.1176/ajp.152.8.1213.	Inadequate
191	Brieger P, Rohde A. "Maskierte" oder "verheimlichte" Schizophrenie: Erstdiagnose einer 12 Jahre bestehenden Psychose anhand negativer Symptome ["Masked" or "hidden" schizophrenia: initial diagnosis of a 12 year psychoses based on negative symptoms]. Psychiatr Prax. 1995;22(5):213-4. German.	No drug

192	Fennig S, Putnam K, Bromet EJ, Galambos N. Gender, premorbid characteristics and negative symptoms in schizophrenia. Acta Psychiatr Scand. 1995;92(3):173-7. doi: 10.1111/j.1600-0447.1995.tb09563.x.	No drug
193	Palacios-Araus L, Herrán A, Sandoya M, González de la Huebra E, Vázquez- Barquero JL, Díez-Manrique JF. Analysis of positive and negative symptoms in schizophrenia. A study from a population of long-term outpatients. Acta Psychiatr Scand. 1995;92(3):178-82. doi: 10.1111/j.1600-0447.1995.tb09564.x.	No drug
194	Malla AK. Negative symptoms and affective disturbance in schizophrenia and related disorders. Can J Psychiatry. 1995;40(7 Suppl 2):S55-9. doi: 10.1177/070674379504007s05.	Review
195	Donnoli VF, Salvatore A, Basotto J, Ventura A, Cohen D, Fantin Graiño A, Oliva D, Martins J. Esquizofrenia: relación entre síntomas negativos y rasgos esquizotípicos de personalidad [Schizophrenia: relationship between negative symptoms and schizotypic features of personality]. Acta Psiquiatr Psicol Am Lat. 1995;41(3):214-8. Spanish.	No drug
196	Möller HJ. The psychopathology of schizophrenia: an integrated view on positive symptoms and negative symptoms. Int Clin Psychopharmacol. 1995;10(Suppl 3):57-64.	Review
197	Barnes TR, McPhillips MA. How to distinguish between the neuroleptic- induced deficit syndrome, depression and disease-related negative symptoms in schizophrenia. Int Clin Psychopharmacol. 1995;10(Suppl 3):115-21.	Review
198	Peralta V, Cuesta MJ. Negative symptoms in schizophrenia: a confirmatory factor analysis of competing models. Am J Psychiatry. 1995;152(10):1450-7. doi: 10.1176/ajp.152.10.1450.	No drug
199	Dollfus S, Petit M. Negative symptoms in schizophrenia: their evolution during an acute phase. Schizophr Res. 1995;17(2):187-94. doi: 10.1016/0920-9964(94)00087-o.	No drugs
200	Den Boer JA, van Megen HJGM, Fleischhacker WW, Louwerens JW, Slaap BR, Westenberg HGM, Burrows GD, Srivastava ON. Differential effects of the D1-DA receptor antagonist SCH39166 on positive and negative symptoms of schizophrenia. Psychopharmacology (Berl). 1995;121(3):317-22. doi: 10.1007/BF02246069.	Open
201	Klosterkötter J, Albers M, Steinmeyer EM, Hensen A, Sass H. Positive or negative symptoms--which are more appropriate as diagnostic criteria for schizophrenia? Acta Psychiatr Scand. 1995;92(5):321-6. doi: 10.1111/j.1600-0447.1995.tb09592.x.	No drug
202	Carman J, Peuskens J, Vangeneugden A. Risperidone in the treatment of negative symptoms of schizophrenia: a meta-analysis. Int Clin Psychopharmacol. 1995;10(4):207-13. doi: 10.1097/00004850-199511000-00001.	Review
203	Marchesi GF, Santone G, Cotani P, Giordano A, Chelli F. The therapeutic role of naltrexone in negative symptom schizophrenia. Prog Neuropsychopharmacol Biol Psychiatry. 1995;19(8):1239-49. doi: 10.1016/0278-5846(95)00263-4.	**Included**
204	Sanfilipo M, Wolkin A, Angrist B, van Kammen DP, Duncan E, Wieland S, Cooper TB, Peselow ED, Rotrosen J. Amphetamine and negative symptoms of schizophrenia. Psychopharmacology (Berl). 1996;123(2):211-4. doi: 10.1007/BF02246180.	No drug
205	Loas G, Boyer P, Legrand A. Anhedonia and negative symptomatology in chronic schizophrenia. Compr Psychiatry. 1996;37(1):5-11. doi: 10.1016/s0010-440x(96)90043-7.	No drug
206	Zemishlany Z, Alexander GE, Prohovnik I, Goldman RG, Mukherjee S, Sackeim H. Cortical blood flow and negative symptoms in schizophrenia. Neuropsychobiology. 1996;33(3):127-31. doi: 10.1159/000119262.	No drug
207	Volavka J, Cooper TB, Czobor P, Meisner M. Effect of varying haloperidol plasma levels on negative symptoms in schizophrenia and schizoaffective disorder. Psychopharmacol Bull. 1996;32(1):75-9.	Open
208	Chen EY, Lam LC, Chen RY, Nguyen DG. Negative symptoms, neurological signs and neuropsychological impairments in 204 Hong Kong Chinese patients with schizophrenia. Br J Psychiatry. 1996;168(2):227-33. doi: 10.1192/bjp.168.2.227.	No drug
209	Leiderman E, Zylberman I, Zukin SR, Cooper TB, Javitt DC. Preliminary investigation of high-dose oral glycine on serum levels and negative symptoms in schizophrenia: an open-label trial. Biol Psychiatry. 1996;39(3):213-5. doi: 10.1016/0006-3223(95)00585-4.	No drug
210	Kajimura N, Kato M, Okuma T, Sekimoto M, Watanabe T, Takahashi K. Relationship between delta activity during all-night sleep and negative symptoms in schizophrenia: a preliminary study. Biol Psychiatry. 1996;39(6):451-4. doi: 10.1016/0006-3223(95)00376-2.	No drug
211	Bodkin JA, Cohen BM, Salomon MS, Cannon SE, Zornberg GL, Cole JO. Treatment of negative symptoms in schizophrenia and schizoaffective disorder by selegiline augmentation of antipsychotic medication. A pilot study examining the role of dopamine. J Nerv Ment Dis. 1996;184(5):295-301. doi: 10.1097/00005053-199605000-00005.	Open
212	Pélissolo A, Krebs MO, Olié JP. Traitement des symptômes déficitaires de la schizophrénie par l'amisulpride. Revue de la littérature [Treatmen of negative symptoms in schizophrenia by amisulpride. Review of the literature]. Encéhale. 1996;22(3):215-9. French.	Review
213	Merrin EL, Floyd TC. Negative symptoms and EEG alpha in schizophrenia: a replication. Schizophr Res. 1996;19(2-3):151-61. doi: 10.1016/0920-9964(96)88522-7.	No drug
214	Ribeyre JM, Dollfus S. Stabilité des symptômes positifs/négatifs des schizophrénies [Stability of positive/negative symptoms in schizophrenia]. Encéphale. 1996;22(Spec No 2):29-31. French.	Review
215	Vázquez-Barquero JL, Lastra I, Cuesta Nuñez MJ, Herrera Castanedo S, Dunn G. Patterns of positive and negative symptoms in first episode schizophrenia. Br J Psychiatry. 1996;168(6):693-701. doi: 10.1192/bjp.168.6.693	No drug
216	Yuen O, Caligiuri MP, Williams R, Dickson RA. Tardive dyskinesia and positive and negative symptoms of schizophrenia. A study using instrumental measures. Br J Psychiatry. 1996;168(6):702-8. doi: 10.1192/bjp.168.6.702.	No drug
217	Noda Y, Nabeshima T. [Behavioral assessment of neuroleptics (3)--Schizophrenia negative symptoms-like model induced by PCP]. Nihon Shinkei Seishin Yakurigaku Zasshi. 1996;16(4):123-8. Japanese.	No drug
218	Thomas P. Syntactic complexity and negative symptoms in first onset schizophrenia. Cogn Neuropsychiatry. 1996;1(3):191-200. doi: 10.1080/135468096396497.	No drug
219	Smith RC, Chua JW, Lipetsker B, Bhattacharyya A. Efficacy of risperidone in reducing positive and negative symptoms in medication-refractory schizophrenia: an open prospective study. J Clin Psychiatry. 1996;57(10):460-6. doi: 10.4088/jcp.v57n1004.	Open
220	Carter C, Robertson L, Nordahl T, Chaderjian M, Kraft L, O'Shora-Celaya L. Spatial working memory deficits and their relationship to negative symptoms in unmedicated schizophrenia patients. Biol Psychiatry. 1996;40(9):930-2. doi: 10.1016/S0006-3223(96)00350-2.	No drug
221	Brewer WJ, Edwards J, Anderson V, Robinson T, Pantelis C. Neuropsychological, olfactory, and hygiene deficits in men with negative symptom schizophrenia. Biol Psychiatry. 1996;40(10):1021-31. doi: 10.1016/0006-3223(95)00594-3.	No drug
222	Satoh K, Someya T, Shibasaki M. Effects of nemonapride on positive and negative symptoms of schizophrenia. Int Clin Psychopharmacol. 1996;11(4):279-81. doi: 10.1097/00004850-199612000-00010.	Open
223	Lysaker PH, Bell MD, Bioty SM, Zito WS. Cognitive impairment and substance abuse history as predictors of the temporal stability of negative symptoms in schizophrenia. J Nerv Ment Dis. 1997;185(1):21-6. doi: 10.1097/00005053-199701000-00004.	No drug
224	Rossi A, Mancini F, Stratta P, Mattei P, Gismondi R, Pozzi F, Casacchia M. Risperidone, negative symptoms and cognitive deficit in schizophrenia: an open study. Acta Psychiatr Scand. 1997;95(1):40-3. doi: 10.1111/j.1600-0447.1997.tb00371.x.	Open
225	Loo H, Poirier-Littre MF, Theron M, Rein W, Fleurot O. Amisulpride versus placebo in the medium-term treatment of the negative symptoms of schizophrenia. Br J Psychiatry. 1997;170:18-22. doi: 10.1192/bjp.170.1.18.	**Included**
226	Mueser KT, Valentiner DP, Agresta J. Coping with negative symptoms of schizophrenia: patient and family perspectives. Schizophr Bull. 1997;23(2):329-39. doi: 10.1093/schbul/23.2.329.	No drug
227	Szafrański T, Jarema M, Białek J, Buksowicz M, Marciniak E, Choma ME, Ruzikowska A, Milej M. Ocena wpływu terapeutycznego octanu zuklopentyksolu na objawy pozytywne i negatywne w schizofrenii [Therapeutic effect of zuclopenthixol acetate on positive and negative symptoms in schizophrenia]. Psychiatr Pol. 1997;31(1):55-69. Polish.	Open

228	Hwu HG, Wu YC, Lee SF, Yeh LL, Gwo SC, Hsu HC, Chang CJ, Chen WJ. Concordance of positive and negative symptoms in coaffected sib-pairs with schizophrenia. Am J Med Genet. 1997;74(1):1-6.	No drug
229	Kopelowicz A, Liberman RP, Mintz J, Zarate R. Comparison of efficacy of social skills training for deficit and nondeficit negative symptoms in schizophrenia. Am J Psychiatry. 1997;154(3):424-5. doi: 10.1176/ajp.154.3.424.	No drug
230	Spalletta G, Pasini A, De Angelis F, Troisi A. Patients with deficit, nondeficit, and negative symptom schizophrenia: do they differ during episodes of acute psychotic decompensation? Schizophr Res. 1997;24(3):341-8. doi: 10.1016/s0920-9964(96)00124-7.	No drug
231	Berman I, Viegner B, Merson A, Allan E, Pappas D, Green AI. Differential relationships between positive and negative symptoms and neuropsychological deficits in schizophrenia. Schizophr Res. 1997;25(1):1-10. doi: 10.1016/S0920-9964(96)00098-9.	No drug
232	Tugg LA, Desai D, Prendergast P, Remington G, Reed K, Zipursky RB. Relationship between negative symptoms in chronic schizophrenia and neuroleptic dose, plasma levels and side effects. Schizophr Res. 1997;25(1):71-8. doi: 10.1016/s0920-9964(97)00009-1.	Open
233	Awad AG, Lapierre YD, Angus C, Rylander A. Quality of life and response of negative symptoms in schizophrenia to haloperidol and the atypical antipsychotic remoxipride. The Canadian Remoxipride Group. J Psychiatry Neurosci. 1997;22(4):244-8.	**Included**
234	Provencher HL, Mueser KT. Positive and negative symptom behaviors and caregiver burden in the relatives of persons with schizophrenia. Schizophr Res. 1997;26(1):71-80. doi: 10.1016/S0920-9964(97)00043-1.	No drugs
235	Scully PJ, Coakley G, Kinsella A, Waddington JL. Executive (frontal) dysfunction and negative symptoms in schizophrenia: apparent gender differences in 'static' v. 'progressive' profiles. Br J Psychiatry. 1997;171:154-8. doi: 10.1192/bjp.171.2.154.	No drugs
236	Roitman SE, Keefe RS, Harvey PD, Siever LJ, Mohs RC. Attentional and eye tracking deficits correlate with negative symptoms in schizophrenia. Schizophr Res. 1997;26(2-3):139-46. doi: 10.1016/s0920-9964(97)00044-3.	No drugs
237	Brébion G, Smith MJ, Amador X, Malaspina D, Gorman JM. Clinical correlates of memory in schizophrenia: differential links between depression, positive and negative symptoms, and two types of memory impairment. Am J Psychiatry. 1997;154(11):1538-43. doi: 10.1176/ajp.154.11.1538.	No drugs
238	McCreadie RG, Latha S, Thara R, Padmavathi R, Ayankaran JR. Poor memory, negative symptoms and abnormal movements in never-treated Indian patients with schizophrenia. Br J Psychiatry. 1997;171:360-3. doi: 10.1192/bjp.171.4.360.	No drugs
239	McAdams LA, Harris MJ, Heaton SC, Bailey A, Fell R, Jeste DV. Validity of specific subscales of the positive and negative symptom scales in older schizophrenia outpatients. Schizophr Res. 1997;27(2-3):219-26. doi: 10.1016/S0920-9964(97)00066-2.	No drugs
240	King DJ. Drug treatment of the negative symptoms of schizophrenia. Eur Neuropsychopharmacol. 1998;8(1):33-42. doi: 10.1016/s0924-977x(97)00041-2.	Review
241	Nkam I, Langlois-Thery S, Dollfus S, Petit M. L'alexithymie chez des schizophrènes déficitaires et non déficitaires [Alexithymia in negative symptom and non-negative symptom schizophrenia]. Encéphale. 1997;23(5):358-63. French.	No drug
242	Dassori AM, Miller AL, Velligan D, Saldana D, Diamond P, Mahurin R. Ethnicity and negative symptoms in patients with schizophrenia. Cult Divers Ment Health. 1998;4(1):65-9.	No drug
243	Puri BK, Richardson AJ. Sustained remission of positive and negative symptoms of schizophrenia following treatment with eicosapentaenoic acid. Arch Gen Psychiatry. 1998;55(2):188-9. doi: 10.1001/archpsyc.55.2.188.	Case
244	Slaghuis WL. Contrast sensitivity for stationary and drifting spatial frequency gratings in positive- and negative-symptom schizophrenia. J Abnorm Psychol. 1998;107(1):49-62. doi: 10.1037//0021-843x.107.1.49.	No drug
245	Brar JS, Chengappa KN, Parepally H, Sandman AR, Kreinbrook SB, Sheth SA, Ganguli R. The effects of clozapine on negative symptoms in patients with schizophrenia with minimal positive symptoms. Ann Clin Psychiatry. 1997;9(4):227-34. doi: 10.1023/a:1022352326334.	Open
246	Speller JC, Barnes TR, Curson DA, Pantelis C, Alberts JL. One-year, low- dose neuroleptic study of in-patients with chronic schizophrenia characterised by persistent negative symptoms. Amisulpride v. haloperidol. Br J Psychiatry. 1997;171:564-8. doi: 10.1192/bjp.171.6.564.	**Included**
247	Knable MB, Egan MF, Heinz A, Gorey J, Lee KS, Coppola R, Weinberger DR. Altered dopaminergic function and negative symptoms in drug-free patients with schizophrenia. [123I]-iodobenzamide SPECT study. Br J Psychiatry. 1997;171:574-7. doi: 10.1192/bjp.171.6.574.	No drug
248	Figuerido JL, Gutiérrez M, González Pinto A, Ballesteros J, Ramírez F, Elizagarate E, González Oliveros R, López P, Pérez de Heredia JL. Síntomas positivos y negativos en la esquizofrenia: patrones de cambio durante la exacerbación aguda [Positive and negative symptoms in schizophrenia: standards of change during acute exacerbation]. Actas Luso Esp Neurol Psiquiatr Cienc Afines. 1997;25(5):295-302. Spanish.	No drug
249	Allan ER, Sison CE, Alpert M, Connolly B, Crichton J. The relationship between negative symptoms of schizophrenia and extrapyramidal side effects with haloperidol and olanzapine. Psychopharmacol Bull. 1998;34(1):71-4.	Unfocused
250	Karakuła H, Grzywa A. Objawy depresyjne w aktywnej fazie schizofrenii paranoidalnej. Cześć I: Powiazania z objawami negatywnymi [Depressive symptoms in active phase of paranoid schizophrenia. Part I: Their link to negative symptoms]. Psychiatr Pol. 1998;32(1):15-23. Polish. PMID: 9594580.	No drug
251	Silver H, Shmugliakov N. Augmentation with fluvoxamine but not maprotiline improves negative symptoms in treated schizophrenia: evidence for a specific serotonergic effect from a double-blind study. J Clin Psychopharmacol. 1998;18(3):208-11. doi: 10.1097/00004714-199806000-00005.	Overlap= 116
252	Tamminga CA, Buchanan RW, Gold JM. The role of negative symptoms and cognitive dysfunction in schizophrenia outcome. Int Clin Psychopharmacol. 1998;13(Suppl 3):S21-6. doi: 10.1097/00004850-199803003-00004.	No drug
253	Suslow T, Junghanns K, Weitzsch C, Arolt V. Relations between neuropsychological vulnerability markers and negative symptoms in schizophrenia. Psychopathology. 1998;31(4):178-87. doi: 10.1159/000029038.	No drug
254	Gray R. Olanzapine: efficacy in treating the positive and negative symptoms of schizophrenia. Ment Health Care. 1998;1(6):193-4.	Opinion
255	Acuña MJ, Martín J, Noval D, Blanco M. Estudio crítico de la influencia de los síntomas negativos en la respuesta terapeútica de la esquizofrenia [A critical study of the influence of negative symptoms on the therapeutic response in schizophrenia]. Actas Luso Esp Neurol Psiquiatr Cienc Afines. 1998;26(4):209-13. Spanish.	Open
256	Heresco-Levy U, Javitt DC, Ermilov M, Mordel C, Silipo G, Lichtenstein M. Efficacy of high-dose glycine in the treatment of enduring negative symptoms of schizophrenia. Arch Gen Psychiatry. 1999;56(1):29-36. doi: 10.1001/archpsyc.56.1.29.	**Included**
257	Rosenheck R, Dunn L, Peszke M, Cramer J, Xu W, Thomas J, Charney D. Impact of clozapine on negative symptoms and on the deficit syndrome in refractory schizophrenia. Department of Veterans Affairs Cooperative Study Group on Clozapine in Refractory Schizophrenia. Am J Psychiatry. 1999;156(1):88-93. doi: 10.1176/ajp.156.1.88.	**Included**
258	Filbey FM, Holcomb J, Nair TR, Christensen JD, Garver DL. Negative symptoms of familial schizophrenia breed true in unstable (vs. stable) cerebral-ventricle pedigrees. Schizophr Res. 1999;35(1):15-23. doi: 10.1016/s0920-9964(98)00107-8.	No drug
259	Slaghuis WL, Curran CE. Spatial frequency masking in positive- and negative-symptom schizophrenia. J Abnorm Psychol. 1999;108(1):42-50. doi: 10.1037//0021-843x.108.1.42.	No drug
260	Gupta S, Droney T, Kyser A, Keller P. Selegiline augmentation of antipsychotics for the treatment of negative symptoms in schizophrenia. Compr Psychiatry. 1999;40(2):148-50. doi: 10.1016/s0010-440x(99)90119-0.	Case
261	Kato M, Kajimura N, Okuma T, Sekimoto M, Watanabe T, Yamadera H, Takahashi K. Association between delta waves during sleep and negative symptoms in schizophrenia. Pharmaco-eeg studies by using structurally different hypnotics. Neuropsychobiology. 1999;39(3):165-72. doi: 10.1159/000026577.	No drug
262	Goff DC, Evins AE. Negative symptoms in schizophrenia: neurobiological models and treatment response. Harv Rev Psychiatry. 1998;6(2):59-77. doi: 10.3109/10673229809000313.	No drug
263	Häfner H, Löffler W, Maurer K, Hambrecht M, an der Heiden W. Depression, negative symptoms, social stagnation and social decline in the early course of schizophrenia. Acta Psychiatr Scand. 1999;100(2):105-18. doi: 10.1111/j.1600-0447.1999.tb10831.x.	No drug

264	Sharma RP, Singh V, Janicak PG, Javaid JI, Pandey GN. The prolactin response to fenfluramine in schizophrenia is associated with negative symptoms. Schizophr Res. 1999;39(1):85-9. doi: 10.1016/s0920-9964(99)00014-6.	No drug
265	Tsutsumi T, Uchimura H. Algorithm for the treatment of negative symptoms in chronic schizophrenia. Psychiatry Clin Neurosci. 1999;53(Suppl):S15-7.	Unfocused
266	Jungerman T, Rabinowitz D, Klein E. Deprenyl augmentation for treating negative symptoms of schizophrenia: a double-blind, controlled study. J Clin Psychopharmacol. 1999;19(6):522-5. doi: 10.1097/00004714-199912000-00006.	**Included**
267	Hale A, Azorin J-M, Kasper S, Maier W, Syvalahti E, Van Der Burght M, Sloth-Nielsen M, Wehnert A. Sertindole improves both the positive and negative symptoms of schizophrenia: Results of a phase III trial. Int J Psychiatry Clin Pract. 2000;4(1):55-62. doi: 10.1080/13651500050518406.	**Included**
268	Wighton A, Tweed JA, Butler A, Welch CP, Reynolds C, Bratty JR. The efficacy of zotepine in treating acute negative symptoms of schizophrenia: The results of a meta-analysis. Int J Psychiatry Clin Pract. 2000;4(3):209-14. doi: 10.1080/13651500050518091.	Review
269	Brébion G, Amador X, Smith MJ, Malaspina D, Sharif Z, Gorman JM. Opposite links of positive and negative symptomatology with memory errors in schizophrenia. Psychiatry Res. 1999;88(1):15-24. doi: 10.1016/s0165-1781(99)00076-1.	No drug
270	Craver JC, Pogue-Geile MF. Familial liability to schizophrenia: a sibling study of negative symptoms. Schizophr Bull. 1999;25(4):827-39. doi: 10.1093/oxfordjournals.schbul.a033422.	No drug
271	McDaniel WF, Heindel CS, Harris DW. Verbal memory and negative symptoms of schizophrenia revisited. Schizophr Res. 2000;41(3):473-5. doi: 10.1016/s0920-9964(99)00096-1.	No drug
272	Dyck DG, Short RA, Hendryx MS, Norell D, Myers M, Patterson T, McDonell MG, Voss WD, McFarlane WR. Management of negative symptoms among patients with schizophrenia attending multiple-family groups. Psychiatr Serv. 2000;51(4):513-9. doi: 10.1176/appi.ps.51.4.513.	No drug
273	Sanfilipo M, Lafargue T, Rusinek H, Arena L, Loneragan C, Lautin A, Feiner D, Rotrosen J, Wolkin A. Volumetric measure of the frontal and temporal lobe regions in schizophrenia: relationship to negative symptoms. Arch Gen Psychiatry. 2000;57(5):471-80. doi: 10.1001/archpsyc.57.5.471.	No drug
274	Kopelowicz A, Zarate R, Tripodis K, Gonzalez V, Mintz J. Differential efficacy of olanzapine for deficit and nondeficit negative symptoms in schizophrenia. Am J Psychiatry. 2000;157(6):987-93. doi: 10.1176/appi.ajp.157.6.987.	Open
275	Malaspina D, Goetz RR, Yale S, Berman A, Friedman JH, Tremeau F, Printz D, Amador X, Johnson J, Brown A, Gorman JM. Relation of familial schizophrenia to negative symptoms but not to the deficit syndrome. Am J Psychiatry. 2000;157(6):994-1003. doi: 10.1176/appi.ajp.157.6.994.	No drug
276	Möller HJ. Neue bzw.atypische Neuroleptika bei schizophrener Negativsymptomatik. Ergebnisse und methodische Probleme der Evaluation [New i.e. atypical neuroleptic agents for negative symptoms of schizophrenia: results and methodological problems of evaluation]. Nervenarzt. 2000;71(5):345-53. German. doi: 10.1007/s001150050568.	Review
277	Aliev NA. Исследование влияния пикротоксина на негативные симптомы шизофрении [The study of the influence of picrotoxin on the negative symptoms of schizophrenia]. Алиев Н.А. Исследование влияния пикротоксина на негативные симптомы шизофрении. Журнал неврологии и психиатрии 100 N 5-2000 59-60 Zh Nevrol Psikhiatr Im S S Korsakova. 2000;100(5):59-60. Russian.	Open
278	Brébion G, Amador X, Smith M, Malaspina D, Sharif Z, Gorman JM. Depression, psychomotor retardation, negative symptoms, and memory in schizophrenia. Neuropsychiatry Neuropsychol Behav Neurol. 2000;13(3):177-83.	No drug
279	Noda Y, Nabeshima T. [Neuropsychopharmacological study on an animal model for negative symptom of schizophrenia induced by repeated phencyclidine treatment]. Yakugaku Zasshi. 2000;120(8):677-82. Japanese.	Animal
280	Peralta V, Cuesta MJ, Martinez-Larrea A, Serrano JF. Differentiating primary from secondary negative symptoms in schizophrenia: a study of neuroleptic-naive patients before and after treatment. Am J Psychiatry. 2000;157(9):1461-6. doi: 10.1176/appi.ajp.157.9.1461.	No drug
281	Selten JP, Wiersma D, van den Bosch RJ. Distress attributed to negative symptoms in schizophrenia. Schizophr Bull. 2000;26(3):737-44. doi: 10.1093/oxfordjournals.schbul.a033490. PMID: 10993410.	No drug
282	McGurk SR, Moriarty PJ, Harvey PD, Parrella M, White L, Friedman J, Davis KL. Relationship of cognitive functioning, adaptive life skills, and negative symptom severity in poor-outcome geriatric schizophrenia patients. J Neuropsychiatry Clin Neurosci. 2000;12(2):257-64. doi: 10.1176/jnp.12.2.257.	No drug
283	Callicott JH, Bertolino A, Egan MF, Mattay VS, Langheim FJ, Weinberger DR. Selective relationship between prefrontal N-acetylaspartate measures and negative symptoms in schizophrenia. Am J Psychiatry. 2000;157(10):1646-51. doi: 10.1176/appi.ajp.157.10.1646.	No drug
284	Provencher HL, Fincham FD. Attributions of causality, responsibility and blame for positive and negative symptom behaviours in caregivers of persons with schizophrenia. Psychol Med. 2000;30(4):899-910. doi: 10.1017/s0033291799002342.	No drug
285	Tandon R, DeQuardo JR, Taylor SF, McGrath M, Jibson M, Eiser A, Goldman M. Phasic and enduring negative symptoms in schizophrenia: biological markers and relationship to outcome. Schizophr Res. 2000;45(3):191-201. doi: 10.1016/s0920-9964(99)00163-2. Erratum in: Schizophr Res 2001;51(2-3):185.	No drug
286	Purnine DM, Carey KB, Maisto SA, Carey MP. Assessing positive and negative symptoms in outpatients with schizophrenia and mood disorders. J Nerv Ment Dis. 2000;188(10):653-61. doi: 10.1097/00005053-200010000-00003.	No drug
287	Putnam KM, Harvey PD. Cognitive impairment and enduring negative symptoms: a comparative study of geriatric and nongeriatric schizophrenia patients. Schizophr Bull. 2000;26(4):867-78. doi: 10.1093/oxfordjournals.schbul.a033501.	No drug
288	Ellenbroek BA, Cools AR. Animal models for the negative symptoms of schizophrenia. Behav Pharmacol. 2000;11(3-4):223-33. doi: 10.1097/00008877-200006000-00006.	Review
289	Penadés R, Gastó C, Boget T, Catalán R, Salamero M. Deficit in schizophrenia: the relationship between negative symptoms and neurocognition. Compr Psychiatry. 2001;42(1):64-9. doi: 10.1053/comp.2001.19745.	No drug
290	Lindamer LA, Buse DC, Lohr JB, Jeste DV. Hormone replacement therapy in postmenopausal women with schizophrenia: positive effect on negative symptoms? Biol Psychiatry. 2001;49(1):47-51. doi: 10.1016/s0006-3223(00)00995-1.	Unfocused
291	Gilbert EA, Liberman RP, Ventura J, Kern R, Robertson MJ, Hwang S, Green MF. Concurrent validity of negative symptom assessments in treatment refractory schizophrenia: relationship between interview-based ratings and inpatient ward observations. J Psychiatr Res. 2000;34(6):443-7. doi: 10.1016/s0022-3956(00)00041-8.	No drug
292	Menon V, Anagnoson RT, Mathalon DH, Glover GH, Pfefferbaum A. Functional neuroanatomy of auditory working memory in schizophrenia: relation to positive and negative symptoms. Neuroimage. 2001;13(3):433-46. doi: 10.1006/nimg.2000.0699.	No drug
293	Tattan TM, Creed FH. Negative symptoms of schizophrenia and compliance with medication. Schizophr Bull. 2001;27(1):149-55. doi: 10.1093/oxfordjournals.schbul.a006853.	No drug
294	Berk M, Ichim C, Brook S. Efficacy of mirtazapine add on therapy to haloperidol in the treatment of the negative symptoms of schizophrenia: a double-blind randomized placebo-controlled study. Int Clin Psychopharmacol. 2001;16(2):87-92. doi: 10.1097/00004850-200103000-00003.	**Included**
295	Emsley RA, Niehaus DJ, Mbanga NI, Oosthuizen PP, Stein DJ, Maritz JS, Pimstone SN, Hayden MR, Laurent C, Deleuze JF, Mallet J. The factor structure for positive and negative symptoms in South African Xhosa patients with schizophrenia. Schizophr Res. 2001;47(2-3):149-57. doi: 10.1016/s0920-9964(00)00010-4.	No drug
296	Fleischhacker W. Negative symptoms in patients with schizophrenia with special reference to the primary versus secondary distinction. Encéphale. 2000;26 Spec No 1:12-4. English, French.	Opinion
297	Slaghuis WL, Bishop AM. Luminance flicker sensitivity in positive- and negative-symptom schizophrenia. Exp Brain Res. 2001;138(1):88-99. doi: 10.1007/s002210100683.	No drug

298	Fanous A, Gardner C, Walsh D, Kendler KS. Relationship between positive and negative symptoms of schizophrenia and schizotypal symptoms in nonpsychotic relatives. Arch Gen Psychiatry. 2001;58(7):669-73. doi: 10.1001/archpsyc.58.7.669.	No drug
299	Pantelis C, Stuart GW, Nelson HE, Robbins TW, Barnes TR. Spatial working memory deficits in schizophrenia: relationship with tardive dyskinesia and negative symptoms. Am J Psychiatry. 2001;158(8):1276-85. doi: 10.1176/appi.ajp.158.8.1276.	No drug
300	Jockers-Scherübl M, Godemann F, Pietzcker A. Negative symptoms of schizophrenia are improved by paroxetine added to neuroleptics: a pilot study. J Clin Psychiatry. 2001;62(7):573. doi: 10.4088/jcp.v62n07c13.	Open
301	Javitt DC. Management of negative symptoms of schizophrenia. Curr Psychiatry Rep. 2001;3(5):413-7. doi: 10.1007/s11920-996-0036-9.	Review
302	Troisi A, Pasini A, Spalletta G. Season of birth, gender and negative symptoms in schizophrenia. Eur Psychiatry. 2001;16(6):342-8. doi: 10.1016/s0924-9338(01)00589-2.	No drug
303	Herbener ES, Harrow M. Longitudinal assessment of negative symptoms in schizophrenia/schizoaffective patients, other psychotic patients, and depressed patients. Schizophr Bull. 2001;27(3):527-37. doi: 10.1093/oxfordjournals.schbul.a006893.	No drug
304	Lahti AC, Holcomb HH, Medoff DR, Weiler MA, Tamminga CA, Carpenter WT Jr. Abnormal patterns of regional cerebral blood flow in schizophrenia with primary negative symptoms during an effortful auditory recognition task. Am J Psychiatry. 2001;158(11):1797-808. doi: 10.1176/appi.ajp.158.11.1797.	No drug
305	Noel-Jorand MC, Giudicelli S, Dassa D, Reinert M. Discourse characteristics of subjects with schizophrenia and prominent negative symptoms Can J Psychiatry. 2001;46(8):761-2. doi: 10.1177/070674370104600816.	No drug
306	Silver H, Shlomo N. Perception of facial emotions in chronic schizophrenia does not correlate with negative symptoms but correlates with cognitive and motor dysfunction. Schizophr Res. 2001;52(3):265-73. doi: 10.1016/s0920-9964(00)00093-1.	No drug
307	Schuepbach D, Keshavan MS, Kmiec JA, Sweeney JA. Negative symptom resolution and improvements in specific cognitive deficits after acute treatment in first-episode schizophrenia. Schizophr Res. 2002;53(3):249-61. doi: 10.1016/s0920-9964(01)00195-5. PMID: 11738538.	No drug
308	Wible CG, Anderson J, Shenton ME, Kricun A, Hirayasu Y, Tanaka S, Levitt JJ, O'Donnell BF, Kikinis R, Jolesz FA, McCarley RW. Prefrontal cortex, negative symptoms, and schizophrenia: an MRI study. Psychiatry Res. 2001;108(2):65-78. doi: 10.1016/s0925-4927(01)00109-3.	No drug
309	Semkovska M, Bédard MA, Stip E. Hypofrontalité et symptômes négatifs dans la schizophrénie: synthèse des acquis anatomiques et neuropsychologiques et perspectives écologiques [Hypofrontality and negative symptoms in schizophrenia: synthesis of anatomic and neuropsychological knowledge and ecological perspectives]. Encéphale. 2001;27(5):405-15. French.	Review
310	Potkin SG, Alva G, Fleming K, Anand R, Keator D, Carreon D, Doo M, Jin Y, Wu JC, Fallon JH. A PET study of the pathophysiology of negative symptoms in schizophrenia. Positron emission tomography. Am J Psychiatry. 2002;159(2):227-37. doi: 10.1176/appi.ajp.159.2.227.	No drug
311	Möller H-J. Amisulpride: efficacy in the management of chronic patients with predominant negative symptoms of schizophrenia. Eur Arch Psychiatry Clin Neurosci. 2001;251(5):217-24. doi: 10.1007/s004060170030.	Review
312	Pani L, Gessa GL. The substituted benzamides and their clinical potential on dysthymia and on the negative symptoms of schizophrenia. Mol Psychiatry. 2002;7(3):247-53. doi: 10.1038/sj.mp.4001040.	Review
313	Evins AE, Amico E, Posever TA, Toker R, Goff DC. D-Cycloserine added to risperidone in patients with primary negative symptoms of schizophrenia. Schizophr Res. 2002;56(1-2):19-23. doi: 10.1016/s0920-9964(01)00220-1.	Open
314	Gaertner I, Gaertner HJ, Vonthein R, Dietz K. Prospective 6-year trial with clozapine: negative symptoms in outpatients with schizophrenia improve despite intermittent positive symptoms. J Clin Psychopharmacol. 2002;22(4):437-8. doi: 10.1097/00004714-200208000-00019.	Open
315	Müller MJ, Wetzel H, Benkert O. Differential effects of high-dose amisulpride versus flupentixol on latent dimensions of depressive and negative symptomatology in acute schizophrenia: an evaluation using confirmatory factor analysis. Int Clin Psychopharmacol. 2002;17(5):249-61. doi: 10.1097/00004850-200209000-00005.	Unfocused
316	Patkar AA, Gopalakrishnan R, Lundy A, Leone FT, Certa KM, Weinstein SP. Relationship between tobacco smoking and positive and negative symptoms in schizophrenia. J Nerv Ment Dis. 2002;190(9):604-10. doi: 10.1097/00005053-200209000-00005.	No drug
317	Shirayama Y, Hashimoto K, Suzuki Y, Higuchi T. Correlation of plasma neurosteroid levels to the severity of negative symptoms in male patients with schizophrenia. Schizophr Res. 2002;58(1):69-74. doi: 10.1016/s0920-9964(01)00367-x.	No drug
318	Brébion G, Gorman JM, Amador X, Malaspina D, Sharif Z. Source monitoring impairments in schizophrenia: characterisation and associations with positive and negative symptomatology. Psychiatry Res. 2002;112(1):27-39. doi: 10.1016/s0165-1781(02)00187-7.	No drug
319	Anderson JE, Wible CG, McCarley RW, Jakab M, Kasai K, Shenton ME. An MRI study of temporal lobe abnormalities and negative symptoms in chronic schizophrenia. Schizophr Res. 2002;58(2-3):123-34. doi: 10.1016/s0920-9964(01)00372-3.	No drug
320	Oosthuizen P, Emsley RA, Roberts MC, Turner J, Keyter L, Keyter N, Torreman M. Depressive symptoms at baseline predict fewer negative symptoms at follow-up in patients with first-episode schizophrenia. Schizophr Res. 2002;58(2-3):247-52. doi: 10.1016/s0920-9964(01)00375-9. PMID: 12409165.	No drug
321	Rosse RB, Deutsch SI. Adjuvant galantamine administration improves negative symptoms in a patient with treatment-refractory schizophrenia. Clin Neuropharmacol. 2002;25(5):272-5. doi: 10.1097/00002826-200209000-00010.	Case
322	Salokangas RK, Honkonen T, Stengård E, Koivisto AM, Hietala J. Negative symptoms and neuroleptics in catatonic schizophrenia. Schizophr Res. 2003;59(1):73-6. doi: 10.1016/s0920-9964(02)00155-x.	Unfocused
323	Lysaker PH, Bryson GJ, Marks KA, Greig TC, Bell MD. Association of obsessions and compulsions in schizophrenia with neurocognition and negative symptoms. J Neuropsychiatry Clin Neurosci. 2002;14(4):449-53. doi: 10.1176/jnp.14.4.449.	No drug
324	Bottlender R, Sato T, Jäger M, Kunze I, Groll C, Borski I, Möller HJ. Does considering duration of negative symptoms increase their specificity for schizophrenia? Schizophr Res. 2003;60(2-3):321-2. doi: 10.1016/s0920-9964(02)00317-1.	No drug
325	Weiner I. The "two-headed" latent inhibition model of schizophrenia: modeling positive and negative symptoms and their treatment. Psychopharmacology (Berl). 2003;169(3-4):257-97. doi: 10.1007/s00213-002-1313-x. Epub 2003 Feb 25.	Review
326	Wolkin A, Choi SJ, Szilagyi S, Sanfilipo M, Rotrosen JP, Lim KO. Inferior frontal white matter anisotropy and negative symptoms of schizophrenia: a diffusion tensor imaging study. Am J Psychiatry. 2003;160(3):572-4. doi: 10.1176/appi.ajp.160.3.572.	No drug
327	Rao ML, Kölsch H. Effects of estrogen on brain development and neuroprotection--implications for negative symptoms in schizophrenia. Psychoneuroendocrinology. 2003;28(Suppl 2):83-96. doi: 10.1016/s0306-4530(02)00126-9.	Review
328	Slaghuis WL, Thompson AK. The effect of peripheral visual motion on focal contrast sensitivity in positive- and negative-symptom schizophrenia. Neuropsychologia. 2003;41(8):968-80. doi: 10.1016/s0028-3932(02)00321-4.	No drug
329	Storosum JG, Elferink AJ, van Zwieten BJ, van Strik R, Hoogendijk WJ, Broekmans AW. Amisulpride: is there a treatment for negative symptoms in schizophrenia patients? Schizophr Bull. 2002;28(2):193-201. doi: 10.1093/oxfordjournals.schbul.a006931.	Review
330	Evren EC, Evren B, Erkiran M. Sizofrenili hastalarda ozkiyim düşüncesi: pozitif, negatif belirtiler, depresyon ve içgörüyle ilişkisi [Suicidal ideation in schizophrenia: relationship with positive and negative symptoms, depression and insight]. Turk Psikiyatri Derg. 2002;13(4):255-64. Turkish.	No drug
331	Skurkovich SV, Aleksandrovsky YA, Chekhonin VP, Ryabukhin IA, Chakhava KO, Skurkovich B. Improvement in negative symptoms of schizophrenia with antibodies to tumor necrosis factor-alpha and to interferon-gamma: a case report. J Clin Psychiatry. 2003;64(6):734-5. doi: 10.4088/jcp.v64n0618e.	Case
332	Oshima I, Mino Y, Inomata Y. Institutionalisation and schizophrenia in Japan: social environments and negative symptoms: Nationwide survey of in- patients. Br J Psychiatry. 2003;183:50-6. doi: 10.1192/bjp.183.1.50.	No drug
333	Takaoka K, Yoshida M. [Two syndromes in schizophrenia; positive and negative symptoms]. Ryoikibetsu Shokogun Shirizu. 2003;(38):57-9. Japanese.	Review

334	Möller HJ. Management of the negative symptoms of schizophrenia: new treatment options. CNS Drugs. 2003;17(11):793-823. doi: 10.2165/00023210-200317110-00003.	Review
335	Suslow T, Roestel C, Ohrmann P, Arolt V. The experience of basic emotions in schizophrenia with and without affective negative symptoms. Compr Psychiatry. 2003;44(4):303-10. doi: 10.1016/S0010-440X(03)00085-3.	No drug
336	Donohoe G, Robertson IH. Can specific deficits in executive functioning explain the negative symptoms of schizophrenia? A review. Neurocase. 2003;9(2):97-108. doi: 10.1076/neur.9.2.97.15075.	Review
337	Yasui-Furukori N, Kondo T, Mihara K, Inoue Y, Kaneko S. Fluvoxamine dose-dependent interaction with haloperidol and the effects on negative symptoms in schizophrenia. Psychopharmacology (Berl). 2004;171(2):223-7. doi: 10.1007/s00213-003-1567-y. Epub 2003 Sep 4.	Open
338	Philipp M, Lesch OM, Schmauss M, Dose M, Glaser T. Vergleichbare Wirksamkeit von Flupentixol und Risperidon auf schizophrene Negativsymptomatik [Comparative effectiveness of flupenthixol and risperidone on negative symptoms of schizophrenia]. Psychiatr Prax. 2003;30(Suppl 2):S94-6. German.	Overlap=452
339	Ellingrod VL, Lund BC, Miller D, Fleming F, Perry P, Holman TL, Bever- Stille K. 5-HT2A receptor promoter polymorphism, -1438G/A and negative symptom response to olanzapine in schizophrenia. Psychopharmacol Bull. 2003;37(2):109-12.	Unfocused
340	Silver H. Selective serotonin reuptake inhibitor augmentation in the treatment of negative symptoms of schizophrenia. Int Clin Psychopharmacol. 2003;18(6):305-13. doi: 10.1097/00004850-200311000-00001.	Review
341	Wang CS, Yang YK, Chen M, Chiu NT, Yeh TL, Lee IH. Negative symptoms and regional cerebral blood flow in patients with schizophrenia: a single photon emission computed tomography study. Kaohsiung J Med Sci. 2003;19(9):464-9. doi: 10.1016/S1607-551X(09)70492-9.	No drug
342	Sperling W, Kornhuber J, Bleich S. Dipole elevations over the temporoparietal brain area are associated with negative symptoms in schizophrenia. Schizophr Res. 2003;64(2-3):187-8. doi: 10.1016/s0920-9964(03)00005-7.	No drug
343	Feldman PD, Kaiser CJ, Kennedy JS, Sutton VK, Tran PV, Tollefson GD, Zhang F, Breier A. Comparison of risperidone and olanzapine in the control of negative symptoms of chronic schizophrenia and related psychotic disorders in patients aged 50 to 65 years. J Clin Psychiatry. 2003;64(9):998-1004. doi: 10.4088/jcp.v64n0904.	**Included**
344	Suslow T, Roestel C, Arolt V. Affective priming in schizophrenia with and without affective negative symptoms. Eur Arch Psychiatry Clin Neurosci. 2003;253(6):292-300. doi: 10.1007/s00406-003-0443-4.	No drug
345	Sumiyoshi T, Anil AE, Jin D, Jayathilake K, Lee M, Meltzer HY. Plasma glycine and serine levels in schizophrenia compared to normal controls and major depression: relation to negative symptoms. Int J Neuropsychopharmacol. 2004;7(1):1-8. doi: 10.1017/S1461145703003900. Epub 2004 Jan 13.	No drug
346	Arango C, Buchanan RW, Kirkpatrick B, Carpenter WT. The deficit syndrome in schizophrenia: implications for the treatment of negative symptoms. Eur Psychiatry. 2004;19(1):21-6. doi: 10.1016/j.eurpsy.2003.10.004.	Opinion
347	Rothermundt M, Ponath G, Glaser T, Hetzel G, Arolt V. S100B serum levels and long-term improvement of negative symptoms in patients with schizophrenia. Neuropsychopharmacology. 2004;29(5):1004-11. doi: 10.1038/sj.npp.1300403.	No drug
348	Lencz T, Smith CW, Auther A, Correll CU, Cornblatt B. Nonspecific and attenuated negative symptoms in patients at clinical high-risk for schizophrenia. Schizophr Res. 2004;68(1):37-48. doi: 10.1016/S0920-9964(03)00214-7.	No drug
349	Galeno R, Molina M, Guirao M, Isoardi R. Severity of negative symptoms in schizophrenia correlated to hyperactivity of the left globus pallidus and the right claustrum. A PET study. World J Biol Psychiatry. 2004;5(1):20-5. doi: 10.1080/15622970410029903.	No drug
350	Heresco-Levy U, Javitt DC. Comparative effects of glycine and D-cycloserine on persistent negative symptoms in schizophrenia: a retrospective analysis. Schizophr Res. 2004;66(2-3):89-96. doi: 10.1016/S0920-9964(03)00129-4.	Unfocused
351	Zoccali R, Muscatello MR, Cedro C, Neri P, La Torre D, Spina E, Di Rosa AE, Meduri M. The effect of mirtazapine augmentation of clozapine in the treatment of negative symptoms of schizophrenia: a double-blind, placebo-controlled study. Int Clin Psychopharmacol. 2004;19(2):71-6. doi: 10.1097/00004850-200403000-00003.	**Included**
352	Sheitman BB, Kraus JE, Bodfish JW, Carmel H. Are the negative symptoms of schizophrenia consistent with an autistic spectrum illness? Schizophr Res. 2004;69(1):119-20. doi: 10.1016/S0920-9964(03)00177-4.	No drug
353	Möller H-J. Non-neuroleptic approaches to treating negative symptoms in schizophrenia. Eur Arch Psychiatry Clin Neurosci. 2004;254(2):108-16. doi: 10.1007/s00406-004-0503-4.	Review
354	Rueter LE, Ballard ME, Gallagher KB, Basso AM, Curzon P, Kohlhaas KL. Chronic low dose risperidone and clozapine alleviate positive but not negative symptoms in the rat neonatal ventral hippocampal lesion model of schizophrenia. Psychopharmacology (Berl). 2004;176(3-4):312-9. doi: 10.1007/s00213-004-1897-4. Epub 2004 Jun 4.	Animal
355	Coyle JT, Tsai G. The NMDA receptor glycine modulatory site: a therapeutic target for improving cognition and reducing negative symptoms in schizophrenia. Psychopharmacology (Berl). 2004;174(1):32-8. doi: 10.1007/s00213-003-1709-2. Epub 2003 Nov 25.	Opinion
356	Soreni N, Weizman A, Weiss M. Beneficial effects of gonadotropin-releasing hormone analogue treatment on positive and negative symptoms of schizophrenia: a case report. J Clin Psychiatry. 2004;65(7):1020-1. doi: 10.4088/jcp.v65n0720f.	Case
357	Goff DC, Bottiglieri T, Arning E, Shih V, Freudenreich O, Evins AE, Henderson DC, Baer L, Coyle J. Folate, homocysteine, and negative symptoms in schizophrenia. Am J Psychiatry. 2004;161(9):1705-8. doi: 10.1176/appi.ajp.161.9.1705.	No drug
358	Compton MT, Furman AC, Kaslow NJ. Lower negative symptom scores among cannabis-dependent patients with schizophrenia-spectrum disorders: preliminary evidence from an African American first-episode sample. Schizophr Res. 2004;71(1):61-4. doi: 10.1016/j.schres.2004.01.005.	No drug
359	Lenert LA, Sturley AP, Rapaport MH, Chavez S, Mohr PE, Rupnow M. Public preferences for health states with schizophrenia and a mapping function to estimate utilities from positive and negative symptom scale scores. Schizophr Res. 2004;71(1):155-65. doi: 10.1016/j.schres.2003.10.010. Erratum in: Schizophr Res. 2005;80(1):135-6.	No drug
360	Silver H. Selective serotonin re-uptake inhibitor augmentation in the treatment of negative symptoms of schizophrenia. Expert Opin Pharmacother. 2004;5(10):2053-8. doi: 10.1517/14656566.5.10.2053.	Review
361	Ventura J, Nuechterlein KH, Green MF, Horan WP, Subotnik KL, Mintz J. The timing of negative symptom exacerbations in relationship to positive symptom exacerbations in the early course of schizophrenia. Schizophr Res. 2004;69(2-3):333-42. doi: 10.1016/s0920-9964(03)00096-3.	No drug
362	Duncan EJ, Szilagyi S, Schwartz MP, Bugarski-Kirola D, Kunzova A, Negi S, Stephanides M, Efferen TR, Angrist B, Peselow E, Corwin J, Gonzenbach S, Rotrosen JP. Effects of D-cycloserine on negative symptoms in schizophrenia. Schizophr Res. 2004;71(2-3):239-48. doi: 10.1016/j.schres.2004.03.013.	**Included**
363	Tandon R. Quetiapine has a direct effect on the negative symptoms of schizophrenia. Hum Psychopharmacol. 2004;19(8):559-63. doi: 10.1002/hup.642.	Review
364	Martín Reyes M, Mendoza Quiñónez R, Díaz de Villalvilla T, Lomba P, Padrón Fernando A, Valdés Sosa M. Transmisión familiar de los síntomas positivos y negativos en la esquizofrenia familiar y esporádica [Family transmission of positive and negative symptoms in familial and sporadic schizophrenia]. Actas Esp Psiquiatr. 2004;32(6):353-7. Spanish.	No drug
365	Sams-Dodd F. (+) MK-801 and phencyclidine induced neurotoxicity do not cause enduring behaviours resembling the positive and negative symptoms of schizophrenia in the rat. Basic Clin Pharmacol Toxicol. 2004;95(5):241-6. doi: 10.1111/j.1742-7843.2004.pto950507.x.	Animal
366	Liu Z, Tam WC, Xue Z, Yao S, Wu D. Positive and negative symptom profile schizophrenia and abnormalities in the P300 component of the event-related potential: a longitudinal controlled study. Psychiatry Res. 2004;132(2):131-9. doi: 10.1016/j.pscychresns.2004.03.003.	No drug

367	Jockers-Scherübl MC, Bauer A, Godemann F, Reischies FM, Selig F, Schlattmann P. Negative symptoms of schizophrenia are improved by the addition of paroxetine to neuroleptics: a double-blind placebo-controlled study. Int Clin Psychopharmacol. 2005;20(1):27-31. doi: 10.1097/00004850-200501000-00006.	**Included**
368	Thoma RJ, Hanlon FM, Moses SN, Ricker D, Huang M, Edgar C, Irwin J, Torres F, Weisend MP, Adler LE, Miller GA, Canive JM. M50 sensory gating predicts negative symptoms in schizophrenia. Schizophr Res. 2005;73(2-3):311-8. doi: 10.1016/j.schres.2004.07.001.	No drug
369	Goyal RO, Sagar R, Ammini AC, Khurana ML, Alias AG. Negative correlation between negative symptoms of schizophrenia and testosterone levels. Ann N Y Acad Sci. 2004;1032:291-4. doi: 10.1196/annals.1314.042.	No drug
370	Bodkin JA, Siris SG, Bermanzohn PC, Hennen J, Cole JO. Double-blind, placebo-controlled, multicenter trial of selegiline augmentation of antipsychotic medication to treat negative symptoms in outpatients with schizophrenia. Am J Psychiatry. 2005;162(2):388-90. doi: 10.1176/appi.ajp.162.2.388.	**Included**
371	Reynolds GP, Yao Z, Zhang X, Sun J, Zhang Z. Pharmacogenetics of treatment in first-episode schizophrenia: D3 and 5-HT2C receptor polymorphisms separately associate with positive and negative symptom response. Eur Neuropsychopharmacol. 2005;15(2):143-51. doi: 10.1016/j.euroneuro.2004.07.001.	No drug
372	Brazo P, Delamillieure P, Morello R, Halbecq I, Marié RM, Dollfus S. Impairments of executive/attentional functions in schizophrenia with primary and secondary negative symptoms. Psychiatry Res. 2005;133(1):45-55. doi: 10.1016/j.psychres.2004.10.001.	No drug
373	Iancu I, Poreh A, Lehman B, Shamir E, Kotler M. The Positive and Negative Symptoms Questionnaire: a self-report scale in schizophrenia. Compr Psychiatry. 2005;46(1):61-6. doi: 10.1016/j.comppsych.2004.07.014.	No drug
374	Hamdani N, Bonnière M, Adès J, Hamon M, Boni C, Gorwood P. Negative symptoms of schizophrenia could explain discrepant data on the association between the 5-HT2A receptor gene and response to antipsychotics. Neurosci Lett. 2005;377(1):69-74. doi: 10.1016/j.neulet.2004.11.070. Epub 2004 Dec 22.	No drug
375	Milev P, Ho BC, Arndt S, Andreasen NC. Predictive values of neurocognition and negative symptoms on functional outcome in schizophrenia: a longitudinal first-episode study with 7-year follow-up. Am J Psychiatry. 2005;162(3):495-506. doi: 10.1176/appi.ajp.162.3.495.	No drug
376	Mubarak A, Badawy A. The effect of smoking on brainstem auditory evoked potentials in positive- and negative-symptom schizophrenia. CNS Spectr. 2001;6(6):514-6, 519-22. doi: 10.1017/s1092852900008063.	No drug
377	Lysaker PH, Wickett A, Davis LW. Narrative qualities in schizophrenia: associations with impairments in neurocognition and negative symptoms. J Nerv Ment Dis. 2005;193(4):244-9. doi: 10.1097/01.nmd.0000158376.53165.de.	No drug
378	Sumiyoshi T, Jin D, Jayathilake K, Lee M, Meltzer HY. Prediction of the ability of clozapine to treat negative symptoms from plasma glycine and serine levels in schizophrenia. Int J Neuropsychopharmacol. 2005;8(3):451-5. doi: 10.1017/S1461145705005237. Epub 2005 Apr 7.	Unfocused
379	Sekine Y, Takei N, Suzuki K, Nakamura K, Tsuchiya KJ, Takebayashi K, Toulopoulou T, Mori N. Effective adjunctive use of pergolide with quetiapine for cognitive impairment and negative symptoms in schizophrenia. J Clin Psychopharmacol. 2005;25(3):281-3. doi: 10.1097/01.jcp.0000162813.22689.d9.	Case
380	Rung JP, Carlsson A, Rydén Markinhuhta K, Carlsson ML. (+)-MK-801 induced social withdrawal in rats; a model for negative symptoms of schizophrenia. Prog Neuropsychopharmacol Biol Psychiatry. 2005;29(5):827-32. doi: 10.1016/j.pnpbp.2005.03.004.	Animal
381	Blanchard JJ, Horan WP, Collins LM. Examining the latent structure of negative symptoms: is there a distinct subtype of negative symptom schizophrenia? Schizophr Res. 2005;77(2-3):151-65. doi: 10.1016/j.schres.2005.03.022.	No drug
382	Kaneda Y, Ohmori T. Relation between estradiol and negative symptoms in men with schizophrenia. J Neuropsychiatry Clin Neurosci. 2005;17(2):239-42. doi: 10.1176/jnp.17.2.239.	No drug
383	Thoma P, Daum I. Neurokognitive Veränderungen und Negativsymptomatik bei schizophrenen Erkrankungen [Neurocognitive changes and negative symptoms in schizophrenia]. Fortschr Neurol Psychiatr. 2005;73(6):333-42. German. doi: 10.1055/s-2004-830233.	No drug
384	Herbener ES, Harrow M. Are negative symptoms associated with functioning deficits in both schizophrenia and nonschizophrenia patients? A 10-year longitudinal analysis. Schizophr Bull. 2004;30(4):813-25. doi: 10.1093/oxfordjournals.schbul.a007134.	No drug
385	Schuepbach D, Hill SK, Sanders RD, Hell D, Keshavan MS, Sweeney JA. Early treatment-induced improvement of negative symptoms predicts cognitive functioning in treatment-naive first episode schizophrenia: a 2-year followup. Schizophr Bull. 2004;30(4):837-48. doi: 10.1093/oxfordjournals.schbul.a007136.	No drug
386	Rector NA, Beck AT, Stolar N. The negative symptoms of schizophrenia: a cognitive perspective. Can J Psychiatry. 2005;50(5):247-57. doi: 10.1177/070674370505000503.	No drug
387	Niehaus DJ, Koen L, Laurent C, Muller J, Deleuze JF, Mallet J, Seller C, Jordaan E, Emsley R. Positive and negative symptoms in affected sib pairs with schizophrenia: implications for genetic studies in an African Xhosa sample. Schizophr Res. 2005;79(2-3):239-49. doi: 10.1016/j.schres.2005.04.026. Epub 2005 Jul 1.	No drug
388	Schell AM, Dawson ME, Rissling A, Ventura J, Subotnik KL, Gitlin MJ, Nuechterlein KH. Electrodermal predictors of functional outcome and negative symptoms in schizophrenia. Psychophysiology. 2005;42(4):483-92. doi: 10.1111/j.1469-8986.2005.00300.x.	No drug
389	Louchart-de la Chapelle S, Levillain D, Ménard JF, Van der Elst A, Allio G, Haouzir S, Dollfus S, Campion D, Thibaut F. P50 inhibitory gating deficit is correlated with the negative symptomatology of schizophrenia. Psychiatry Res. 2005;136(1):27-34. doi: 10.1016/j.psychres.2003.04.001.	No drug
390	Stip E, Trudeau LE. Glycine and D-serine improve the negative symptoms of schizophrenia. Evid Based Ment Health. 2005;8(3):82. doi: 10.1136/ebmh.8.3.82.	Opinion
391	Laughren T, Levin R. Food and Drug Administration perspective on negative symptoms in schizophrenia as a target for a drug treatment claim. Schizophr Bull. 2006;32(2):220-2. doi: 10.1093/schbul/sbi039. Epub 2005 Aug 3.	Opinion
392	Lysaker PH, Davis LW, Lightfoot J, Hunter N, Stasburger A. Association of neurocognition, anxiety, positive and negative symptoms with coping preference in schizophrenia spectrum disorders. Schizophr Res. 2005;80(2-3):163-71. doi: 10.1016/j.schres.2005.07.005. Epub 2005 Aug 24.	No drug
393	Oshima I, Mino Y, Inomata Y. Effects of environmental deprivation on negative symptoms of schizophrenia: a nationwide survey in Japan's psychiatric hospitals. Psychiatry Res. 2005;136(2-3):163-71. doi: 10.1016/j.psychres.2005.06.001.	No drug
394	Kéri S, Kiss I, Kelemen O, Benedek G, Janka Z. Anomalous visual experiences, negative symptoms, perceptual organization and the magnocellular pathway in schizophrenia: a shared construct? Psychol Med. 2005;35(10):1445-55. doi: 10.1017/S0033291705005398.	No drug
395	Rocca P, Bellino S, Calvarese P, Marchiaro L, Patria L, Rasetti R, Bogetto F. Depressive and negative symptoms in schizophrenia: different effects on clinical features. Compr Psychiatry. 2005;46(4):304-10. doi: 10.1016/j.comppsych.2004.09.001.	No drug
396	Rummel C, Kissling W, Leucht S. Antidepressants as add-on treatment to antipsychotics for people with schizophrenia and pronounced negative symptoms: a systematic review of randomized trials. Schizophr Res. 2005;80(1):85-97. doi: 10.1016/j.schres.2005.07.035. Epub 2005 Sep 22.	Review
397	Brébion G, David AS, Jones H, Pilowsky LS. Hallucinations, negative symptoms, and response bias in a verbal recognition task in schizophrenia. Neuropsychology. 2005;19(5):612-617. doi: 10.1037/0894-4105.19.5.612.	No drug
398	Todarello O, Porcelli P, Grilletti F, Bellomo A. Is alexithymia related to negative symptoms of schizophrenia? A preliminary longitudinal study. Psychopathology. 2005;38(6):310-4. doi: 10.1159/000088919. Epub 2005 Oct 12.	No drug
399	Gama CS, Antunes P, Moser C, Belmonte-de-Abreu PS. A memantina como terapia adjuvante para os sintomas negativos da esquizofrenia [Memantine as an adjunctive therapy for schizophrenia negative symptoms]. Braz J Psychiatry. 2005;27(3):257-8. Portuguese. doi: 10.1590/s1516-44462005000300023. Epub 2005 Oct 4.	Case

400	Blanchard JJ, Cohen AS. The structure of negative symptoms within schizophrenia: implications for assessment. Schizophr Bull. 2006;32(2):238-45. doi: 10.1093/schbul/sbj013. Epub 2005 Oct 27. PMID: 16254064; PMCID: PMC2632211.	No drug
401	Jin Y, Potkin SG, Kemp AS, Huerta ST, Alva G, Thai TM, Carreon D, Bunney WE Jr. Therapeutic effects of individualized alpha frequency transcranial magnetic stimulation (alphaTMS) on the negative symptoms of schizophrenia. Schizophr Bull. 2006;32(3):556-61. doi: 10.1093/schbul/sbj020. Epub 2005 Oct 27. PMID: 16254067; PMCID: PMC2632240.	No drug
402	Riedel M, Müller N, Strassnig M, Spellmann I, Engel RR, Musil R, Dehning S, Douhet A, Schwarz MJ, Möller HJ. Quetiapine has equivalent efficacy and superior tolerability to risperidone in the treatment of schizophrenia with predominantly negative symptoms. Eur Arch Psychiatry Clin Neurosci. 2005;255(6):432-7. doi: 10.1007/s00406-005-0622-6. Epub 2005 Nov 4. PMID: 16267634.	**Included**
403	Bell MD, Mishara AL. Does negative symptom change relate to neurocognitive change in schizophrenia? Implications for targeted treatments. Schizophr Res. 2006;81(1):17-27. doi: 10.1016/j.schres.2005.09.016. Epub 2005 Nov 16. PMID: 16297601.	No drug
404	Erhart SM, Marder SR, Carpenter WT. Treatment of schizophrenia negative symptoms: future prospects. Schizophr Bull. 2006;32(2):234-7. doi: 10.1093/schbul/sbj055. Epub 2006 Feb 21. PMID: 16492797; PMCID: PMC2632230.	Opinion
405	Kirkpatrick B, Fischer B. Subdomains within the negative symptoms of schizophrenia: commentary. Schizophr Bull. 2006;32(2):246-9. doi: 10.1093/schbul/sbj054. Epub 2006 Feb 21. PMID: 16492798; PMCID: PMC2632226.	Opinion
406	Tarrier N. Negative symptoms in schizophrenia: comments from a clinical psychology perspective. Schizophr Bull. 2006;32(2):231-3. doi: 10.1093/schbul/sbj051. Epub 2006 Feb 21. PMID: 16492799; PMCID: PMC2632217.	Opinion
407	DeRosse P, Funke B, Burdick KE, Lencz T, Ekholm JM, Kane JM, Kucherlapati R, Malhotra AK. Dysbindin genotype and negative symptoms in schizophrenia. Am J Psychiatry. 2006;163(3):532-4. doi: 10.1176/appi.ajp.163.3.532. PMID: 16513878.	No drug
408	Lysaker PH, Whitney KA, Davis LW. Obsessive-compulsive and negative symptoms in schizophrenia: associations with coping preference and hope. Psychiatry Res. 2006;141(3):253-9. doi: 10.1016/j.psychres.2005.09.016. Epub 2006 Mar 6. PMID: 16519948.	No drug
409	Olié JP, Spina E, Murray S, Yang R. Ziprasidone and amisulpride effectively treat negative symptoms of schizophrenia: results of a 12-week, double-blind study. Int Clin Psychopharmacol. 2006;21(3):143-51. doi: 10.1097/01.yic.0000182121.59296.70. PMID: 16528136.	**Included**
410	Potvin S, Sepehry AA, Stip E. A meta-analysis of negative symptoms in dual diagnosis schizophrenia. Psychol Med. 2006;36(4):431-40. doi: 10.1017/S003329170500574X. PMID: 16536885.	No drug
411	Donohoe G, Corvin A, Robertson IH. Evidence that specific executive functions predict symptom variance among schizophrenia patients with a predominantly negative symptom profile. Cogn Neuropsychiatry. 2006;11(1):13-32. doi: 10.1080/13546800444000155. PMID: 16537231.	No drug
412	Akhondzadeh S, Rezaei F, Larijani B, Nejatisafa AA, Kashani L, Abbasi SH. Correlation between testosterone, gonadotropins and prolactin and severity of negative symptoms in male patients with chronic schizophrenia. Schizophr Res. 2006;84(2-3):405-10. doi: 10.1016/j.schres.2006.02.008. Epub 2006 Mar 20. PMID: 16545544.	No drug
413	Suslow T, Droste T, Roestel C, Arolt V. Automatic processing of facial emotion in schizophrenia with and without affective negative symptoms. Cogn Neuropsychiatry. 2005;10(1):35-56. doi: 10.1080/13546800344000318. PMID: 16571450.	No drug
414	Röhricht F, Priebe S. Effect of body-oriented psychological therapy on negative symptoms in schizophrenia: a randomized controlled trial. Psychol Med. 2006 May;36(5):669-78. doi: 10.1017/S0033291706007161. PMID: 16608559.	No drug
415	Novák T, Horácek J, Mohr P, Kopecek M, Skrdlantová L, Klirova M, Rodriguez M, Spaniel F, Dockery C, Höschl C. The double-blind sham-controlled study of high-frequency rTMS (20 Hz) for negative symptoms in schizophrenia: negative results. Neuro Endocrinol Lett. 2006;27(1-2):209-13. PMID: 16648775.	No drug
416	Álvarez E, Ciudad A, Olivares JM, Bousoño M, Gómez JC. A randomized, 1-year follow-up study of olanzapine and risperidone in the treatment of negative symptoms in outpatients with schizophrenia. J Clin Psychopharmacol. 2006;26(3):238-49. doi: 10.1097/01.jcp.0000222513.63767.de. PMID: 16702888.	**Included**
417	Talamo A, Centorrino F, Tondo L, Dimitri A, Hennen J, Baldessarini RJ. Comorbid substance-use in schizophrenia: relation to positive and negative symptoms. Schizophr Res. 2006;86(1-3):251-5. doi: 10.1016/j.schres.2006.04.004. Epub 2006 Jun 5. PMID: 16750347.	No drug
418	Ko YH, Joe SH, Cho W, Park JH, Lee JJ, Jung IK, Kim L, Kim SH. Estrogen, cognitive function and negative symptoms in female schizophrenia. Neuropsychobiology. 2006;53(4):169-75. doi: 10.1159/000093780. Epub 2006 Jun 6. PMID: 16763376.	No drug
419	Sirota P, Pannet I, Koren A, Tchernichovsky E. Quetiapine versus olanzapine for the treatment of negative symptoms in patients with schizophrenia. Hum Psychopharmacol. 2006;21(4):227-34. doi: 10.1002/hup.763. PMID: 16783811.	**Included**
420	Ciudad A, Olivares JM, Bousoño M, Gómez JC, Alvarez E. Improvement in social functioning in outpatients with schizophrenia with prominent negative symptoms treated with olanzapine or risperidone in a 1 year randomized, open- label trial. Prog Neuropsychopharmacol Biol Psychiatry. 2006;30(8):1515-22. doi: 10.1016/j.pnpbp.2006.05.010. Epub 2006 Jul 3. PMID: 16820255.	Unfocused
421	REMOVED: Tan YL, Zhou DF, Cao LY, Zou YZ, Wu GY, Yang Zhang X. Association between the BDNF C270T polymorphism and negative symptoms of schizophrenia. Schizophr Res. 2006 Jul 17. doi: 10.1016/j.schres.2005.01.022. Epub ahead of print. PMID: 16846718.	Retracted/ Removed
422	Rummel C, Kissling W, Leucht S. Antidepressants for the negative symptoms of schizophrenia. Cochrane Database Syst Rev. 2006;2006(3):CD005581. doi: 10.1002/14651858.CD005581.pub2. PMID: 16856105; PMCID: PMC9804000.	Review
423	Villalta-Gil V, Vilaplana M, Ochoa S, Haro JM, Dolz M, Usall J, Cervilla J; NEDENA Group. Neurocognitive performance and negative symptoms: are they equal in explaining disability in schizophrenia outpatients? Schizophr Res. 2006;87(1-3):246-53. doi: 10.1016/j.schres.2006.06.013. Epub 2006 Jul 21. PMID: 16859898.	No drug
424	The negative symptoms of schizophrenia. They are pervasive but sometimes invisible--and especially difficult to treat. Harv Ment Health Lett. 2006;23(1):1-3. PMID: 16862704.	Opinion
425	Jordaan E, Niehaus DJ, Koen L, Seller C, Mbanga I, Emsley RA. Season of birth, age and negative symptoms in a Xhosa schizophrenia sample from the Southern Hemisphere. Aust N Z J Psychiatry. 2006;40(8):698-703. doi: 10.1080/j.1440-1614.2006.01870.x. PMID: 16866766.	No drug
426	Murphy BP, Chung YC, Park TW, McGorry PD. Pharmacological treatment of primary negative symptoms in schizophrenia: a systematic review. Schizophr Res. 2006;88(1-3):5-25. doi: 10.1016/j.schres.2006.07.002. Epub 2006 Aug 23. PMID: 16930948.	Review
427	Granholm E, Verney SP, Perivoliotis D, Miura T. Effortful cognitive resource allocation and negative symptom severity in chronic schizophrenia. Schizophr Bull. 2007;33(3):831-42. doi: 10.1093/schbul/sbl040. Epub 2006 Sep 6. PMID: 16956985; PMCID: PMC2526135.	No drug
428	Kinon BJ, Noordsy DL, Liu-Seifert H, Gulliver AH, Ascher-Svanum H, Kollack-Walker S. Randomized, double-blind 6-month comparison of olanzapine and quetiapine in patients with schizophrenia or schizoaffective disorder with prominent negative symptoms and poor functioning. J Clin Psychopharmacol. 2006;26(5):453-61. doi: 10.1097/01.jcp.0000236658.16286.25. Erratum in: J Clin Psychopharmacol. 2009;29(2):169. PMID: 16974184.	**Included**
429	Brébion G, David AS, Jones HM, Ohlsen R, Pilowsky LS. Temporal context discrimination in patients with schizophrenia: associations with auditory hallucinations and negative symptoms. Neuropsychologia. 2007;45(4):817-23. doi: 10.1016/j.neuropsychologia.2006.08.009. Epub 2006 Sep 22. PMID: 16996090.	No drug
430	Aziz MA, Pepler A, McNeely C, Gorman JM. Remission of positive and negative symptoms in refractory schizophrenia with a combination of haloperidol and quetiapine: Two case studies. J Psychiatr Pract. 2006;12(5):332-6. doi: 10.1097/00131746-200609000-00011. PMID: 16998424.	Case
431	Stahl SM. Positive findings for negative symptoms of schizophrenia: no longer untreatable? Acta Psychiatr Scand. 2006 Nov;114(5):301-2. doi 10.1111/j.1600-0447.2006.00904.x. PMID: 17022789.	Review
432	Lecrubier Y, Quintin P, Bouhassira M, Perrin E, Lancrenon S. The treatment of negative symptoms and deficit states of chronic schizophrenia: olanzapine compared to amisulpride and placebo in a 6-month double-blind controlled clinical trial. Acta Psychiatr Scand. 2006;114(5):319-27. doi: 10.1111/j.1600-0447.2006.00887.x. PMID: 17022791.	**Included**

433	Winograd-Gurvich C, Fitzgerald PB, Georgiou-Karistianis N, Bradshaw JL, White OB. Negative symptoms: A review of schizophrenia, melancholic depression and Parkinson's disease. Brain Res Bull. 2006;70(4-6):312-21. doi: 10.1016/j.brainresbull.2006.06.007. Epub 2006 Jul 5. PMID: 17027767.	Review
434	Potvin S, Sepehry AA, Stip E. Comorbid substance-use in schizophrenia: the file drawer effect, In reply to: Talamo et al., 2006. "Comorbid substance-use in schizophrenia: relation to positive and negative symptoms". Schizophr Res. 2007;90(1-3):351-2; author reply 353-4. doi: 10.1016/j.schres.2006.09.006. Epub 2006 Oct 20. PMID: 17055705.	Opinion
435	Buchanan RW. Persistent negative symptoms in schizophrenia: an overview. Schizophr Bull. 2007;33(4):1013-22. doi: 10.1093/schbul/sbl057. Epub 2006 Nov 10. PMID: 17099070; PMCID: PMC2632326.	Review
436	Tejedor-Real P, Sahagún M, Biguet NF, Mallet J. Neonatal handling prevents the effects of phencyclidine in an animal model of negative symptoms of schizophrenia. Biol Psychiatry. 2007;61(7):865-72. doi: 10.1016/j.biopsych.2006.08.033. Epub 2006 Nov 27. PMID: 17125743.	Animal
437	Sergi MJ, Rassovsky Y, Widmark C, Reist C, Erhart S, Braff DL, Marder SR, Green MF. Social cognition in schizophrenia: relationships with neurocognition and negative symptoms. Schizophr Res. 2007;90(1-3):316-24. doi: 10.1016/j.schres.2006.09.028. Epub 2006 Dec 1. PMID: 17141477.	No drug
438	Zhao J, He X, Liu Z, Yang D. The effects of clozapine on cognitive function and regional cerebral blood flow in the negative symptom profile schizophrenia. Int J Psychiatry Med. 2006;36(2):171-81. doi: 10.2190/1AA0-UW9Q-1CNK-3E2N. PMID: 17154147.	Open
439	Powell AJ, Hansen LK. Gilbert's syndrome in a patient with predominantly negative symptoms of schizophrenia. Int J Psychiatry Clin Pract. 2007;11(3):239-41. doi: 10.1080/13651500600811735. PMID: 24941364.	Case
440	Stahl SM, Buckley PF. Negative symptoms of schizophrenia: a problem that will not go away. Acta Psychiatr Scand. 2007;115(1):4-11. doi: 10.1111/j.1600-0447.2006.00947.x. PMID: 17201860.	Review
441	Mancevski B, Keilp J, Kurzon M, Berman RM, Ortakov V, Harkavy-Friedman J, Rosoklija G, Dwork AJ. Lifelong course of positive and negative symptoms in chronically institutionalized patients with schizophrenia. Psychopathology. 2007;40(2):83-92. doi: 10.1159/000098488. Epub 2007 Jan 11. PMID: 17215594.	No drug
442	Lacerda AL, Hardan AY, Yorbik O, Vemulapalli M, Prasad KM, Keshavan MS. Morphology of the orbitofrontal cortex in first-episode schizophrenia: relationship with negative symptomatology. Prog Neuropsychopharmacol Biol Psychiatry. 2007;31(2):510-6. doi: 10.1016/j.pnpbp.2006.11.022. Epub 2007 Jan 18. PMID: 17239513.	No drug
443	Buckley PF, Stahl SM. Pharmacological treatment of negative symptoms of schizophrenia: therapeutic opportunity or cul-de-sac? Acta Psychiatr Scand. 2007;115(2):93-100. doi: 10.1111/j.1600-0447.2007.00992.x. PMID: 17244172.	Review
444	Smith B. Nurse-led cognitive behaviour therapy improved insight and reduced negative symptoms and readmissions in schizophrenia. Evid Based Med. 2007;12(1):21. doi: 10.1136/ebm.12.1.21. PMID: 17264269.	No drug
445	Prikryl R, Skotakova S, Kasparek T, Ceskova E, Kucerova H, Ustohal L. Influencing negative symptoms of schizophrenia with repetitive transcranial magnetic stimulation: a case study. Acta Neuropsychiatr. 2007;19(1):53-5. doi: 10.1111/j.1601-5215.2006.00166.x. PMID: 26952798.	Case
446	Koen L, Uys S, Niehaus DJ, Emsley RA. Negative symptoms and HIV/AIDS risk- behavior knowledge in schizophrenia. Psychosomatics. 2007;48(2):128-34. doi: 10.1176/appi.psy.48.2.128. PMID: 17329606.	No drug
447	Landeen J. Brief cognitive behaviour therapy improved insight and reduced negative symptoms and readmissions in schizophrenia. Evid Based Nurs. 2007;10(2):51. doi: 10.1136/ebn.10.2.51. PMID: 17384103.	No drug
448	Lindenmayer J-P, Khan A, Iskander A, Abad MT, Parker B. A randomized controlled trial of olanzapine versus haloperidol in the treatment of primary negative symptoms and neurocognitive deficits in schizophrenia. J Clin Psychiatry. 2007;68(3):368-79. doi: 10.4088/jcp.v68n0303. PMID: 17388705.	**Included**
449	Ko YH, Jung SW, Joe SH, Lee CH, Jung HG, Jung IK, Kim SH, Lee MS. Association between serum testosterone levels and the severity of negative symptoms in male patients with chronic schizophrenia. Psychoneuroendocrinology. 2007;32(4):385-91. doi: 10.1016/j.psyneuen.2007.02.002. Epub 2007 Mar 28. PMID: 17395394.	No drug
450	Ciudad A, Alvarez E, Bousoño M, Olivares JM, Gómez JC. Seguridad y tolerabilidad de olanzapina y risperidona: un estudio aleatorizado de 1 año de duración en pacientes con esquizofrenia y sintomatologia negativa prominente tratados de manera ambulatoria [Safety and tolerability of olanzapine versus risperidone: a one-year randomized study in outpatients with schizophrenia with prominent negative symptoms]. Actas Esp Psiquiatr. 2007;35(2):105-14. Spanish. PMID: 17401781.	Unfocused
451	Fohey KD, Hieber R, Nelson LA. The role of selegiline in the treatment of negative symptoms associated with schizophrenia. Ann Pharmacother. 2007;41(5):851-6. doi: 10.1345/aph.1H556. Epub 2007 Apr 3. PMID: 17405823.	Review
452	Ruhrmann S, Kissling W, Lesch OM, Schmauss M, Seemann U, Philipp M. Efficacy of flupentixol and risperidone in chronic schizophrenia with predominantly negative symptoms. Prog Neuropsychopharmacol Biol Psychiatry. 2007;31(5):1012-22. doi: 10.1016/j.pnpbp.2007.02.014. Epub 2007 Mar 1. PMID: 17412473.	**Included**
453	Pondé MP, Novaes CM. Aripiprazole worsening positive symptoms and memantine reducing negative symptoms in a patient with paranoid schizophrenia. Braz J Psychiatry. 2007;29(1):92. doi: 10.1590/s1516-44462007000100028. PMID: 17435943.	Case
454	Salvatore G, Dimaggio G, Lysaker PH. An intersubjective perspective on negative symptoms of schizophrenia: implications of simulation theory. Cogn Neuropsychiatry. 2007;12(2):144-64. doi: 10.1080/13546800600819921. PMID: 17453896.	Case
455	Tarrier N, Gooding P, Gregg L, Johnson J, Drake R; Socrates Trial Group. Suicide schema in schizophrenia: the effect of emotional reactivity, negative symptoms and schema elaboration. Behav Res Ther. 2007;45(9):2090-7. doi: 10.1016/j.brat.2007.03.007. Epub 2007 Mar 18. PMID: 17466940.	No drug
456	Sepehry AA, Potvin S, Elie R, Stip E. Selective serotonin reuptake inhibitor (SSRI) add-on therapy for the negative symptoms of schizophrenia: a meta-analysis. J Clin Psychiatry. 2007;68(4):604-10. doi: 10.4088/jcp.v68n0417. PMID: 17474817.	Review
457	Mogg A, Purvis R, Eranti S, Contell F, Taylor JP, Nicholson T, Brown RG, McLoughlin DM. Repetitive transcranial magnetic stimulation for negative symptoms of schizophrenia: a randomized controlled pilot study. Schizophr Res. 2007;93(1-3):221-8. doi: 10.1016/j.schres.2007.03.016. Epub 2007 May 2. PMID: 17478080.	No drug
458	Müller MJ. Gender-specific associations of depression with positive and negative symptoms in acute schizophrenia. Prog Neuropsychopharmacol Biol Psychiatry. 2007;31(5):1095-100. doi: 10.1016/j.pnpbp.2007.03.014. Epub 2007 Mar 30. PMID: 17493732.	No drug
459	Thoma P, Zoppelt D, Wiebel B, Daum I. Context processing and negative symptoms in schizophrenia. J Clin Exp Neuropsychol. 2007;29(4):428-35. doi: 10.1080/13803390600744814. PMID: 17497566.	No drug
460	Pierre JM, Peloian JH, Wirshing DA, Wirshing WC, Marder SR. A randomized, double-blind, placebo-controlled trial of modafinil for negative symptoms in schizophrenia. J Clin Psychiatry. 2007;68(5):705-10. doi: 10.4088/jcp.v68n0507. PMID: 17503979.	**Included**
461	Martino DJ, Bucay D, Butman JT, Allegri RF. Neuropsychological frontal impairments and negative symptoms in schizophrenia. Psychiatry Res. 2007;152(2-3):121-8. doi: 10.1016/j.psychres.2006.03.002. Epub 2007 May 15. PMID: 17507100.	No drug
462	Alphs L, Panagides J, Lancaster S. Asenapine in the treatment of negative symptoms of schizophrenia: clinical trial design and rationale. Psychopharmacol Bull. 2007;40(2):41-53. PMID: 17514185.	Protocol
463	Lecrubier Y, Perry R, Milligan G, Leeuwenkamp O, Morlock R. Physician observations and perceptions of positive and negative symptoms of schizophrenia: a multinational, cross-sectional survey. Eur Psychiatry. 2007;22(6):371-9. doi: 10.1016/j.eurpsy.2007.03.003. Epub 2007 May 23. PMID: 17521890.	No drug
464	Möller HJ. Clinical evaluation of negative symptoms in schizophrenia. Eur Psychiatry. 2007;22(6):380-6. doi: 10.1016/j.eurpsy.2007.03.010. Epub 2007 May 23. PMID: 17524626.	No drug

465	Gurpegui M, Alvarez E, Bousoño M, Ciudad A, Carlos Gómez J, Olivares JM. Effect of olanzapine or risperidone treatment on some cognitive functions in a one-year follow-up of schizophrenia outpatients with prominent negative symptoms. Eur Neuropsychopharmacol. 2007;17(11):725-34. doi: 10.1016/j.euroneuro.2007.04.003. Epub 2007 May 31. PMID: 17543505.	Open
466	Roffman JL, Weiss AP, Purcell S, Caffalette CA, Freudenreich O, Henderson DC, Bottiglieri T, Wong DH, Halsted CH, Goff DC. Contribution of methylenetetrahydrofolate reductase (MTHFR) polymorphisms to negative symptoms in schizophrenia. Biol Psychiatry. 2008;63(1):42-8. doi: 10.1016/j.biopsych.2006.12.017. Epub 2007 Jun 1. PMID: 17543893.	No drug
467	Kawasaki Y, Sumiyoshi T, Higuchi Y, Ito T, Takeuchi M, Kurachi M. Voxel- based analysis of P300 electrophysiological topography associated with positive and negative symptoms of schizophrenia. Schizophr Res. 2007;94(1-3):164-71. doi: 10.1016/j.schres.2007.04.015. Epub 2007 Jun 4. PMID: 17544631.	No drug
468	Danki D, Dilbaz N, Okay IT, Telci S. Sizofreni Tansi Olan Hastalarda Içgörünün Aile Oyküsü, Pozitif ve Negatif Belirtilerle Ilişkisi [Insight in schizophrenia: relationship to family history, and positive and negative symptoms]. Türk Psikiyatri Derg. 2007;18(2):129-36. Turkish. PMID: 17566878.	No drug
469	Riedel M, Spellmann I, Strassnig M, Douhet A, Dehning S, Opgen-Rhein M, Valdevit R, Engel RR, Kleindienst N, Müller N, Möller HJ. Effects of risperidone and quetiapine on cognition in patients with schizophrenia and predominantly negative symptoms. Eur Arch Psychiatry Clin Neurosci. 2007;257(6):360-70. doi: 10.1007/s00406-007-0739-x. Epub 2007 Jul 14. PMID: 17629731.	Overlap
470	Bertolino A, Caforio G, Blasi G, Rampino A, Nardini M, Weinberger DR, Dallapiccola B, Sinibaldi L, Douzgou S. COMT Val158Met polymorphism predicts negative symptoms response to treatment with olanzapine in schizophrenia. Schizophr Res. 2007;95(1-3):253-5. doi: 10.1016/j.schres.2007.06.014. Epub 2007 Jul 17. PMID: 17644310.	Unfocused
471	Aichhorn W, Stelzig-Schoeler R, Geretsegger C, Stuppaeck C, Kemmler G. Bright light therapy for negative symptoms in schizophrenia: a pilot study. J Clin Psychiatry. 2007;68(7):1146. doi: 10.4088/jcp.v68n0726a. PMID: 17685757.	No drug
472	Prikryl R, Kasparek T, Skotakova S, Ustohal L, Kucerova H, Ceskova E. Treatment of negative symptoms of schizophrenia using repetitive transcranial magnetic stimulation in a double-blind, randomized controlled study. Schizophr Res. 2007;95(1-3):151-7. doi: 10.1016/j.schres.2007.06.019. Epub 2007 Aug 8. Erratum in: Schizophr Res. 2008 Feb;99(1-3):380-1. PMID: 17689931.	No drug
473	Lysaker PH, Salyers MP. Anxiety symptoms in schizophrenia spectrum disorders: associations with social function, positive and negative symptoms, hope and trauma history. Acta Psychiatr Scand. 2007;116(4):290-8. doi: 10.1111/j.1600-0447.2007.01067.x. PMID: 17803759.	No drug
474	Carter CS. Some rewarding insights into the cognitive and neurobiological basis of negative symptoms in schizophrenia. Biol Psychiatry. 2007;62(7):709-10. doi: 10.1016/j.biopsych.2007.07.012. PMID: 17854580.	Opinion
475	Winograd-Gurvich C, Fitzgerald PB, Georgiou-Karistianis N, Millist L, White O. Inhibitory control and spatial working memory: a saccadic eye movement study of negative symptoms in schizophrenia. Psychiatry Res. 2008;157(1-3):9-19. doi: 10.1016/j.psychres.2007.02.004. Epub 2007 Sep 27. PMID: 17897722.	No drug
476	Buchanan RW, Javitt DC, Marder SR, Schooler NR, Gold JM, McMahon RP, Heresco-Levy U, Carpenter WT. The Cognitive and Negative Symptoms in Schizophrenia Trial (CONSIST): the efficacy of glutamatergic agents for negative symptoms and cognitive impairments. Am J Psychiatry. 2007;164(10):1593-602. doi: 10.1176/appi.ajp.2007.06081358. PMID: 17898352.	**Included**
477	Cohen AS, Alpert M, Nienow TM, Dinzeo TJ, Docherty NM. Computerized measurement of negative symptoms in schizophrenia. J Psychiatr Res. 2008;42(10):827-36. doi: 10.1016/j.jpsychires.2007.08.008. Epub 2007 Oct 24. PMID: 17920078; PMCID: PMC2488151.	No drug
478	Prikryl R, Kucerova H. Repetitive transcranial magnetic stimulation as new technique for treatment of negative symptoms of schizophrenia. Acta Psychiatr Scand. 2008;117(1):78-9; author reply 79. doi: 10.1111/j.1600-0447.2007.01104.x. Epub 2007 Oct 17. PMID: 17941965.	Opinion
479	Lecrubier Y. Review: adding SSRIs to antipsychotics does not improve negative symptoms in people with schizophrenia. Evid Based Ment Health. 2007;10(4):113. doi: 10.1136/ebmh.10.4.113. PMID: 17962663.	Review
480	Amiri A, Noorbala AA, Nejatisafa AA, Ghoreishi A, Derakhshan MK, Khodaie- Ardakani MR, Hajiazim M, Raznahan M, Akhondzadeh S. Efficacy of selegiline add on therapy to risperidone in the treatment of the negative symptoms of schizophrenia: a double-blind randomized placebo-controlled study. Hum Psychopharmacol. 2008;23(2):79-86. doi: 10.1002/hup.902. PMID: 17972359.	**Included**
481	Puig O, Penadés R, Gastó C, Catalán R, Torres A, Salamero M. Verbal memory, negative symptomatology and prediction of psychosocial functioning in schizophrenia. Psychiatry Res. 2008;158(1):11-7. doi: 10.1016/j.psychres.2007.04.017. Epub 2007 Nov 14. PMID: 18001844.	No drug
482	Compton MT, Whicker NE, Hochman KM. Alcohol and cannabis use in Urban, African American, first-episode schizophrenia-spectrum patients: associations with positive and negative symptoms. J Clin Psychiatry. 2007;68(12):1939-45. doi: 10.4088/jcp.v68n1215. PMID: 18162026.	No drug
483	Fitzgerald PB, Herring S, Hoy K, McQueen S, Segrave R, Kulkarni J, Daskalakis ZJ. A study of the effectiveness of bilateral transcranial magnetic stimulation in the treatment of the negative symptoms of schizophrenia. Brain Stimul. 2008;1(1):27-32. doi: 10.1016/j.brs.2007.08.001. Epub 2007 Dec 3. PMID: 20633367	No drug
484	Grant PM, Beck AT. Defeatist beliefs as a mediator of cognitive impairment, negative symptoms, and functioning in schizophrenia. Schizophr Bull. 2009;35(4):798-806. doi: 10.1093/schbul/sbn008. Epub 2008 Feb 27. PMID: 18308717; PMCID: PMC2696369.	No drug
485	Wang L, Fang C, Zhang A, Du J, Yu L, Ma J, Feng G, Xing Q, He L. The –1019 C/G polymorphism of the 5-HT1A receptor gene is associated with negative symptom response to risperidone treatment in schizophrenia patients. J Psychopharmacol. 2008;22(8):904-9. doi: 10.1177/0269881107081522. Epub 2008 Feb 28. PMID: 18308786.	Open
486	Silver H. Selective serotonin reuptake inhibitor add-on therapy for the negative symptoms of schizophrenia. J Clin Psychiatry. 2008;69(1):163-4. doi: 10.4088/jcp.v69n0122b. PMID: 18312053.	Opinion
487	Murphy BP. Inconclusive evidence for the efficacy of olanzapine in the treatment of negative symptoms in schizophrenia. J Clin Psychiatry. 2008;69(1):164; author reply 164-5. doi: 10.4088/jcp.v69n0122c. PMID: 18312055.	Opinion
488	Schneider AL, Schneider TL, Stark H. Repetitive transcranial magnetic stimulation (rTMS) as an augmentation treatment for the negative symptoms of schizophrenia: a 4-week randomized placebo controlled study. Brain Stimul. 2008;1(2):106-11. doi: 10.1016/j.brs.2008.01.001. Epub 2008 Mar 31. PMID: 20633377.	No drug
489	Matsuzawa D, Obata T, Shirayama Y, Nonaka H, Kanazawa Y, Yoshitome E, Takanashi J, Matsuda T, Shimizu E, Ikehira H, Iyo M, Hashimoto K. Negative correlation between brain glutathione level and negative symptoms in schizophrenia: a 3T 1H-MRS study. PLoS One. 2008;3(4):e1944. doi: 10.1371/journal.pone.0001944. PMID: 18398470; PMCID: PMC2275307.	No drug
490	Zetzsche T, Preuss UW, Frodl T, Leinsinger G, Born C, Reiser M, Hegerl U, Möller HJ, Meisenzahl EM. White matter alterations in schizophrenic patients with pronounced negative symptomatology and with positive family history for schizophrenia. Eur Arch Psychiatry Clin Neurosci. 2008;258(5):278-84. doi: 10.1007/s00406-007-0793-4. Epub 2008 Apr 24. PMID: 18437282.	No drug
491	Wang YH, Li WQ, Huang Z, Shi YZ, Wang XY, Huang JS, Zhou XH, Chen HX, Hao W. 5-HT2A receptor gene polymorphism and negative symptoms in first episode (drug-naive) Chinese Han nationality individuals with schizophrenia. Zhong Nan Da Xue Xue Bao Yi Xue Ban. 2008;33(4):293-8. PMID: 18460771.	No drug
492	Wolf DH, Turetsky BI, Loughead J, Elliott MA, Pratiwadi R, Gur RE, Gur RC. Auditory oddball fMRI in schizophrenia: Association of negative symptoms with regional hypoactivation to novel distractors. Brain Imaging Behav. 2008;2(2):132-145. doi: 10.1007/s11682-008-9022-7. PMID: 19756228; PMCID: PMC2743436.	No drug
493	Vuchetich JP, Liska JL, Dionisio DP, Stanwyck JJ, McGuire KA, Sponheim SR. Elevated nailfold plexus visibility aggregates in families and is associated with a specific negative symptom pattern in schizophrenia. Psychiatry Res. 2008;160(1):30-7. doi: 10.1016/j.psychres.2007.04.024. Epub 2008 Jun 2. PMID: 18514327; PMCID: PMC6379205.	No drug

494	Melle I, Larsen TK, Haahr U, Friis S, Johannesen JO, Opjordsmoen S, Rund BR, Simonsen E, Vaglum P, McGlashan T. Prevention of negative symptom psychopathologies in first-episode schizophrenia: two-year effects of reducing the duration of untreated psychosis. Arch Gen Psychiatry. 2008;65(6):634-40. doi: 10.1001/archpsyc.65.6.634. PMID: 18519821.	No drug
495	Kelley ME, Haas GL, van Kammen DP. Longitudinal progression of negative symptoms in schizophrenia: a new look at an old problem. Schizophr Res. 2008;105(1-3):188-96. doi: 10.1016/j.schres.2008.06.003. Epub 2008 Jul 11. PMID: 18619815; PMCID: PMC2600772.	No drug
496	Foussias G, Remington G. Negative symptoms in schizophrenia: avolition and Occam's razor. Schizophr Bull. 2010;36(2):359-69. doi: 10.1093/schbul/sbn094. Epub 2008 Jul 21. PMID: 18644851; PMCID: PMC2833114.	Review
497	Batki SL, Leontieva L, Dimmock JA, Ploutz-Snyder R. Negative symptoms are associated with less alcohol use, craving, and "high" in alcohol dependent patients with schizophrenia. Schizophr Res. 2008;105(1-3):201-7. doi: 10.1016/j.schres.2008.06.020. Epub 2008 Aug 13. PMID: 18701256; PMCID: PMC2582942.	No drug
498	Mäkinen J, Miettunen J, Isohanni M, Koponen H. Negative symptoms in schizophrenia: a review. Nord J Psychiatry. 2008;62(5):334-41. doi: 10.1080/08039480801959307. PMID: 18752104.	Review
499	Gurvich CT, Fitzgerald PB, Georgiou-Karistianis N, White OB. Saccadic impairment in schizophrenia with prominent negative symptoms. Neuroreport. 2008;19(14):1435-9. doi: 10.1097/WNR.0b013e32830e4622. PMID: 18766026.	No drug
500	Goff DC, Cather C, Gottlieb JD, Evins AE, Walsh J, Raeke L, Otto MW, Schoenfeld D, Green MF. Once-weekly D-cycloserine effects on negative symptoms and cognition in schizophrenia: an exploratory study. Schizophr Res. 2008;106(2-3):320-7. doi: 10.1016/j.schres.2008.08.012. Epub 2008 Sep 16. PMID: 18799288; PMCID: PMC2628436.	**Included**
501	Akhondzadeh S, Malek-Hosseini M, Ghoreishi A, Raznahan M, Rezazadeh SA. Effect of ritanserin, a 5HT2A/2C antagonist, on negative symptoms of schizophrenia: a double-blind randomized placebo-controlled study. Prog Neuropsychopharmacol Biol Psychiatry. 2008;32(8):1879-83. doi: 10.1016/j.pnpbp.2008.08.020. Epub 2008 Sep 4. PMID: 18801405.	**Included**
502	Saavedra-Velez C, Yusim A, Anbarasan D, Lindenmayer JP. Modafinil as an adjunctive treatment of sedation, negative symptoms, and cognition in schizophrenia: a critical review. J Clin Psychiatry. 2009;70(1):104-12. doi: 10.4088/jcp.07r03982. Epub 2008 Nov 18. PMID: 19026265.	Review
503	Rodríguez-Sánchez JM, Crespo-Facorro B, González-Blanch C, Pérez-Iglesias R, Alvarez-Jiménez M, Martínez O, Vázquez-Barquero JL. Cognitive functioning and negative symptoms in first episode schizophrenia: different patterns of correlates. Neurotox Res. 2008;14(2-3):227-35. doi: 10.1007/BF03033812. PMID: 19073428.	No drug
504	Stip E, Mancini-Marïe A, Letourneau G, Fahim C, Mensour B, Crivello F, Dollfus S. Increased grey matter densities in schizophrenia patients with negative symptoms after treatment with quetiapine: a voxel-based morphometry study. Int Clin Psychopharmacol. 2009;24(1):34-41. doi: 10.1097/YIC.0b013e32831daf6c. PMID: 19077676.	Unfocused
505	Silver H, Chertkow Y, Weinreb O, Danovich L, Youdim M. Multifunctional pharmacotherapy: what can we learn from study of selective serotonin reuptake inhibitor augmentation of antipsychotics in negative-symptom schizophrenia? Neurotherapeutics. 2009;6(1):86-93. doi: 10.1016/j.nurt.2008.10.034. PMID: 19110201; PMCID: PMC5084258.	Review
506	Turkoz I, Bossie CA, Dirks B, Canuso CM. Direct and indirect effects of paliperidone extended-release tablets on negative symptoms of schizophrenia. Neuropsychiatr Dis Treat. 2008;4(5):949-58. doi: 10.2147/ndt.s3668. PMID: 19183785; PMCID: PMC2626923.	*Post hoc*
507	Rocca P, Montemagni C, Castagna F, Giugiario M, Scalese M, Bogetto F. Relative contribution of antipsychotics, negative symptoms and executive functions to social functioning in stable schizophrenia. Prog Neuropsychopharmacol Biol Psychiatry. 2009;33(2):373-9. doi: 10.1016/j.pnpbp.2009.01.002. Epub 2009 Jan 11. PMID: 19211031.	Open
508	Lysaker PH, Vohs JL, Tsai J. Negative symptoms and concordant impairments in attention in schizophrenia: associations with social functioning, hope, self- esteem and internalized stigma. Schizophr Res. 2009;110(1-3):165-72. doi: 10.1016/j.schres.2009.01.015. Epub 2009 Feb 20. PMID: 19230622.	No drug
509	Johnson DP, Penn DL, Fredrickson BL, Meyer PS, Kring AM, Brantley M. Loving-kindness meditation to enhance recovery from negative symptoms of schizophrenia. J Clin Psychol. 2009;65(5):499-509. doi: 10.1002/jclp.20591. PMID: 19267396.	No drug
510	Brill N, Levine SZ, Reichenberg A, Lubin G, Weiser M, Rabinowitz J. Pathways to functional outcomes in schizophrenia: the role of premorbid functioning, negative symptoms and intelligence. Schizophr Res. 2009;110(1-3):40-6. doi: 10.1016/j.schres.2009.02.016. Epub 2009 Mar 17. PMID: 19297133.	No drug
511	Avery R, Startup M, Calabria K. The role of effort, cognitive expectancy appraisals and coping style in the maintenance of the negative symptoms of schizophrenia. Psychiatry Res. 2009;167(1-2):36-46. doi: 10.1016/j.psychres.2008.04.016. Epub 2009 Mar 31. PMID: 19339056.	No drug
512	Bor J, Brunelin J, Rivet A, d'Amato T, Poulet E, Saoud M, Padberg F. Effects of theta burst stimulation on glutamate levels in a patient with negative symptoms of schizophrenia. Schizophr Res. 2009;111(1-3):196-7. doi: 10.1016/j.schres.2009.03.012. Epub 2009 Mar 31. PMID: 19339158.	No drug
513	Marx CE, Keefe RS, Buchanan RW, Hamer RM, Kilts JD, Bradford DW, Strauss JL, Naylor JC, Payne VM, Lieberman JA, Savitz AJ, Leimone LA, Dunn L, Porcu P, Morrow AL, Shampine LJ. Proof-of-concept trial with the neurosteroid pregnenolone targeting cognitive and negative symptoms in schizophrenia. Neuropsychopharmacology. 2009;34(8):1885-903. doi: 10.1038/npp.2009.26. Epub 2009 Apr 1. PMID: 19339966; PMCID: PMC3427920.	**Included**
514	Anghelescu I. Successful smoking cessation and improvement of negative symptoms with varenicline in a stable schizophrenia patient. J Neuropsychiatry Clin Neurosci. 2009;21(1):102-3. doi: 10.1176/jnp.2009.21.1.102. PMID: 19359464.	Case
515	Hauser TA, Kucinski A, Jordan KG, Gatto GJ, Wersinger SR, Hesse RA, Stachowiak EK, Stachowiak MK, Papke RL, Lippiello PM, Bencherif M. TC-5619: an alpha7 neuronal nicotinic receptor-selective agonist that demonstrates efficacy in animal models of the positive and negative symptoms and cognitive dysfunction of schizophrenia. Biochem Pharmacol. 2009;78(7):803-12. doi: 10.1016/j.bcp.2009.05.030. Epub 2009 May 29. PMID: 19482012; PMCID: PMC3005247.	Animal
516	Canuso CM, Bossie CA, Turkoz I, Alphs L. Paliperidone extended-release for schizophrenia: effects on symptoms and functioning in acutely ill patients with negative symptoms. Schizophr Res. 2009;113(1):56-64. doi: 10.1016/j.schres.2009.05.021. Epub 2009 Jun 26. PMID: 19560322.	*Post hoc*
517	Chertkow Y, Weinreb O, Youdim MB, Silver H. Molecular mechanisms underlying synergistic effects of SSRI-antipsychotic augmentation in treatment of negative symptoms in schizophrenia. J Neural Transm (Vienna). 2009;116(11):1529-41. doi: 10.1007/s00702-009-0255-4. Epub 2009 Jul 4. PMID: 19578925.	Review
518	Bennett M. Positive and negative symptoms in schizophrenia: the NMDA receptor hypofunction hypothesis, neuregulin/ErbB4 and synapse regression. Aust N Z J Psychiatry. 2009;43(8):711-21. doi: 10.1080/00048670903001943. PMID: 19629792.	No drug
519	Grauer SM, Pulito VL, Navarra RL, Kelly MP, Kelley C, Graf R, Langen B, Logue S, Brennan J, Jiang L, Charych E, Egerland U, Liu F, Marquis KL, Malamas M, Hage T, Comery TA, Brandon NJ. Phosphodiesterase 10A inhibitor activity in preclinical models of the positive, cognitive, and negative symptoms of schizophrenia. J Pharmacol Exp Ther. 2009;331(2):574-90. doi: 10.1124/jpet.109.155994. Epub 2009 Aug 6. PMID: 19661377.	Animal
520	Velligan DI, Alphs L, Lancaster S, Morlock R, Mintz J. Association between changes on the Negative Symptom Assessment scale (NSA-16) and measures of functional outcome in schizophrenia. Psychiatry Res. 2009;169(2):97-100. doi: 10.1016/j.psychres.2008.10.009. Epub 2009 Aug 7. PMID: 19664826.	No drug
521	Wirgenes KV, Djurovic S, Agartz I, Jonsson EG, Werge T, Melle I, Andreassen OA. Dysbindin and d-amino-acid-oxidase gene polymorphisms associated with positive and negative symptoms in schizophrenia. Neuropsychobiology. 2009;60(1):31-6. doi: 10.1159/000235799. Epub 2009 Sep 1. PMID: 19729970.	No drug

522	Hough D, Nuamah IF, Lim P, Sampson A, Gagnon DD, Rothman M. Independent effect of paliperidone extended release on social functioning beyond its effect on positive and negative symptoms of schizophrenia: a mediation analysis. J Clin Psychopharmacol. 2009;29(5):496-7. doi: 10.1097/JCP.0b013e3181b5f40b. PMID: 19745652.	Unfocused
523	Halari R, Premkumar P, Farquharson L, Fannon D, Kuipers E, Kumari V. Rumination and negative symptoms in schizophrenia. J Nerv Ment Dis. 2009;197(9):703-6. doi: 10.1097/NMD.0b013e3181b3af20. PMID: 19752652.	No drug
524	Ishizuka K, Tajinda K, Colantuoni C, Morita M, Winicki J, Le C, Lin S, Schretlen D, Sawa A, Cascella NG. Negative symptoms of schizophrenia correlate with impairment on the University of Pennsylvania smell identification test. Neurosci Res. 2010;66(1):106-10. doi: 10.1016/j.neures.2009.10.001. Epub 2009 Oct 9. PMID: 19819272; PMCID: PMC4064297.	No drug
525	Strauss GP, Duke LA, Ross SA, Allen DN. Posttraumatic stress disorder and negative symptoms of schizophrenia. Schizophr Bull. 2011;37(3):603-10. doi: 10.1093/schbul/sbp122. Epub 2009 Nov 4. PMID: 19889949; PMCID: PMC3080684.	No drug
526	Bobes J, Arango C, Garcia-Garcia M, Rejas J; CLAMORS Study Collaborative Group. Prevalence of negative symptoms in outpatients with schizophrenia spectrum disorders treated with antipsychotics in routine clinical practice: findings from the CLAMORS study. J Clin Psychiatry. 2010;71(3):280-6. doi: 10.4088/JCP.08m04250yel. Epub 2009 Nov 3. Erratum in: J Clin Psychiatry. 2011;72(7):1017. PMID: 19895779.	No drug
527	Klingberg S, Wittorf A, Herrlich J, Wiedemann G, Meisner C, Buchkremer G, Frommann N, Wölwer W. Cognitive behavioural treatment of negative symptoms in schizophrenia patients: study design of the TONES study, feasibility and safety of treatment. Eur Arch Psychiatry Clin Neurosci. 2009;259 Suppl 2:S149-54. doi: 10.1007/s00406-009-0047-8. PMID: 19876673.	No drug
528	Cordes J, Falkai P, Guse B, Hasan A, Schneider-Axmann T, Arends M, Winterer G, Wölwer W, Ben Sliman E, Ramacher M, Schmidt-Kraepelin C, Ohmann C, Langguth B, Landgrebe M, Eichhammer P, Frank E, Burger J, Hajak G, Rietschel M, Wobrock T. Repetitive transcranial magnetic stimulation for the treatment of negative symptoms in residual schizophrenia: rationale and design of a sham-controlled, randomized multicenter study. Eur Arch Psychiatry Clin Neurosci. 2009;259 Suppl 2:S189-97. doi: 10.1007/s00406-009-0060-y. PMID: 19876678.	No drug
529	O'Tuathaigh CM, Kirby BP, Moran PM, Waddington JL. Mutant mouse models: genotype-phenotype relationships to negative symptoms in schizophrenia. Schizophr Bull. 2010;36(2):271-88. doi: 10.1093/schbul/sbp125. Epub 2009 Nov 24. PMID: 19934211; PMCID: PMC2833123.	Animal
530	Goto N, Yoshimura R, Kakeda S, Moriya J, Hayashi K, Ikenouchi-Sugita A, Umene-Nakano W, Hori H, Ueda N, Korogi Y, Nakamura J. Associations between plasma levels of 3-methoxy-4-hydroxyphenylglycol (MHPG) and negative symptoms or cognitive impairments in early-stage schizophrenia. Hum Psychopharmacol. 2009;24(8):639-45. doi: 10.1002/hup.1070. PMID: 19946939.	No drug
531	Ozguven HD, Oner O, Baskak B, Oktem F, Olmez S, Munir K. Theory of Mind in schizophrenia and Asperger's Syndrome: Relationship with negative symptoms. Klinik Psikofarmakol Bulteni. 2010;20(1):5-13. doi: 10.1080/10177833.2010.11790628. PMID: 25584026; PMCID: PMC4288770.	No drug
532	Weinberg D, Shahar G, Davidson L, McGlashan TH, Fennig S. Longitudinal associations between negative symptoms and social functioning in schizophrenia: the moderating role of employment status and setting. Psychiatry. 2009;72(4):370-81. doi: 10.1521/psyc.2009.72.4.370. PMID: 20070135.	No drug
533	Vakalopoulos C. A new nosology of psychosis and the pharmacological basis of affective and negative symptom dimensions in schizophrenia. Ment Illn. 2010;2(1):e7. doi: 10.4081/mi.2010.e7. PMID: 25478090; PMCID: PMC4253346.	Unrelated
534	Hons J, Zirko R, Ulrychova M, Cermakova E, Doubek P, Libiger J. Glycine serum level in schizophrenia: relation to negative symptoms. Psychiatry Res. 2010;176(2-3):103-8. doi: 10.1016/j.psychres.2009.11.008. Epub 2010 Jan 22. PMID: 20096465.	No drug
535	Tsygankov BD, Ovsiannikov SA, Khannanova AN. [Methodological approaches to assessment of negative symptoms during pharmacotherapy of schizophrenia]. Zh Nevrol Psikhiatr Im S S Korsakova. 2009;109(11):101-6. Russian. PMID: 20120071.	No drug
536	Vardigan JD, Huszar SL, McNaughton CH, Hutson PH, Uslaner JM. MK-801 produces a deficit in sucrose preference that is reversed by clozapine, D-serine, and the metabotropic glutamate 5 receptor positive allosteric modulator CDPPB: relevance to negative symptoms associated with schizophrenia? Pharmacol Biochem Behav. 2010;95(2):223-9. doi: 10.1016/j.pbb.2010.01.010. Epub 2010 Feb 1. PMID: 20122952.	Animal
537	Makara-Studzińska M, Koślak A. Wpływ objawów pozytywnych i negatywnych na zachowania samobójcze w schizofrenii. Przeglad aktualnej literatury [Influence of positive and negative symptoms on suicidal behaviour in schizophrenia. Review of current literature]. Psychiatr Pol. 2009;43(4):411-20. Polish. PMID: 20128249.	Review
538	Nakano Y, Yoshimura R, Nakano H, Ikenouchi-Sugita A, Hori H, Umene-Nakano W, Ueda N, Nakamura J. Association between plasma nitric oxide metabolites levels and negative symptoms of schizophrenia: a pilot study. Hum Psychopharmacol. 2010;25(2):139-44. doi: 10.1002/hup.1102. PMID: 20196178.	No drug
539	Esterberg ML, Trotman HD, Holtzman C, Compton MT, Walker EF. The impact of a family history of psychosis on age-at-onset and positive and negative symptoms of schizophrenia: a meta-analysis. Schizophr Res. 2010;120(1-3):121-30. doi: 10.1016/j.schres.2010.01.011. Epub 2010 Mar 19. PMID: 20303240.	Review
540	Dlabac-de Lange JJ, Knegtering R, Aleman A. Repetitive transcranial magnetic stimulation for negative symptoms of schizophrenia: review and meta- analysis. J Clin Psychiatry. 2010;71(4):411-8. doi: 10.4088/JCP.08r04808yel. Epub 2010 Feb 23. PMID: 20361909.	Review
541	Tsai J, Lysaker PH, Vohs JL. Negative symptoms and concomitant attention deficits in schizophrenia: associations with prospective assessments of anxiety, social dysfunction, and avoidant coping. J Ment Health. 2010;19(2):184-92. doi: 10.3109/09638230903469277. PMID: 20433326; PMCID: PMC2864941.	No drug
542	Kupper Z, Ramseyer F, Hoffmann H, Kalbermatten S, Tschacher W. Video-based quantification of body movement during social interaction indicates the severity of negative symptoms in patients with schizophrenia. Schizophr Res. 2010;121(1-3):90-100. doi: 10.1016/j.schres.2010.03.032. PMID: 20434313.	No drug
543	Magomedov RA, Garakh ZhV, Orekhov IuV, Zaĭtseva IuS, Strelets VB. [Gamma- rhythm, positive, negative symptoms and cognitive dysfunction in schizophrenia]. Zh Nevrol Psikhiatr Im S S Korsakova. 2010;110(1):78-83. Russian. PMID: 20436442.	No drug
544	Iancu I, Tschernihovsky E, Bodner E, Piconne AS, Lowengrub K. Escitalopram in the treatment of negative symptoms in patients with chronic schizophrenia: a randomized double-blind placebo-controlled trial. Psychiatry Res. 2010;179(1):19-23. doi: 10.1016/j.psychres.2010.04.035. Epub 2010 May 15. PMID: 20472299.	**Included**
545	Mäkinen J, Miettunen J, Jääskeläinen E, Veijola J, Isohanni M, Koponen H. Negative symptoms and their predictors in schizophrenia within the Northern Finland 1966 Birth Cohort. Psychiatry Res. 2010;178(1):121-5. doi: 10.1016/j.psychres.2009.05.011. Epub 2010 May 16. PMID: 20483165.	No drug
546	Wang Y, Fang Y, Shen Y, Xu Q. Analysis of association between the catechol- O-methyltransferase (COMT) gene and negative symptoms in chronic schizophrenia. Psychiatry Res. 2010;179(2):147-50. doi: 10.1016/j.psychres.2009.03.029. Epub 2010 May 18. PMID: 20483479.	No drug
547	Matheson SL, Green MJ, Loo C, Carr VJ. A change in the conclusions of a recent systematic meta-review: repetitive transcranial magnetic stimulation is effective for the negative symptoms of schizophrenia. Schizophr Res. 2010;122(1-3):276-7. doi: 10.1016/j.schres.2010.05.029. Epub 2010 Jun 16. PMID: 20558042.	Opinion
548	Stahl SM, Malla A, Newcomer JW, Potkin SG, Weiden PJ, Harvey PD, Loebel A, Watsky E, Siu CO, Romano S. A post hoc analysis of negative symptoms and psychosocial function in patients with schizophrenia: a 40-week randomized, double-blind study of ziprasidone versus haloperidol followed by a 3-year double-blind extension trial. J Clin Psychopharmacol. 2010;30(4):425-30. doi: 10.1097/JCP.0b013e3181e69042. Erratum in: J Clin Psychopharmacol. 2010;30(5):652.	**Included**

549	Preuss UW, Zetzsche T, Pogarell O, Mulert C, Frodl T, Müller D, Schmidt G, Born C, Reiser M, Möller HJ, Hegerl U, Meisenzahl EM. Anterior cingulum volumetry, auditory P300 in schizophrenia with negative symptoms. Psychiatry Res. 2010;183(2):133-9. doi: 10.1016/j.pscychresns.2010.05.008. Epub 2010 Jul 13. PMID: 20630714.	No drug
550	Sethom MM, Fares S, Bouaziz N, Melki W, Jemaa R, Feki M, Hechmi Z, Kaabachi N. Polyunsaturated fatty acids deficits are associated with psychotic state and negative symptoms in patients with schizophrenia. Prostaglandins Leukot Essent Fatty Acids. 2010;83(3):131-6. doi: 10.1016/j.plefa.2010.07.001. Epub 2010 Jul 27. PMID: 20667702.	No drug
551	Neill JC, Barnes S, Cook S, Grayson B, Idris NF, McLean SL, Snigdha S, Rajagopal L, Harte MK. Animal models of cognitive dysfunction and negative symptoms of schizophrenia: focus on NMDA receptor antagonism. Pharmacol Ther. 2010;128(3):419-32. doi: 10.1016/j.pharmthera.2010.07.004. Epub 2010 Aug 10. PMID: 20705091.	Animal
552	Bitanihirwe BK, Peleg-Raibstein D, Mouttet F, Feldon J, Meyer U. Late prenatal immune activation in mice leads to behavioral and neurochemical abnormalities relevant to the negative symptoms of schizophrenia. Neuropsychopharmacology. 2010;35(12):2462-78. doi: 10.1038/npp.2010.129. Epub 2010 Aug 25. PMID: 20736993; PMCID: PMC3055332.	Animal
553	Bio DS, Gattaz WF. Vocational rehabilitation improves cognition and negative symptoms in schizophrenia. Schizophr Res. 2011;126(1-3):265-9. doi: 10.1016/j.schres.2010.08.003. Epub 2010 Aug 25. PMID: 20800453.	No drug
554	Alphs L, Morlock R, Coon C, van Willigenburg A, Panagides J. The 4-item Negative Symptom Assessment (NSA-4) instrument: A simple tool for evaluating negative symptoms in schizophrenia following brief training. Psychiatry (Edgmont). 2010;7(7):26-32. PMID: 20805916; PMCID: PMC2922363.	No drug
555	Singh SP, Singh V, Kar N, Chan K. Efficacy of antidepressants in treating the negative symptoms of chronic schizophrenia: meta-analysis. Br J Psychiatry. 2010;197(3):174-9. doi: 10.1192/bjp.bp.109.067710. Erratum in: Br J Psychiatry. 2011;198:159. PMID: 20807960.	Review
556	Hanson E, Healey K, Wolf D, Kohler C. Assessment of pharmacotherapy for negative symptoms of schizophrenia. Curr Psychiatry Rep. 2010;12(6):563-71. doi: 10.1007/s11920-010-0148-0. PMID: 20821286.	Review
557	Blanchard JJ, Kring AM, Horan WP, Gur R. Toward the next generation of negative symptom assessments: the collaboration to advance negative symptom assessment in schizophrenia. Schizophr Bull. 2011;37(2):291-9. doi: 10.1093/schbul/sbq104. Epub 2010 Sep 22. PMID: 20861151; PMCID: PMC3044636.	No drug
558	Jiawan VC, Arends J, Slooff CJ, Knegtering H. Medicamenteuze interventie bij negatieve symptomen bij patiënten met schizofrenie [Pharmacological treatment of negative symptoms in schizophrenia; research and practice]. Tijdschr Psychiatr. 2010;52(9):627-37. Dutch. PMID: 20862645.	Review
559	Messinger JW, Trémeau F, Antonius D, Mendelsohn E, Prudent V, Stanford AD, Malaspina D. Avolition and expressive deficits capture negative symptom phenomenology: implications for DSM-5 and schizophrenia research. Clin Psychol Rev. 2011;31(1):161-8. doi: 10.1016/j.cpr.2010.09.002. Epub 2010 Sep 18. PMID: 20889248; PMCID: PMC2997909.	No drug
560	Vedeniapin A. Natural antiviral cellular defense in relation to positive and negative symptoms of schizophrenia? Am J Psychiatry. 2010;167(10):1276; author reply 1276. doi: 10.1176/appi.ajp.2010.10040535. PMID: 20889667.	No drug
561	Akhondzadeh S, Ghayyoumi R, Rezaei F, Salehi B, Modabbernia AH, Maroufi A, Esfandiari GR, Naderi M, Ghebleh F, Tabrizi M, Rezazadeh SA. Sildenafil adjunctive therapy to risperidone in the treatment of the negative symptoms of schizophrenia: a double-blind randomized placebo-controlled trial. Psychopharmacology (Berl). 2011;213(4):809-15. doi: 10.1007/s00213-010-2044-z. Epub 2010 Oct 15. PMID: 20949350.	**Included**
562	Couture SM, Granholm EL, Fish SC. A path model investigation of neurocognition, theory of mind, social competence, negative symptoms and real- world functioning in schizophrenia. Schizophr Res. 2011;125(2-3):152-60. doi: 10.1016/j.schres.2010.09.020. Epub 2010 Oct 20. PMID: 20965699; PMCID: PMC3031755.	No drug
563	Stanford AD, Corcoran C, Bulow P, Bellovin-Weiss S, Malaspina D, Lisanby SH. High-frequency prefrontal repetitive transcranial magnetic stimulation for the negative symptoms of schizophrenia: a case series. J ECT. 2011;27(1):11-7. doi: 10.1097/YCT.0b013e3181f41ea3. PMID: 20966771; PMCID: PMC3042491.	Case
564	Dyck B, Guest K, Sookram C, Basu D, Johnson R, Mishra RK. PAOPA, a potent analogue of Pro-Leu-glycinamide and allosteric modulator of the dopamine D2 receptor, prevents NMDA receptor antagonist (MK-801)-induced deficits in social interaction in the rat: implications for the treatment of negative symptoms in schizophrenia. Schizophr Res. 2011;125(1):88-92. doi: 10.1016/j.schres.2010.09.025. Epub 2010 Oct 30. PMID: 21036015; PMCID: PMC3010311.	Animal
565	Rummel-Kluge C. Negative symptoms are prevalent in antipsychotic-treated adult outpatients with schizophrenia spectrum disorders. Evid Based Ment Health. 2010;13(4):106. doi: 10.1136/ebmh.13.4.106. PMID: 21036968.	Opinion
566	Cho SJ, Yook K, Kim B, Choi TK, Lee KS, Kim YW, Lee JE, Suh S, Yook KH, Lee SH. Mirtazapine augmentation enhances cognitive and reduces negative symptoms in schizophrenia patients treated with risperidone: a randomized controlled trial. Prog Neuropsychopharmacol Biol Psychiatry. 2011;35(1):208-11. doi: 10.1016/j.pnpbp.2010.11.006. Epub 2010 Nov 21. PMID: 21095214.	**Included**
567	Sugishita K, Yamasue H, Kasai K. Continuous positive airway pressure for obstructive sleep apnea improved negative symptoms in a patient with schizophrenia. Psychiatry Clin Neurosci. 2010;64(6):665. doi: 10.1111/j.1440-1819.2010.02146.x. PMID: 21105956.	Case
568	Rabany L, Weiser M, Werbeloff N, Levkovitz Y. Assessment of negative symptoms and depression in schizophrenia: revision of the SANS and how it relates to the PANSS and CDSS. Schizophr Res. 2011;126(1-3):226-30. doi: 10.1016/j.schres.2010.09.023. Epub 2010 Nov 5. PMID: 21115328.	No drug
569	Strauss GP, Frank MJ, Waltz JA, Kasanova Z, Herbener ES, Gold JM. Deficits in positive reinforcement learning and uncertainty-driven exploration are associated with distinct aspects of negative symptoms in schizophrenia. Biol Psychiatry. 2011;69(5):424-31. doi: 10.1016/j.biopsych.2010.10.015. Epub 2010 Dec 17. PMID: 21168124; PMCID: PMC3039035.	No drug
570	Tatsumi E, Yotsumoto K, Nakamae T, Hashimoto T. Effects of occupational therapy on hospitalized chronic schizophrenia patients with severe negative symptoms. Kobe J Med Sci. 2012;57(4):E145-54. PMID: 22971985.	No drug
571	Morrison PD, Stone JM. Synthetic delta-9-tetrahydrocannabinol elicits schizophrenia-like negative symptoms which are distinct from sedation. Hum Psychopharmacol. 2011;26(1):77-80. doi: 10.1002/hup.1166. Epub 2011 Feb 23. PMID: 23055415.	Unrelated
572	Kaushik GN. Re: sildenafil adjunctive therapy to risperidone in the treatment of the negative symptoms of schizophrenia. Psychopharmacology (Berl). 2011;215(2):401; author reply 399. doi: 10.1007/s00213-010-2155-6. Epub 2010 Dec 31. PMID: 21193982.	Opinion
573	Kasanova Z, Waltz JA, Strauss GP, Frank MJ, Gold JM. Optimizing vs. matching: response strategy in a probabilistic learning task is associated with negative symptoms of schizophrenia. Schizophr Res. 2011;127(1-3):215-22. doi: 10.1016/j.schres.2010.12.003. Epub 2011 Jan 15. PMID: 21239143; PMCID: PMC3051026.	No drug
574	Watanabe N. Fluoxetine, trazodone and ritanserin are more effective than placebo when used as add-on therapies for negative symptoms of schizophrenia. Evid Based Ment Health. 2011;14(1):21. doi: 10.1136/ebmh.14.1.21. PMID: 21266618.	Opinion
575	Vogel M, Meier J, Grönke S, Waage M, Schneider W, Freyberger HJ, Klauer T. Differential effects of childhood abuse and neglect: mediation by posttraumatic distress in neurotic disorder and negative symptoms in schizophrenia? Psychiatry Res. 2011;189(1):121-7. doi: 10.1016/j.psychres.2011.01.008. Epub 2011 Feb 3. PMID: 21295351.	No drug
576	McDowd J, Tang TC, Tsai PC, Wang SY, Su CY. The association between verbal memory, processing speed, negative symptoms and functional capacity in schizophrenia. Psychiatry Res. 2011;187(3):329-34. doi: 10.1016/j.psychres.2011.01.017. Epub 2011 Feb 12. PMID: 21320727.	No drug

577	Lysaker PH, Davis LW, Buck KD, Outcalt S, Ringer JM. Negative symptoms and poor insight as predictors of the similarity between client and therapist ratings of therapeutic alliance in cognitive behavior therapy for patients with schizophrenia. J Nerv Ment Dis. 2011;199(3):191-5. doi: 10.1097/NMD.0b013e31820c73eb. PMID: 21346490.	No drug
578	Park HJ, Kim JW, Lee SK, Kim SK, Park JK, Cho AR, Chung JH, Song JY. Association between the SLC6A12 gene and negative symptoms of schizophrenia in a Korean population. Psychiatry Res. 2011;189(3):478-9. doi: 10.1016/j.psychres.2011.01.023. Epub 2011 Mar 2. PMID: 21367462.	No drug
579	Johnson DP, Penn DL, Fredrickson BL, Kring AM, Meyer PS, Catalino LI, Brantley M. A pilot study of loving-kindness meditation for the negative symptoms of schizophrenia. Schizophr Res. 2011;129(2-3):137-40. doi: 10.1016/j.schres.2011.02.015. Epub 2011 Mar 8. PMID: 21385664.	No drug
580	Simpson EH, Kellendonk C, Ward RD, Richards V, Lipatova O, Fairhurst S, Kandel ER, Balsam PD. Pharmacologic rescue of motivational deficit in an animal model of the negative symptoms of schizophrenia. Biol Psychiatry. 2011;69(10):928-35. doi: 10.1016/j.biopsych.2011.01.012. Epub 2011 Mar 16. PMID: 21414604; PMCID: PMC3170714.	Animal
581	Levkovitz Y, Rabany L, Harel EV, Zangen A. Deep transcranial magnetic stimulation add-on for treatment of negative symptoms and cognitive deficits of schizophrenia: a feasibility study. Int J Neuropsychopharmacol. 2011;14(7):991-6. doi: 10.1017/S1461145711000642. Epub 2011 Apr 28. PMID: 21524336.	No drug
582	Alphs L, Morlock R, Coon C, Cazorla P, Szegedi A, Panagides J. Validation of a 4-item Negative Symptom Assessment (NSA-4): a short, practical clinical tool for the assessment of negative symptoms in schizophrenia. Int J Methods Psychiatr Res. 2011;20(2):e31-7. doi: 10.1002/mpr.339. PMID: 21538654; PMCID: PMC6878310.	No drug
583	Prikryl R. Repetitive transcranial magnetic stimulation and treatment of negative symptoms of schizophrenia. Neuro Endocrinol Lett. 2011;32(2):121-6. PMID: 21552191.	Review
584	Hunter R, Barry S. Negative symptoms and psychosocial functioning in schizophrenia: neglected but important targets for treatment. Eur Psychiatry. 2012;27(6):432-6. doi: 10.1016/j.eurpsy.2011.02.015. Epub 2011 May 23. PMID: 21602034.	No drug
585	Lovisi GM. Do the effects of yoga therapy improve positive and negative symptoms and emotion recognition abilities in antipsychotic-stabilized patients with schizophrenia? Acta Psychiatr Scand. 2011;124(3):234-5; author reply 235-6. doi: 10.1111/j.1600-0447.2011.01727.x. Epub 2011 May 26. PMID: 21615363.	No drug
586	Itoh T, Sumiyoshi T, Higuchi Y, Suzuki M, Kawasaki Y. LORETA analysis of three-dimensional distribution of δ band activity in schizophrenia: relation to negative symptoms. Neurosci Res. 2011;70(4):442-8. doi: 10.1016/j.neures.2011.05.003. Epub 2011 May 27. PMID: 21641943.	No drug
587	Barnes TR, Paton C. Do antidepressants improve negative symptoms in schizophrenia? BMJ. 2011;342:d3371. doi: 10.1136/bmj.d3371. PMID: 21665932.	Opinion
588	Brunelin J, Szekely D, Costes N, Mondino M, Bougerol T, Saoud M, Suaud- Chagny MF, Poulet E, Polosan M. Theta burst stimulation in the negative symptoms of schizophrenia and striatal dopamine release. An iTBS-[11C]raclopride PET case study. Schizophr Res. 2011;131(1-3):264-5. doi: 10.1016/j.schres.2011.05.019. Epub 2011 Jun 12. PMID: 21669513.	Case
589	Daniel DG, Alphs L, Cazorla P, Bartko JJ, Panagides J. Training for assessment of negative symptoms of schizophrenia across languages and cultures: comparison of the NSA-16 with the PANSS Negative Subscale and Negative Symptom factor. Clin Schizophr Relat Psychoses. 2011;5(2):87-94. doi: 10.3371/CSRP.5.2.5. PMID: 21693432.	No drug
590	Couture SM, Blanchard JJ, Bennett ME. Negative expectancy appraisals and defeatist performance beliefs and negative symptoms of schizophrenia. Psychiatry Res. 2011;189(1):43-8. doi: 10.1016/j.psychres.2011.05.032. Epub 2011 Jun 24. PMID: 21704387; PMCID: PMC3156874.	No drug
591	Darbà J, Minoves A, Rojo E, Jimenez F, Rejas J. Efficacy of second- generation-antipsychotics in the treatment of negative symptoms of schizophrenia: a meta-analysis of randomized clinical trials. Rev Psiquiatr Salud Ment. 2011;4(3):126-43. English, Spanish. doi: 10.1016/j.rpsm.2011.02.005. Epub 2011 Jun 14. PMID: 23446193.	Review
592	Ehrlich S, Yendiki A, Greve DN, Manoach DS, Ho BC, White T, Schulz SC, Goff DC, Gollub RL, Holt DJ. Striatal function in relation to negative symptoms in schizophrenia. Psychol Med. 2012;42(2):267-82. doi: 10.1017/S003329171100119X. Epub 2011 Jul 7. PMID: 21733291.	No drug
593	Barr MS, Farzan F, Tran LC, Fitzgerald PB, Daskalakis ZJ. A randomized controlled trial of sequentially bilateral prefrontal cortex repetitive transcranial magnetic stimulation in the treatment of negative symptoms in schizophrenia. Brain Stimul. 2012;5(3):337-346. doi: 10.1016/j.brs.2011.06.003. Epub 2011 Jul 13. PMID: 21782542.	No drug
594	Xiao S-F, Xue H-B, Li X, Chen C, Li G-J, Yuan C-M, Zhang M-Y. A double-blind, placebo-controlled study of traditional Chinese medicine sarsasapogenin added to risperidone in patients with negative symptoms dominated schizophrenia. Neurosci Bull. 2011;27(4):258-68. doi: 10.1007/s12264-011-1417-6. PMID: 21788997; PMCID: PMC5560307.	**Included**
595	Erickson M, Jaafari N, Lysaker P. Insight and negative symptoms as predictors of functioning in a work setting in patients with schizophrenia. Psychiatry Res. 2011;189(2):161-5. doi: 10.1016/j.psychres.2011.06.019. Epub 2011 Aug 2. PMID: 21813183.	No drug
596	Kaiser S, Heekeren K, Simon JJ. The negative symptoms of schizophrenia: category or continuum? Psychopathology. 2011;44(6):345-53. doi: 10.1159/000325912. Epub 2011 Aug 17. PMID: 21847001.	Review
597	Coppola M, Mondola R. Potential action of betel alkaloids on positive and negative symptoms of schizophrenia: a review. Nord J Psychiatry. 2012;66(2):73-8. doi: 10.3109/08039488.2011.605172. Epub 2011 Aug 23. PMID: 21859398.	Review
598	Klingberg S, Wölwer W, Engel C, Wittorf A, Herrlich J, Meisner C, Buchkremer G, Wiedemann G. Negative symptoms of schizophrenia as primary target of cognitive behavioral therapy: results of the randomized clinical TONES study. Schizophr Bull. 2011;37(Suppl 2):S98-110. doi: 10.1093/schbul/sbr073. PMID: 21860053; PMCID: PMC3160126.	No drug
599	Niitsu T, Shirayama Y, Matsuzawa D, Hasegawa T, Kanahara N, Hashimoto T, Shiraishi T, Shiina A, Fukami G, Fujisaki M, Watanabe H, Nakazato M, Asano M, Kimura S, Hashimoto K, Iyo M. Associations of serum brain-derived neurotrophic factor with cognitive impairments and negative symptoms in schizophrenia. Prog Neuropsychopharmacol Biol Psychiatry. 2011;35(8):1836-40. doi: 10.1016/j.pnpbp.2011.09.004. Epub 2011 Sep 10. PMID: 21930178.	No drug
600	Phan SV, Kreys TJ. Adjunct mirtazapine for negative symptoms of schizophrenia. Pharmacotherapy. 2011;31(10):1017-30. doi: 10.1592/phco.31.10.1017. PMID: 21950644.	Review
601	Chang WC, Hui CL, Tang JY, Wong GH, Lam MM, Chan SK, Chen EY. Persistent negative symptoms in first-episode schizophrenia: a prospective three-year follow-up study. Schizophr Res. 2011;133(1-3):22-8. doi: 10.1016/j.schres.2011.09.006. Epub 2011 Oct 2. PMID: 21968080.	No drug
602	Turkington D, Morrison AP. Cognitive therapy for negative symptoms of schizophrenia. Arch Gen Psychiatry. 2012;69(2):119-20. doi: 10.1001/archgenpsychiatry.2011.141. Epub 2011 Oct 3. PMID: 21969421.	No drug
603	Bell MD, Corbera S, Johannesen JK, Fiszdon JM, Wexler BE. Social cognitive impairments and negative symptoms in schizophrenia: are there subtypes with distinct functional correlates? Schizophr Bull. 2013;39(1):186-96. doi: 10.1093/schbul/sbr125. Epub 2011 Oct 5. PMID: 21976710; PMCID: PMC3523908.	No drug
604	Stauffer VL, Song G, Kinon BJ, Ascher-Svanum H, Chen L, Feldman PD, Conley RR. Responses to antipsychotic therapy among patients with schizophrenia or schizoaffective disorder and either predominant or prominent negative symptoms. Schizophr Res. 2012;134(2-3):195-201. doi: 10.1016/j.schres.2011.09.028. Epub 2011 Oct 21. PMID: 22019076.	Unfocused

605	Roffman JL, Brohawn DG, Nitenson AZ, Macklin EA, Smoller JW, Goff DC. Genetic variation throughout the folate metabolic pathway influences negative symptom severity in schizophrenia. Schizophr Bull. 2013;39(2):330-8. doi: 10.1093/schbul/sbr150. Epub 2011 Oct 20. PMID: 22021659; PMCID: PMC3576161.	No drug
606	Oorschot M, Lataster T, Thewissen V, Lardinois M, Wichers M, van Os J, Delespaul P, Myin-Germeys I. Emotional experience in negative symptoms of schizophrenia—no evidence for a generalized hedonic deficit. Schizophr Bull. 2013;39(1):217-25. doi: 10.1093/schbul/sbr137. Epub 2011 Oct 20. PMID: 22021660; PMCID: PMC3523912.	No drug
607	Luckhaus C, Henning U, Ferrea S, Musso F, Mobascher A, Winterer G. Nicotinic acetylcholine receptor expression on B-lymphoblasts of healthy versus schizophrenic subjects stratified for smoking: [3H]-nicotine binding is decreased in schizophrenia and correlates with negative symptoms. J Neural Transm (Vienna). 2012;119(5):587-95. doi: 10.1007/s00702-011-0743-1. Epub 2011 Dec 11. PMID: 22160487.	No drugs
608	Kane JM, Yang R, Youakim JM. Adjunctive armodafinil for negative symptoms in adults with schizophrenia: a double-blind, placebo-controlled study. Schizophr Res. 2012;135(1-3):116-22. doi: 10.1016/j.schres.2011.11.006. Epub 2011 Dec 16. PMID: 22178084.	**Included**
609	Mori T. [Case of schizophrenia in which depressive and negative symptoms relapsed on switching from oral risperidone to risperidone long-acting injection]. Seishin Shinkeigaku Zasshi. 2011;113(10):977-82. Japanese. PMID: 22187885.	Case
610	Buchanan RW, Panagides J, Zhao J, Phiri P, den Hollander W, Ha X, Kouassi A, Alphs L, Schooler N, Szegedi A, Cazorla P. Asenapine versus olanzapine in people with persistent negative symptoms of schizophrenia. J Clin Psychopharmacol. 2012;32(1):36-45. doi: 10.1097/JCP.0b013e31823f880a. PMID: 22198451.	**Included**
611	Chatterjee M, Jaiswal M, Palit G. Comparative evaluation of forced swim test and tail suspension test as models of negative symptom of schizophrenia in rodents. ISRN Psychiatry. 2012;2012:595141. doi: 10.5402/2012/595141. PMID: 23738205; PMCID: PMC3658575.	Animal
612	Hovington CL, Lepage M. Neurocognition and neuroimaging of persistent negative symptoms of schizophrenia. Expert Rev Neurother. 2012;12(1):53-69. doi: 10.1586/ern.11.173. PMID: 22243045.	No drug
613	Xiao S, Xue H, Li G, Yuan C, Li X, Chen C, Wu HZ, Mitchell P, Zhang M. Therapeutic effects of cerebrolysin added to risperidone in patients with schizophrenia dominated by negative symptoms. Aust N Z J Psychiatry. 2012;46(2):153-60. doi: 10.1177/0004867411433213. PMID: 22311531.	**Included**
614	Rabinowitz J, Levine SZ, Garibaldi G, Bugarski-Kirola D, Berardo CG, Kapur S. Negative symptoms have greater impact on functioning than positive symptoms in schizophrenia: analysis of CATIE data. Schizophr Res. 2012;137(1-3):147-50. doi: 10.1016/j.schres.2012.01.015. Epub 2012 Feb 6. PMID: 22316568.	Unfocused
615	Ojeda N, Sánchez P, Peña J, Elizagárate E, Yoller AB, Gutiérrez-Fraile M, Ezcurra J, Napal O. An explanatory model of quality of life in schizophrenia: the role of processing speed and negative symptoms. Actas Esp Psiquiatr. 2012;40(1):10-8. Epub 2012 Jan 1. PMID: 22344491.	No drug
616	Singam AP, Mamarde A, Behere PB. A single blind comparative clinical study of the effects of chlorpromazine and risperidone on positive and negative symptoms in patients of schizophrenia. Indian J Psychol Med. 2011;33(2):134-40. doi: 10.4103/0253-7176.92061. PMID: 22345836; PMCID: PMC3271486.	Open
617	Li WJ, Kou CG, Yu Y, Sun S, Zhang X, Kosten TR, Zhang XY. Association of catechol-O-methyltransferase gene polymorphisms with schizophrenia and negative symptoms in a Chinese population. Am J Med Genet B Neuropsychiatr Genet. 2012;159B(4):370-5. doi: 10.1002/ajmg.b.32038. Epub 2012 Feb 21. PMID: 22354729; PMCID: PMC4190670.	No drug
618	Kendall T. Treating negative symptoms of schizophrenia. BMJ. 2012;344:e664. doi: 10.1136/bmj.e664. PMID: 22374931.	Opinion
619	Kang WS, Park JK, Park HJ, Kim SK, Cho AR, Lee SM, Kim JW, Song JY. Association between catechol-O-methyltransferase Val158Met polymorphism and negative symptoms of schizophrenia in the Korean population. Psychiatry Res. 2012;198(2):338-9. doi: 10.1016/j.psychres.2011.12.048. Epub 2012 Mar 11. PMID: 22414663.	No drug
620	Ward RD, Simpson EH, Richards VL, Deo G, Taylor K, Glendinning JI, Kandel ER, Balsam PD. Dissociation of hedonic reaction to reward and incentive motivation in an animal model of the negative symptoms of schizophrenia. Neuropsychopharmacology. 2012;37(7):1699-707. doi: 10.1038/npp.2012.15. Epub 2012 Mar 14. PMID: 22414818; PMCID: PMC3358738.	Animal
621	Lyne JP, Turner N, Clarke M. Treat negative symptoms of schizophrenia early on. BMJ. 2012;344:e2297. doi: 10.1136/bmj.e2297. PMID: 22453888.	Opinion
622	Lysaker PH, Erikson M, Macapagal KR, Tunze C, Gilmore E, Ringer JM. Development of personal narratives as a mediator of the impact of deficits in social cognition and social withdrawal on negative symptoms in schizophrenia. J Nerv Ment Dis. 2012;200(4):290-5. doi: 10.1097/NMD.0b013e31824cb0f4. PMID: 22456581.	No drug
623	Prikryl R, Mikl M, Prikrylova Kucerová H, Ustohal L, Kasparek T, Marecek R, Vrzalova M, Ceskova E, Vanicek J. Does repetitive transcranial magnetic stimulation have a positive effect on working memory and neuronal activation in treatment of negative symptoms of schizophrenia? Neuro Endocrinol Lett. 2012;33(1):90-7. PMID: 22467118.	No drug
624	Levine SZ, Leucht S. Delayed- and early-onset hypotheses of antipsychotic drug action in the negative symptoms of schizophrenia. Eur Neuropsychopharmacol. 2012;22(11):812-7. doi: 10.1016/j.euroneuro.2012.03.001. Epub 2012 Apr 14. PMID: 22507686.	Overlap
625	Chaudhry IB, Hallak J, Husain N, Minhas F, Stirling J, Richardson P, Dursun S, Dunn G, Deakin B. Minocycline benefits negative symptoms in early schizophrenia: a randomised double-blind placebo-controlled clinical trial in patients on standard treatment. J Psychopharmacol. 2012;26(9):1185-93. doi: 10.1177/0269881112444941. Epub 2012 Apr 23. PMID: 22526685.	**Included**
626	Takase K, Yamamoto Y, Yagami T. Maternal deprivation in the middle of a stress hyporesponsive period decreases hippocampal calcineurin expression and causes abnormal social and cognitive behaviours in adult male Wistar rats: relevance to negative symptoms of schizophrenia. Behav Brain Res. 2012;232(1):306-15. doi: 10.1016/j.bbr.2012.04.016. Epub 2012 Apr 21. PMID: 22543011.	Animal
627	Sanz JC, Gómez V, Vargas ML, Marín JJ. Dimensions of attention impairment and negative symptoms in schizophrenia: a multidimensional approach using the conners continuous performance test in a Spanish population. Cogn Behav Neurol. 2012;25(2):63-71. doi: 10.1097/WNN.0b013e318255feaf. PMID: 22543264.	No drug
628	Suazo V, Díez Á, Martín C, Ballesteros A, Casado P, Martín-Loeches M, Molina V. Elevated noise power in gamma band related to negative symptoms and memory deficit in schizophrenia. Prog Neuropsychopharmacol Biol Psychiatry. 2012;38(2):270-5. doi: 10.1016/j.pnpbp.2012.04.010. Epub 2012 Apr 21. PMID: 22549114.	No drug
629	Chindo BA, Adzu B, Yahaya TA, Gamaniel KS. Ketamine-enhanced immobility in forced swim test: a possible animal model for the negative symptoms of schizophrenia. Prog Neuropsychopharmacol Biol Psychiatry. 2012;38(2):310-6. doi: 10.1016/j.pnpbp.2012.04.018. Epub 2012 Apr 26. PMID: 22561603.	Animal
630	Pogarell O, Koch W, Karch S, Dehning S, Müller N, Tatsch K, Poepperl G, Möller HJ. Dopaminergic neurotransmission in patients with schizophrenia in relation to positive and negative symptoms. Pharmacopsychiatry. 2012;45(Suppl 1):S36-41. doi: 10.1055/s-0032-1306313. Epub 2012 May 7. PMID: 22565233.	Unfocused
631	Galderisi S, Mucci A, Bitter I, Libiger J, Bucci P, Fleischhacker WW, Kahn RS; Eufest Study Group. Persistent negative symptoms in first episode patients with schizophrenia: results from the European First Episode Schizophrenia Trial. Eur Neuropsychopharmacol. 2013;23(3):196-204. doi: 10.1016/j.euroneuro.2012.04.019. Epub 2012 May 28. PMID: 22647933.	No drug
632	Brébion G, Ohlsen RI, Bressan RA, David AS. Source memory errors in schizophrenia, hallucinations and negative symptoms: a synthesis of research findings. Psychol Med. 2012;42(12):2543-54. doi: 10.1017/S003329171200075X. Epub 2012 Apr 27. PMID: 22716666.	Review
633	Green MJ, Cairns MJ, Wu J, Dragovic M, Jablensky A, Tooney PA, Scott RJ, Carr VJ; Australian Schizophrenia Research Bank. Genome-wide supported variant MIR137 and severe negative symptoms predict membership of an impaired cognitive subtype of schizophrenia. Mol Psychiatry. 2013;18(7):774-80. doi: 10.1038/mp.2012.84. Epub 2012 Jun 26. Erratum in: Mol Psychiatry. 2013;18(7):843. PMID: 22733126.	No drug

634	Sasayama D, Hattori K, Teraishi T, Hori H, Ota M, Yoshida S, Arima K, Higuchi T, Amano N, Kunugi H. Negative correlation between cerebrospinal fluid oxytocin levels and negative symptoms of male patients with schizophrenia. Schizophr Res. 2012;139(1-3):201-6. doi: 10.1016/j.schres.2012.06.016. Epub 2012 Jun 27. PMID: 22742979.	No drug
635	Cazorla P, Mackle M, Zhao J, Ha X, Szegedi A. Safety and tolerability of switching to asenapine from other antipsychotic agents: pooled results from two randomized multicenter trials in stable patients with persistent negative symptoms in schizophrenia. Neuropsychiatr Dis Treat. 2012;8:247-57. doi: 10.2147/NDT.S29891. Epub 2012 Jun 15. PMID: 22745558; PMCID: PMC3383320.	Unfocused
636	Klingberg S, Herrlich J, Wiedemann G, Wölwer W, Meisner C, Engel C, Jakobi- Malterre UE, Buchkremer G, Wittorf A. Adverse effects of cognitive behavioral therapy and cognitive remediation in schizophrenia: results of the treatment of negative symptoms study. J Nerv Ment Dis. 2012;200(7):569-76. doi: 10.1097/NMD.0b013e31825bfa1d. PMID: 22759932.	No drug
637	Bosker FJ, Gladkevich AV, Pietersen CY, Kooi KA, Bakker PL, Gerbens F, den Boer JA, Korf J, te Meerman G. Comparison of brain and blood gene expression in an animal model of negative symptoms in schizophrenia. Prog Neuropsychopharmacol Biol Psychiatry. 2012;38(2):142-8. doi: 10.1016/j.pnpbp.2012.03.003. Epub 2012 Mar 15. PMID: 22763037.	Animal
638	Strauss GP, Sandt AR, Catalano LT, Allen DN. Negative symptoms and depression predict lower psychological well-being in individuals with schizophrenia. Compr Psychiatry. 2012;53(8):1137-44. doi: 10.1016/j.comppsych.2012.05.009. Epub 2012 Jul 6. PMID: 22770716.	No drug
639	Michalopoulou PG, Azim A, Tracy D, Shergill SS. Ropinirole as an effective adjunctive treatment for clozapine-resistant negative symptoms in simple schizophrenia: a case report. J Clin Psychopharmacol. 2012;32(5):719-20. doi: 10.1097/JCP.0b013e318267062c. PMID: 22926612.	Case
640	Wegelius A, Pankakoski M, Lehto U, Suokas J, Häkkinen L, Tuulio-Henriksson A, Lönnqvist J, Paunio T, Suvisaari J. An association between both low and high birth weight and increased disorganized and negative symptom severity in schizophrenia and other psychoses. Psychiatry Res. 2013;205(1-2):18-24. doi: 10.1016/j.psychres.2012.08.026. Epub 2012 Sep 15. PMID: 22985549.	No drug
641	Rabany L, Weiser M, Levkovitz Y. Guilt and depression: two different factors in individuals with negative symptoms of schizophrenia. Eur Psychiatry. 2013;28(6):327-31. doi: 10.1016/j.eurpsy.2012.02.008. Epub 2012 Sep 27. PMID: 23021933.	No drug
642	McCormick BP, Snethen G, Lysaker PH. Emotional episodes in the everyday lives of people with schizophrenia: the role of intrinsic motivation and negative symptoms. Schizophr Res. 2012;142(1-3):46-51. doi: 10.1016/j.schres.2012.09.002. Epub 2012 Sep 28. PMID: 23022211.	No drug
643	Covington MA, Lunden SL, Cristofaro SL, Wan CR, Bailey CT, Broussard B, Fogarty R, Johnson S, Zhang S, Compton MT. Phonetic measures of reduced tongue movement correlate with negative symptom severity in hospitalized patients with first-episode schizophrenia-spectrum disorders. Schizophr Res. 2012;142(1-3):93-5. doi: 10.1016/j.schres.2012.10.005. Epub 2012 Oct 24. PMID: 23102940; PMCID: PMC3523277.	No drug
644	Cruz BF, de Resende CB, Abreu MN, Rocha FL, Teixeira AL, Keefe RS, Salgado JV. How specific are negative symptoms and cognitive impairment in schizophrenia? An analysis of PANSS and SCoRS. Cogn Neuropsychiatry. 2013;18(3):243-51. doi: 10.1080/13546805.2012.730995. Epub 2012 Nov 12. PMID: 23145794.	No drug
645	Bagney A, Rodriguez-Jimenez R, Martinez-Gras I, Sanchez-Morla EM, Santos JL, Jimenez-Arriero MA, Lobo A, McGorry PD, Palomo T. Negative symptoms and executive function in schizophrenia: does their relationship change with illness duration? Psychopathology. 2013;46(4):241-8. doi: 10.1159/000342345. Epub 2012 Nov 9. PMID: 23147471.	No drug
646	Nejad AB, Madsen KH, Ebdrup BH, Siebner HR, Rasmussen H, Aggernæs B, Glenthøj BY, Baaré WF. Neural markers of negative symptom outcomes in distributed working memory brain activity of antipsychotic-naive schizophrenia patients. Int J Neuropsychopharmacol. 2013;16(6):1195-204. doi: 10.1017/S1461145712001253. Epub 2012 Nov 20. PMID: 23164479.	No drug
647	Chang WC, Hui CL, Tang JY, Wong GH, Chan SK, Lee EH, Chen EY. Impacts of duration of untreated psychosis on cognition and negative symptoms in first-episode schizophrenia: a 3-year prospective follow-up study. Psychol Med. 2013;43(9):1883-93. doi: 10.1017/S0033291712002838. Epub 2012 Dec 7. PMID: 23217676.	No drug
648	Hill K, Startup M. The relationship between internalized stigma, negative symptoms and social functioning in schizophrenia: the mediating role of self- efficacy. Psychiatry Res. 2013;206(2-3):151-7. doi: 10.1016/j.psychres.2012.09.056. Epub 2012 Dec 4. PMID: 23218915.	No drug
649	Ventura J, Tom SR, Jetton C, Kern RS. Memory functioning and negative symptoms as differential predictors of social problem solving skills in schizophrenia. Schizophr Res. 2013;143(2-3):307-11. doi: 10.1016/j.schres.2012.10.043. Epub 2012 Dec 9. PMID: 23235142; PMCID: PMC4104115.	No drug
650	Prikryl R, Kucerova HP. Can repetitive transcranial magnetic stimulation be considered effective treatment option for negative symptoms of schizophrenia? J ECT. 2013;29(1):67-74. doi: 10.1097/YCT.0b013e318270295f. PMID: 23303418.	No drug
651	Ho RT, Au Yeung FS, Lo PH, Law KY, Wong KO, Cheung IK, Ng SM. Tai-chi for residential patients with schizophrenia on movement coordination, negative symptoms, and functioning: a pilot randomized controlled trial. Evid Based Complement Alternat Med. 2012;2012:923925. doi: 10.1155/2012/923925. Epub 2012 Nov 24. PMID: 23304224; PMCID: PMC3524789.	No drug
652	Priebe S, Savill M, Reininghaus U, Wykes T, Bentall R, Lauber C, McCrone P, Röhricht F, Eldridge S. Effectiveness and cost-effectiveness of body psychotherapy in the treatment of negative symptoms of schizophrenia--a multi-centre randomised controlled trial. BMC Psychiatry. 2013;13:26. doi: 10.1186/1471-244X-13-26. PMID: 23317474; PMCID: PMC3556155.	No drug
653	Palomino A, González-Pinto A, Martinez-Cengotitabengoa M, Ruiz de Azua S, Alberich S, Mosquera F, Matute C. Relationship between negative symptoms and plasma levels of insulin-like growth factor 1 in first-episode schizophrenia and bipolar disorder patients. Prog Neuropsychopharmacol Biol Psychiatry. 2013;44:29-33. doi: 10.1016/j.pnpbp.2013.01.008. Epub 2013 Jan 18. PMID: 23337034.	No drug
654	Cohen CI, Natarajan N, Araujo M, Solanki D. Prevalence of negative symptoms and associated factors in older adults with schizophrenia spectrum disorder. Am J Geriatr Psychiatry. 2013;21(2):100-7. doi: 10.1016/j.jagp.2012.10.009. Epub 2013 Jan 22. PMID: 23343483.	No drug
655	Chien YL, Hwu HG, Fann CS, Chang CC, Tsuang MT, Liu CM. DRD2 haplotype associated with negative symptoms and sustained attention deficits in Han Chinese with schizophrenia in Taiwan. J Hum Genet. 2013;58(4):229-32. doi: 10.1038/jhg.2012.157. Epub 2013 Jan 31. PMID: 23364393.	No drug
656	Khodaie-Ardakani MR, Seddighi S, Modabbernia A, Rezaei F, Salehi B, Ashrafi M, Shams-Alizadeh N, Mohammad-Karimi M, Esfandiari GR, Hajiaghaee R, Akhondzadeh S. Granisetron as an add-on to risperidone for treatment of negative symptoms in patients with stable schizophrenia: randomized double-blind placebo-controlled study. J Psychiatr Res. 2013;47(4):472-8. doi: 10.1016/j.jpsychires.2013.01.011. Epub 2013 Jan 30. PMID: 23375406.	**Included**
657	Kurtz MM, Olfson RH, Rose J. Self-efficacy and functional status in schizophrenia: relationship to insight, cognition and negative symptoms. Schizophr Res. 2013;145(1-3):69-74. doi: 10.1016/j.schres.2012.12.030. Epub 2013 Feb 1. PMID: 23375941; PMCID: PMC5180360.	No drug
658	Barch DM. The CAINS: theoretical and practical advances in the assessment of negative symptoms in schizophrenia. Am J Psychiatry. 2013;170(2):133-5. doi: 10.1176/appi.ajp.2012.12101329. PMID: 23377630.	No drug
659	Xu C, Aragam N, Li X, Villla EC, Wang L, Briones D, Petty L, Posada Y, Arana TB, Cruz G, Mao C, Camarillo C, Su BB, Escamilla MA, Wang K. BCL9 and C9orf5 are associated with negative symptoms in schizophrenia: meta-analysis of two genome-wide association studies. PLoS One. 2013;8(1):e51674. doi: 10.1371/journal.pone.0051674. Epub 2013 Jan 29. PMID: 23382809; PMCID: PMC3558516.	No drug
660	Gold JM, Strauss GP, Waltz JA, Robinson BM, Brown JK, Frank MJ. Negative symptoms of schizophrenia are associated with abnormal effort-cost computations. Biol Psychiatry. 2013;74(2):130-6. doi: 10.1016/j.biopsych.2012.12.022. Epub 2013 Feb 7. PMID: 23394903; PMCID: PMC3703817.	No drug
661	Marder SR. Clinician perceptions, expectations, and management of negative symptoms in schizophrenia. J Clin Psychiatry. 2013;74(1):e01. doi: 10.4088/JCP.12045tx4c. PMID: 23419229.	Opinion

662	Boutros NN, Mucci A, Diwadkar V, Tandon R. Negative symptoms in schizophrenia. Clin Schizophr Relat Psychoses. 2014;8(1):28-35B. doi: 10.3371/CSRP.BOMU.012513. PMID: 23428787.	No drug
663	Wang YM. A commentary on DRD2 haplotype associated with negative symptoms and sustained attention deficits in Han Chinese with schizophrenia in Taiwan. J Hum Genet. 2013;58(4):182. doi: 10.1038/jhg.2013.13. Epub 2013 Feb 28. PMID: 23446888.	No drug
664	Strauss GP, Horan WP, Kirkpatrick B, Fischer BA, Keller WR, Miski P, Buchanan RW, Green MF, Carpenter WT Jr. Deconstructing negative symptoms of schizophrenia: avolition-apathy and diminished expression clusters predict clinical presentation and functional outcome. J Psychiatr Res. 2013;47(6):783-90. doi: 10.1016/j.jpsychires.2013.01.015. Epub 2013 Feb 27. PMID: 23453820; PMCID: PMC3686506.	No drug
665	Chang WC, Tang JY, Hui CL, Wong GH, Chan SK, Lee EH, Chen EY. The relationship of early premorbid adjustment with negative symptoms and cognitive functions in first-episode schizophrenia: a prospective three-year follow-up study. Psychiatry Res. 2013;209(3):353-60. doi: 10.1016/j.psychres.2013.02.014. Epub 2013 Mar 7. PMID: 23473654.	No drug
666	Lin CH, Huang CL, Chang YC, Chen PW, Lin CY, Tsai GE, Lane HY. Clinical symptoms, mainly negative symptoms, mediate the influence of neurocognition and social cognition on functional outcome of schizophrenia. Schizophr Res. 2013;146(1-3):231-7. doi: 10.1016/j.schres.2013.02.009. Epub 2013 Mar 9. PMID: 23478155.	No drug
667	Jhamnani K, Shivakumar V, Kalmady S, Rao NP, Venkatasubramanian G. Successful use of add-on minocycline for treatment of persistent negative symptoms in schizophrenia. J Neuropsychiatry Clin Neurosci. 2013;25(1):E06-7. doi: 10.1176/appi.neuropsych.11120376. PMID: 23487204.	Case
668	Vidal C, Reese C, Fischer BA, Chiapelli J, Himelhoch S. Meta-analysis of efficacy of mirtazapine as an adjunctive treatment of negative symptoms in schizophrenia. Clin Schizophr Relat Psychoses. 2015;9(2):88-95. doi: 10.3371/CSRP.VIRE.030813. Epub 2013 Mar 14. PMID: 23491969.	Review
669	Noroozian M, Ghasemi S, Hosseini SM, Modabbernia A, Khodaie-Ardakani MR, Mirshafiee O, Farokhnia M, Tajdini M, Rezaei F, Salehi B, Ashrafi M, Yekehtaz H, Tabrizi M, Akhondzadeh S. A placebo-controlled study of tropisetron added to risperidone for the treatment of negative symptoms in chronic and stable schizophrenia. Psychopharmacology (Berl). 2013;228(4):595-602. doi: 10.1007/s00213-013-3064-2. Epub 2013 Mar 21. PMID: 23515583.	**Included**
670	Palm U, Hasan A, Keeser D, Falkai P, Padberg F. Transcranial random noise stimulation for the treatment of negative symptoms in schizophrenia. Schizophr Res. 2013;146(1-3):372-3. doi: 10.1016/j.schres.2013.03.003. Epub 2013 Mar 19. PMID: 23517664.	No drug
671	Pinacho R, Villalmanzo N, Roca M, Iniesta R, Monje A, Haro JM, Meana JJ, Ferrer I, Gill G, Ramos B. Analysis of Sp transcription factors in the postmortem brain of chronic schizophrenia: a pilot study of relationship to negative symptoms. J Psychiatr Res. 2013;47(7):926-34. doi: 10.1016/j.jpsychires.2013.03.004. Epub 2013 Mar 27. PMID: 23540600.	*Post-mortem*
672	Noh K, Shin KS, Shin D, Hwang JY, Kim JS, Jang JH, Chung CK, Kwon JS, Cho KH. Impaired coupling of local and global functional feedbacks underlies abnormal synchronization and negative symptoms of schizophrenia. BMC Syst Biol. 2013;7:30. doi: 10.1186/1752-0509-7-30. PMID: 23575114; PMCID: PMC3639871.	No drug
673	Galderisi S, Bucci P, Mucci A, Kirkpatrick B, Pini S, Rossi A, Vita A, Maj M. Categorical and dimensional approaches to negative symptoms of schizophrenia: focus on long-term stability and functional outcome. Schizophr Res. 2013;147(1):157-162. doi: 10.1016/j.schres.2013.03.020 Epub 2013 Apr 19. PMID: 23608244.	No drug
674	Rezaei F, Mohammad-Karimi M, Seddighi S, Modabbernia A, Ashrafi M, Salehi B, Hammidi S, Motasami H, Hajiaghaee R, Tabrizi M, Akhondzadeh S. Memantine add- on to risperidone for treatment of negative symptoms in patients with stable schizophrenia: randomized, double-blind, placebo-controlled study. J Clin Psychopharmacol. 2013;33(3):336-42. doi: 10.1097/JCP.0b013e31828b50a7. PMID: 23609382.	**Included**
675	Lindenmayer JP, Nasrallah H, Pucci M, James S, Citrome L. A systematic review of psychostimulant treatment of negative symptoms of schizophrenia: challenges and therapeutic opportunities. Schizophr Res. 2013;147(2-3):241-52. doi: 10.1016/j.schres.2013.03.019. Epub 2013 Apr 22. PMID: 23619055.	Review
676	Ceccarini J, De Hert M, Van Winkel R, Peuskens J, Bormans G, Kranaster L, Enning F, Koethe D, Leweke FM, Van Laere K. Increased ventra striatal CB1 receptor binding is related to negative symptoms in drug-free patients with schizophrenia. Neuroimage. 2013;79:304-12. doi: 10.1016/j.neuroimage.2013.04.052. Epub 2013 Apr 25. PMID: 23624489.	No drug
677	Dhandapani A, Narayanaswamy JC, Venkatasubramanian G. Adjuvant raloxifene treatment for negative symptoms of schizophrenia. Asian J Psychiatr. 2013;6(3):254-5. doi: 10.1016/j.ajp.2012.12.014. Epub 2013 Feb 20. PMID: 23642987.	Case
678	Harvey PD. Assessment of everyday functioning in schizophrenia: implications for treatments aimed at negative symptoms. Schizophr Res. 2013;150(2-3):353-5. doi: 10.1016/j.schres.2013.04.022. Epub 2013 May 11. PMID: 23668973; PMCID: PMC3825780.	No drug
679	Maeda T, Takahata K, Muramatsu T, Okimura T, Koreki A, Iwashita S, Mimura M, Kato M. Reduced sense of agency in chronic schizophrenia with predominant negative symptoms. Psychiatry Res. 2013;209(3):386-92. doi: 10.1016/j.psychres.2013.04.017. Epub 2013 May 14. PMID: 23680465.	No drug
680	Bervoets C, Docx L, Sabbe B, Vermeylen S, Van Den Bossche MJ, Morsel A, Morrens M. The nature of the relationship of psychomotor slowing with negative symptomatology in schizophrenia. Cogn Neuropsychiatry. 2014;19(1):36-46. doi: 10.1080/13546805.2013.779578. Epub 2013 Jun 3. PMID: 23725330.	No drug
681	Manoliu A, Riedl V, Doll A, Bäuml JG, Mühlau M, Schwerthöffer D, Scherr M, Zimmer C, Förstl H, Bäuml J, Wohlschläger AM, Koch K, Sorg C. Insular dysfunction reflects altered between-network connectivity and severity of negative symptoms in schizophrenia during psychotic remission. Front Hum Neurosci. 2013;7:216. doi: 10.3389/fnhum.2013.00216. PMID: 23730284; PMCID: PMC3657709.	No drug
682	Goff DC. Future perspectives on the treatment of cognitive deficits and negative symptoms in schizophrenia. World Psychiatry. 2013;12(2):99-107. doi: 10.1002/wps.20026. PMID: 23737409; PMCID: PMC3683252.	Opinion
683	Lasser RA, Dirks B, Nasrallah H, Kirsch C, Gao J, Pucci ML, Knesevich MA, Lindenmayer JP. Adjunctive lisdexamfetamine dimesylate therapy in adult outpatients with predominant negative symptoms of schizophrenia: open-label and randomized-withdrawal phases. Neuropsychopharmacology. 2013;38(11):2140-9. doi: 10.1038/npp.2013.111. Epub 2013 May 8. PMID: 23756608; PMCID: PMC3773663.	Open
684	Stathopoulou A, Beratis IN, Beratis S. Prenatal tobacco smoke exposure, risk of schizophrenia, and severity of positive/negative symptoms. Schizophr Res. 2013;148(1-3):105-10. doi: 10.1016/j.schres.2013.04.031. Epub 2013 Jun 13. PMID: 23768812.	No drug
685	Hanks AN, Dlugolenski K, Hughes ZA, Seymour PA, Majchrzak MJ. Pharmacological disruption of mouse social approach behavior: relevance to negative symptoms of schizophrenia. Behav Brain Res. 2013;252:405-14. doi: 10.1016/j.bbr.2013.06.017. Epub 2013 Jun 25. PMID: 23806621.	Animal
686	Prikryl R, Ustohal L, Prikrylova Kucerova H, Kasparek T, Venclikova S, Vrzalova M, Ceskova E. A detailed analysis of the effect of repetitive transcranial magnetic stimulation on negative symptoms of schizophrenia: a double-blind trial. Schizophr Res. 2013;149(1-3):167-73. doi: 10.1016/j.schres.2013.06.015. Epub 2013 Jun 25. PMID: 23810122.	No drug
687	Rabinowitz J, Werbeloff N, Caers I, Mandel FS, Stauffer V, Menard F, Kinon BJ, Kapur S. Negative symptoms in schizophrenia—the remarkable impact of inclusion definitions in clinical trials and their consequences. Schizophr Res. 2013;150(2-3):334-8. doi: 10.1016/j.schres.2013.06.023. Epub 2013 Jun 29. PMID: 23815975.	No drug
688	Vahia IV, Lanouette NM, Golshan S, Fellows I, Mohamed S, Kasckow JW, Zisook S. Adding antidepressants to antipsychotics for treatment of subsyndromal depressive symptoms in schizophrenia: Impact on positive and negative symptoms. Indian J Psychiatry. 2013;55(2):144-8. doi: 10.4103/0019-5545.111452. PMID: 23825848; PMCID: PMC3696237.	**Included**
689	Kane JM. Tools to assess negative symptoms in schizophrenia. J Clin Psychiatry. 2013;74(6):e12. doi: 10.4088/JCP.12045tx2c. PMID: 23842020.	No drug

690	Hinkelmann K, Yassouridis A, Kellner M, Jahn H, Wiedemann K, Raedler TJ. No effects of antidepressants on negative symptoms in schizophrenia. J Clin Psychopharmacol. 2013;33(5):686-90. doi: 10.1097/JCP.0b013e3182971e68. PMID: 23857309.	**Included**
691	Schrank B, Amering M, Hay AG, Weber M, Sibitz I. Insight, positive and negative symptoms, hope, depression and self-stigma: a comprehensive model of mutual influences in schizophrenia spectrum disorders. Epidemiol Psychiatr Sci. 2014;23(3):271-9. doi: 10.1017/S2045796013000322. Epub 2013 Jul 24. PMID: 23883668; PMCID: PMC6998353.	No drug
692	Kim DW, Shim M, Kim JI, Im CH, Lee SH. Source activation of P300 correlates with negative symptom severity in patients with schizophrenia. Brain Topogr. 2014;27(2):307-17. doi: 10.1007/s10548-013-0306-x. Epub 2013 Jul 30. PMID: 23897409.	No drug
693	Silver H, Bilker WB. Add-on fluvoxamine and negative symptoms of schizophrenia: analysis of data from augmentation studies in a single center. J Clin Psychopharmacol. 2013;33(5):710-1. doi: 10.1097/JCP.0b013e3182983d42. PMID: 23899641.	Review
694	Lutgens D, Lepage M, Manchanda R, Malla A. Persistent negative symptoms in schizophrenia: survey of Canadian psychiatrists. Int Psychiatry. 2013;10(3):69-72. PMID: 31507741; PMCID: PMC6735122.	Opinion
695	Khan A, Lindenmayer JP, Opler M, Yavorsky C, Rothman B, Lucic L. A new Integrated Negative Symptom structure of the Positive and Negative Syndrome Scale (PANSS) in schizophrenia using item response analysis. Schizophr Res. 2013;150(1):185-96. doi: 10.1016/j.schres.2013.07.007. Epub 2013 Jul 30. PMID: 23911252.	No drug
696	Mwansisya TE, Wang Z, Tao H, Zhang H, Hu A, Guo S, Liu Z. The diminished interhemispheric connectivity correlates with negative symptoms and cognitive impairment in first-episode schizophrenia. Schizophr Res. 2013;150(1):144-50. doi: 10.1016/j.schres.2013.07.018. Epub 2013 Aug 3. PMID: 23920057.	No drug
697	Engel M, Fritzsche A, Lincoln TM. Anticipatory pleasure and approach motivation in schizophrenia-like negative symptoms. Psychiatry Res. 2013;210(2):422-6. doi: 10.1016/j.psychres.2013.07.025. Epub 2013 Aug 5. PMID: 23928213.	No drug
698	Boulay D, Ho-Van S, Bergis O, Avenet P, Griebel G. Phencyclidine decreases tickling-induced 50-kHz ultrasound vocalizations in juvenile rats: a putative model of the negative symptoms of schizophrenia? Behav Pharmacol. 2013;24(7):543-51. doi: 10.1097/FBP.0b013e3283654044. PMID: 23928693.	Animal
999	Iasevoli F, Balletta R, Gilardi V, Giordano S, de Bartolomeis A. Tobacco smoking in treatment-resistant schizophrenia patients is associated with impaired cognitive functioning, more severe negative symptoms, and poorer social adjustment. Neuropsychiatr Dis Treat. 2013;9:1113-20. doi: 10.2147/NDT.S47571. Epub 2013 Aug 7. PMID: 23950651; PMCID: PMC3742345.	No drug
700	Elis O, Caponigro JM, Kring AM. Psychosocial treatments for negative symptoms in schizophrenia: current practices and future directions. Clin Psychol Rev. 2013;33(8):914-28. doi: 10.1016/j.cpr.2013.07.001. Epub 2013 Jul 16. PMID: 23988452; PMCID: PMC4092118.	No drug
701	Farreny A, Aguado J, Ochoa S, Haro JM, Usall J. The role of negative symptoms in the context of cognitive remediation for schizophrenia. Schizophr Res. 2013;150(1):58-63. doi: 10.1016/j.schres.2013.08.008. Epub 2013 Aug 29. PMID: 23993864.	No drug
702	Markou A, Salamone JD, Bussey TJ, Mar AC, Brunner D, Gilmour G, Balsam P. Measuring reinforcement learning and motivation constructs in experimental animals: relevance to the negative symptoms of schizophrenia. Neurosci Biobehav Rev. 2013;37(9 Pt B):2149-65. doi: 10.1016/j.neubiorev.2013.08.007. Epub 2013 Aug 28. PMID: 23994273; PMCID: PMC3849135.	Review
703	Farokhnia M, Sabzabadi M, Pourmahmoud H, Khodaie-Ardakani MR, Hosseini SM, Yekehtaz H, Tabrizi M, Rezaei F, Salehi B, Akhondzadeh S. A double-blind, placebo controlled, randomized trial of riluzole as an adjunct to risperidone for treatment of negative symptoms in patients with chronic schizophrenia. Psychopharmacology (Berl). 2014;231(3):533-42. doi: 10.1007/s00213-013-3261-z. Epub 2013 Sep 8. PMID: 24013610.	**Included**
704	Marder SR, Alphs L, Anghelescu IG, Arango C, Barnes TR, Caers I, Daniel DG, Dunayevich E, Fleischhacker WW, Garibaldi G, Green MF, Harvey PD, Kahn RS, Kane JM, Keefe RS, Kinon B, Leucht S, Lindenmayer JP, Malhotra AK, Stauffer V, Umbricht D, Wesnes K, Kapur S, Rabinowitz J. Issues and perspectives in designing clinical trials for negative symptoms in schizophrenia. Schizophr Res. 2013;150(2-3):328-33. doi: 10.1016/j.schres.2013.07.058. Epub 2013 Sep 9. PMID: 24028744.	Opinion
705	Stauffer VL, Millen BA, Andersen S, Kinon BJ, Lagrandeur L, Lindenmayer JP, Gomez JC. Pomaglumetad methionil: no significant difference as an adjunctive treatment for patients with prominent negative symptoms of schizophrenia compared to placebo. Schizophr Res. 2013;150(2-3):434-41. doi: 10.1016/j.schres.2013.08.020. Epub 2013 Sep 12. PMID: 24035403.	Non-marketed
706	Palm U, Keeser D, Blautzik J, Pogarell O, Ertl-Wagner B, Kupka MJ, Reiser M, Padberg F. Prefrontal transcranial direct current stimulation (tDCS) changes negative symptoms and functional connectivity MRI (fcMRI) in a single case of treatment-resistant schizophrenia. Schizophr Res. 2013;150(2-3):583-5. doi: 10.1016/j.schres.2013.08.043. Epub 2013 Sep 21. PMID: 24060570.	Case
707	Potkin SG, Phiri P, Szegedi A, Zhao J, Alphs L, Cazorla P. Long-term effects of asenapine or olanzapine in patients with persistent negative symptoms of schizophrenia: a pooled analysis. Schizophr Res. 2013;150(2-3):442-9. doi: 10.1016/j.schres.2013.08.024. Epub 2013 Sep 26. PMID: 24075603.	Open
708	Kim DW, Lee SH, Im CH. Source activation during facial emotion perception correlates with positive and negative symptoms scores of schizophrenia. Annu Int Conf IEEE Eng Med Biol Soc. 2013;2013:6325-8. doi: 10.1109/EMBC.2013.6611000. PMID: 24111187.	No drug
709	Caforio G, Di Giorgio A, Rampino A, Rizzo M, Romano R, Taurisano P, Fazio L, De Simeis G, Ursini G, Blasi G, Nardini M, Mancini M, Bertolino A. Mirtazapine add-on improves olanzapine effect on negative symptoms of schizophrenia. J Clin Psychopharmacol. 2013;33(6):810-2. doi: 10.1097/JCP.0b013e3182a4ec77. PMID: 24113675.	**Included**
710	Barnes SA, Der-Avakian A, Markou A. Anhedonia, avolition, and anticipatory deficits: assessments in animals with relevance to the negative symptoms of schizophrenia. Eur Neuropsychopharmacol. 2014;24(5):744-58. doi: 10.1016/j.euroneuro.2013.10.001. Epub 2013 Oct 14. PMID: 24183826; PMCID: PMC3986268.	Animal
711	Farokhnia M, Azarkolah A, Adinehfar F, Khodaie-Ardakani MR, Hosseini SM, Yekehtaz H, Tabrizi M, Rezaei F, Salehi B, Sadeghi SM, Moghadam M, Gharibi F, Mirshafiee O, Akhondzadeh S. N-acetylcysteine as an adjunct to risperidone for treatment of negative symptoms in patients with chronic schizophrenia: a randomized, double-blind, placebo-controlled study. Clin Neuropharmacol. 2013;36(6):185-92. doi: 10.1097/WNF.0000000000000001. PMID: 24201233.	**Included**
712	Vogel SJ, Strauss GP, Allen DN. Using negative feedback to guide behavior: impairments on the first 4 cards of the Wisconsin Card Sorting Test predict negative symptoms of schizophrenia. Schizophr Res. 2013;151(1-3):97-101. doi: 10.1016/j.schres.2013.07.052. Epub 2013 Nov 6. PMID: 24210530.	No drug
713	Kim DW, Kim HS, Lee SH, Im CH. Positive and negative symptom scores are correlated with activation in different brain regions during facial emotion perception in schizophrenia patients: a voxel-based sLORETA source activity study. Schizophr Res. 2013;151(1-3):165-74. doi: 10.1016/j.schres.2013.10.025. Epub 2013 Nov 20. PMID: 24268468.	No drug
714	Marder SR, Kirkpatrick B. Defining and measuring negative symptoms of schizophrenia in clinical trials. Eur Neuropsychopharmacol. 2014;24(5):737-43. doi: 10.1016/j.euroneuro.2013.10.016. Epub 2013 Nov 11. PMID: 24275698.	No drug
715	Foussias G, Agid O, Fervaha G, Remington G. Negative symptoms of schizophrenia: clinical features, relevance to real world functioning and specificity versus other CNS disorders. Eur Neuropsychopharmacol. 2014;24(5):693-709. doi: 10.1016/j.euroneuro.2013.10.017. Epub 2013 Nov 11. PMID: 24275699.	No drug
716	Neill JC, Harte MK, Haddad PM, Lydall ES, Dwyer DM. Acute and chronic effects of NMDA receptor antagonists in rodents, relevance to negative symptoms of schizophrenia: a translational link to humans. Eur Neuropsychopharmacol. 2014;24(5):822-35. doi: 10.1016/j.euroneuro.2013.09.011. Epub 2013 Oct 12. PMID: 24287012.	Animal
717	O'Tuathaigh CM, Desbonnet L, Waddington JL. Genetically modified mice related to schizophrenia and other psychoses: seeking phenotypic insights into the pathobiology and treatment of negative symptoms. Eur Neuropsychopharmacol. 2014;24(5):800-21. doi: 10.1016/j.euroneuro.2013.08.009. Epub 2013 Oct 12. PMID: 24290531.	Animal

718	Reeves KC, Virk S, Niedermier J, Duchemin AM. Addition of amoxapine improves positive and negative symptoms in a patient with schizophrenia. Ther Adv Psychopharmacol. 2013;3(6):340-2. doi: 10.1177/2045125313499363. PMID: 24294487; PMCID: PMC3840811.	Case
719	Mehta UM, Thirthalli J, Kumar CN, Kumar JK, Gangadhar BN. Negative symptoms mediate the influence of theory of mind on functional status in schizophrenia. Soc Psychiatry Psychiatr Epidemiol. 2014;49(7):1151-6. doi: 10.1007/s00127-013-0804-x. Epub 2013. PMID: 24297622.	No drug
720	Campellone TR, Caponigro JM, Kring AM. The power to resist: the relationship between power, stigma, and negative symptoms in schizophrenia. Psychiatry Res. 2014;215(2):280-5. doi: 10.1016/j.psychres.2013.11.020. Epub 2013. PMID: 24326180; PMCID: PMC4005826	No drug
721	Tang H, Dalton CF, Srisawat U, Zhang ZJ, Reynolds GP. Methylation at a transcription factor-binding site on the 5-HT1A receptor gene correlates with negative symptom treatment response in first episode schizophrenia. Int J Neuropsychopharmacol. 2014;17(4):645-9. doi: 10.1017/S1461145713001442. Epub 2013. PMID: 24331356.	Unfocused
722	Moser P. Evaluating negative-symptom-like behavioural changes in developmental models of schizophrenia. Eur Neuropsychopharmacol. 2014;24(5):774-87. doi: 10.1016/j.euroneuro.2013.11.004. Epub 2013. PMID: 24332891.	Animal
723	Wilson CA, Koenig JI. Social interaction and social withdrawal in rodents as readouts for investigating the negative symptoms of schizophrenia. Eur Neuropsychopharmacol. 2014;24(5):759-73. doi: 10.1016/j.euroneuro.2013.11.008. Epub 2013. PMID: 24342774; PMCID: PMC4481734.	Animal
724	Niitsu T, Ishima T, Yoshida T, Hashimoto T, Matsuzawa D, Shirayama Y, Nakazato M, Shimizu E, Hashimoto K, Iyo M. A positive correlation between serum levels of mature brain-derived neurotrophic factor and negative symptoms in schizophrenia. Psychiatry Res. 2014;215(2):268-73. doi: 10.1016/j.psychres.2013.12.009. Epub 2013. PMID: 24377440.	No drug
725	Shi C, Yu X, Cheung EF, Shum DH, Chan RC. Revisiting the therapeutic effect of rTMS on negative symptoms in schizophrenia: a meta-analysis. Psychiatry Res. 2014;215(3):505-13. doi: 10.1016/j.psychres.2013.12.019. Epub 2013. PMID: 24411074; PMCID: PMC4127383.	Review
726	Khodaie-Ardakani MR, Mirshafiee O, Farokhnia M, Tajdini M, Hosseini SM, Modabbernia A, Rezaei F, Salehi B, Yekehtaz H, Ashrafi M, Tabrizi M, Akhondzadeh S. Minocycline add-on to risperidone for treatment of negative symptoms in patients with stable schizophrenia: randomized double-blind placebo-controlled study. Psychiatry Res. 2014;215(3):540-6. doi: 10.1016/j.psychres.2013.12.051. Epub 2014.	**Included**
727	Bracht T, Horn H, Strik W, Federspiel A, Razavi N, Stegmayer K, Wiest R, Dierks T, Müller TJ, Walther S. White matter pathway organization of the reward system is related to positive and negative symptoms in schizophrenia. Schizophr Res. 2014;153(1-3):136-42. doi: 10.1016/j.schres.2014.01.015. Epub 2014.	No drug
728	Liu F, Guo X, Wu R, Ou J, Zheng Y, Zhang B, Xie L, Zhang L, Yang L, Yang S, Yang J, Ruan Y, Zeng Y, Xu X, Zhao J. Minocycline supplementation for treatment of negative symptoms in early-phase schizophrenia: a double blind, randomized, controlled trial. Schizophr Res. 2014;153(1-3):169-76. doi: 10.1016/j.schres.2014.01.011. Epub 2014. PMID: 24503176.	**Included**
729	Tsai CH, Chen TT, Huang WL. Combination of escitalopram and aripiprazole causes significant improvement of negative symptoms of simple schizophrenia. Psychiatry Clin Neurosci. 2014;68(7):582-3. doi: 10.1111/pcn.12167. Epub 2014. PMID: 24521035.	Case
730	Schoemaker JH, Jansen WT, Schipper J, Szegedi A. The selective glycine uptake inhibitor org 25935 as an adjunctive treatment to atypical antipsychotics in predominant persistent negative symptoms of schizophrenia: results from the GIANT trial. J Clin Psychopharmacol. 2014;34(2):190-8. doi: 10.1097/JCP.0000000000000073. PMID: 24525661.	Non-marketed
731	Ritsner MS, Bawakny H, Kreinin A. Pregnenolone treatment reduces severity of negative symptoms in recent-onset schizophrenia: an 8-week, double-blind, randomized add-on two-center trial. Psychiatry Clin Neurosci. 2014;68(6):432-40. doi: 10.1111/pcn.12150. Epub 2014. PMID: 24548129.	**Included**
732	Ventura J, Subotnik KL, Ered A, Gretchen-Doorly D, Hellemann GS, Vaskinn A, Nuechterlein KH. The relationship of attitudinal beliefs to negative symptoms, neurocognition, and daily functioning in recent-onset schizophrenia. Schizophr Bull. 2014;40(6):1308-18. doi: 10.1093/schbul/sbu002. Epub 2014. PMID: 24561318; PMCID: PMC4193707.	No drug
733	Asevedo E, Rizzo LB, Gadelha A, Mansur RB, Ota VK, Berberian AA, Scarpato BS, Teixeira AL, Bressan RA, Brietzke E. Peripheral interleukin-2 level is associated with negative symptoms and cognitive performance in schizophrenia. Physiol Behav. 2014;129:194-8. doi: 10.1016/j.physbeh.2014.02.032. Epub 2014. PMID: 24576679.	No drug
734	Fervaha G, Foussias G, Agid O, Remington G. Impact of primary negative symptoms on functional outcomes in schizophrenia. Eur Psychiatry. 2014;29(7):449-55. doi: 10.1016/j.eurpsy.2014.01.007. Epub 2014. PMID: 24630742.	No drug
735	Hosseini SM, Farokhnia M, Rezaei F, Gougol A, Yekehtaz H, Iranpour N, Salehi B, Tabrizi M, Tajdini M, Ghaleiha A, Akhondzadeh S. Intranasal desmopressin as an adjunct to risperidone for negative symptoms of schizophrenia: a randomized, double-blind, placebo-controlled, clinical trial. Eur Neuropsychopharmacol. 2014;24(6):846-55. doi: 10.1016/j.euroneuro.2014.02.001. Epub 2014. PMID: 24636461.	**Included**
736	Ceccarini J. Beschikbaarheid cannabinoïd type 1-receptor in de nucleus accumbens en negatieve symptomen bij medicatievrije patiënten met schizofrenie [Increased ventral striatal CB1 receptor binding is related to negative symptoms in drug-free patients with schizophrenia]. Tijdschr Psychiatr. 2014;56(2):129-30. Dutch. PMID: 24665487.	Opinion
737	Limosin F. Neurodevelopmental and environmental hypotheses of negative symptoms of schizophrenia. BMC Psychiatry. 2014;14:88. doi: 10.1186/1471-244X-14-88. PMID: 24670212; PMCID: PMC3986891.	Review
738	Boutros NN, Mucci A, Vignapiano A, Galderisi S. Electrophysiological aberrations associated with negative symptoms in schizophrenia. Curr Top Behav Neurosci. 2014;21:129-56. doi: 10.1007/7854_2014_303. PMID: 24671702.	Review
739	Kingwell K. Schizophrenia drug gets negative results for negative symptoms. Nat Rev Drug Discov. 2014;13(4):244-5. doi: 10.1038/nrd4294. PMID: 24687052.	Opinion
740	Umbricht D, Alberati D, Martin-Facklam M, Borroni E, Youssef EA, Ostland M, Wallace TL, Knoflach F, Dorflinger E, Wettstein JG, Bausch A, Garibaldi G, Santarelli L. Effect of bitopertin, a glycine reuptake inhibitor, on negative symptoms of schizophrenia: a randomized, double-blind, proof-of-concept study. JAMA Psychiatry. 2014;71(6):637-46. doi: 10.1001/jamapsychiatry.2014.163. PMID: 24696094.	Non-marketed
741	Graux J, Bidet-Caulet A, Bonnet-Brilhault F, Camus V, Bruneau N. Hallucinations and negative symptoms differentially revealed by frontal and temporal responses to speech in schizophrenia. Schizophr Res. 2014;155(1-3):39-44. doi: 10.1016/j.schres.2014.03.007. Epub 2014. PMID 24703528.	No drug
742	Bodapati AS, Herbener ES. The impact of social content and negative symptoms on affective ratings in schizophrenia. Psychiatry Res. 2014;218(1-2):25-30. doi: 10.1016/j.psychres.2014.03.039. Epub 2014. PMID: 24745467; PMCID: PMC4063555.	No drug
743	He Q, Chen Y, Chow SL, Huang MM, Zhang ZQ, Zhang SS, Shen WW, Deng H. [Negative symptoms predict the improvement of social functioning of patients with schizophrenia]. Sichuan Da Xue Xue Bao Yi Xue Ban. 2014;45(2):284-8. Chinese. PMID: 24749359.	No drug
744	Gruber O, Chadha Santuccione A, Aach H. Magnetic resonance imaging in studying schizophrenia, negative symptoms, and the glutamate system. Front Psychiatry. 2014;5:32. doi: 10.3389/fpsyt.2014.00032. PMID: 24765078; PMCID: PMC3982059.	No drug
745	Gorynia I, Schwaiger M, Heinz A. Effects of eye dominance (left vs. right) and cannabis use on intermanual coordination and negative symptoms in schizophrenia patients. Eur Arch Psychiatry Clin Neurosci. 2014;264(8):683-95. doi: 10.1007/s00406-014-0503-y. Epub 2014. PMID: 24792218.	No drug
746	Levine SZ, Leucht S. Treatment response heterogeneity in the predominant negative symptoms of schizophrenia: analysis of amisulpride vs placebo in three clinical trials. Schizophr Res. 2014;156(1):107-14. doi: 10.1016/j.schres.2014.04.005. Epub 2014. PMID: 24794879.	Duplicate=176,225,1433
747	Edgar CJ, Blaettler T, Bugarski-Kirola D, Le Scouiller S, Garibaldi GM, Marder SR. Reliability, validity and ability to detect change of the PANSS negative symptom factor score in outpatients with schizophrenia on select antipsychotics and with prominent negative or disorganized thought symptoms. Psychiatry Res. 2014;218(1-2):219-24. doi: 10.1016/j.psychres.2014.04.009. Epub 2014. PMID: 24809242.	No drug

748	Rocca P, Montemagni C, Zappia S, Piterà R, Sigaudo M, Bogetto F. Negative symptoms and everyday functioning in schizophrenia: a cross-sectional study in a real world-setting. Psychiatry Res. 2014;218(3):284-9. doi: 10.1016/j.psychres.2014.04.018. Epub 2014. PMID: 24814140.	No drug
749	Paraschakis A. Tackling negative symptoms of schizophrenia with memantine. Case Rep Psychiatry. 2014;2014:384783. doi: 10.1155/2014/384783. Epub 2014. PMID: 24818033; PMCID: PMC4003755.	Case
750	Millan MJ, Fone K, Steckler T, Horan WP. Negative symptoms of schizophrenia: clinical characteristics, pathophysiological substrates, experimental models and prospects for improved treatment. Eur Neuropsychopharmacol. 2014;24(5):645-92. doi: 10.1016/j.euroneuro.2014.03.008. Epub 2014. PMID: 24820238.	Review
751	Rabany L, Deutsch L, Levkovitz Y. Double-blind, randomized sham controlled study of deep-TMS add-on treatment for negative symptoms and cognitive deficits in schizophrenia. J Psychopharmacol. 2014;28(7):686-90. doi: 10.1177/0269881114533600. Epub 2014. PMID: 24829210.	No drug
752	Chue P, Lalonde JK. Addressing the unmet needs of patients with persistent negative symptoms of schizophrenia: emerging pharmacological treatment options. Neuropsychiatr Dis Treat. 2014;10:777-89. doi: 10.2147/NDT.S43404. PMID: 24855363; PMCID: PMC4020880.	Review
753	Granholm E, Holden J, Link PC, McQuaid JR. Randomized clinical trial of cognitive behavioral social skills training for schizophrenia: improvement in functioning and experiential negative symptoms. J Consult Clin Psychol. 2014;82(6):1173-85. doi: 10.1037/a0037098. Epub 2014. PMID: 24911420; PMCID: PMC4244255.	No drug
754	Quinlan T, Roesch S, Granholm E. The role of dysfunctional attitudes in models of negative symptoms and functioning in schizophrenia. Schizophr Res. 2014;157(1-3):182-9. doi: 10.1016/j.schres.2014.05.025. Epub 2014. PMID: 24924405; PMCID: PMC4099260.	No drug
755	Azorin JM, Belzeaux R, Adida M. Negative symptoms in schizophrenia: Where we have been and where we are heading. CNS Neurosci Ther. 2014;20(9):801-8. doi: 10.1111/cns.12292. Epub 2014. PMID: 24931186; PMCID: PMC6493052.	Review
756	Narayanaswamy JC, Shivakumar V, Bose A, Agarwal SM, Venkatasubramanian G, Gangadhar BN. Sustained improvement of negative symptoms in schizophrenia with add-on tDCS: a case report. Clin Schizophr Relat Psychoses. 2014;8(3):135-6. doi: 10.3371/CSRP.JNVS.061314. PMID: 24951718.	Case
757	Bijanki KR, Hodis B, Magnotta VA, Zeien E, Andreasen NC. Effects of age on white matter integrity and negative symptoms in schizophrenia. Schizophr Res. 2015;161(1):29-35. doi: 10.1016/j.schres.2014.05.031. Epub 2014. PMID: 24957354; PMCID: PMC4272674.	No drug
758	Ohtani T, Bouix S, Hosokawa T, Saito Y, Eckbo R, Ballinger T, Rausch A, Melonakos E, Kubicki M. Abnormalities in white matter connections between orbitofrontal cortex and anterior cingulate cortex and their associations with negative symptoms in schizophrenia: a DTI study. Schizophr Res. 2014;157(1-3):190-7. doi: 10.1016/j.schres.2014.05.016. Epub 2014. PMID: 24962436; PMCID: PMC4679151.	No drug
759	Gupta M, Holshausen K, Gou L, Bowie C. Measuring negative symptom change in schizophrenia: considering alternatives to self-report. Expert Rev Neurother. 2014;14(8):911-22. doi: 10.1586/14737175.2014.935341. Epub 2014. PMID: 24976136.	No drug
760	Velthorst E, Koeter M, van der Gaag M, Nieman DH, Fett AK, Smit F, Staring AB, Meijer C, de Haan L. Adapted cognitive-behavioural therapy required for targeting negative symptoms in schizophrenia: meta-analysis and meta-regression. Psychol Med. 2015;45(3):453-65. doi: 10.1017/S0033291714001147. Epub 2014. PMID: 24993642.	No drug
761	Strauss GP, Morra LF, Sullivan SK, Gold JM. The role of low cognitive effort and negative symptoms in neuropsychological impairment in schizophrenia. Neuropsychology. 2015;29(2):282-91. doi: 10.1037/neu0000113. Epub 2014. PMID: 25000322; PMCID: PMC4286525.	No drug
762	Usall J, López-Carrilero R, Iniesta R, Roca M, Caballero M, Rodriguez- Jimenez R, Oliveira C, Bernardo M, Corripio I, Sindreu SD, González Piqueras JC, Felipe AE, Fernandez de Corres B, Ibáñez A, Huerta R; Abordaje Síntomas Negativos Esquizofrenia Group. Double-blind, placebo-controlled study of the efficacy of reboxetine and citalopram as adjuncts to atypical antipsychotics for negative symptoms of schizophrenia. J Clin Psychiatry. 2014;75(6):608-15. doi: 10.4088/JCP.13m08551. PMID: 25004184.	**Included**
763	Thirioux B, Tandonnet L, Jaafari N, Berthoz A. Disturbances of spontaneous empathic processing relate with the severity of the negative symptoms in patients with schizophrenia: a behavioural pilot-study using virtual reality technology. Brain Cogn. 2014;90:87-99. doi: 10.1016/j.bandc.2014.06.006. Epub 2014. PMID: 25014409.	No drug
764	Krishnadas R, Ramanathan S, Wong E, Nayak A, Moore B. Residual negative symptoms differentiate cognitive performance in clinically stable patients with schizophrenia and bipolar disorder. Schizophr Res Treatment. 2014;2014:785310. doi: 10.1155/2014/785310. Epub 2014. PMID: 25024847; PMCID: PMC4082914.	No drug
765	Sicras-Mainar A, Maurino J, Ruiz-Beato E, Navarro-Artieda R. Impact of negative symptoms on healthcare resource utilization and associated costs in adult outpatients with schizophrenia: a population-based study. BMC Psychiatry. 2014;14:225. doi: 10.1186/s12888-014-0225-8. PMID: 25096022; PMCID: PMC4149268.	No drug
766	Üçok A, Ergül C. Persistent negative symptoms after first episode schizophrenia: A 2-year follow-up study. Schizophr Res. 2014;158(1-3):241-6. doi: 10.1016/j.schres.2014.07.021. Epub 2014 Aug 5. PMID: 25107850.	No drug
767	Subotnik KL, Ventura J, Gretchen-Doorly D, Hellemann GS, Agee ER, Casaus LR, Luo JS, Villa KF, Nuechterlein KH. The impact of second-generation antipsychotic adherence on positive and negative symptoms in recent-onset schizophrenia. Schizophr Res. 2014;159(1):95-100. doi: 10.1016/j.schres.2014.07.008. Epub 2014 Aug 6. PMID: 25108771; PMCID: PMC4177349.	Open
768	Horton LE, Smith AA, Haas GL. The nature and timing of social deficits in child and adolescent offspring of parents with schizophrenia: preliminary evidence for precursors of negative symptoms? Schizophr Res. 2014;159(1):27-30. doi: 10.1016/j.schres.2014.07.007. Epub 2014 Aug 10. PMID: 25112161; PMCID: PMC4243614.	No drug
769	Zhao S, Kong J, Li S, Tong Z, Yang C, Zhong H. Randomized controlled trial of four protocols of repetitive transcranial magnetic stimulation for treating the negative symptoms of schizophrenia. Shanghai Arch Psychiatry. 2014;26(1):15-21. doi: 10.3969/j.issn.1002-0829.2014.01.003. PMID: 25114477; PMCID: PMC4117998.	No drug
770	Montemagni C, Castagna F, Crivelli B, De Marzi G, Frieri T, Macrì A, Rocca P. Relative contributions of negative symptoms, insight, and coping strategies to quality of life in stable schizophrenia. Psychiatry Res. 2014;220(1-2):102-11. doi: 10.1016/j.psychres.2014.07.019. Epub 2014 Jul 30. PMID: 25128248.	No drug
771	Pawełczyk T, Kołodziej-Kowalska E, Pawełczyk A, Rabe-Jabłońska J. Effectiveness and clinical predictors of response to combined ECT and antipsychotic therapy in patients with treatment-resistant schizophrenia and dominant negative symptoms. Psychiatry Res. 2014;220(1-2):175-80. doi: 10.1016/j.psychres.2014.07.071. Epub 2014 Aug 1. PMID: 25129562.	No drug
772	Sisek-Šprem M, Križaj A, Jukić V, Milošević M, Petrović Z, Herceg M. Testosterone levels and clinical features of schizophrenia with emphasis on negative symptoms and aggression. Nord J Psychiatry. 2015;69(2):102-9. doi: 10.3109/08039488.2014.947320. Epub 2014 Aug 25. PMID: 25151994.	No drug
773	Dunayevich E, Chen CY, Marder SR, Rabinowitz J. Restrictive symptomatic inclusion criteria create barriers to clinical research in schizophrenia negative symptoms: an analysis of the CATIE dataset. Eur Neuropsychopharmacol. 2014;24(10):1615-21. doi: 10.1016/j.euroneuro.2014.08.004. Epub 2014 Aug 17. PMID: 25172269.	No drug
774	Ergül C, Üçok A. Negative symptom subgroups have different effects on the clinical course of schizophrenia after the first episode: a 24-month follow up study. Eur Psychiatry. 2015;30(1):14-9. doi: 10.1016/j.eurpsy.2014.07.005. Epub 2014 Aug 27. PMID: 25174272.	No drug
775	Mehta VS, Ram D. Role of ranitidine in negative symptoms of schizophrenia—an open label study. Asian J Psychiatr. 2014;12:150-4. doi: 10.1016/j.ajp.2014.08.005. Epub 2014 Aug 23. PMID: 25193506.	Open
776	Chuang JY, Murray GK, Metastasio A, Segarra N, Tait R, Spencer J, Ziauddeen H, Dudas RB, Fletcher PC, Suckling J. Brain structural signatures of negative symptoms in depression and schizophrenia. Front Psychiatry. 2014;5:116. doi: 10.3389/fpsyt.2014.00116.	No drug
777	Nestor PG, Choate V, Niznikiewicz M, Levitt JJ, Shenton ME, McCarley RW. Neuropsychology of reward learning and negative symptoms in schizophrenia. Schizophr Res. 2014;159(2-3):506-8. doi: 10.1016/j.schres.2014.08.028. Epub 2014 Sep 26.	No drug

778	Andrade C, Kisely S, Monteiro I, Rao S. Antipsychotic augmentation with modafinil or armodafinil for negative symptoms of schizophrenia: systematic review and meta-analysis of randomized controlled trials. J Psychiatr Res. 2015;60:14-21. doi: 10.1016/j.jpsychires.2014.09.013. Epub 2014 Sep 20. PMID: 25306261.	Review
**779**	**Mirabzadeh A, Kimiaghalam P, Fadai F, Samiei M, Daneshmand R. The therapeutic effectiveness of risperidone on negative symptoms of schizophrenia in comparison with haloperidol: a randomized clinical trial. Basic Clin Neurosci. 2014;5(3):212-7.**	**Included**
**780**	**Ghanizadeh A, Dehbozorgi S, Omrani Sigaroodi M, Rezaei Z. Minocycline as add-on treatment decreases the negative symptoms of schizophrenia; a randomized placebo-controlled clinical trial. Recent Pat Inflamm Allergy Drug Discov. 2014;8(3):211-5. doi: 10.2174/1872213x08666141029123524.**	**Included**
781	Dlabac-de Lange JJ, Bais L, van Es FD, Visser BG, Reinink E, Bakker B, van den Heuvel ER, Aleman A, Knegtering H. Efficacy of bilateral repetitive transcranial magnetic stimulation for negative symptoms of schizophrenia: results of a multicenter double-blind randomized controlled trial. Psychol Med. 2015;45(6):1263-75. doi: 10.1017/S0033291714002360. Epub 2014 Oct 30. PMID: 25354751.	No drug
782	Zhu Y, Wang Z, Ni J, Zhang Y, Chen M, Cai J, Li X, Zhang W, Zhang C. Genetic variant in NDUFS1 gene is associated with schizophrenia and negative symptoms in Han Chinese. J Hum Genet. 2015;60(1):11-6. doi: 10.1038/jhg.2014.94. Epub 2014 Oct 30. PMID: 25354934.	No drug
783	Millier A, Siegrist K, Amri I, Toumi M, Aballéa S. Social contacts reduce negative symptoms, especially emotional withdrawal in patients with schizophrenia. Value Health. 2014;17(7):A455. doi: 10.1016/j.jval.2014.08.1244. Epub 2014 Oct 26. PMID: 27201263.	No drug
784	Sicras-Mainar A, Ruiz-Beato E, Mauriño J, Navarro-Artieda R. Prevalence of metabolic syndrome in patients with schizophrenia according to the presence or absence of negative symptoms. Value Health. 2014;17(7):A456. doi: 10.1016/j.jval.2014.08.1248. Epub 2014 Oct 26. PMID: 27201269.	No drug
785	Refaï T, Millier A, Toumi M. Evolution of presence of predominant negative symptoms in patients with schizophrenia. Value Health. 2014;17(7):A766. doi: 10.1016/j.jval.2014.08.290. Epub 2014 Oct 26. PMID: 27202815.	No drug
786	Pawełczyk T, Kołodziej-Kowalska E, Pawełczyk A, Rabe-Jabłońska J. Augmentation of antipsychotics with electroconvulsive therapy in treatment-resistant schizophrenia patients with dominant negative symptoms: a pilot study of effectiveness. Neuropsychobiology. 2014;70(3):158-64. doi: 10.1159/000366484. Epub 2014 Oct 24. PMID: 25358377.	No drug
**787**	**Buchanan RW, Weiner E, Kelly DL, Gold JM, Keller WR, Waltz JA, McMahon RP, Gorelick DA. Rasagiline in the treatment of the persistent negative symptoms of schizophrenia. Schizophr Bull. 2015;41(4):900-8. doi: 10.1093/schbul/sbu151. Epub 2014 Nov 2.**	**Included**
788	Spiros A, Roberts P, Geerts H. A computer-based quantitative systems pharmacology model of negative symptoms in schizophrenia: exploring glycine modulation of excitation-inhibition balance. Front Pharmacol. 2014;5:229. doi: 10.3389/fphar.2014.00229. PMID: 25374541; PMCID: PMC4204440.	No drug
789	Park S, Lee MK. Successful electroconvulsive therapy and improvement of negative symptoms in refractory schizophrenia with clozapine-induced seizures: a case report. Psychiatr Danub. 2014;26(4):360-2. PMID: 25377372.	No drug
790	Ahmed AO, Strauss GP, Buchanan RW, Kirkpatrick B, Carpenter WT. Are negative symptoms dimensional or categorical? Detection and validation of deficit schizophrenia with taxometric and latent variable mixture models. Schizophr Bull. 2015;41(4):879-91. doi: 10.1093/schbul/sbu163. Epub 2014 Nov 14. PMID: 25399026; PMCID: PMC4466177.	No drug
791	Carbon M, Correll CU. Thinking and acting beyond the positive: the role of the cognitive and negative symptoms in schizophrenia. CNS Spectr. 2014;19(Suppl 1):38-52; quiz 35-7, 53. doi: 10.1017/S1092852914000601. Epub 2014 Nov 18. PMID: 25403863.	No drug
792	Miyamoto Y, Nitta A. Behavioral phenotypes for negative symptoms in animal models of schizophrenia. J Pharmacol Sci. 2014;126(4):310-20. doi: 10.1254/jphs.14R02CR. Epub 2014 Nov 18. PMID: 25409784.	Animal
793	Hornig T, Valerius G, Feige B, Bubl E, Olbrich HM, van Elst LT. Neuropsychological and cerebral morphometric aspects of negative symptoms in schizophrenia: negative symptomatology is associated with specific mnestic deficits in schizophrenic patients. BMC Psychiatry. 2014;14:326. doi: 10.1186/s12888-014-0326-4. PMID: 25420531; PMCID: PMC4247219.	No drug
794	Savill M, Banks C, Khanom H, Priebe S. Do negative symptoms of schizophrenia change over time? A meta-analysis of longitudinal data. Psychol Med. 2015;45(8):1613-27. doi: 10.1017/S0033291714002712. Epub 2014 Nov 26. PMID: 25425086.	Review
795	Quan WX, Zhu XL, Qiao H, Zhang WF, Tan SP, Zhou DF, Wang XQ. The effects of high-frequency repetitive transcranial magnetic stimulation (rTMS) on negative symptoms of schizophrenia and the follow-up study. Neurosci Lett. 2015;584:197-201. doi: 10.1016/j.neulet.2014.10.029. Epub 2014 Oct 24. PMID: 25449864.	No drug
796	Vancampfort D, De Hert M, Stubbs B, Ward PB, Rosenbaum S, Soundy A, Probst M. Negative symptoms are associated with lower autonomous motivation towards physical activity in people with schizophrenia. Compr Psychiatry. 2015;56:128-32. doi: 10.1016/j.comppsych.2014.10.007. Epub 2014 Oct 16. PMID: 25458480.	No drug
797	Robertson BR, Prestia D, Twamley EW, Patterson TL, Bowie CR, Harvey PD. Social competence versus negative symptoms as predictors of real world social functioning in schizophrenia. Schizophr Res. 2014;160(1-3):136-41. doi: 10.1016/j.schres.2014.10.037. Epub 2014 Nov 7. PMID: 25468184; PMCID: PMC4258126.	No drug
798	Bersani FS, Minichino A, Fojanesi M, Gallo M, Maglio G, Valeriani G, Biondi M, Fitzgerald PB. Cingulate cortex in schizophrenia: its relation with negative symptoms and psychotic onset. A review study. Eur Rev Med Pharmacol Sci. 2014;18(22):3354-67. PMID: 25491609.	Review
799	Ventura J, Subotnik KL, Gitlin MJ, Gretchen-Doorly D, Ered A, Villa KF, Hellemann GS, Nuechterlein KH. Negative symptoms and functioning during the first year after a recent onset of schizophrenia and 8 years later. Schizophr Res. 2015;161(2-3):407-13. doi: 10.1016/j.schres.2014.10.043. Epub 2014 Dec 8. PMID: 25499044; PMCID: PMC4308531.	No drug
800	Foussias G, Siddiqui I, Fervaha G, Agid O, Remington G. Dissecting negative symptoms in schizophrenia: opportunities for translation into new treatments. J Psychopharmacol. 2015;29(2):116-26. doi: 10.1177/0269881114562092. Epub 2014 Dec 16. PMID: 25516370.	Review
801	Fusar-Poli P, Papanastasiou E, Stahl D, Rocchetti M, Carpenter W, Shergill S, McGuire P. Treatments of negative symptoms in schizophrenia: Meta-analysis of 168 randomized placebo-controlled trials. Schizophr Bull. 2015;41(4):892-9. doi: 10.1093/schbul/sbu170. Epub 2014 Dec 20. Erratum in: Schizophr Bull. 2019 Aug 26;:null. PMID: 25528757; PMCID: PMC4466178.	Review
802	Docx L, de la Asuncion J, Sabbe B, Hoste L, Baeten R, Warnaerts N, Morrens M. Effort discounting and its association with negative symptoms in schizophrenia. Cogn Neuropsychiatry. 2015;20(2):172-85. doi: 10.1080/13546805.2014.993463. Epub 2015 Jan 3. PMID: 25559619.	No drug
803	Sicras-Mainar A, Maurino J, Ruiz-Beato E, Navarro-Artieda R. Prevalence of metabolic syndrome according to the presence of negative symptoms in patients with schizophrenia. Neuropsychiatr Dis Treat. 2014;11:51-7. doi: 10.2147/NDT.S75449. PMID: 25565850; PMCID: PMC4283985.	No drug
804	Harsing LG Jr, Timar J, Szabo G, Udvari S, Nagy KM, Marko B, Zsilla G, Czompa A, Tapolcsanyi P, Kocsis A, Matyus P. Sarcosine-based glycine transporter type-1 (GlyT-1) inhibitors containing pyridazine moiety: A further search for drugs with potential to influence schizophrenia negative symptoms. Curr Pharm Des. 2015;21(17):2291-303. doi: 10.2174/1381612821666150109125623. PMID: 25578890.	Animal
805	Schooler NR, Buchanan RW, Laughren T, Leucht S, Nasrallah HA, Potkin SG, Abi-Saab D, Berardo CG, Bugarski-Kirola D, Blaettler T, Edgar CJ, Nordstroem AL, O'Gorman C, Garibaldi G. Defining therapeutic benefit for people with schizophrenia: focus on negative symptoms. Schizophr Res. 2015;162(1-3):169-74. doi: 10.1016/j.schres.2014.12.001. Epub 2015 Jan 9. PMID: 25579053.	Review
806	Wobrock T, Guse B, Cordes J, Wölwer W, Winterer G, Gaebel W, Langguth B, Landgrebe M, Eichhammer P, Frank E, Hajak G, Ohmann C, Verde PE, Rietschel M, Ahmed R, Honer WG, Malchow B, Schneider-Axmann T, Falkai P, Hasan A. Left prefrontal high-frequency repetitive transcranial magnetic stimulation for the treatment of schizophrenia with predominant negative symptoms: a sham- controlled, randomized multicenter trial. Biol Psychiatry. 2015;77(11):979-88. doi: 10.1016/j.biopsych.2014.10.009. Epub 2014 Oct 23. PMID: 25582269.	No drug

807	Strauss GP, Keller WR, Koenig JI, Gold JM, Ossenfort KL, Buchanan RW. Plasma oxytocin levels predict olfactory identification and negative symptoms in individuals with schizophrenia. Schizophr Res. 2015;162(1-3):57-61. doi: 10.1016/j.schres.2014.12.023. Epub 2015 Jan 9. PMID: 25583247; PMCID: PMC4339311.	No drug
808	Yassini M, Shariat N, Nadi M, Amini F, Vafaee M. The effects of bupropion on negative symptoms in schizophrenia. Iran J Pharm Res. 2014;13(4):1227-33.	**Included**
809	Yefet K, Goldstein A, Rabany L, Levkovitz Y. Impairments of event-related magnetic fields in schizophrenia patients with predominant negative symptoms. Psychiatry Res. 2015;231(3):325-32. doi: 10.1016/j.pscychresns.2015.01.016. Epub 2015 Jan 23. PMID: 25680554.	No drug
810	Werbeloff N, Dohrenwend BP, Yoffe R, van Os J, Davidson M, Weiser M. The association between negative symptoms, psychotic experiences and later schizophrenia: a population-based longitudinal study. PLoS One. 2015;10(3):e0119852. doi: 10.1371/journal.pone.0119852. PMID: 25748557; PMCID: PMC4351950.	No drug
811	Mucci A, Galderisi S, Merlotti E, Rossi A, Rocca P, Bucci P, Piegari G, Chieffi M, Vignapiano A, Maj M; Italian Network for Research on Psychoses. The Brief Negative Symptom Scale (BNSS): Independent validation in a large sample of Italian patients with schizophrenia. Eur Psychiatry. 2015;30(5):641-7. doi: 10.1016/j.eurpsy.2015.01.014. Epub 2015 Mar 8. PMID: 25758156	No drug
812	Rahm C, Liberg B, Reckless G, Ousdal O, Melle I, Andreassen OA, Agartz I. Negative symptoms in schizophrenia show association with amygdala volumes and neural activation during affective processing. Acta Neuropsychiatr. 2015;27(4):213-20. doi: 10.1017/neu.2015.11. Epub 2015 Mar 17. PMID: 25777814.	No drug
813	Levental U, Bersudsky Y, Dwalatzky T, Lerner V, Medina S, Levine J. A pilot open study of long term high dose creatine augmentation in patients with treatment resistant negative symptoms schizophrenia. Isr J Psychiatry Relat Sci. 2015;52(1):6-10. PMID: 25841104.	Open
814	Strassnig MT, Raykov T, O'Gorman C, Bowie CR, Sabbag S, Durand D, Patterson TL, Pinkham A, Penn DL, Harvey PD. Determinants of different aspects of everyday outcome in schizophrenia: The roles of negative symptoms, cognition, and functional capacity. Schizophr Res. 2015;165(1):76-82. doi: 10.1016/j.schres.2015.03.033. Epub 2015 Apr 11. PMID: 25868935; PMCID: PMC4437911.	No drug
815	Chaki S, Shimazaki T, Karasawa J, Aoki T, Kaku A, Iijima M, Kambe D, Yamamoto S, Kawakita Y, Shibata T, Abe K, Okubo T, Sekiguchi Y, Okuyama S. Efficacy of a glycine transporter 1 inhibitor TASP0315003 in animal models of cognitive dysfunction and negative symptoms of schizophrenia. Psychopharmacology (Berl). 2015;232(15):2849-61. doi: 10.1007/s00213-015-3920-3. Epub 2015 Apr 15. PMID: 25869273.	Animal
816	Möller HJ, Czobor P. Pharmacological treatment of negative symptoms in schizophrenia. Eur Arch Psychiatry Clin Neurosci. 2015;265(7):567-78. doi: 10.1007/s00406-015-0596-y. Epub 2015 Apr 21. PMID: 25895634.	Review
817	Bochkarev VK, Kirenskaya AV, Tkachenko AA, Samylkin DV, Novototsky-Vlasov VY, Kovaleva ME. [EEG frequency and regional properties in patients with paranoid schizophrenia: effects of positive and negative symptomatology prevalence]. Zh Nevrol Psikhiatr Im S S Korsakova. 2015;115(1):66-74. Russian. doi: 10.17116/jnevro20151151166-74. PMID: 25909792.	No drug
818	Kuswanto CN, Sum MY, Qiu A, Sitoh YY, Liu J, Sim K. The impact of genome wide supported microRNA-137 (MIR137) risk variants on frontal and striatal white matter integrity, neurocognitive functioning, and negative symptoms in schizophrenia. Am J Med Genet B Neuropsychiatr Genet. 2015;168B(5):317-26. doi: 10.1002/ajmg.b.32314. Epub 2015 Apr 29. PMID: 25921703.	No drug
819	Velligan DI, Roberts D, Mintz J, Maples N, Li X, Medellin E, Brown M. A randomized pilot study of MOtiVation and Enhancement (MOVE) Training for negative symptoms in schizophrenia. Schizophr Res. 2015;165(2-3):175-80. doi: 10.1016/j.schres.2015.04.008. Epub 2015 May 1. PMID: 25937461; PMCID: PMC4484604.	No drug
820	Kurimori M, Shiozawa P, Bikson M, Aboseria M, Cordeiro Q. Targeting negative symptoms in schizophrenia: results from a proof-of-concept trial assessing prefrontal anodic tDCS protocol. Schizophr Res. 2015;166(1-3):362-3. doi: 10.1016/j.schres.2015.05.029. Epub 2015 May 29. PMID: 26032568.	No drug
821	Chan RCK, Geng FL, Lui SSY, Wang Y, Ho KKY, Hung KSY, Gur RE, Gur RC, Cheung EFC. Course of neurological soft signs in first-episode schizophrenia: Relationship with negative symptoms and cognitive performances. Sci Rep. 2015;5:11053. doi: 10.1038/srep11053. PMID: 26053141; PMCID: PMC4459190.	No drug
822	Vaskinn A, Ventura J, Andreassen OA, Melle I, Sundet K. A social path to functioning in schizophrenia: From social self-efficacy through negative symptoms to social functional capacity. Psychiatry Res. 2015;228(3):803-7. doi: 10.1016/j.psychres.2015.05.019. Epub 2015 May 29. PMID: 26089018.	No drug
823	Reckless GE, Andreassen OA, Server A, Østefjells T, Jensen J. Negative symptoms in schizophrenia are associated with aberrant striato-cortical connectivity in a rewarded perceptual decision-making task. Neuroimage Clin. 2015;8:290-7. doi: 10.1016/j.nicl.2015.04.025. PMID: 26106553; PMCID: PMC4474284.	No drug
824	Tsapakis EM, Dimopoulou T, Tarazi FI. Clinical management of negative symptoms of schizophrenia: An update. Pharmacol Ther. 2015;153:135-47. doi: 10.1016/j.pharmthera.2015.06.008. Epub 2015 Jun 24. PMID: 26116809.	Review
825	Luther L, Fukui S, Firmin RL, McGuire AB, White DA, Minor KS, Salyers MP. Expectancies of success as a predictor of negative symptoms reduction over 18 months in individuals with schizophrenia. Psychiatry Res. 2015;229(1-2):505-10. doi: 10.1016/j.psychres.2015.06.022. Epub 2015 Jun 28. PMID: 26162662.	No drug
826	Reddy LF, Horan WP, Green MF. Motivational deficits and negative symptoms in schizophrenia: Concepts and assessments. Curr Top Behav Neurosci. 2016;27:357-73. doi: 10.1007/7854_2015_379. PMID: 26164592.	Review
827	Lysaker PH, Kukla M, Dubreucq J, Gumley A, McLeod H, Vohs JL, Buck KD, Minor KS, Luther L, Leonhardt BL, Belanger EA, Popolo R, Dimaggio G. Metacognitive deficits predict future levels of negative symptoms in schizophrenia controlling for neurocognition, affect recognition, and self- expectation of goal attainment. Schizophr Res. 2015;168(1-2):267-72. doi: 10.1016/j.schres.2015.06.015. Epub 2015 Jul 9. PMID: 26164820.	No drug
828	Dlabac-de Lange JJ, Liemburg EJ, Bais L, Renken RJ, Knegtering H, Aleman A. Effect of rTMS on brain activation in schizophrenia with negative symptoms: A proof-of-principle study. Schizophr Res. 2015;168(1-2):475-82. doi: 10.1016/j.schres.2015.06.018. Epub 2015 Jul 14. PMID: 26187147.	No drug
829	Chen YH, Stone-Howell B, Edgar JC, Huang M, Wootton C, Hunter MA, Lu BY, Sadek JR, Miller GA, Cañive JM. Frontal slow-wave activity as a predictor of negative symptoms, cognition and functional capacity in schizophrenia. Br J Psychiatry. 2016;208(2):160-7. doi: 10.1192/bjp.bp.114.156075. Epub 2015 Jul 23. PMID: 26206861; PMCID: PMC4837382.	No drug
830	Kalin M, Kaplan S, Gould F, Pinkham AE, Penn DL, Harvey PD. Social cognition, social competence, negative symptoms and social outcomes: Inter- relationships in people with schizophrenia. J Psychiatr Res. 2015;68:254-60. doi: 10.1016/j.jpsychires.2015.07.008. Epub 2015 Jul 13. PMID: 26228427; PMCID: PMC4524806.	No drug
831	Tabak NT, Horan WP, Green MF. Mindfulness in schizophrenia: Associations with self-reported motivation, emotion regulation, dysfunctional attitudes, and negative symptoms. Schizophr Res. 2015;168(1-2):537-42. doi: 10.1016/j.schres.2015.07.030. Epub 2015 Jul 29. PMID: 26232242; PMCID: PMC4591204.	No drug
832	Blanchard JJ, Park SG, Catalano LT, Bennett ME. Social affiliation and negative symptoms in schizophrenia: Examining the role of behavioral skills and subjective responding. Schizophr Res. 2015;168(1-2):491-7. doi: 10.1016/j.schres.2015.07.019. Epub 2015 Jul 31. PMID: 26235753; PMCID: PMC4762010.	No drug
833	Urban-Kowalczyk M, Pigońska J, Śmigielski J. Pain perception in schizophrenia: influence of neuropeptides, cognitive disorders, and negative symptoms. Neuropsychiatr Dis Treat. 2015;11:2023-31. doi: 10.2147/NDT.S87666. PMID: 26273205; PMCID: PMC4532169.	No drug
834	Gomes JS, Shiozawa P, Dias ÁM, Valverde Ducos D, Akiba H, Trevizol AP, Bikson M, Aboseria M, Gadelha A, de Lacerda AL, Cordeiro Q. Left dorsolateral prefrontal cortex anodal tDCS effects on negative symptoms in schizophrenia. Brain Stimul. 2015;8(5):989-91. doi: 10.1016/j.brs.2015.07.033. Epub 2015 Aug 3. PMID: 26279407.	No drug

835	Canuti M, van Beveren NJ, Jazaeri Farsani SM, de Vries M, Deijs M, Jebbink MF, Zaaijer HL, van Schaik BD, van Kampen AH, van der Kuyl AC, de Haan L, Storosum JG, van der Hoek L. Viral metagenomics in drug-naïve, first-onset schizophrenia patients with prominent negative symptoms. Psychiatry Res. 2015;229(3):678-84. doi: 10.1016/j.psychres.2015.08.025. Epub 2015 Aug 15. PMID: 26304023.	No drug
836	Edwards AC, Bigdeli TB, Docherty AR, Bacanu S, Lee D, de Candia TR, Moscati A, Thiselton DL, Maher BS, Wormley BK; Molecular Genetics of Schizophrenia Collaboration (MGS); Walsh D, O'Neill FA, Kendler KS, Riley BP, Fanous AH. Meta- analysis of positive and negative symptoms reveals schizophrenia modifier genes. Schizophr Bull. 2016;42(2):279-87. doi: 10.1093/schbul/sbv119. Epub 2015 Aug 27. PMID: 26316594; PMCID: PMC4753595.	Review
837	Schmitt A, Falkai P. Negative symptoms and therapy strategies in schizophrenia. Eur Arch Psychiatry Clin Neurosci. 2015;265(7):541-2. doi: 10.1007/s00406-015-0637-6. PMID: 26319528.	Opinion
838	Lee SY, Chen SL, Chang YH, Chen PS, Huang SY, Tzeng NS, Wang LJ, Lee IH, Wang TY, Chen KC, Yang YK, Hong JS, Lu RB. ALDH2 polymorphism, associated with attenuating negative symptoms in patients with schizophrenia treated with add-on dextromethorphan. J Psychiatr Res. 2015;69:50-6. doi: 10.1016/j.jpsychires.2015.07.027. Epub 2015 Jul 26. PMID: 26343594.	Unfocused
839	Patel R, Jayatilleke N, Broadbent M, Chang CK, Foskett N, Gorrell G, Hayes RD, Jackson R, Johnston C, Shetty H, Roberts A, McGuire P, Stewart R. Negative symptoms in schizophrenia: a study in a large clinical sample of patients using a novel automated method. BMJ Open. 2015;5(9):e007619. doi: 10.1136/bmjopen-2015-007619. PMID: 26346872; PMCID: PMC4577949.	No drug
840	Kantrowitz JT, Woods SW, Petkova E, Cornblatt B, Corcoran CM, Chen H, Silipo G, Javitt DC. D-serine for the treatment of negative symptoms in individuals at clinical high risk of schizophrenia: a pilot, double-blind, placebo-controlled, randomised parallel group mechanistic proof-of-concept trial. Lancet Psychiatry. 2015;2(5):403-412. doi: 10.1016/S2215-0366(15)00098-X. Epub 2015 Apr 28.	**Included**
841	Hager OM, Kirschner M, Bischof M, Hartmann-Riemer MN, Kluge A, Seifritz E, Tobler PN, Kaiser S. Reward-dependent modulation of working memory is associated with negative symptoms in schizophrenia. Schizophr Res. 2015;168(1-2):238-44. doi: 10.1016/j.schres.2015.08.024. Epub 2015 Sep 9. PMID: 26362736.	No drug
842	Franza F, Fasano V, De Guglielmo S, Solomita B. Management of primary negative symptoms in schizophrenia: an one-year observational study. Psychiatr Danub. 2015;27(Suppl 1):S245-9. PMID: 26417773.	Open
843	Hasan A, Guse B, Cordes J, Wölwer W, Winterer G, Gaebel W, Langguth B, Landgrebe M, Eichhammer P, Frank E, Hajak G, Ohmann C, Verde PE, Rietschel M, Ahmed R, Honer WG, Malchow B, Karch S, Schneider-Axmann T, Falkai P, Wobrock T. Cognitive effects of high-frequency rTMS in schizophrenia patients with predominant negative symptoms: Results from a multicenter randomized sham- controlled trial. Schizophr Bull. 2016;42(3):608-18. doi: 10.1093/schbul/sbv142. Epub 2015 Oct 3. PMID: 26433217; PMCID: PMC4838079.	No drug
844	Bagney A, Dompablo M, Santabárbara J, Moreno-Ortega M, Lobo A, Jimenez- Arriero MA, Palomo T, Rodriguez-Jimenez R. Are negative symptoms really related to cognition in schizophrenia? Psychiatry Res. 2015;230(2):377-82. doi: 10.1016/j.psychres.2015.09.022. Epub 2015 Sep 14. PMID: 26454405.	No drug
845	Piras F, Schiff M, Chiapponi C, Bossù P, Mühlenhoff M, Caltagirone C, Gerardy-Schahn R, Hildebrandt H, Spalletta G. Brain structure, cognition and negative symptoms in schizophrenia are associated with serum levels of polysialic acid-modified NCAM. Transl Psychiatry. 2015;5(10):e658. doi: 10.1038/tp.2015.156. PMID: 26460482; PMCID: PMC4930132.	No drug
846	Granholm E, Ruiz I, Gallegos-Rodriguez Y, Holden J, Link PC. Pupillary responses as a biomarker of diminished effort associated with defeatist attitudes and negative symptoms in schizophrenia. Biol Psychiatry. 2016;80(8):581-8. doi: 10.1016/j.biopsych.2015.08.037. Epub 2015 Sep 15. PMID: 26475673; PMCID: PMC4792801.	No drug
847	Kimura H, Tanaka S, Kushima I, Koide T, Banno M, Kikuchi T, Nakamura Y, Shiino T, Yoshimi A, Oya-Ito T, Xing J, Wang C, Takasaki Y, Aleksic B, Okada T, Ikeda M, Inada T, Iidaka T, Iwata N, Ozaki N. Association study of BCL9 gene polymorphism rs583583 with schizophrenia and negative symptoms in Japanese population. Sci Rep. 2015;5:15705. doi: 10.1038/srep15705. PMID: 26494551; PMCID: PMC4616162.	No drug
848	Brusov OS, Zlobina GP. [Peculiarities of platelet activation changes in patients with chronic schizophrenia, depending on the severity of positive and negative symptoms during the remission formation]. Vestn Ross Akad Med Nauk. 2015;(3):355-9. Russian. doi: 10.15690/vramn.v70i3.1333. PMID: 26495725.	No drug
849	Ikebuchi E. [Negative symptoms revisited--toward the recovery of persons with schizophrenia]. Seishin Shinkeigaku Zasshi. 2015;117(3):179-94. Japanese. PMID: 26524844.	Opinion
850	Szkultecka-Dębek M, Walczak J, Augustyńska J, Miernik K, Stelmachowski J, Pieniążek I, Obrzut G, Pogroszewska A, Paulić G, Damir M, Antolić S, Tavčar R, Indrikson A, Aadamsoo K, Jankovic S, Pulay AJ, Rimay J, Varga M, Sulkova I, Veržun P. Epidemiology and treatment guidelines of negative symptoms in schizophrenia in Central and Eastern Europe: A literature review. Clin Pract Epidemiol Ment Health. 2015;11:158-65. doi: 10.2174/1745017901511010158. PMID: 26535049; PMCID: PMC4627386.	Review
851	Siegrist K, Millier A, Amri I, Aballéa S, Toumi M. Association between social contact frequency and negative symptoms, psychosocial functioning and quality of life in patients with schizophrenia. Psychiatry Res. 2015;230(3):860-6. doi: 10.1016/j.psychres.2015.11.039. Epub 2015 Nov 25. PMID: 26626950.	No drug
852	Neill JC, Grayson B, Kiss B, Gyertyán I, Ferguson P, Adham N. Effects of cariprazine, a novel antipsychotic, on cognitive deficit and negative symptoms in a rodent model of schizophrenia symptomatology. Eur Neuropsychopharmacol. 2016;26(1):3-14. doi: 10.1016/j.euroneuro.2015.11.016. Epub 2015 Nov 19. PMID: 26655189.	Animal
853	Engel M, Fritzsche A, Lincoln TM. Anticipation and experience of emotions in patients with schizophrenia and negative symptoms. An experimental study in a social context. Schizophr Res. 2016;170(1):191-7. doi: 10.1016/j.schres.2015.11.028. Epub 2015 Dec 7. PMID: 26673972.	No drug
854	Chen CY, Yeh YW, Kuo SC, Ho PS, Liang CS, Yen CH, Lu RB, Huang SY. Catechol-O-methyltransferase gene variants may associate with negative symptom response and plasma concentrations of prolactin in schizophrenia after amisulpride treatment. Psychoneuroendocrinology. 2016;65:67-75. doi: 10.1016/j.psyneuen.2015.12.003. Epub 2015 Dec 8. PMID: 26724569.	Unfocused
855	Sarkar S, Hillner K, Velligan DI. Conceptualization and treatment of negative symptoms in schizophrenia. World J Psychiatry. 2015;5(4):352-61. doi: 10.5498/wjp.v5.i4.352. PMID: 26740926; PMCID: PMC4694548.	Review
856	Strauss GP, Vertinski M, Vogel SJ, Ringdahl EN, Allen DN. Negative symptoms in bipolar disorder and schizophrenia: A psychometric evaluation of the brief negative symptom scale across diagnostic categories. Schizophr Res. 2016;170(2-3):285-9. doi: 10.1016/j.schres.2015.12.014. Epub 2015 Dec 29. PMID: 26742510.	No drug
857	Jahn DR, Bennett ME, Park SG, Gur RE, Horan WP, Kring AM, Blanchard JJ. The interactive effects of negative symptoms and social role functioning on suicide ideation in individuals with schizophrenia. Schizophr Res. 2016;170(2-3):271-7. doi: 10.1016/j.schres.2015.12.011. Epub 2015 Dec 30. PMID: 26746862; PMCID: PMC4762008.	No drug
858	McCarthy JM, Treadway MT, Bennett ME, Blanchard JJ. Inefficient effort allocation and negative symptoms in individuals with schizophrenia Schizophr Res. 2016;170(2-3):278-84. doi: 10.1016/j.schres.2015.12.017. Epub 2016 Jan 4. PMID: 26763628; PMCID: PMC4740196.	No drug
859	Engel M, Lincoln TM. Motivation and Pleasure Scale-Self-Report (MAP-SR): Validation of the German version of a self-report measure for screening negative symptoms in schizophrenia. Compr Psychiatry. 2016;65:110-5. doi: 10.1016/j.comppsych.2015.11.001. Epub 2015 Nov 17. PMID: 26773998.	No drug
860	Azorin J-M, Belzeaux R, Adida M. Les symptômes négatifs de la schizophrénie: quelles avancées? [Negative symptoms in schizophrenia: what progress?]. Encéphale. 2015;41(6 Suppl 1):6S1-2. French. doi: 10.1016/S0013-7006(16)30001-X. PMID: 26776384.	Opinion
861	Fakra E, Belzeaux R, Azorin J-M, Adida M. Symptômes négatifs, émotions et cognition dans la schizophrénie [Negative symptoms, emotion and cognition in schizophrenia]. Encéphale. 2015;41(6 Suppl 1):6S18-21. French. doi: 10.1016/S0013-7006(16)30005-7. PMID: 26776386.	Opinion

862	Simon N, Belzeaux R, Adida M, Azorin J-M. Symptômes négatifs dans la schizophrénie et addiction [Negative symptoms in schizophrenia and substance-related disorders]. Encéphale. 2015;41(6 Suppl 1):6S27-31. French. doi: 10.1016/S0013-7006(16)30007-0. PMID: 26776388.	Review
863	Pringuey D, Paquin N, Cherikh F, Giordana B, Belzeaux R, Cermolacce M, Adida M, Azorin J-M. Les symptômes négatifs de la schizophrénie aspects historiques [Negative symptoms of schizophrenia: historical aspects]. Encéphale. 2015;41(6 Suppl 1):6S3-8. French. doi: 10.1016/S0013-7006(16)30002-1. PMID: 26776389.	Review
864	Palomba A, Lodovighi M-A, Belzeaux R, Adida M, Azorin J-M. Utilisation des antidépresseurs dans le traitement des symptômes négatifs de la schizophrénie [Use of antidepressants in the treatment of negative symptoms of schizophrenia]. Encéphale. 2015;41(6 Suppl 1):6S36-40. French. doi: 10.1016/S0013-7006(16)30009-4. PMID: 26776391.	Review
865	Lodovighi MA, Palomba A, Belzeaux R, Adida M, Azorin J-M. Symptômes négatifs de la schizophrénie: nouvelles approches pharmacologiques [Negative symptoms in schizophrenia: new pharmacological approaches]. Encéphale. 2015;41(6 Suppl 1):6S41-9. French. doi: 10.1016/S0013-7006(16)30010-0. PMID: 26776392.	Review
866	Micoulaud Franchi JA, Quiles C, Belzeaux R, Adida M, Azorin J-M. Symptômes négatifs de la schizophrénie: de l'électrophysiologie à l'électrothérapie [Negative symptoms of schizophrenia: from electrophysiology to electrotherapy]. Encéphale. 2015;41(6 Suppl 1):6S50-6. French. doi: 10.1016/S0013-7006(16)30011-2. PMID: 26776393.	Review
867	Azorin J-M, Adida M, Belzeaux R, Pringuey D, Micoulaud Franchi J-A, Simon N, Cermolacce M, Kaladjian A, Fakra E. Symptômes négatifs de la schizophrénie: approches psychothérapeutiques [Negative symptoms in schizophrenia: psychotherapeutic approaches]. Encéphale. 2015;41(6 Suppl 1):6S57-60. French. doi: 10.1016/S0013-7006(16)30012-4. PMID: 26776394.	Review
868	Cermolacce M, Belzeaux R, Pringuey D, Adida M, Azorin JM. Aspects psychopathologiques des signes négatifs dans la schizophrénie [Psychopathological aspects of negative symptoms in schizophrenia]. Encéphale. 2015;41(6 Suppl 1):6S9-14. French. doi: 10.1016/S0013-7006(16)30003-3. PMID: 26776395.	Review
869	Jang SK, Park SC, Lee SH, Cho YS, Choi KH. Attention and memory bias to facial emotions underlying negative symptoms of schizophrenia. Cogn Neuropsychiatry. 2016;21(1):45-59. doi: 10.1080/13546805.2015.1127222. Epub 2016 Jan 20. PMID: 26786812.	No drug
870	Harvey RC, James AC, Shields GE. A systematic review and network meta-analysis to assess the relative efficacy of antipsychotics for the treatment of positive and negative symptoms in early-onset schizophrenia. CNS Drugs. 2016;30(1):27-39. doi: 10.1007/s40263-015-0308-1. PMID: 26801655.	Review
871	Nikiforuk A, Hołuj M, Kos T, Popik P. The effects of a 5-HT5A receptor antagonist in a ketamine-based rat model of cognitive dysfunction and the negative symptoms of schizophrenia. Neuropharmacology. 2016;105:351-360. doi: 10.1016/j.neuropharm.2016.01.035. Epub 2016 Jan 28. PMID: 26826431.	Animal
872	Gallagher BJ III, Jones BJ. Neglect and hereditary risk: Their relative contribution to schizophrenia with negative symptomatology. Int J Soc Psychiatry. 2016;62(3):235-42. doi: 10.1177/0020764015623974. Epub 2016 Feb 3. PMID: 26842730.	No drug
873	Iranpour N, Zandifar A, Farokhnia M, Goguol A, Yekehtaz H, Khodaie-Ardakani MR, Salehi B, Esalatmanesh S, Zeionoddini A, Mohammadinejad P, Zeinoddini A, Akhondzadeh S. The effects of pioglitazone adjuvant therapy on negative symptoms of patients with chronic schizophrenia: a double-blind and placebo-controlled trial. Hum Psychopharmacol. 2016;31(2):103-12. doi: 10.1002/hup.2517. Epub 2016 Feb 8. PMID: 26856695.	**Included**
874	Priebe S, Savill M, Wykes T, Bentall R, Lauber C, Reininghaus U, McCrone P, Mosweu I, Bremner S, Eldridge S, Röhricht F; NESS team. Clinical effectiveness and cost-effectiveness of body psychotherapy in the treatment of negative symptoms of schizophrenia: a multicentre randomised controlled trial. Health Technol Assess. 2016;20(11):vii-xxiii, 1-100. doi: 10.3310/hta20110. PMID: 26869182; PMCID: PMC4782808.	No drug
875	Nikbakhat MR, Arabzadeh S, Zeinoddini A, Khalili Z, Rezaei F, Mohammadinejad P, Ghaleiha A, Akhondzadeh S. Duloxetine add-on to risperidone for treatment of negative symptoms in patients with stable schizophrenia: Randomized double-blind placebo-controlled study. Pharmacopsychiatry. 2016;49(4):162-9. doi: 10.1055/s-0042-101557. Epub 2016 Feb 22. PMID: 26902281.	**Included**
876	Mach C, Dollfus S. Symptômes négatifs de la schizophrénie: une revue des instruments d'évaluation [Scale for assessing negative symptoms in schizophrenia: A systematic review]. Encéphale. 2016;42(2):165-71. French. doi: 10.1016/j.encep.2015.12.020. Epub 2016 Feb 26. PMID: 26923997.	Review
877	Campellone TR, Sanchez AH, Kring AM. Defeatist performance beliefs, negative symptoms, and functional outcome in schizophrenia: A meta-analytic review. Schizophr Bull. 2016;42(6):1343-1352. doi: 10.1093/schbul/sbw026. Epub 2016 Mar 15. PMID: 26980144; PMCID: PMC5049520.	Review
878	James S, Kapugama C, Al-Uzri M. Use of aripiprazole long acting injection in negative symptoms of schizophrenia. Case Rep Psychiatry. 2016;2016:7912083. doi: 10.1155/2016/7912083. Epub 2016 Feb 15. Erratum in: Case Rep Psychiatry. 2019 Sep 16;2019:9595638. PMID: 26981301; PMCID: PMC4770118.	Case
879	Keil J, Roa Romero Y, Balz J, Henjes M, Senkowski D. Positive and negative symptoms in schizophrenia relate to distinct oscillatory signatures of sensory gating. Front Hum Neurosci. 2016;10:104. doi: 10.3389/fnhum.2016.00104. Erratum in: Front Hum Neurosci. 2016;10:162. PMID: 27014035; PMCID: PMC4789458.	No drug
880	Bernardini F, Lunden A, Covington M, Broussard B, Halpern B, Alolayan Y, Crisafio A, Pauselli L, Balducci PM, Capulong L, Attademo L, Lucarini E, Salierno G, Natalicchi L, Quartesan R, Compton MT. Associations of acoustically measured tongue/jaw movements and portion of time speaking with negative symptom severity in patients with schizophrenia in Italy and the United States. Psychiatry Res. 2016;239:253-8. doi: 10.1016/j.psychres.2016.03.037. Epub 2016 Mar 25. PMID: 27039009.	No drug
881	Martin LA, Koch SC, Hirjak D, Fuchs T. Overcoming disembodiment: The effect of movement therapy on negative symptoms in schizophrenia-A multicenter randomized controlled trial. Front Psychol. 2016;7:483. doi: 10.3389/fpsyg.2016.00483. PMID: 27064347; PMCID: PMC4815039.	No drug
882	Barnes TR, Leeson VC, Paton C, Costelloe C, Simon J, Kiss N, Osborn D, Killaspy H, Craig TK, Lewis S, Keown P, Ismail S, Crawford M, Baldwin D, Lewis G, Geddes J, Kumar M, Pathak R, Taylor S. Antidepressant Controlled Trial For Negative Symptoms In Schizophrenia (ACTIONS): a double-blind, placebo- controlled, randomised clinical trial. Health Technol Assess. 2016;20(29):1-46. doi: 10.3310/hta20290. PMID: 27094189; PMCID: PMC4860560.	**Included**
883	Bian Y, Wang ZX, Han XL, Chen L, Zhu Y, Wu CJ. Sleep state misperception in schizophrenia: Are negative symptoms at work? Compr Psychiatry. 2016;67:33-8. doi: 10.1016/j.comppsych.2016.02.008. Epub 2016 Feb 18. PMID: 27095332.	No drug
884	Palm U, Keeser D, Hasan A, Kupka MJ, Blautzik J, Sarubin N, Kaymakanova F, Unger I, Falkai P, Meindl T, Ertl-Wagner B, Padberg F. Prefrontal transcranial direct current stimulation for treatment of schizophrenia with predominant negative symptoms: A double-blind, sham-controlled proof-of-concept study. Schizophr Bull. 2016;42(5):1253-61. doi: 10.1093/schbul/sbw041. Epub 2016 Apr 20. PMID: 27098066; PMCID: PMC4988747.	No drug
885	An der Heiden W, Leber A, Häfner H. Negative symptoms and their association with depressive symptoms in the long-term course of schizophrenia. Eur Arch Psychiatry Clin Neurosci. 2016;266(5):387-96. doi: 10.1007/s00406-016-0697-2. Epub 2016 Apr 23. PMID: 27107764.	No drug
886	Jha S, Garg A. Add on testosterone therapy in negative symptoms of schizophrenia with gonadal trauma: Hitting the bull's eye. Psychiatry Res. 2016;240:265-266. doi: 10.1016/j.psychres.2016.04.066. Epub 2016 Apr 21. PMID: 27138816.	Case
887	Li Z, Yin M, Lyu XL, Zhang LL, Du XD, Hung GC. Delayed effect of repetitive transcranial magnetic stimulation (rTMS) on negative symptoms of schizophrenia: Findings from a randomized controlled trial. Psychiatry Res. 2016;240:333-335. doi: 10.1016/j.psychres.2016.04.046. Epub 2016 Apr 22. PMID: 27138827.	No drug

888	Keil J, Romero YR, Balz J, Henjes MS, Senkowski D. Corrigendum: Positive and negative symptoms in schizophrenia relate to distinct oscillatory signatures of sensory gating. Front Hum Neurosci. 2016;10:162. doi: 10.3389/fnhum.2016.00162. Erratum for: Front Hum Neurosci 2016;10:104. PMID: 27148017; PMCID: PMC4830825.	Duplicate= 879
889	Priebe S, Savill M, Wykes T, Bentall RP, Reininghaus U, Lauber C, Bremner S, Eldridge S, Röhricht F. Effectiveness of group body psychotherapy for negative symptoms of schizophrenia: multicentre randomised controlled trial. Br J Psychiatry. 2016;209(1):54-61. doi: 10.1192/bjp.bp.115.171397. Epub 2016 May 5. PMID: 27151073; PMCID: PMC4929407.	No drug
890	Galderisi S, Färden A, Kaiser S. Dissecting negative symptoms of schizophrenia: History, assessment, pathophysiological mechanisms and treatment. Schizophr Res. 2017;186:1-2. doi: 10.1016/j.schres.2016.04.046. Epub 2016 May 13. PMID: 27185482.	No drug
891	Kallel L, Mondino M, Brunelin J. Effects of theta-rhythm transcranial alternating current stimulation (4.5 Hz-tACS) in patients with clozapine-resistant negative symptoms of schizophrenia: a case series. J Neural Transm (Vienna). 2016;123(10):1213-7. doi: 10.1007/s00702-016-1574-x Epub 2016 May 19. PMID: 27194229.	Case
892	Fernandez-Egea E, Vértes PE, Flint SM, Turner L, Mustafa S, Hatton A, Smith KG, Lyons PA, Bullmore ET. Peripheral immune cell populations associated with cognitive deficits and negative symptoms of treatment-resistant schizophrenia. PLoS One. 2016;11(5):e0155631. doi: 10.1371/journal.pone.0155631. PMID: 27244229; PMCID: PMC4887013.	No drug
893	Kim JS, Jang SK, Park SC, Yi JS, Park JK, Lee JS, Choi KH, Lee SH. Measuring negative symptoms in patients with schizophrenia: reliability and validity of the Korean version of the Motivation and Pleasure Scale-Self-Report. Neuropsychiatr Dis Treat. 2016;12:1167-72. doi: 10.2147/NDT.S107775. PMID: 27274251; PMCID: PMC4869782.	No drug
894	Bolu A, Oznur T, Tok D, Balikci A, Sener K, Celik C, Gulsun M. Seropositivity of neurotropic infectious agents in first-episode schizophrenia patients and the relationship with positive and negative symptoms. Psychiatr Danub. 2016;28(2):132-8. PMID: 27287787.	No drug
895	Cruz BF, Resende CB, Carvalhaes CF, Cardoso CS, Teixeira AL, Keefe RS, Rocha FL, Salgado JV. Interview-based assessment of cognition is a strong predictor of quality of life in patients with schizophrenia and severe negative symptoms. Braz J Psychiatry. 2016;38(3):216-21. doi: 10.1590/1516-4446-2015-1776. Epub 2016 Jun 14. PMID: 27304257; PMCID: PMC7194260.	No drug
896	Sahin C, Doostdar N, Neill JC. Towards the development of improved tests for negative symptoms of schizophrenia in a validated animal model. Behav Brain Res. 2016;312:93-101. doi: 10.1016/j.bbr.2016.06.021. Epub 2016 Jun 14. PMID: 27312268.	Animal
897	Mao Q, Tan YL, Luo XG, Tian L, Wang ZR, Tan SP, Chen S, Yang GG, An HM, Yang FD, Zhang XY. Association of catechol-O-methyltransferase Val108/158 Met genetic polymorphism with schizophrenia, P50 sensory gating, and negative symptoms in a Chinese population. Psychiatry Res. 2016;242:271-276. doi: 10.1016/j.psychres.2016.04.029. Epub 2016 Apr 14. PMID: 27315458.	No drug
898	Savill M, Orfanos S, Reininghaus U, Wykes T, Bentall R, Priebe S. The relationship between experiential deficits of negative symptoms and subjective quality of life in schizophrenia. Schizophr Res. 2016;176(2-3):387-391. doi: 10.1016/j.schres.2016.06.017. Epub 2016 Jun 18. PMID: 27328889.	No drug
899	Gan J, Duan H, Chen Z, Shi Z, Gao C, Zhu X, Liang X. [Effectiveness and safety of high dose transcranial magnetic stimulation in schizophrenia with refractory negative symptoms: a randomized controlled study]. Zhonghua Yi Xue Za Zhi. 2015;95(47):3808-12. Chinese. PMID: 27337795.	No drug
900	Jung SI, Woo J, Kim YT, Kwak SG. Validation of the Korean-Version of the Clinical Assessment Interview for Negative Symptoms of Schizophrenia (CAINS). J Korean Med Sci. 2016;31(7):1114-20. doi: 10.3346/jkms.2016.31.7.1114. Epub 2016 May 16. PMID: 27366011; PMCID: PMC4901005.	No drug
901	Remington G, Foussias G, Fervaha G, Agid O, Takeuchi H, Lee J, Hahn M. Treating negative symptoms in schizophrenia: an update. Curr Treat Options Psychiatry. 2016;3:133-150. doi: 10.1007/s40501-016-0075-8. Epub 2016 Apr 8. PMID: 27376016; PMCID: PMC4908169.	Review
902	Dollfus S, Lyne J. Negative symptoms: History of the concept and their position in diagnosis of schizophrenia. Schizophr Res. 2017;186:3-7. doi: 10.1016/j.schres.2016.06.024. Epub 2016 Jul 8. PMID: 27401529.	No drug
903	Mantovani LM, Ferretjans R, Marçal IM, Oliveira AM, Guimarães FC, Salgado JV. Family burden in schizophrenia: the influence of age of onset and negative symptoms. Trends Psychiatry Psychother. 2016;38(2):96-9. doi: 10.1590/2237-6089-2015-0082. PMID: 27409135.	No drug
904	Labad J, Martorell L, Huerta-Ramos E, Cobo J, Vilella E, Rubio-Abadal E, Garcia-Pares G, Creus M, Núñez C, Ortega L, Miquel E; RALOPSYCAT Group; Usall J. Pharmacogenetic study of the effects of raloxifene on negative symptoms of postmenopausal women with schizophrenia: A double-blind, randomized, placebo-controlled trial. Eur Neuropsychopharmacol. 2016;26(10):1683-9. doi: 10.1016/j.euroneuro.2016.08.006. Epub 2016 Aug 18. PMID: 27546373.	**Included**
905	Fervaha G, Caravaggio F, Mamo DC, Mulsant BH, Pollock BG, Nakajima S, Gerretsen P, Rajji TK, Mar W, Iwata Y, Plitman E, Chung JK, Remington G, Graff- Guerrero A. Lack of association between dopaminergic antagonism and negative symptoms in schizophrenia: a positron emission tomography dopamine D2/3 receptor occupancy study. Psychopharmacology (Berl). 2016;233(21-22):3803-3813. doi: 10.1007/s00213-016-4415-6. Epub 2016 Aug 24. PMID: 27557949; PMCID: PMC5065392.	No drug
906	Shaffer JJ, Peterson MJ, McMahon MA, Bizzell J, Calhoun V, van Erp TG, Ford JM, Lauriello J, Lim KO, Manoach DS, McEwen SC, Mathalon DH, O'Leary D, Potkin SG, Preda A, Turner J, Voyvodic J, Wible CG, Belger A. Neural correlates of schizophrenia negative symptoms: Distinct subtypes impact dissociable brain circuits. Mol Neuropsychiatry. 2015;1(4):191-200. doi: 10.1159/000440979. Epub 2015 Oct 17. PMID: 27606313; PMCID: PMC4996000.	No drug
907	Dlabac-de Lange JJ, Liemburg EJ, Bais L, van de Poel-Mustafayeva AT, de Lange-de Klerk ESM, Knegtering H, Aleman A. Effect of bilateral prefrontal rTMS on left prefrontal NAA and Glx levels in schizophrenia patients with predominant negative symptoms: An exploratory study. Brain Stimul. 2017;10(1):59-64. doi: 10.1016/j.brs.2016.08.002. Epub 2016 Aug 5. PMID: 27615793.	No drug
908	Clelland CL, Drouet V, Rilett KC, Smeed JA, Nadrich RH, Rajparia A, Read LL, Clelland JD. Evidence that COMT genotype and proline interact on negative- symptom outcomes in schizophrenia and bipolar disorder. Transl Psychiatry. 2016;6(9):e891. doi: 10.1038/tp.2016.157. PMID: 27622935; PMCID: PMC5048199.	No drug
909	Rossell SL, Francis PS, Galletly C, Harris A, Siskind D, Berk M, Bozaoglu K, Dark F, Dean O, Liu D, Meyer D, Neill E, Phillipou A, Sarris J, Castle DJ. N-acetylcysteine (NAC) in schizophrenia resistant to clozapine: a double blind randomised placebo controlled trial targeting negative symptoms. BMC Psychiatry. 2016;16(1):320. doi: 10.1186/s12888-016-1030-3. PMID: 27629871; PMCID: PMC5024434.	Protocol
910	Franza F, Carpentieri G, De Guglielmo S, Fasano V, Fiorentino N, Perito M, Solomita B, Del Buono G. Neurocognitive management of the primary negative symptoms of schizophrenia: a role of atypical antipsychotics. Psychiatr Danub. 2016;28(Suppl.1):145-148. PMID: 27663826.	Open
911	Mezquida G, Penadés R, Cabrera B, Savulich G, Lobo A, González-Pinto A, Penzol MJ, Corripio I, Fernandez-Egea E, Gassó P, Cuesta MJ, Bernardo M; PEPs group. Association of the brain-derived neurotrophic factor Val66Met polymorphism with negative symptoms severity, but not cognitive function, in first-episode schizophrenia spectrum disorders. Eur Psychiatry. 2016;38:61-69. doi: 10.1016/j.eurpsy.2016.04.011. Epub 2016 Sep 24. PMID: 27668551.	No drug
912	Galbusera L, Finn MT, Fuchs T. Interactional synchrony and negative symptoms: An outcome study of body-oriented psychotherapy for schizophrenia. Psychother Res. 2018;28(3):457-469. doi: 10.1080/10503307.2016.1216624. Epub 2016 Aug 12. PMID: 27687477.	No drug
913	Huang M, Huang Y, Yu L, Hu J, Chen J, Jin P, Xu W, Wei N, Hu S, Qi H, Xu Y. Relationship between negative symptoms and neurocognitive functions in adolescent and adult patients with first-episode schizophrenia. BMC Psychiatry. 2016;16(1):344. doi: 10.1186/s12888-016-1052-x. PMID: 27716115; PMCID: PMC5054618.	No drug
914	Hasan A, Wobrock T, Guse B, Langguth B, Landgrebe M, Eichhammer P, Frank E, Cordes J, Wölwer W, Musso F, Winterer G, Gaebel W, Hajak G, Ohmann C, Verde PE, Rietschel M, Ahmed R, Honer WG, Dechent P, Malchow B, Castro MFU, Dwyer D, Cabral C, Kreuzer PM, Poeppl TB, Schneider-Axmann T, Falkai P, Koutsouleris N. Structural brain changes are associated with response of negative symptoms to	No drug

	prefrontal repetitive transcranial magnetic stimulation in patients with schizophrenia. Mol Psychiatry. 2017;22(6):857-864. doi: 10.1038/mp.2016.161. Epub 2016 Oct 11. PMID: 27725655.	
915	Abram SV, Wisner KM, Fox JM, Barch DM, Wang L, Csernansky JG, MacDonald AW 3rd, Smith MJ. Fronto-temporal connectivity predicts cognitive empathy deficits and experiential negative symptoms in schizophrenia. Hum Brain Mapp. 2017;38(3):1111-1124. doi: 10.1002/hbm.23439. Epub 2016 Oct 24. PMID: 27774734; PMCID: PMC6866816.	No drug
916	Canga E, Puerto M, Ortega L, Solé M, Vilella E, Labad J, Martorell L. Parental antecedents of psychosis are associated with severity of positive and negative symptoms in schizophrenia patients. J Clin Psychiatry. 2016;77(9):1201-1202. doi: 10.4088/JCP.15l10241. PMID: 27780321.	No drug
917	Dunayevich E, Buchanan RW, Chen CY, Yang J, Nilsen J, Dietrich JM, Sun H, Marder S. Efficacy and safety of the glycine transporter type-1 inhibitor AMG 747 for the treatment of negative symptoms associated with schizophrenia. Schizophr Res. 2017;182:90-97. doi: 10.1016/j.schres.2016.10.027. Epub 2016 Oct 24. PMID: 27789188.	Non-marketed
918	Bliksted V, Videbech P, Fagerlund B, Frith C. The effect of positive symptoms on social cognition in first-episode schizophrenia is modified by the presence of negative symptoms. Neuropsychology. 2017;31(2):209-219. doi: 10.1037/neu0000309. Epub 2016 Nov 3. PMID: 27808537.	No drug
919	Akiyama K, Saito S, Saito A, Ozeki Y, Watanabe T, Fujii K, Honda G, Shimoda K. Predictive value of premorbid IQ, negative symptoms, and age for cognitive and social functions in Japanese patients with schizophrenia: A study using the Japanese version of the Brief Assessment of Cognition in Schizophrenia. Psychiatry Res. 2016;246:663-671. doi: 10.1016/j.psychres.2016.10.070. Epub 2016 Nov 1. PMID: 27838018.	No drug
920	Möller HJ. The relevance of negative symptoms in schizophrenia and how to treat them with psychopharmaceuticals? Psychiatr Danub. 2016;28(4):435-440. PMID: 27855437.	Opinion
921	Savill M, Orfanos S, Bentall R, Reininghaus U, Wykes T, Priebe S. The impact of gender on treatment effectiveness of body psychotherapy for negative symptoms of schizophrenia: A secondary analysis of the NESS trial data. Psychiatry Res. 2017;247:73-78. doi: 10.1016/j.psychres.2016.11.020. Epub 2016 Nov 14. PMID: 27871030; PMCID: PMC5191935.	No drug
922	Eisenstein SA, Bogdan R, Chen L, Moerlein SM, Black KJ, Perlmutter JS, Hershey T, Barch DM. Preliminary evidence that negative symptom severity relates to multilocus genetic profile for dopamine signaling capacity and D2 receptor binding in healthy controls and in schizophrenia. J Psychiatr Res. 2017;86:9-17. doi: 10.1016/j.jpsychires.2016.11.007. Epub 2016 Nov 17. PMID: 27886638; PMCID: PMC5272837.	No drug
923	Moran EK, Culbreth AJ, Barch DM. Ecological momentary assessment of negative symptoms in schizophrenia: Relationships to effort-based decision making and reinforcement learning. J Abnorm Psychol. 2017;126(1):96-105. doi: 10.1037/abn0000240. Epub 2016 Nov 28. PMID: 27893230; PMCID: PMC5433621.	No drug
924	Choi KH, Jaekal E, Lee GY. Motivational and behavioral activation as an adjunct to psychiatric rehabilitation for mild to moderate negative symptoms in individuals with schizophrenia: A proof-of-concept pilot study. Front Psychol. 2016;7:1759. doi: 10.3389/fpsyg.2016.01759. PMID: 27895602; PMCID: PMC5107574.	No drug
925	Lanfredi M, Deste G, Ferrari C, Barlati S, Magni LR, Rossi R, de Peri L, Bonomi M, Rossi G, Vita A. Effects of cognitive remediation therapy on neurocognition and negative symptoms in schizophrenia: an Italian naturalistic study. Cogn Neuropsychiatry. 2017;22(1):53-68. doi: 10.1080/13546805.2016.1260537. Epub 2016 Dec 6. PMID: 27921860.	No drug
926	Yoosefee S, Shahsavand Ananloo E, Joghataei MT, Karimipour M, Hadjighassem M, Mohaghghegh H, Tehrani-Doost M, Rahimi AA, Mostafavi Abdolmaleky H, Hatami M. Association between neuregulin-1 gene variant (rs2439272) and schizophrenia and its negative symptoms in an Iranian population. Iran J Psychiatry. 2016;11(3):147-153. PMID: 27928246; PMCID: PMC5139949.	No drug
927	Cella M, Preti A, Edwards C, Dow T, Wykes T. Cognitive remediation for negative symptoms of schizophrenia: A network meta-analysis. Clin Psychol Rev. 2017;52:43-51. doi: 10.1016/j.cpr.2016.11.009. Epub 2016 Nov 28. PMID: 27930934.	Review
928	Tajik-Esmaeeli S, Moazen-Zadeh E, Abbasi N, Shariat SV, Rezaei F, Salehi B, Akhondzadeh S. Simvastatin adjunct therapy for negative symptoms of schizophrenia: a randomized double-blind placebo-controlled trial. Int Clin Psychopharmacol. 2017;32(2):87-94. doi: 10.1097/YIC.0000000000000159. PMID: 27941358.	**Included**
929	Shiozawa P, Fasanella NA, Marinho C, Taiar I, Soares A, Calderoni DM, Ratto LRC, Cordeiro Q. Trigeminal nerve stimulation (TNS) for treating negative symptoms in schizophrenia: A case study. Schizophr Res. 2017;184:139-140. doi: 10.1016/j.schres.2016.11.036. Epub 2016 Dec 7. PMID: 27955801.	Case
930	Deserno L, Heinz A, Schlagenhauf F. Computational approaches to schizophrenia: A perspective on negative symptoms. Schizophr Res. 2017;186:46-54. doi: 10.1016/j.schres.2016.10.004. Epub 2016 Dec 13. PMID: 27986430.	Opinion
931	Akhondzadeh S, Moazen-Zadeh E. More ACTIONS needed to reach a consensus on adjunctive antidepressant therapy for negative symptoms of schizophrenia. Evid Based Ment Health. 2017;20(1):e3. doi: 10.1136/eb-2016-102500. Epub 2016 Dec 23. PMID: 28011708; PMCID: PMC10688420.	Opinion
932	Grin-Yatsenko VA, Ponomarev VA, Pronina MV, Poliakov YI, Plotnikova IV, Kropotov JD. Local and widely distributed EEG activity in schizophrenia with prevalence of negative symptoms. Clin EEG Neurosci. 2017;48(5):307-315. doi: 10.1177/1550059416683283. Epub 2017 Jan 5. PMID: 28056537.	No drug
933	Suresh Kumar PN, Anish PK, Rajmohan V. Olanzapine has better efficacy compared to risperidone for treatment of negative symptoms in schizophrenia. Indian J Psychiatry. 2016;58(3):311-316. doi: 10.4103/0019-5545.192016. PMID: 28066010; PMCID: PMC5100124.	**Included**
934	Blanchard JJ, Bradshaw KR, Garcia CP, Nasrallah HA, Harvey PD, Casey D, Csoboth CT, Hudson JI, Julian L, Lentz E, Nuechterlein KH, Perkins DO, Skale TG, Snowden LR, Tandon R, Tek C, Velligan D, Vinogradov S, O'Gorman C. Examining the reliability and validity of the Clinical Assessment Interview for Negative Symptoms within the Management of Schizophrenia in Clinical Practice (MOSAIC) multisite national study. Schizophr Res. 2017;185:137-143. doi: 10.1016/j.schres.2017.01.011. Epub 2017 Jan 11. PMID: 28087270.	No drug
935	Hirayasu Y, Sato SI, Shuto N, Nakano M, Higuchi T. Efficacy and safety of bitopertin in patients with schizophrenia and predominant negative symptoms: subgroup analysis of Japanese patients from the Global Randomized Phase 2 Trial. Psychiatry Investig. 2017;14(1):63-73. doi: 10.4306/pi.2017.14.1.63. Epub 2016 Dec 29. PMID: 28096877; PMCID: PMC5240458.	Non-marketed
936	Andrianarisoa M, Boyer L, Godin O, Brunel L, Bulzacka E, Aouizerate B, Berna F, Capdevielle D, Dorey JM, Dubertret C, Dubreucq J, Faget C, Gabayet F, Llorca PM, Mallet J, Misdrahi D, Rey R, Richieri R, Passerieux C, Schandrin A, Tronche AM, Urbach M, Vidailhet P, Schürhoff F, Fond G; FACE-SCZ Group. Childhood trauma, depression and negative symptoms are independently associated with impaired quality of life in schizophrenia. Results from the national FACE- SZ cohort. Schizophr Res. 2017;185:173-181. doi: 10.1016/j.schres.2016.12.021. Epub 2017 Jan 19. PMID: 28109668.	No drug
937	Bugarski-Kirola D, Blaettler T, Arango C, Fleischhacker WW, Garibaldi G, Wang A, Dixon M, Bressan RA, Nasrallah H, Lawrie S, Napieralski J, Ochi-Lohmann T, Reid C, Marder SR. Bitopertin in negative symptoms of schizophrenia-Results from the phase III FlashLyte and DayLyte studies. Biol Psychiatry. 2017;82(1):8-16. doi: 10.1016/j.biopsych.2016.11.014. Epub 2016 Dec 15. PMID: 28117049.	Non-marketed
938	Marder SR, Galderisi S. The current conceptualization of negative symptoms in schizophrenia. World Psychiatry. 2017;16(1):14-24. doi: 10.1002/wps.20385. PMID: 28127915; PMCID: PMC5269507.	Opinion
939	Leucht S, Davis JM. Schizophrenia, primary negative symptoms, and soft outcomes in psychiatry. Lancet. 2017;389(10074):1077-1078. doi: 10.1016/S0140-6736(17)30181-2. Epub 2017 Feb 7. PMID: 28185671.	Opinion
940	Németh G, Laszlovszky I, Czobor P, Szalai E, Szatmári B, Harsányi J, Barabássy Á, Debelle M, Durgam S, Bitter I, Marder S, Fleischhacker WW. Cariprazine versus risperidone monotherapy for treatment of predominant negative symptoms in patients with schizophrenia: a randomised, double-blind, controlled trial. Lancet. 2017;389(10074):1103-1113. doi: 10.1016/S0140-6736(17)30060-0. Epub 2017 Feb 7. Erratum in: Lancet. 2017;389(10074):1102. PMID: 28185672.	**Included**
941	Mueller DR, Khalesi Z, Benzing V, Castiglione CI, Roder V. Does Integrated Neurocognitive Therapy (INT) reduce severe negative symptoms in schizophrenia outpatients? Schizophr Res. 2017;188:92-97. doi: 10.1016/j.schres.2017.01.037. Epub 2017 Feb 6. PMID: 28185784.	No drug

942	Mezquida G, Cabrera B, Bioque M, Amoretti S, Lobo A, González-Pinto A, Espliego A, Corripio I, Vieta E, Castro-Fornieles J, Bergé D, Escartí MJ, Ibañez Á, Penadés R, Sánchez-Torres AM, Bernardo M; PEPs Group. The course of negative symptoms in first-episode schizophrenia and its predictors: A prospective two- year follow-up study. Schizophr Res. 2017;189:84-90. doi: 10.1016/j.schres.2017.01.047. Epub 2017 Feb 6. PMID: 28185786.	No drug
943	Novick D, Montgomery W, Treuer T, Moneta MV, Haro JM. Real-world effectiveness of antipsychotics for the treatment of negative symptoms in patients with schizophrenia with predominantly negative symptoms. Pharmacopsychiatry. 2017;50(2):56-63. doi: 10.1055/s-0042-112818. Epub 2017 Feb 16. PMID: 28208190.	*Post hoc*
944	Shenker JJ, Sengupta SM, Joober R, Malla A, Chakravarty MM, Lepage M. Bipolar disorder risk gene FOXO6 modulates negative symptoms in schizophrenia: a neuroimaging genetics study. J Psychiatry Neurosci. 2017;42(3):172-180. doi: 10.1503/jpn.150332. PMID: 28234206; PMCID: PMC5403662.	No drug
945	Saito Y, Kubicki M, Koerte I, Otsuka T, Rathi Y, Pasternak O, Bouix S, Eckbo R, Kikinis Z, von Hohenberg CC, Roppongi T, Del Re E, Asami T, Lee SH, Karmacharya S, Mesholam-Gately RI, Seidman LJ, Levitt J, McCarley RW, Shenton ME, Niznikiewicz MA. Impaired white matter connectivity between regions containing mirror neurons, and relationship to negative symptoms and social cognition, in patients with first-episode schizophrenia. Brain Imaging Behav. 2018;12(1):229-237. doi: 10.1007/s11682-017-9685-z. PMID: 28247157; PMCID: PMC5809316.	No drug
946	Lee SJ, Kim KR, Lee SY, An SK. Impaired Social and Role Function in Ultra- High Risk for Psychosis and First-Episode Schizophrenia: Its Relations with Negative Symptoms. Psychiatry Investig. 2017;14(2):186-192. doi: 10.4306/pi.2017.14.2.186. Epub 2017 Mar 6. PMID: 28326117; PMCID: PMC5355017.	No drug
947	Kantrowitz JT. Managing negative symptoms of schizophrenia: How far have we come? CNS Drugs. 2017;31(5):373-388. doi: 10.1007/s40263-017-0428-x. PMID: 28397113.	Review
948	Mezquida G, Savulich G, Garcia-Rizo C, Garcia-Portilla MP, Toll A, Garcia- Alvarez L, Bobes J, Mané A, Bernardo M, Fernandez-Egea E. Inverse association between negative symptoms and body mass index in chronic schizophrenia. Schizophr Res. 2018;192:69-74. doi: 10.1016/j.schres.2017.04.002. Epub 2017 Apr 12. PMID: 28412089.	No drug
949	Rezaei F, Mesgarpour B, Jeddian A, Zeionoddini A, Mohammadinejad P, Salardini E, Shahriari M, Zeinoddini A, Akhondzadeh S. Cilostazol adjunctive therapy in treatment of negative symptoms in chronic schizophrenia: Randomized, double-blind, placebo-controlled study. Hum Psychopharmacol. 2017;32(4):e2583. doi: 10.1002/hup.2583. Epub 2017 Apr 18. PMID: 28421639.	**Included**
950	Mitra S, Nizamie SH, Goyal N, Tikka SK. Electroencephalogram alpha-to-theta ratio over left fronto-temporal region correlates with negative symptoms in schizophrenia. Asian J Psychiatr. 2017;26:70-76. doi: 10.1016/j.ajp.2017.01.013. Epub 2017 Jan 21. PMID: 28483096.	No drug
951	Németh B, Molnár A, Akehurst R, Horváth M, Kóczián K, Németh G, Götze Á, Vokó Z. Quality-adjusted life year difference in patients with predominant negative symptoms of schizophrenia treated with cariprazine and risperidone. J Comp Eff Res. 2017;6(8):639-648. doi: 10.2217/cer-2017-0024. Epub 2017 May 17. PMID: 28511548.	Overlap
952	Schmidt SJ, Lange M, Schöttle D, Karow A, Schimmelmann BG, Lambert M. Negative symptoms, anxiety, and depression as mechanisms of change of a 12-month trial of assertive community treatment as part of integrated care in patients with first- and multi-episode schizophrenia spectrum disorders (ACCESS I trial). Eur Arch Psychiatry Clin Neurosci. 2018;268(6):593-602. doi: 10.1007/s00406-017-0810-1. Epub 2017 May 24. PMID: 28540411.	No drug
953	Walton E, Hibar DP, van Erp TGM, Potkin SG, Roiz-Santiañez R, Crespo- Facorro B, Suarez-Pinilla P, van Haren NEM, de Zwarte SMC, Kahn RS, Cahn W, Doan NT, Jørgensen KN, Gurholt TP, Agartz I, Andreassen OA, Westlye LT, Melle I, Berg AO, Morch-Johnsen L, Færden A, Flyckt L, Fatouros-Bergman H; Karolinska Schizophrenia Project Consortium (KaSP); Jönsson EG, Hashimoto R, Yamamori H, Fukunaga M, Jahanshad N, De Rossi P, Piras F, Banaj N, Spalletta G, Gur RE, Gur RC, Wolf DH, Satterthwaite TD, Beard LM, Sommer IE, Koops S, Gruber O, Richter A, Krämer B, Kelly S, Donohoe G, McDonald C, Cannon DM, Corvin A, Gill M, Di Giorgio A, Bertolino A, Lawrie S, Nickson T, Whalley HC, Neilson E, Calhoun VD, Thompson PM, Turner JA, Ehrlich S. Prefrontal cortical thinning links to negative symptoms in schizophrenia via the ENIGMA consortium. Psychol Med. 2018;48(1):82-94. doi: 10.1017/S0033291717001283. Epub 2017 May 26. PMID: 28545597; PMCID: PMC5826665.	No drug
954	Thiebes S, Leicht G, Curic S, Steinmann S, Polomac N, Andreou C, Eichler I, Eichler L, Zöllner C, Gallinat J, Hanganu-Opatz I, Mulert C. Glutamatergic deficit and schizophrenia-like negative symptoms: new evidence from ketamine- induced mismatch negativity alterations in healthy male humans. J Psychiatry Neurosci. 2017;42(4):273-283. doi: 10.1503/jpn.160187. PMID: 28556775; PMCID: PMC5487274.	Unfocused
955	Mota NB, Copelli M, Ribeiro S. Thought disorder measured as random speech structure classifies negative symptoms and schizophrenia diagnosis 6 months in advance. NPJ Schizophr. 2017;3:18. doi: 10.1038/s41537-017-0019-3. PMID: 28560264; PMCID: PMC5441540.	No drug
956	Barnes SA, Der-Avakian A, Young JW. Preclinical models to investigate mechanisms of negative symptoms in schizophrenia. Schizophr Bull. 2017;43(4):706-711. doi: 10.1093/schbul/sbx065. PMID: 28586462; PMCID: PMC5472160.	Opinion
957	Buchanan RW, Kelly DL, Weiner E, Gold JM, Strauss GP, Koola MM, McMahon RP, Carpenter WT. A randomized clinical trial of oxytocin or galantamine for the treatment of negative symptoms and cognitive impairments in people with schizophrenia. J Clin Psychopharmacol. 2017;37(4):394-400. doi: 10.1097/JCP.0000000000000720. PMID: 28590362; PMCID: PMC5484721.	**Included**
958	Llerena K, Reddy LF, Kern RS. The role of experiential and expressive negative symptoms on job obtainment and work outcome in individuals with schizophrenia. Schizophr Res. 2018;192:148-153. doi: 10.1016/j.schres.2017.06.001. Epub 2017 Jun 7. PMID: 28599750.	No drug
959	Uhl I, Kulik A, Roser P, Theodoridou A, Wyss C, Norra C, Brüne M, Kawohl W, Juckel G. Central serotonergic function in patients with predominantly negative symptoms of schizophrenia. Schizophr Res. 2018;193:443-444. doi: 10.1016/j.schres.2017.05.041. Epub 2017 Jun 20. PMID: 28641887.	No drug
960	Mehta UM, Ravishankar V, Thirthalli J. Eszopiclone for persistent negative symptoms in schizophrenia – An unintended N-of-1 study. Schizophr Res. 2018;193:438-440. doi: 10.1016/j.schres.2017.06.035. Epub 2017 Jun 21. PMID: 28647492.	Case
961	Mitra S, Mahintamani T, Kavoor AR, Nizamie SH. Negative symptoms in schizophrenia. Ind Psychiatry J. 2016;25(2):135-144. doi: 10.4103/ipj.ipj_30_15. PMID: 28659691; PMCID: PMC5479085.	Review
962	Bhandari SS, Bhagabati D. Prevalence of spontaneous dyskinesia in first episode, drug naive schizophrenia, and its relation to the positive and negative symptoms of schizophrenia. Open J Psychiatry Allied Sci. 2017;8(2):113-123. doi: 10.5958/2394-2061.2017.00005.2. Epub 2016 Dec 30. PMID: 31304241; PMCID: PMC6625795.	No drug
963	Xavier RM, Vorderstrasse A. Genetic basis of positive and negative symptom domains in schizophrenia. Biol Res Nurs. 2017;19(5):559-575. doi: 10.1177/1099800417715907. Epub 2017 Jul 10. PMID: 28691507.	Review
964	Popolo R, Smith E, Lysaker PH, Lestingi K, Cavallo F, Melchiorre L, Santone C, Dimaggio G. Metacognitive profiles in schizophrenia and bipolar disorder: Comparisons with healthy controls and correlations with negative symptoms. Psychiatry Res. 2017;257:45-50. doi: 10.1016/j.psychres.2017.07.022. Epub 2017 Jul 11. PMID: 28719831.	No drug
965	Liang Y, Yu X. Effectiveness of amisulpride in Chinese patients with predominantly negative symptoms of schizophrenia: a subanalysis of the ESCAPE study. Neuropsychiatr Dis Treat. 2017;13:1703-1712. doi: 10.2147/NDT.S140905. PMID: 28721051; PMCID: PMC5499925.	Open
966	Davidson M, Saoud J, Staner C, Noel N, Luthringer E, Werner S, Reilly J, Schaffhauser JY, Rabinowitz J, Weiser M, Luthringer R. Efficacy and safety of MIN-101: A 12-week randomized, double-blind, placebo-controlled trial of a new drug in development for the treatment of negative symptoms in schizophrenia. Am J Psychiatry. 2017;174(12):1195-1202. doi: 10.1176/appi.ajp.2017.17010122. Epub 2017 Jul 28. PMID: 28750582.	Non-marketed

967	Wang J, Zhou Y, Gan H, Pang J, Li H, Wang J, Li C. Efficacy towards negative symptoms and safety of repetitive transcranial magnetic stimulation treatment for patients with schizophrenia: A systematic review. Shanghai Arch Psychiatry. 2017;29(2):61-76. doi: 10.11919/j.issn.1002-0829.217024. PMID: 28765677; PMCID: PMC5518263.	Review
968	Wahid N, Kim JJ, Bota RG. Psychostimulant use in schizophrenia for treatment of negative symptoms and weight loss. Prim Care Companion CNS Disord. 2017;19(4):17l02093. doi: 10.4088/PCC.17l02093. PMID: 28767206.	Case
969	Veerman SRT, Schulte PFJ, de Haan L. Treatment for negative symptoms in schizophrenia: A comprehensive review. Drugs. 2017;77(13):1423-1459. doi: 10.1007/s40265-017-0789-y. PMID: 28776162.	Review
970	McCarthy JM, Bradshaw KR, Catalano LT, Garcia CP, Malik A, Bennett ME, Blanchard JJ. Negative symptoms and the formation of social affiliative bonds in schizophrenia. Schizophr Res. 2018;193:225-231. doi: 10.1016/j.schres.2017.07.034. Epub 2017 Aug 2. PMID: 28779852; PMCID: PMC5796871.	No drug
971	Vadlamani LN, Banwari G, Dinakaran D, Menon V, Andrade C. Olanzapine has poorer efficacy than risperidone for the treatment of the negative symptoms of schizophrenia. Indian J Psychiatry. 2017;59(2):248-249. doi: 10.4103/psychiatry.IndianJPsychiatry_95_17. PMID: 28827880; PMCID: PMC5547874.	Opinion
972	Neves G, Borsoi M, Antonio CB, Pranke MA, Betti AH, Rates SMK. Is forced swimming immobility a good endpoint for modeling negative symptoms of schizophrenia? - Study of sub-anesthetic ketamine repeated administration effects. An Acad Bras Cienc. 2017;89(3):1655-1669. doi: 10.1590/0001-3765201720160844. Epub 2017 Aug 17. PMID: 28832723.	Animal
973	Peralta V, de Jalón EG, Campos MS, Cuesta MJ. Covariation between motor signs and negative symptoms in drug-naive subjects with schizophrenia-spectrum disorders before and after antipsychotic treatment. Schizophr Res. 2018;200:85-91. doi: 10.1016/j.schres.2017.08.039. Epub 2017 Aug 30. PMID: 28864283.	No drug
974	Jin Y, Wang Q, Wang Y, Liu M, Sun A, Geng Z, Lin Y, Li X. Alpha7 nAChR agonists for cognitive deficit and negative symptoms in schizophrenia: A meta-analysis of randomized double-blind controlled trials. Shanghai Arch Psychiatry. 2017;29(4):191-199. doi: 10.11919/j.issn.1002-0829.217044. PMID: 28955138; PMCID: PMC5608991.	Review
975	Fujimaki K, Toki S, Yamashita H, Oyamada T, Yamawaki S. Predictors of negative symptoms in the chronic phase of schizophrenia: A cross-sectional study. Psychiatry Res. 2018;262:600-608. doi: 10.1016/j.psychres.2017.09.051. Epub 2017 Sep 24. PMID: 28965809.	No drug
976	Granholm E, Holden J, Worley M. improvement in negative symptoms and functioning in cognitive-behavioral social skills training for schizophrenia: Mediation by defeatist performance attitudes and asocial beliefs. Schizophr Bull. 2018;44(3):653-661. doi: 10.1093/schbul/sbx099. PMID: 29036391; PMCID: PMC5890456.	No drug
977	Veerman S, Schulte P, de Haan L. Memantine add-on to clozapine treatment for residual negative symptoms of schizophrenia. Psychopharmacology (Berl). 2017;234(23-24):3535-3536. doi: 10.1007/s00213-017-4756-9. Epub 2017 Oct 16. PMID: 29038824.	Opinion
978	Lee SJ, Kim KR, Lee SY, An SK. Impaired social and role function in ultra-high risk for psychosis and first-episode schizophrenia: Its relations with negative symptoms. Psychiatry Investig. 2017;14(5):539-545. doi: 10.4306/pi.2017.14.5.539. Epub 2017 Sep 11. PMID: 29042877; PMCID: PMC5639120.	No drug
979	Binford SS, Hubbard EM, Flowers E, Miller BL, Leutwyler H. Serum BDNF is positively associated with negative symptoms in older adults with schizophrenia. Biol Res Nurs. 2018;20(1):63-69. doi: 10.1177/1099800417735634. Epub 2017 Oct 19. PMID: 29050493; PMCID: PMC5942501.	No drug
980	Aleman A, Brummelman J, Dlabac-de Lange JJ, Koops S, Knegtering H, Neggers SFW, Sommer IE. Non-invasieve hersenstimulatie bij schizofrenie, gericht op hallucinaties en negatieve symptomen [Non-invasive brain stimulation in schizophrenia: hallucinations and negative symptoms]. Tijdschr Psychiatr. 2017;59(10):612-616. Dutch. PMID: 29077136.	No drug
981	Ventura J, Subotnik KL, Gretchen-Doorly D, Casaus L, Boucher M, Medalia A, Bell MD, Hellemann GS, Nuechterlein KH. Cognitive remediation can improve negative symptoms and social functioning in first-episode schizophrenia: A randomized controlled trial. Schizophr Res. 2019;203:24-31. doi: 10.1016/j.schres.2017.10.005. Epub 2017 Nov 9. PMID: 29128326; PMCID: PMC6589092.	No drug
982	Li M, Deng W, Das T, Li Y, Zhao L, Ma X, Wang Y, Yu H, Li X, Meng YJ, Wang Q, Palaniyappan L, Li T. Neural substrate of unrelenting negative symptoms in schizophrenia: a longitudinal resting-state fMRI study. Eur Arch Psychiatry Clin Neurosci. 2018;268(7):641-651. doi: 10.1007/s00406-017-0851-5. Epub 2017 Nov 11. PMID: 29128871.	No drug
983	Ødegaard Nielsen M, Rostrup E, Broberg BV, Wulff S, Glenthøj B. Negative symptoms and reward disturbances in schizophrenia before and after antipsychotic monotherapy. Clin EEG Neurosci. 2018;49(1):36-45. doi: 10.1177/1550059417744120. Epub 2017 Nov 17. PMID: 29145751.	Open
984	Ahmed AO, Strauss GP, Buchanan RW, Kirkpatrick B, Carpenter WT. Schizophrenia heterogeneity revisited: Clinical, cognitive, and psychosocial correlates of statistically-derived negative symptoms subgroups. J Psychiatr Res. 2018;97:8-15. doi: 10.1016/j.jpsychires.2017.11.004. Epub 2017 Nov 12. PMID: 29156414.	No drug
985	Yoshikawa A, Nishimura F, Inai A, Eriguchi Y, Nishioka M, Takaya A, Tochigi M, Kawamura Y, Umekage T, Kato K, Sasaki T, Ohashi Y, Iwamoto K, Kasai K, Kakiuchi C. Mutations of the glycine cleavage system genes possibly affect the negative symptoms of schizophrenia through metabolomic profile changes. Psychiatry Clin Neurosci. 2018;72(3):168-179. doi: 10.1111/pcn.12628. Epub 2018 Jan 31. PMID: 29232014.	No drug
986	İnce E, Üçok A. Relationship between persistent negative symptoms and findings of neurocognition and neuroimaging in schizophrenia. Clin EEG Neurosci. 2018;49(1):27-35. doi: 10.1177/1550059417746213. PMID: 29243526.	Review
987	Mørch-Johnsen L, Agartz I, Jensen J. The Neural Correlates of Negative Symptoms in Schizophrenia: Examples From MRI Literature. Clin EEG Neurosci. 2018;49(1):12-17. doi: 10.1177/1550059417746214. PMID: 29243527.	Review
988	Pontillo M, Costanzo F, Menghini D, Averna R, Santonastaso O, Tata MC, Vicari S. Use of transcranial direct stimulation in the treatment of negative symptoms of schizophrenia. Clin EEG Neurosci. 2018;49(1):18-26. doi: 10.1177/1550059417746531. PMID: 29243532.	Review
989	Bakirhan A, Yalcin Sahiner S, Sahiner IV, Safak Y, Goka E. Association of serum brain derived neurotropic factor with duration of drug-naive period and positive-negative symptom scores in drug naive schizophrenia. PLoS One. 2017;12(12):e0189373. doi: 10.1371/journal.pone.0189373. PMID: 29287075; PMCID: PMC5747443.	No drug
990	Granholm E, Harvey PD. Social skills training for negative symptoms of schizophrenia. Schizophr Bull. 2018;44(3):472-474. doi: 10.1093/schbul/sbx184. PMID: 29315427; PMCID: PMC5890477.	Opinion
991	Jagannath V, Theodoridou A, Gerstenberg M, Franscini M, Heekeren K, Correll CU, Rössler W, Grünblatt E, Walitza S. Prediction analysis for transition to schizophrenia in individuals at clinical high risk for psychosis: The relationship of DAO, DAOA, and NRG1 variants with negative symptoms and cognitive deficits. Front Psychiatry. 2017;8:292. doi: 10.3389/fpsyt.2017.00292. PMID: 29326614; PMCID: PMC5742321.	No drug
992	Farreny A, Usall J, Cuevas-Esteban J, Ochoa S, Brébion G. Amendment of traditional assessment measures for the negative symptoms of schizophrenia. Eur Psychiatry. 2018;49:50-55. doi: 10.1016/j.eurpsy.2017.11.003. Epub 2018 Feb 3. PMID: 29366848.	Unfocused
993	Krause M, Zhu Y, Huhn M, Schneider-Thoma J, Bighelli I, Nikolakopoulou A, Leucht S. Antipsychotic drugs for patients with schizophrenia and predominant or prominent negative symptoms: a systematic review and meta-analysis. Eur Arch Psychiatry Clin Neurosci. 2018;268(7):625-639. doi: 10.1007/s00406-018-0869-3. Epub 2018 Jan 24. PMID: 29368205.	Review
994	Fraguas D, Díaz-Caneja CM, Pina-Camacho L, Umbricht D, Arango C. Predictors of placebo response in pharmacological clinical trials of negative symptoms in schizophrenia: A meta-regression analysis. Schizophr Bull. 2019;45(1):57-68. doi: 10.1093/schbul/sbx192. Erratum in: Schizophr Bull. 2023;49(2):534-537. PMID: 29370436; PMCID: PMC6293224.	Review

995	Osoegawa C, Gomes JS, Grigolon RB, Brietzke E, Gadelha A, Lacerda ALT, Dias ÁM, Cordeiro Q, Laranjeira R, de Jesus D, Daskalakis ZJ, Brunelin J, Cordes J, Trevizol AP. Non-invasive brain stimulation for negative symptoms in schizophrenia: An updated systematic review and meta-analysis. Schizophr Res. 2018;197:34-44. doi: 10.1016/j.schres.2018.01.010. Epub 2018 Feb 4. PMID: 29397282.	Review
996	Boozalis T, Teixeira AL, Cho RY, Okusaga O. C-reactive protein correlates with negative symptoms in patients with schizophrenia. Front Public Health. 2018;5:360. doi: 10.3389/fpubh.2017.00360. PMID: 29404313; PMCID: PMC5786820.	No drug
997	Ghajar A, Khoaie-Ardakani MR, Shahmoradi Z, Alavi AR, Afarideh M, Shalbafan MR, Ghazizadeh-Hashemi M, Akhondzadeh S. L-carnosine as an add-on to risperidone for treatment of negative symptoms in patients with stable schizophrenia: A double-blind, randomized placebo-controlled trial. Psychiatry Res. 2018;262:94-101. doi: 10.1016/j.psychres.2018.02.012. Epub 2018 Feb 3. PMID: 29427913.	**Included**
998	Capatina OO, Miclutia IV. Are negative symptoms in schizophrenia a distinct therapeutic target? Clujul Med. 2018;91(1):58-64. doi: 10.15386/cjmed-864. Epub 2018 Jan 15. PMID: 29440953; PMCID: PMC5808269.	No drug
999	Aleman A, Enriquez-Geppert S, Knegtering H, Dlabac-de Lange JJ. Moderate effects of noninvasive brain stimulation of the frontal cortex for improving negative symptoms in schizophrenia: Meta-analysis of controlled trials. Neurosci Biobehav Rev. 2018;89:111-118. doi: 10.1016/j.neubiorev.2018.02.009. Epub 2018 Feb 19. PMID: 29471017.	Review
1000	Hansbauer M, Wobrock T, Kunze B, Langguth B, Landgrebe M, Eichhammer P, Frank E, Cordes J, Wölwer W, Winterer G, Gaebel W, Hajak G, Ohmann C, Verde PE, Rietschel M, Ahmed R, Honer WG, Malchow B, Strube W, Schneider-Axmann T, Falkai P, Hasan A. Efficacy of high-frequency repetitive transcranial magnetic stimulation on PANSS factors in schizophrenia with predominant negative symptoms – Results from an exploratory re-analysis. Psychiatry Res. 2018;263:22-29. doi: 10.1016/j.psychres.2018.02.030. Epub 2018 Feb 17. PMID: 29482042.	No drug
1001	Khan AH, Zaidi S. Clozapine: Improvement of negative symptoms of schizophrenia. Cureus. 2017;9(12):e1973. doi: 10.7759/cureus.1973. PMID: 29492362; PMCID: PMC5820006.	No drug
1002	Goldsmith DR, Haroon E, Miller AH, Strauss GP, Buckley PF, Miller BJ. TNF-α and IL-6 are associated with the deficit syndrome and negative symptoms in patients with chronic schizophrenia. Schizophr Res. 2018;199:281-284. doi: 10.1016/j.schres.2018.02.048. Epub 2018 Feb 28. PMID: 29499967; PMCID: PMC6111000.	No drug
1003	Liu F, Zhang B, Xie L, Ruan Y, Xu X, Zeng Y, Messina L, Zhao J, Fan X. Changes in plasma levels of nitric oxide metabolites and negative symptoms after 16-week minocycline treatment in patients with schizophrenia. Schizophr Res. 2018;199:390-394. doi: 10.1016/j.schres.2018.03.003. Epub 2018 Mar 9.	**Included**
1004	Krynicki CR, Upthegrove R, Deakin JFW, Barnes TRE. The relationship between negative symptoms and depression in schizophrenia: a systematic review. Acta Psychiatr Scand. 2018;137(5):380-390. doi: 10.1111/acps.12873. Epub 2018 Mar 13. PMID: 29532909.	Review
1005	Gomes JS, Trevizol AP, Ducos DV, Gadelha A, Ortiz BB, Fonseca AO, Akiba HT, Azevedo CC, Guimaraes LSP, Shiozawa P, Cordeiro Q, Lacerda A, Dias AM. Effects of transcranial direct current stimulation on working memory and negative symptoms in schizophrenia: a phase II randomized sham-controlled trial. Schizophr Res Cogn. 2018;12:20-28. doi: 10.1016/j.scog.2018.02.003.	No drug
1006	Galderisi S, Mucci A, Buchanan RW, Arango C. Negative symptoms of schizophrenia: new developments and unanswered research questions. Lancet Psychiatry. 2018;5(8):664-677. doi: 10.1016/S2215-0366(18)30050-6. Epub 2018 Mar 27. PMID: 29602739.	Review
1007	Vrbova K, Prasko J, Holubova M, Slepecky M, Ociskova M. Positive and negative symptoms in schizophrenia and their relation to depression, anxiety, hope, self-stigma and personality traits – a cross-sectional study. Neuro Endocrinol Lett. 2018;39(1):9-18. PMID: 29604619.	No drug
1008	Garcia-Alvarez L, Garcia-Portilla MP, Saiz PA, Fonseca-Pedrero E, Bobes- Bascaran MT, Gomar J, Muñiz J, Bobes J. Spanish validation of the Negative Symptom Assessment-16 (NSA-16) in patients with schizophrenia. Rev Psiquiatr Salud Ment (Engl Ed). 2018;11(3):169-175. English, Spanish. doi: 10.1016/j.rpsm.2018.03.001. Epub 2018 Apr 5. PMID: 29628403.	No drug
1009	Riehle M, Mehl S, Lincoln TM. The specific social costs of expressive negative symptoms in schizophrenia: reduced smiling predicts interactional outcome. Acta Psychiatr Scand. 2018;138(2):133-144. doi: 10.1111/acps.12892. Epub 2018 Apr 17. PMID: 29667181.	No drug
1010	Zhang L, Zheng H, Wu R, Zhu F, Kosten TR, Zhang XY, Zhao J. Minocycline adjunctive treatment to risperidone for negative symptoms in schizophrenia: Association with pro-inflammatory cytokine levels. Prog Neuropsychopharmacol Biol Psychiatry. 2018;85:69-76. doi: 10.1016/j.pnpbp.2018.04.004. Epub 2018 Apr 17. PMID: 29678772.	**Included**
1011	González-Blanco L, García-Portilla MP, García-Álvarez L, de la Fuente- Tomás L, Iglesias García C, Sáiz PA, Rodríguez-González S, Coto-Montes A, Bobes J. Can interleukin-2 and interleukin-1β be specific biomarkers of negative symptoms in schizophrenia? Rev Psiquiatr Salud Ment (Engl Ed). 2019;12(1):9-16. English, Spanish. doi: 10.1016/j.rpsm.2018.03.004. Epub 2018 Apr 30. PMID: 29724678.	No drug
1012	Ding N, Li Z, Liu Z. Escitalopram augmentation improves negative symptoms of treatment resistant schizophrenia patients - A randomized controlled trial. Neurosci Lett. 2018;681:68-72. doi: 10.1016/j.neulet.2018.05.030. Epub 2018 May 28. PMID: 29852208.	**Included**
1013	Keefe RSE, Harvey PD, Khan A, Saoud JB, Staner C, Davidson M, Luthringer R. Cognitive effects of MIN-101 in patients with schizophrenia and negative symptoms: Results from a randomized controlled trial. J Clin Psychiatry. 2018;79(3):17m11753. doi: 10.4088/JCP.17m11753. PMID: 29873956.	Overlap=966
1014	Ghajar A, Gholamian F, Tabatabei-Motlagh M, Afarideh M, Rezaei F, Ghazizadeh-Hashemi M, Akhondzadeh S. Citicoline (CDP-choline) add-on therapy to risperidone for treatment of negative symptoms in patients with stable schizophrenia: A double-blind, randomized placebo-controlled trial. Hum Psychopharmacol. 2018;33(4):e2662. doi: 10.1002/hup.2662. Epub 2018 Jun 14. PMID: 29901250.	**Included**
1015	Klaus F, Kaiser S, Kirschner M. Negativsymptome der Schizophrenie – ein Überblick [Negative Symptoms in Schizophrenia - an Overview]. Ther Umsch. 2018;75(1):51-56. German. doi: 10.1024/0040-5930/a000966. PMID: 29909762.	Review
1016	Ahmed AO, Kirkpatrick B, Galderisi S, Mucci A, Rossi A, Bertolino A, Rocca P, Maj M, Kaiser S, Bischof M, Hartmann-Riemer MN, Kirschner M, Schneider K, Garcia-Portilla MP, Mane A, Bernardo M, Fernandez-Egea E, Jiefeng C, Jing Y, Shuping T, Gold JM, Allen DN, Strauss GP. Cross-cultural validation of the 5-factor structure of negative symptoms in schizophrenia. Schizophr Bull. 2019;45(2):305-314. doi: 10.1093/schbul/sby050. PMID: 29912473; PMCID: PMC6403061.	No drug
1017	Stepien M, Manoliu A, Kubli R, Schneider K, Tobler PN, Seifritz E, Herdener M, Kaiser S, Kirschner M. Investigating the association of ventral and dorsal striatal dysfunction during reward anticipation with negative symptoms in patients with schizophrenia and healthy individuals. PLoS One. 2018;13(6):e0198215. doi: 10.1371/journal.pone.0198215. PMID: 29912880; PMCID: PMC6005482.	No drug
1018	Nunes MV, Adelino MPM, Ajub E, Quarantini LC, Lacerda ALT. Efficacy of esketamine in the treatment of negative symptoms in schizophrenia - A case series. Schizophr Res. 2018;202:394-396. doi: 10.1016/j.schres.2018.06.034. Epub 2018 Jun 21. PMID: 29935883.	Case
1019	Preda A, Nguyen DD, Bustillo JR, Belger A, O'Leary DS, McEwen S, Ling S, Faziola L, Mathalon DH, Ford JM, Potkin SG; FBIRN; van Erp TGM. A positive take on schizophrenia negative symptom scales: Converting scores between the SANS, NSA and SDS. Schizophr Res. 2018;201:113-119. doi: 10.1016/j.schres.2018.06.014. Epub 2018 Jun 20. PMID: 29935887; PMCID: PMC7039318.	No drug
1020	Kaneko K. Negative symptoms and cognitive impairments in schizophrenia: Two key symptoms negatively influencing social functioning. Yonago Acta Med. 2018;61(2):91-102. doi: 10.33160/yam.2018.06.001. PMID: 29946215; PMCID: PMC6015796.	No drug
1021	Tharoor H, Mara S, Gopal S. Role of novel dietary supplement N-acetyl cysteine in treating negative symptoms in schizophrenia: A 6-month follow-up study. Indian J Psychol Med. 2018;40(2):139-142. doi: 10.4103/IJPSYM.IJPSYM_322_17. PMID: 29962570; PMCID: PMC6009003.	Open
1022	Kanchanatawan B, Maes M. The effects of tryptophan catabolites on negative symptoms and deficit schizophrenia are partly mediated by executive impairments: Results of partial least squares path modeling. CNS Neurol Disord Drug Targets. 2018;17(6):473-486. doi: 10.2174/1871527317666180702160921. PMID: 29968545.	No drug
1023	Wójciak P, Rybakowski J. Clinical picture, pathogenesis and psychometric assessment of negative symptoms of schizophrenia. Psychiatr Pol. 2018;52(2):185-197. English, Polish. doi: 10.12740/PP/70610. Epub 2018 Apr 30. PMID: 29975360.	Review

1024	Bucci P, Galderisi S, Mucci A, Rossi A, Rocca P, Bertolino A, Aguglia E, Amore M, Andriola I, Bellomo A, Biondi M, Cuomo A, dell'Osso L, Favaro A, Gambi F, Giordano GM, Girardi P, Marchesi C, Monteleone P, Montemagni C, Niolu C, Oldani L, Pacitti F, Pinna F, Roncone R, Vita A, Zeppegno P, Maj M; Italian Network for Research on Psychoses. Premorbid academic and social functioning in patients with schizophrenia and its associations with negative symptoms and cognition. Acta Psychiatr Scand. 2018;138(3):253-266. doi: 10.1111/acps.12938. Epub 2018 Jul 8. PMID: 29984409.	No drug
1025	Strassnig M, Bowie C, Pinkham AE, Penn D, Twamley EW, Patterson TL, Harvey PD. Which levels of cognitive impairments and negative symptoms are related to functional deficits in schizophrenia? J Psychiatr Res. 2018;104:124-129. doi: 10.1016/j.jpsychires.2018.06.018. Epub 2018 Jul 7. PMID: 30029051.	No drug
1026	Fond G, Faugere M, Faget-Agius C, Cermolacce M, Richieri R, Boyer L, Lançon C. Hypovitaminosis D is associated with negative symptoms, suicide risk, agoraphobia, impaired functional remission, and antidepressant consumption in schizophrenia. Eur Arch Psychiatry Clin Neurosci. 2019;269(8):879-886. doi: 10.1007/s00406-018-0932-0. Epub 2018 Aug 4. PMID: 30078128.	No drug
1027	Mote J, Grant PM, Silverstein SM. Treatment implications of situational variability in cognitive and negative symptoms of schizophrenia. Psychiatr Serv. 2018;69(10):1095-1097. doi: 10.1176/appi.ps.201800073. Epub 2018 Aug 20. PMID: 30122135.	Review
1028	Kosugi N, Oshiyama C, Kodama N, Niwa SI. Incorporating music therapy into cognitive remediation to improve both cognitive dysfunction and negative symptoms in schizophrenia. Schizophr Res. 2019;204:423-424. doi: 10.1016/j.schres.2018.08.012. Epub 2018 Aug 26. PMID: 30154028.	No drug
1029	Hare SM, Ford JM, Mathalon DH, Damaraju E, Bustillo J, Belger A, Lee HJ, Mueller BA, Lim KO, Brown GG, Preda A, van Erp TGM, Potkin SG, Calhoun VD, Turner JA. Salience-default mode functional network connectivity linked to positive and negative symptoms of schizophrenia. Schizophr Bull. 2019;45(4):892-901. doi: 10.1093/schbul/sby112. Erratum in: Schizophr Bull. 2019;45(6):1382. PMID: 30169884; PMCID: PMC6581131.	No drug
1030	Earley W, Guo H, Daniel D, Nasrallah H, Durgam S, Zhong Y, Patel M, Barabássy Á, Szatmári B, Németh G. Efficacy of cariprazine on negative symptoms in patients with acute schizophrenia: A post hoc analysis of pooled data. Schizophr Res. 2019;204:282-288. doi: 10.1016/j.schres.2018.08.020. Epub 2018 Aug 29. PMID: 30172595.	**Included**
1031	Carrà G, Crocamo C, Angermeyer M, Brugha T, Toumi M, Bebbington P. Positive and negative symptoms in schizophrenia: A longitudinal analysis using latent variable structural equation modelling. Schizophr Res. 2019;204:58-64. doi: 10.1016/j.schres.2018.08.018. Epub 2018 Aug 31. PMID: 30177344.	No drugs
1032	Strauss GP, Nuñez A, Ahmed AO, Barchard KA, Granholm E, Kirkpatrick B, Gold JM, Allen DN. The latent structure of negative symptoms in schizophrenia. JAMA Psychiatry. 2018;75(12):1271-1279. doi: 10.1001/jamapsychiatry.2018.2475. PMID: 30208377; PMCID: PMC6583036.	No drug
1033	Klaus F, Dorsaz O, Kaiser S. Symptômes négatifs de la schizophrénie – état des lieux et implications pratiques [Negative symptoms in schizophrenia - overview and practical implications]. Rev Med Suisse. 2018;14(619):1660-1664. French. PMID: 30230774.	Review
1034	Muralidharan A, Harvey PD, Bowie CR. Associations of age and gender with negative symptom factors and functioning among middle-aged and older adults with schizophrenia. Am J Geriatr Psychiatry. 2018;26(12):1215-1219. doi: 10.1016/j.jagp.2018.07.006. Epub 2018 Jul 27. PMID: 30236901; PMCID: PMC6537109.	No drug
1035	Corigliano V, Comparelli A, Mancinelli I, Montalbani B, Lamis DA, De Carolis A, Erbuto D, Girardi P, Pompili M. Long-acting injectable second- generation antipsychotics improve negative symptoms and suicidal ideation in recent diagnosed schizophrenia patients: A 1-year follow-up pilot study. Schizophr Res Treatment. 2018;2018:4834135. doi: 10.1155/2018/4834135. PMID: 30245878; PMCID: PMC6136552.	No drug
1036	Kamp D, Engelke C, Wobrock T, Wölwer W, Winterer G, Schmidt-Kraepelin C, Gaebel W, Langguth B, Landgrebe M, Eichhammer P, Frank E, Hajak G, Ohmann C, Verde PE, Rietschel M, Raees A, Honer WG, Malchow B, Schneider-Axmann T, Falkai P, Hasan A, Cordes J. Left prefrontal high-frequency rTMS may improve movement disorder in schizophrenia patients with predominant negative symptoms – A secondary analysis of a sham-controlled, randomized multicenter trial. Schizophr Res. 2019;204:445-447. doi: 10.1016/j.schres.2018.09.017. Epub 2018 Sep 22. PMID: 30249471.	No drug
1037	Strauss GP, Esfahlani FZ, Galderisi S, Mucci A, Rossi A, Bucci P, Rocca P, Maj M, Kirkpatrick B, Ruiz I, Sayama H. Network analysis reveals the latent structure of negative symptoms in schizophrenia. Schizophr Bull. 2019;45(5):1033-1041. doi: 10.1093/schbul/sby133. PMID: 30256991; PMCID: PMC6737465.	No drug
1038	Pazooki K, Leibetseder M, Renner W, Gougleris G, Kapsali E. Neurofeedback treatment of negative symptoms in schizophrenia: Two case reports. Appl Psychophysiol Biofeedback. 2019;44(1):31-39. doi: 10.1007/s10484-018-9417-1. PMID: 30267339; PMCID: PMC6373527.	Case
1039	Azaiez C, Millier A, Lançon C, Clay E, Auquier P, Llorca PM, Toumi M. Health related quality of life in patients having schizophrenia negative symptoms – a systematic review. J Mark Access Health Policy. 2018;6(1):1517573. doi: 10.1080/20016689.2018.1517573. PMID: 30275939; PMCID: PMC6161588.	Review
1040	Deakin B, Suckling J, Barnes TRE, Byrne K, Chaudhry IB, Dazzan P, Drake RJ, Giordano A, Husain N, Jones PB, Joyce E, Knox E, Krynicki C, Lawrie SM, Lewis S, Lisiecka-Ford DM, Nikkheslat N, Pariante CM, Smallman R, Watson A, Williams SCR, Upthegrove R, Dunn G; BeneMin Study team. The benefit of minocycline on negative symptoms of schizophrenia in patients with recent-onset psychosis (BeneMin): a randomised, double-blind, placebo-controlled trial. Lancet Psychiatry. 2018;5(11):885-894. doi: 10.1016/S2215-0366(18)30345-6. Epub 2018 Oct 12. Erratum in: Lancet Psychiatry. 2018 Nov 1.	**Included**
1041	Yohn SE, Conn PJ. Pick your model wisely: Understanding the negative symptoms of schizophrenia in rodent models. ACS Chem Neurosci. 2019;10(1):33-35. doi: 10.1021/acschemneuro.8b00553. Epub 2018 Oct 22. PMID: 30346132; PMCID: PMC6467817.	Animal
1042	George SE, Buck KD. Metacognitive reflection and insight therapy for persons with severe negative symptoms of schizophrenia. Am J Psychother. 2018;71(4):135-144. doi: 10.1176/appi.psychotherapy.20180036. Epub 2018 Nov 7. PMID: 30400763.	No drug
1043	Kim SW, Stewart R, Park WY, Jhon M, Lee JY, Kim SY, Kim JM, Amminger P, Chung YC, Yoon JS. Latent iron deficiency as a marker of negative symptoms in patients with first-episode schizophrenia spectrum disorder. Nutrients. 2018;10(11):1707. doi: 10.3390/nu10111707. PMID: 30412998; PMCID: PMC6266210.	No drug
1044	Bergé D, Pretus C, Guell X, Pous A, Arcos A, Pérez V, Vilarroya O. Reduced willingness to invest effort in schizophrenia with high negative symptoms regardless of reward stimulus presentation and reward value. Compr Psychiatry. 2018;87:153-160. doi: 10.1016/j.comppsych.2018.10.010. Epub 2018 Nov 6. PMID: 30415197.	No drug
1045	Iasevoli F, Avagliano C, Altavilla B, Barone A, D'Ambrosio L, Matrone M, Notar Francesco D, Razzino E, de Bartolomeis A. Disease severity in treatment resistant schizophrenia patients is mainly affected by negative symptoms, which mediate the effects of cognitive dysfunctions and neurological soft signs. Front Psychiatry. 2018 31;9:553. doi: 10.3389/fpsyt.2018.00553. PMID: 30429802; PMCID: PMC6220073.	No drug
1046	Cho JM, Lee K. Effects of motivation interviewing using a group art therapy program on negative symptoms of schizophrenia. Arch Psychiatr Nurs. 2018;32(6):878-884. doi: 10.1016/j.apnu.2018.07.002. Epub 2018 Jul 19. PMID: 30454632.	No drug
1047	Davarinejad O, Hendesi K, Shahi H, Brand S, Khazaie H. A pilot study on daily intensive ECT over 8 days improved positive and negative symptoms and general psychopathology of patients with treatment-resistant schizophrenia up to 4 weeks after treatment. Neuropsychobiology. 2019;77(2):83-91. doi: 10.1159/000494698. PMID: 30463074.	No drug
1048	Maes M, Kanchanatawan B, Sirivichayakul S, Carvalho AF. In schizophrenia, deficits in natural IgM isotype antibodies including those directed to malondialdehyde and azelaic acid strongly predict negative symptoms, neurocognitive impairments, and the deficit syndrome. Mol Neurobiol. 2019;56(7):5122-5135. doi: 10.1007/s12035-018-1437-6. PMID: 30484113.	No drug

1049	Strauss GP, Ahmed AO, Young JW, Kirkpatrick B. Reconsidering the latent structure of negative symptoms in schizophrenia: a review of evidence supporting the 5 consensus domains. Schizophr Bull. 2019;45(4):725-729. doi: 10.1093/schbul/sby169. PMID: 30541136; PMCID: PMC6581128.	Review
1050	Maes M, Kanchanatawan B, Sirivichayakul S, Carvalho AF. In schizophrenia, increased plasma IgM/IgA responses to gut commensal bacteria are associated with negative symptoms, neurocognitive impairments, and the deficit phenotype. Neurotox Res. 2019;35(3):684-698. doi: 10.1007/s12640-018-9987-y. PMID: 30552634.	No drug
1051	Rekhi G, Ang MS, Yuen CKY, Ng WY, Lee J. Assessing negative symptoms in schizophrenia: validity of the clinical assessment interview for negative symptoms in Singapore. Schizophr Res. 2019;206:177-182. doi: 10.1016/j.schres.2018.11.029. PMID: 30558979.	No drug
1052	Shukla DK, Chiappelli JJ, Sampath H, Kochunov P, Hare SM, Wisner K, Rowland LM, Hong LE. Aberrant frontostriatal connectivity in negative symptoms of schizophrenia. Schizophr Bull. 2019;45(5):1051-1059. doi: 10.1093/schbul/sby165. PMID: 30576563; PMCID: PMC6737477	No drug
1053	Luther L, Fischer MW, Firmin RL, Salyers MP. Clarifying the overlap between motivation and negative symptom measures in schizophrenia research: A meta-analysis. Schizophr Res. 2019;206:27-36. doi: 10.1016/j.schres.2018.10.010. PMID: 30577993; PMCID: PMC6525651.	Review
1054	Bellavia A, Centorrino F, Jackson JW, Fitzmaurice G, Valeri L. The role of weight gain in explaining the effects of antipsychotic drugs on positive and negative symptoms: An analysis of the CATIE schizophrenia trial. Schizophr Res. 2019;206:96-102. doi: 10.1016/j.schres.2018.12.006. PMID: 30584025.	Unfocused
1055	Zhang Z, Zhang R, Qin P, Tan L. Cognitive dysfunction and negative symptoms in patients with schizophrenia and their first-degree relatives from simplex and multiplex families. Neuropsychiatr Dis Treat. 2018;14:3339-3348. doi: 10.2147/NDT.S179534. PMID: 30584307; PMCID: PMC6287416.	No drug
1056	İnanç L, Sevinç E, Semiz ÜB. Relationship between alexithymia, depression and the negative symptoms in schizophrenia with and without deficit syndrome. Türk Psikiyatri Derg. 2019;30(4):225-235. PMID: 32594483.	No drug
1057	Gao J, Tang X, Wang C, Yu M, Sha W, Wang X, Zhang H, Zhang X, Zhang X. Aberrant cerebellar neural activity and cerebro-cerebellar functional connectivity involving executive dysfunction in schizophrenia with primary negative symptoms. Brain Imaging Behav. 2020;14(3):869-880. doi: 10.1007/s11682-018-0032-9. PMID: 30612342.	No drug
1058	Wu YF, Sytwu HK, Lung FW. Human aquaporin 4 gene polymorphisms and haplotypes are associated with serum S100B level and negative symptoms of schizophrenia in a Southern Chinese Han Population. Front Psychiatry. 2018;9:657. doi: 10.3389/fpsyt.2018.00657. PMID: 30618856; PMCID: PMC6297372.	No drug
1059	Koola MM. Potential role of antipsychotic-galantamine-memantine combination in the treatment of positive, cognitive, and negative symptoms of schizophrenia. Mol Neuropsychiatry. 2018;4(3):134-148. doi: 10.1159/000494495. PMID: 30643787; PMCID: PMC6323397.	Review
1060	Strauss GP, Esfahlani FZ, Kirkpatrick B, Allen DN, Gold JM, Visser KF, Sayama H. Network Analysis reveals which negative symptom domains are most central in schizophrenia vs bipolar disorder. Schizophr Bull. 2019;45(6):1319-1330. doi: 10.1093/schbul/sby168. PMID: 30649527; PMCID: PMC6811832.	No drug
1061	Brady RO Jr, Gonsalvez I, Lee I, Öngür D, Seidman LJ, Schmahmann JD, Eack SM, Keshavan MS, Pascual-Leone A, Halko MA. Cerebellar-prefrontal network connectivity and negative symptoms in schizophrenia. Am J Psychiatry. 2019;176(7):512-520. doi: 10.1176/appi.ajp.2018.18040429. PMID: 30696271; PMCID: PMC6760327.	No drug
1062	Wagner E, Wobrock T, Kunze B, Langguth B, Landgrebe M, Eichhammer P, Frank E, Cordes J, Wölwer W, Winterer G, Gaebel W, Hajak G, Ohmann C, Verde PE, Rietschel M, Ahmed R, Honer WG, Siskind D, Malchow B, Strube W, Schneider-Axmann T, Falkai P, Hasan A. Efficacy of high-frequency repetitive transcranial magnetic stimulation in schizophrenia patients with treatment-resistant negative symptoms treated with clozapine. Schizophr Res. 2019;208:370-376. doi: 10.1016/j.schres.2019.01.021. PMID: 30704862.	No drug
1063	Fleischhacker W, Galderisi S, Laszlovszky I, Szatmári B, Barabássy Á, Acsai K, Szalai E, Harsányi J, Earley W, Patel M, Németh G. The efficacy of cariprazine in negative symptoms of schizophrenia: Post hoc analyses of PANSS individual items and PANSS-derived factors. Eur Psychiatry. 2019;58:1-9. doi: 10.1016/j.eurpsy.2019.01.015. PMID: 30738380.	*Post hoc*
1064	Koola MM. Antipsychotic-minocycline-acetylcysteine combination for positive, cognitive, and negative symptoms of schizophrenia. Asian J Psychiatr. 2019;40:100-102. doi: 10.1016/j.ajp.2019.02.007. PMID: 30776665.	Opinion
1065	Leucht S, Barabássy Á, Laszlovszky I, Szatmári B, Acsai K, Szalai E, Harsányi J, Earley W, Németh G. Linking PANSS negative symptom scores with the Clinical Global Impressions Scale: understanding negative symptom scores in schizophrenia. Neuropsychopharmacology. 2019;44(9):1589-1596. doi: 10.1038/s41386-019-0363-2. PMID: 30836381; PMCID: PMC6785000.	Overlap=940
1066	Carrà G, Crocamo C, Bartoli F, Angermeyer M, Brugha T, Toumi M, Bebbington P. The mediating role of depression in pathways linking positive and negative symptoms in schizophrenia. A longitudinal analysis using latent variable structural equation modelling. Psychol Med. 2020;50(4):566-574. doi: 10.1017/S0033291719000321. PMID: 30846005.	No drug
1067	Wehbé J, Haddad C, Obeid S, Hallit S, Haddad G. Prevalence of obsessive-compulsive disorder in patients with schizophrenia and outcome on positive and negative symptoms, cognition, and quality of life. J Nerv Ment Dis. 2019;207(4):239-245. doi: 10.1097/NMD.0000000000000956 PMID: 30865076.	No drug
1068	Vogel JS, van der Gaag M, Slofstra C, Knegtering H, Bruins J, Castelein S. The effect of mind-body and aerobic exercise on negative symptoms in schizophrenia: A meta-analysis. Psychiatry Res. 2019;279:295-305. doi: 10.1016/j.psychres.2019.03.012. Epub 2019 Mar 15. PMID: 30879703.	Review
1069	Pedersen IN, Bonde LO, Hannibal NJ, Nielsen J, Aagaard J, Bertelsen LR, Jensen SB, Nielsen RE. Music therapy as treatment of negative symptoms for adult patients diagnosed with schizophrenia-Study protocol for a randomized, controlled and blinded study. Medicines (Basel). 2019;6(2):46. doi: 10.3390/medicines6020046. PMID: 30939778; PMCID: PMC6631001.	No drug
1070	Tahir Y, Yang Z, Chakraborty D, Thalmann N, Thalmann D, Maniam Y, Binte Abdul Rashid NA, Tan BL, Lee Chee Keong J, Dauwels J. Non-verbal speech cues as objective measures for negative symptoms in patients with schizophrenia. PLoS One. 2019;14(4):e0214314. doi: 10.1371/journal.pone.0214314. PMID: 30964869; PMCID: PMC6456189.	No drug
1071	Nemoto T, Uchino T, Aikawa S, Saito J, Matsumoto H, Funatogawa T, Yamaguchi T, Katagiri N, Tsujino N, Mizuno M. Social anxiety and negative symptoms as the characteristics of patients with schizophrenia who show competence-performance discrepancy in social functioning. Psychiatry Clin Neurosci. 2019;73(7):394-399. doi: 10.1111/pcn.12848. PMID: 30968478.	No drug
1072	Soontornniyomkij V, Lee EE, Jin H, Martin AS, Daly RE, Liu J, Tu XM, Eyler LT, Jeste DV. Clinical correlates of insulin resistance in chronic schizophrenia: relationship to negative symptoms. Front Psychiatry. 2019;10:251. doi: 10.3389/fpsyt.2019.00251. PMID: 31065243; PMCID: PMC6488983.	No drug
1073	Li J, Yang S, Liu X, Han Y, Li Y, Feng J, Zhao H. Hypoactivity of the lateral habenula contributes to negative symptoms and cognitive dysfunction of schizophrenia in rats. Exp Neurol. 2019;318:165-173. doi: 10.1016/j.expneurol.2019.05.005. PMID: 31082390.	Animal
1074	Lavallé L, Aleman A. rTMS for treatment of negative symptoms in schizophrenia: clinical effects and neural basis. Encéphale. 2019;45(Suppl 2):S50-S51. doi: 10.1016/j.encep.2019.04.002. PMID: 31101378.	Opinion
1075	Batinic B. Cognitive models of positive and negative symptoms of schizophrenia and implications for treatment. Psychiatr Danub. 2019;31(Suppl 2):181-184. PMID: 31158119.	No drug
1076	Zhuo K, Tang Y, Song Z, Wang Y, Wang J, Qian Z, Li H, Xiang Q, Chen T, Yang Z, Xu Y, Fan X, Wang J, Liu D. Repetitive transcranial magnetic stimulation as an adjunctive treatment for negative symptoms and cognitive impairment in patients with schizophrenia: a randomized, double-blind, sham-controlled trial. Neuropsychiatr Dis Treat. 2019;15:1141-1150. doi: 10.2147/NDT.S196086. PMID: 31190822; PMCID: PMC6514121.	No drug

1077	Naguy A, AlShalabi SR, AlKhadhari S. Betahistine-associated weight loss and improved cognitive and negative symptoms: domain in early-onset schizophrenia. Am J Ther. 2019;26(6):e790-e792. doi: 10.1097/MJT.0000000000000965. PMID: 31206362.	Case
1078	Cerveri G, Gesi C, Mencacci C. Pharmacological treatment of negative symptoms in schizophrenia: update and proposal of a clinical algorithm Neuropsychiatr Dis Treat. 2019;15:1525-1535. doi: 10.2147/NDT.S201726. PMID: 31239687; PMCID: PMC6556563.	Review
1079	Curtin A, Sun J, Zhao Q, Onaral B, Wang J, Tong S, Ayaz H. Visuospatial task-related prefrontal activity is correlated with negative symptoms in schizophrenia. Sci Rep. 2019;9(1):9575. doi: 10.1038/s41598-019-45893-7. PMID: 31270354; PMCID: PMC6610077.	No drug
1080	Yang Y, Li R, Hei G, Wu R. [Role of sulforaphane in improving negative symptoms and cognitive symptoms of schizophrenia and the underlying mechanism]. Zhong Nan Da Xue Xue Bao Yi Xue Ban. 2019;44(6):701-705. Chinese. doi: 10.11817/j.issn.1672-7347.2019.06.014. PMID: 31304933.	Review
1081	Smulevich AB, Kharkova GS, Lobanova VM, Voronova EI. Asteniia v psikhopatologicheskom prostranstve shizofrenii i rasstroĭstv shizofrenicheskogo spektra (kontseptsiia astenicheskogo defekta v aspekte sovremennykh modeleĭ negativnykh rasstroĭstv) [Asthenia in the psychopathological space of schizophrenia and schizophrenia spectrum disorders (The concept of asthenic deficit in aspect of the modern model of negative symptoms)]. Zh Nevrol Psikhiatr Im S S Korsakova. 2019;119(5):7-14. Russian. doi: 10.17116/jnevro20191190517. PMID: 31317883.	No drug
1082	Dollfus S, Delouche C, Hervochon C, Mach C, Bourgeois V, Rotharmel M, Tréhout M, Vandevelde A, Guillin O, Morello R. Specificity and sensitivity of the Self-assessment of Negative Symptoms (SNS) in patients with schizophrenia. Schizophr Res. 2019;211:51-55. doi: 10.1016/j.schres.2019.07.012. PMID: 31345705.	No drug
1083	Hereta M, Kamińska K, Rogóż Z. Co-treatment with antidepressants and aripiprazole reversed the MK-801-induced some negative symptoms of schizophrenia in rats. Pharmacol Rep. 2019;71(5):768-773. doi: 10.1016/j.pharep.2019.04.007. Epub 2019 Apr 9. PMID: 31351318.	Animal
1084	Garg H, Kumar S, Singh S, Kumar N, Verma R. New onset obsessive compulsive disorder following high frequency repetitive transcranial magnetic stimulation over left dorsolateral prefrontal cortex for treatment of negative symptoms in a patient with schizophrenia. Clin Psychopharmacol Neurosci. 2019;17(3):443-445. doi: 10.9758/cpn.2019.17.3.443. PMID: 31352712; PMCID: PMC6705105.	Case
1085	Chang CC, Kao YC, Chao CY, Tzeng NS, Chang HA. Examining bi-anodal transcranial direct current stimulation (tDCS) over bilateral dorsolateral prefrontal cortex coupled with bilateral extracephalic references as a treatment for negative symptoms in non-acute schizophrenia patients: A randomized, double- blind, sham-controlled trial. Prog Neuropsychopharmacol Biol Psychiatry. 2020;96:109715. doi: 10.1016/j.pnpbp.2019.109715. PMID: 31362034.	No drug
1086	Deakin B, Suckling J, Dazzan P, Joyce E, Lawrie SM, Upthegrove R, Husain N, Chaudhry IB, Dunn G, Jones PB, Lisiecka-Ford D, Lewis S, Barnes TRE, Williams SCR, Pariante CM, Knox E, Drake RJ, Smallman R, Barnes NM. Minocycline for negative symptoms of schizophrenia and possible mechanistic actions: the BeneMin RCT. Southampton (UK): NIHR Journals Library; 2019 Aug.	Overlap= 1040
1087	Rabinowitz J, Badescu S, Palamarchuk P, Filyk V, Voloshchuk A, Rud V, Melnyk E, Skrypnikov A, Davidson M, Saoud J, Luthringer R. Personal and social adjustment effects of roluperidone in patients with schizophrenia and negative symptoms: Results from an exploratory outcome of a randomized placebo-controlled trial. Schizophr Res. 2019;211:103-104. doi: 10.1016/j.schres.2019.07.029. PMID: 31375316.	Overlap= 1269,1358
1088	Sabe M, Sentissi O, Kaiser S. Meditation-based mind-body therapies for negative symptoms of schizophrenia: Systematic review of randomized controlled trials and meta-analysis. Schizophr Res. 2019;212:15-25. doi: 10.1016/j.schres.2019.07.030. PMID: 31378557.	Review
1089	Favrod J, Nguyen A, Tronche AM, Blanc O, Dubreucq J, Chereau-Boudet I, Capdevielle D, Llorca PM. Impact of positive emotion regulation training on negative symptoms and social functioning in schizophrenia: a field test. Front Psychiatry. 2019;10:532. doi: 10.3389/fpsyt.2019.00532. PMID: 31404331; PMCID: PMC6677145.	No drug
1090	Corrigendum: Treatments of negative symptoms in schizophrenia: meta-analysis of randomized placebo-controlled trials. Schizophr Bull. 2022;48(3):721. doi: 10.1093/schbul/sbz071. Erratum for: Fusar-Poli et al 2015 Schizophr Bull 41 892-9. PMID: 31504973; PMCID: PMC9077424.	Review
1091	Szulc A, Samochowiec J, Gałecki P, Wojnar M, Heitzman J, Dudek D. Recommendations for the treatment of schizophrenia with negative symptoms. Standards of pharmacotherapy by the Polish Psychiatric Association (Polskie Towarzystwo Psychiatryczne), part 1. Psychiatr Pol. 2019;53(3):497-524. doi: 10.12740/PP/OnlineFirst/100698. PMID: 31522193.	Review
1092	Szulc A, Dudek D, Samochowiec J, Wojnar M, Heitzman J, Gałecki P. Recommendations for the treatment of schizophrenia with negative symptoms. Standards of pharmacotherapy by the Polish Psychiatric Association (Polskie Towarzystwo Psychiatryczne), part 2. Psychiatr Pol. 2019;53(3):525-540. English, Polish. doi: 10.12740/PP/OnlineFirst/100697. PMID: 31522194.	Review
1093	Laszlovszky I, Kiss B, Barabassy A, Kapas M, Nemeth G. Kariprazin, egy új típusú – dopamin D_3 receptort preferáló – parciális agonista atípusos antipszichotikum a szkizofrénia és primer negatív tüneteinek kezelésére [Cariprazine, a new type - dopamine D_3 receptor preferring - partial agonist atypical antipsychotic for the treatment of schizophrenia and the primary negative symptoms]. Neuropsychopharmacol Hung. 2019;21(3):103-118. Hungarian. PMID: 31537751.	Review
1094	Chen H, Xu J, Mao Y, Sun L, Sun Y, Zhou Y. Positive coping and resilience as mediators between negative symptoms and disability among patients with schizophrenia. Front Psychiatry. 2019;10:641. doi: 10.3389/fpsyt.2019.00641. PMID: 31551832; PMCID: PMC6748026.	No drug
1095	Li X, Chu MY, Lv QY, Hu HX, Li Z, Yi ZH, Wang JH, Zhang JY, Lui SSY, Cheung EFC, Shum DHK, Chan RCK. The remediation effects of working memory training in schizophrenia patients with prominent negative symptoms. Cogn Neuropsychiatry. 2019;24(6):434-453. doi: 10.1080/13546805.2019.1674644. Epub 2019 Oct 4. PMID: 31583951.	No drug
1096	Vanes LD, Mouchlianitis E, Patel K, Barry E, Wong K, Thomas M, Szentgyorgyi T, Joyce D, Shergill S. Neural correlates of positive and negative symptoms through the illness course: an fMRI study in early psychosis and chronic schizophrenia. Sci Rep. 2019;9(1):14444. doi: 10.1038/s41598-019-51023-0. PMID: 31595009; PMCID: PMC6783468.	No drug
1097	Abram SV, De Coster L, Roach BJ, Mueller BA, van Erp TGM, Calhoun VD, Preda A, Lim KO, Turner JA, Ford JM, Mathalon DH, Woolley JD. Oxytocin enhances an amygdala circuit associated with negative symptoms in schizophrenia: A single-dose, placebo-controlled, crossover, randomized control trial. Schizophr Bull. 2020;46(3):661-669. doi: 10.1093/schbul/sbz091. PMID: 31595302; PMCID: PMC7147578.	Unfocused
1098	Valiengo LDCL, Goerigk S, Gordon PC, Padberg F, Serpa MH, Koebe S, Santos LAD, Lovera RAM, Carvalho JB, van de Bilt M, Lacerda ALT, Elkis H, Gattaz WF, Brunoni AR. Efficacy and safety of transcranial direct current stimulation for treating negative symptoms in schizophrenia: A randomized clinical trial. JAMA Psychiatry. 2020;77(2):121-129. doi: 10.1001/jamapsychiatry.2019.3199. PMID: 31617873; PMCID: PMC6802484.	No drug
1099	Ivanov SV, Kharkova GS, Smulevich AB, Morozova YV. Primenenie kontsentrata iadrosoderzhashchikh kletok pupovinnoĭ krovi u bol'nykh s astenicheskimi rasstroĭstvami (shizoasteniia) v ramkakh shizofrenii, protekaiushcheĭ s preobladaniem negativnykh rasstroĭstv [The use of the concentrate of nucleus containing cells of umbilical cord blood for treatment of asthenic disorders (schizoasthenia) in patients with schizophrenia with negative symptoms]. Zh Nevrol Psikhiatr Im S S Korsakova. 2019;119(9):68-73. Russian. doi: 10.17116/jnevro201911909168. PMID: 31626221.	No drug
1100	James S, Kapugama C, Al-Uzri M. Corrigendum to "Use of Aripiprazole Long Acting Injection in Negative Symptoms of Schizophrenia". Case Rep Psychiatry. 2019;2019:9595638. doi: 10.1155/2019/9595638. Erratum for: Case Rep Psychiatry. 2016;2016:7912083. PMID: 31637079; PMCID: PMC6766089.	Duplicate= 878
1101	Sisek-Šprem M, Gradiški IP, Žaja N, Herceg M. The longitudinal course of schizophrenia: testosterone and progression of the negative symptoms. Nord J Psychiatry. 2020;74(2):147-154. doi: 10.1080/08039488.2019.1681509. Epub 2019 Oct 25. PMID: 31651218.	No drug
1102	Ditlevsen JV, Simonsen A, Bliksted VF. Predicting mentalizing deficits in first-episode schizophrenia from different subdomains of negative symptoms. Schizophr Res. 2020;215:439-441. doi: 10.1016/j.schres.2019.10.036. Epub 2019 Oct 28. PMID: 31672383.	No drug

1103	Sabe M, Kirschner M, Kaiser S. Prodopaminergic drugs for treating the negative symptoms of schizophrenia: Systematic review and meta-analysis of randomized controlled trials. J Clin Psychopharmacol. 2019;39(6):658-664. doi: 10.1097/JCP.0000000000001124. PMID: 31688399.	Review
1104	Depp CA, Bashem J, Moore RC, Holden JL, Mikhael T, Swendsen J, Harvey PD, Granholm EL. GPS mobility as a digital biomarker of negative symptoms in schizophrenia: a case control study. NPJ Digit Med. 2019;2:108. doi: 10.1038/s41746-019-0182-1. PMID: 31728415; PMCID: PMC6841669.	No drug
1105	Sabe M, Kaiser S, Sentissi O. Physical exercise for negative symptoms of schizophrenia: Systematic review of randomized controlled trials and meta- analysis. Gen Hosp Psychiatry. 2020;62:13-20. doi: 10.1016/j.genhosppsych.2019.11.002. Epub 2019 Nov 12. PMID: 31751931.	Review
1106	Rekhi G, Alphs L, Ang MS, Lee J. Clinical utility of the Negative Symptom Assessment-16 in individuals with schizophrenia. Eur Neuropsychopharmacol. 2019;29(12):1433-1441. doi: 10.1016/j.euroneuro.2019.10.009. Epub 2019 Nov 22. PMID: 31761524.	No drug
1107	Pelizza L, Landi G, Pellegrini C, Quattrone E, Azzali S, Pellegrini P, Leuci E. Negative symptom configuration in first episode Schizophrenia: findings from the "Parma Early Psychosis" program. Nord J Psychiatry. 2020;74(4):251-258. doi: 10.1080/08039488.2019.1695286. Epub 2019 Nov 25. PMID: 31762390.	No drug
1108	Linsambarth S, Jeria A, Avirame K, Todder D, Riquelme R, Stehberg J. Deep transcranial magnetic stimulation for the treatment of negative symptoms in schizophrenia: Beyond an antidepressant effect. J ECT. 2019;35(4):e46-e54. doi: 10.1097/YCT.0000000000000592. PMID: 31764455.	No drug
1109	Green MF. From social cognition to negative symptoms in schizophrenia: How do we get there from here? Schizophr Bull. 2020;46(2):225-226. doi: 10.1093/schbul/sbz113. PMID: 31776580; PMCID: PMC7043055.	No drug
1110	Vita A, Perin AP, Cavanna M, Cobelli F, Rosa J, Valsecchi P, Zanigni M, Reggiardo G, Sacchetti E. Negative symptom severity at discharge from an index hospitalization and subsequent use of psychiatric care resources: A retrospective 1-year follow-up study on 450 patients with schizophrenia spectrum disorders. Schizophr Res. 2020;216:243-248. doi: 10.1016/j.schres.2019.11.052. Epub 2019 Dec 7. PMID: 31818634.	No drug
1111	Sevy S, Lindenmayer JP, Khan A, Ljuri I, Kulsa MKC, Jones O. Differential improvement of negative-symptom subfactors after cognitive remediation in low-functioning individuals with schizophrenia. Schizophr Res Cogn. 2019;19:100145. doi: 10.1016/j.scog.2019.100145. PMID: 31828020; PMCID: PMC6889361.	No drug
1112	Lui SSY, Chiu MWY, Chui WWH, Wong JOY, Man CMY, Cheung EFC, Chan RCK. Impaired olfactory identification and hedonic judgment in schizophrenia patients with prominent negative symptoms. Cogn Neuropsychiatry. 2020;25(2):126-138. doi: 10.1080/13546805.2019.1704709. Epub 2019 Dec 19. PMID: 31856651.	No drug
1113	Liu J, Chua JJ, Chong SA, Subramaniam M, Mahendran R. The impact of emotion dysregulation on positive and negative symptoms in schizophrenia spectrum disorders: A systematic review. J Clin Psychol. 2020;76(4):612-624. doi: 10.1002/jclp.22915. Epub 2020 Jan 7. PMID: 31909833.	Review
1114	Guessoum SB, Le Strat Y, Dubertret C, Mallet J. A transnosographic approach of negative symptoms pathophysiology in schizophrenia and depressive disorders. Prog Neuropsychopharmacol Biol Psychiatry. 2020;99:109862. doi: 10.1016/j.pnpbp.2020.109862. Epub 2020 Jan 10. PMID: 31927053.	No drug
1115	Jeakal E, Park K, Lee E, Strauss GP, Choi KH. Validation of the Brief Negative Symptom Scale in Korean patients with schizophrenia. Asia Pac Psychiatry. 2020;12(3):e12382. doi: 10.1111/appy.12382. Epub 2020 Jan 20. PMID: 31960582.	No drug
1116	Singh S, Kumar N, Verma R, Nehra A. The safety and efficacy of adjunctive 20-Hz repetitive transcranial magnetic stimulation for treatment of negative symptoms in patients with schizophrenia: A double-blinded, randomized, sham- controlled study. Indian J Psychiatry. 2020;62(1):21-29. doi: 10.4103/psychiatry.IndianJPsychiatry_361_19. Epub 2020 Jan 3. PMID: 32001927; PMCID: PMC6964461.	No drug
1117	Andrade C. Transcranial direct current stimulation for negative symptoms of schizophrenia: Why the reader must choose a clinically relevant outcome. J Clin Psychiatry. 2020;81(1):20f13256. doi: 10.4088/JCP.20f13256. PMID: 32023365.	Opinion
1118	Correll CU, Schooler NR. Negative symptoms in schizophrenia: A review and clinical guide for recognition, assessment, and treatment. Neuropsychiatr Dis Treat. 2020;16:519-534. doi: 10.2147/NDT.S225643. PMID: 32110026; PMCID: PMC7041437.	Review
1119	López-Díaz Á, Menéndez-Sampil C, Pérez-Romero A, Palermo-Zeballos FJ, Valdés-Florido MJ. Characterization of deficit schizophrenia and reliability of the bidimensional model of its negative symptomatology. Nord J Psychiatry. 2020;74(6):400-406. doi: 10.1080/08039488.2020.1736151. Epub 2020 Mar 9. PMID: 32149549.	No drug
1120	Régio Brambilla C, Veselinović T, Rajkumar R, Mauler J, Orth L, Ruch A, Ramkiran S, Heekeren K, Kawohl W, Wyss C, Kops ER, Scheins J, Tellmann L, Boers F, Neumaier B, Ermert J, Herzog H, Langen KJ, Jon Shah N, Lerche C, Neuner I. mGluR5 receptor availability is associated with lower levels of negative symptoms and better cognition in male patients with chronic schizophrenia. Hum Brain Mapp. 2020;41(10):2762-2781. doi: 10.1002/hbm.24976. Epub 2020 Mar 9. PMID: 32150317; PMCID: PMC7294054.	No drug
1121	Goldsmith DR, Rapaport MH. Inflammation and negative symptoms of schizophrenia: Implications for reward processing and motivational deficits. Front Psychiatry. 2020;11:46. doi: 10.3389/fpsyt.2020.00046. PMID: 32153436; PMCID: PMC7044128.	No drug
1122	Gopal S, Gogate J, Pungor K, Kim E, Singh A, Mathews M. Improvement of negative symptoms in schizophrenia with paliperidone palmitate 1-month and 3-month long-acting injectables: Results from a phase 3 non-inferiority study. Neuropsychiatr Dis Treat. 2020;16:681-690. doi: 10.2147/NDT.S226296. PMID: 32184607; PMCID: PMC7064291.	No placebo comparator
1123	Xiu MH, Guan HY, Zhao JM, Wang KQ, Pan YF, Su XR, Wang YH, Guo JM, Jiang L, Liu HY, Sun SG, Wu HR, Geng HS, Liu XW, Yu HJ, Wei BC, Li XP, Trinh T, Tan SP, Zhang XY. Cognitive enhancing effect of high-frequency neuronavigated rTMS in chronic schizophrenia patients with predominant negative symptoms: A double- blind controlled 32-week follow-up study. Schizophr Bull. 2020;46(5):1219-1230. doi: 10.1093/schbul/sbaa035. PMID: 32185388; PMCID: PMC7505170.	No drug
1124	Kayo M, Scemes S, Savoia MG, Bichuette A, Abreu AC, da Silva EP, Baria P, Piovaccari V, Petreche B, Bressan RA, Gadelha A, Elkis H. A randomized controlled trial of social skills training for patients with treatment-resistant schizophrenia with predominantly negative symptoms. Psychiatry Res. 2020;287:112914. doi: 10.1016/j.psychres.2020.112914. Epub 2020 Mar 18. PMID: 32199184.	No drug
1125	Butcher I, Berry K, Haddock G. Understanding individuals' subjective experiences of negative symptoms of schizophrenia: A qualitative study. Br J Clin Psychol. 2020;59(3):319-334. doi: 10.1111/bjc.12248. Epub 2020 Apr 3. PMID: 32242945.	No drug
1126	Chan SKW, Chan HYV, Pang HH, Hui CLM, Suen YN, Chang WC, Lee EHM, Chen EYH. Ten-year trajectory and outcomes of negative symptoms of patients with first-episode schizophrenia spectrum disorders. Schizophr Res. 2020;220:85-91. doi: 10.1016/j.schres.2020.03.061. Epub 2020 Apr 8. PMID: 32278539.	No drug
1127	Kumar N, Vishnubhatla S, Wadhawan AN, Minhas S, Gupta P. A randomized, double blind, sham-controlled trial of repetitive transcranial magnetic stimulation (rTMS) in the treatment of negative symptoms in schizophrenia. Brain Stimul. 2020;13(3):840-849. doi: 10.1016/j.brs.2020.02.016. Epub 2020 Feb 29. PMID: 32289715.	No drug
1128	Ishiwata S, Hattori K, Hidese S, Sasayama D, Miyakawa T, Matsumura R, Yokota Y, Yoshida S, Kunugi H. Lower cerebrospinal fluid CRH concentration in chronic schizophrenia with negative symptoms. J Psychiatr Res. 2020;127:13-19. doi: 10.1016/j.jpsychires.2020.03.010. Epub 2020 Apr 16. PMID: 32339962.	No drug
1129	Liu Z, Zhang Y, Zhao T, Wang J, Xia L, Zhong Y, Yang Y, Ning X, Zhang Y, Ren Z, Liu H. A higher body mass index in Chinese inpatients with chronic schizophrenia is associated with elevated plasma orexin-A levels and fewer negative symptoms. Nord J Psychiatry. 2020;74(7):525-532. doi: 10.1080/08039488.2020.1755995. Epub 2020 May 4. PMID: 32363986.	No drug
1130	Molnar MJ, Jimoh IJ, Zeke H, Palásti Á, Fedor M. Early-onset schizophrenia with predominantly negative symptoms: A case study of a drug-naive female patient treated with cariprazine. Front Pharmacol. 2020;11:477. doi: 10.3389/fphar.2020.00477. PMID: 32390838; PMCID: PMC7191004.	Case

1131	Bryl K, Bradt J, Cechnicki A, Fisher K, Sossin KM, Goodill S. The role of dance/movement therapy in the treatment of negative symptoms in schizophrenia: a mixed methods pilot study. J Ment Health. 2022;31(5):613-623. doi: 10.1080/09638237.2020.1757051. Epub 2020 May 13. PMID: 32401072.	No drug
1132	Hidalgo S, Castro C, Zárate RV, Valderrama BP, Hodge JJL, Campusano JM. The behavioral and neurochemical characterization of a Drosophila dysbindin mutant supports the contribution of serotonin to schizophrenia negative symptoms. Neurochem Int. 2020;138:104753. doi: 10.1016/j.neuint.2020.104753. Epub 2020 May 13. PMID: 32416114.	Animal
1133	Li X, Yuan X, Kang Y, Pang L, Liu Y, Zhu Q, Lv L, Huang XF, Song X. A synergistic effect between family intervention and rTMS improves cognitive and negative symptoms in schizophrenia: A randomized controlled trial. J Psychiatr Res. 2020;126:81-91. doi: 10.1016/j.jpsychires.2020.04.009. Epub 2020 Apr 28. PMID: 32428747.	No drug
1134	Almulla AF, Al-Hakeim HK, Maes M. Schizophrenia phenomenology revisited: positive and negative symptoms are strongly related reflective manifestations of an underlying single trait indicating overall severity of schizophrenia. CNS Spectr. 2021;26(4):368-377. doi: 10.1017/S1092852920001182. Epub 2020 May 20. PMID: 32431263.	No drug
1135	Baandrup L, Allerup P, Nielsen MØ, Bak N, Düring SW, Leucht S, Galderisi S, Mucci A, Bucci P, Arango C, Díaz-Caneja CM, Dazzan P, McGuire P, Demjaha A, Ebdrup BH, Kahn RS, Glenthøj BY. Rasch analysis of the PANSS negative subscale and exploration of negative symptom trajectories in first-episode schizophrenia – data from the OPTiMiSE trial. Psychiatry Res. 2020;289:112970. doi: 10.1016/j.psychres.2020.112970. Epub 2020 Apr 18. PMID: 32438207.	Unfocused
1136	Huang WC, Liu WS, Chen TT, Chen WH, Huang WL. Parasympathetic activity as a potential biomarker of negative symptoms in patients with schizophrenia. Asia Pac Psychiatry. 2020;12(3):e12392. doi: 10.1111/appy.12392. Epub 2020 May 26. PMID: 32452616.	No drug
1137	Thonon B, Levaux MN, Della Libera C, Larøi F. Switch, a new intervention that targets motivational negative symptoms in people with schizophrenia: An uncontrolled pilot study. J Clin Psychol. 2020;76(10):1797-1806. doi: 10.1002/jclp.22963. Epub 2020 May 26. PMID: 32453892.	No drug
1138	Wang X, Lu F, Duan X, Han S, Guo X, Yang M, Zhang Y, Xiao J, Sheng W, Zhao J, Chen H. Frontal white matter abnormalities reveal the pathological basis underlying negative symptoms in antipsychotic-naïve, first-episode patients with adolescent-onset schizophrenia: Evidence from multimodal brain imaging. Schizophr Res. 2020;222:258-266. doi: 10.1016/j.schres.2020.05.039. Epub 2020 May 24. PMID: 32461088.	No drug
1139	Dai N, Jie H, Duan Y, Xiong P, Xu X, Chen P, Kang M, Li M, Li T, Huang Z, Chen H. Different serum protein factor levels in first-episode drug-naive patients with schizophrenia characterized by positive and negative symptoms. Psychiatry Clin Neurosci. 2020;74(9):472-479. doi: 10.1111/pcn.13078. Epub 2020 Jul 10. PMID: 32478952.	No drug
1140	Basavaraju R, Ithal D, Ramalingaiah AH, Thirthalli J, Mehta UM, Kesavan M. "Apathetic to hypomanic/manic": A case series-illustration of emergent mood symptoms during intermittent theta burst stimulation (iTBS) of cerebellar vermis in schizophrenia with predominant negative symptoms. Schizophr Res. 2020;222:501-502. doi: 10.1016/j.schres.2020.05.037. Epub 2020 Jun 3. PMID: 32505445.	Case
1141	García-Álvarez L, Martínez-Cao C, Bobes-Bascarán T, Portilla A, Courtet P, de la Fuente-Tomás L, Velasco Á, González-Blanco L, Zurrón-Madera P, Fonseca- Pedrero E, Sáiz PA, García-Portilla MP, Bobes J. Validation of a European Spanish-version of the Self-Evaluation of Negative Symptoms (SNS) in patients with schizophrenia. Rev Psiquiatr Salud Ment (Engl Ed). 2020:S1888-9891(20)30036-7. English, Spanish. doi: 10.1016/j.rpsm.2020.04.011. Epub ahead of print. PMID: 32517967.	No drug
1142	Bègue I, Kaiser S, Kirschner M. Pathophysiology of negative symptom dimensions of schizophrenia - Current developments and implications for treatment. Neurosci Biobehav Rev. 2020;116:74-88. doi: 10.1016/j.neubiorev.2020.06.004. Epub 2020 Jun 10. PMID: 32533996.	Review
1143	Okada H, Hirano D, Taniguchi T. Negative symptoms in schizophrenia: Modeling the role of experience factor and expression factor. Asian J Psychiatr. 2020;53:102182. doi: 10.1016/j.ajp.2020.102182. Epub 2020 May 26. PMID: 32544821.	No drug
1144	Trevisan DA, Foss-Feig JH, Naples AJ, Srihari V, Anticevic A, McPartland JC. Autism spectrum disorder and schizophrenia are better differentiated by positive symptoms than negative symptoms. Front Psychiatry. 2020;11:548. doi: 10.3389/fpsyt.2020.00548. PMID: 32595540 PMCID: PMC7301837.	No drug
1145	Lysaker PH, Chernov N, Moiseeva T, Sozinova M, Dmitryeva N, Alyoshin V, Faith LA, Karpenko O, Kostyuk G. Contrasting metacognitive profiles and their association with negative symptoms in groups with schizophrenia, early psychosis and depression in a Russian sample. Psychiatry Res. 2020;291:113177. doi: 10.1016/j.psychres.2020.113177. Epub 2020 Jun 7. PMID: 32615314.	No drug
1146	Collo G, Mucci A, Giordano GM, Merlo Pich E, Galderisi S. Negative symptoms of schizophrenia and dopaminergic transmission: translational models and perspectives opened by iPSC techniques. Front Neurosci. 2020;14:632. doi: 10.3389/fnins.2020.00632. Erratum in: Front Neurosci. 2021;15:729082. PMID: 32625059; PMCID: PMC7315891.	Opinion
1147	Haller N, Hasan A, Padberg F, Brunelin J, da Costa Lane Valiengo L, Palm U. Gamma transcranial alternating current stimulation in patients with negative symptoms in schizophrenia: A case series. Neurophysiol Clin. 2020;50(4):301-304. doi: 10.1016/j.neucli.2020.06.004. Epub 2020 Jul 4. PMID: 32631667.	Case
1148	Sabe M, Zhao N, Kaiser S. Cannabis, nicotine and the negative symptoms of schizophrenia: Systematic review and meta-analysis of observational studies. Neurosci Biobehav Rev. 2020;116:415-425. doi: 10.1016/j.neubiorev.2020.07.007. Epub 2020 Jul 15. PMID: 32679232.	Review
1149	Chao XL, Jiang SZ, Xiong JW, Zhan JQ, Wei B, Chen CN, Yang YJ. Changes of serum insulin-like growth factor-2 response to negative symptom improvements in schizophrenia patients treated with atypical antipsychotics. Curr Med Sci. 2020;40(3):563-569. doi: 10.1007/s11596-020-2214-0. Epub 2020 Jul 17. Erratum in: Curr Med Sci. 2020;40(5):997. PMID: 32681260.	No drug
1150	Duan X, He C, Ou J, Wang R, Xiao J, Li L, Wu R, Zhang Y, Zhao J, Chen H. Reduced hippocampal volume and its relationship with verbal memory and negative symptoms in treatment-naive first-episode adolescent-onset schizophrenia. Schizophr Bull. 2021;47(1):64-74. doi: 10.1093/schbul/sbaa092. PMID: 32691057; PMCID: PMC7825026.	No drug
1151	Wang J, Zhang Y, Liu Z, Yang Y, Zhong Y, Ning X, Zhang Y, Zhao T, Xia L, Geng F, Tao R, Fan M, Ren Z, Liu H. Schizophrenia patients with a metabolically abnormal obese phenotype have milder negative symptoms. BMC Psychiatry. 2020;20(1):410. doi: 10.1186/s12888-020-02809-4. PMID: 32811450; PMCID: PMC7437037.	No drug
1152	Wójciak P, Domowicz K, Andrzejewska M, Rybakowski JK. Negative symptoms in schizophrenia, assessed by the brief negative symptom scale, self-evaluation of negative symptom scale, and social cognition: a gender effect. Int J Psychiatry Clin Pract. 2021;25(3):252-257. doi: 10.1080/13651501.2020.1810278. Epub 2020 Aug 31. PMID: 32862741.	No drug
1153	Vokó Z, Bitter I, Mersich B, Réthelyi J, Molnár A, Pitter JG, Götze Á, Horváth M, Kóczián K, Fonticoli L, Lelli F, Németh B. Using informative prior based on expert opinion in Bayesian estimation of the transition probability matrix in Markov modelling-an example from the cost-effectiveness analysis of the treatment of patients with predominantly negative symptoms of schizophrenia with cariprazine. Cost Eff Resour Alloc. 2020;18:28. doi: 10.1186/s12962-020-00224-w. PMID: 32874137; PMCID: PMC7457290.	Unfocused
1154	Thonon B, Van Aubel E, Lafit G, Della Libera C, Larøi F. Idiographic analyses of motivation and related processes in participants with schizophrenia following a therapeutic intervention for negative symptoms. BMC Psychiatry. 2020;20(1):464. doi: 10.1186/s12888-020-02824-5. PMID: 32977798; PMCID: PMC7517640.	No drug
1155	Riehle M, Böhl MC, Pillny M, Lincoln TM. Efficacy of psychological treatments for patients with schizophrenia and relevant negative symptoms: a meta-analysis. Clin Psychol Eur. 2020;2(3):e2899. doi: 10.32872/cpe.v2i3.2899. PMID: 36398145; PMCID: PMC9645476.	Review
1156	Satodiya R, Palekar N. Synthetic cannabinoids and its association with persistent negative symptoms of schizophrenia. Cureus. 2020;12(9):e10329. doi: 10.7759/cureus.10329. PMID: 33052290; PMCID: PMC7546590.	No drug
1157	Goldring A, Borne S, Hefner A, Thanju A, Khan A, Lindenmayer JP. The psychometric properties of the Self-Evaluation of Negative Symptoms Scale (SNS) in treatment-resistant schizophrenia (TRS). Schizophr Res. 2020;224:159-166. doi: 10.1016/j.schres.2020.08.008. Epub 2020 Oct 15. PMID: 33071071.	No drug

1158	Chang CC, Lin YY, Tzeng NS, Kao YC, Chang HA. Adjunct high-frequency transcranial random noise stimulation over the lateral prefrontal cortex improves negative symptoms of schizophrenia: A randomized, double-blind, sham- controlled pilot study. J Psychiatr Res. 2021;132:151-160. doi: 10.1016/j.jpsychires.2020.10.008. Epub 2020 Oct 16. PMID: 33096356.	No drug
1159	Chao XL, Jiang SZ, Xiong JW, Zhan JQ, Wei B, Chen CN, Yang YJ. Erratum to: Changes of serum insulin-like growth factor-2 response to negative symptom improvements in schizophrenia patients treated with atypical antipsychotics. Curr Med Sci. 2020;40(5):997. doi: 10.1007/s11596-020-2256-3. Erratum for: Curr Med Sci. 2020;40(3):563-569. PMID: 33123913; PMCID: PMC7596002.	Duplicate= 1149
1160	Yu L, Fang X, Chen Y, Wang Y, Wang D, Zhang C. Efficacy of transcranial direct current stimulation in ameliorating negative symptoms and cognitive impairments in schizophrenia: A systematic review and meta-analysis. Schizophr Res. 2020;224:2-10. doi: 10.1016/j.schres.2020.10.006. Epub 2020 Oct 28. PMID: 33129639.	Review
1161	Pelizza L, Maestri D, Leuci E, Quattrone E, Azzali S, Paulillo G, Pellegrini P. Negative symptom configuration within and outside schizophrenia spectrum disorders: results from the "Parma Early Psychosis" program. Psychiatry Res. 2020;294:113519. doi: 10.1016/j.psychres.2020.113519. Epub 2020 Oct 26. PMID: 33130513.	No drug
1162	Gopalakrishnan M, Farchione T, Mathis M, Zhu H, Mehta M, Uppoor R, Younis I. Shortened Positive and Negative Symptom Scale as an alternate clinical endpoint for acute schizophrenia trials: Analysis from the US Food & Drug Administration. Psychiatr Res Clin Pract. 2020;3(1):38-45. doi: 10.1176/appi.prcp.20200003. PMID: 36101554; PMCID: PMC9175851.	No drug
1163	Krynicki CR, Dazzan P, Pariante CM, Barnes NM, Vincent RC, Roberts A, Giordano A, Watson A, Suckling J, Barnes TRE, Husain N, Jones PB, Joyce E, Lawrie SM, Lewis S, Deakin B, Upthegrove R; BeneMin Study team. Deconstructing depression and negative symptoms of schizophrenia; differential and longitudinal immune correlates, and response to minocycline treatment. Brain Behav Immun. 2021;91:498-504. doi: 10.1016/j.bbi.2020.10.026. Epub 2020 Nov 5. PMID: 33161162.	Unfocused
1164	Wójciak P, Domowicz K, Rybakowski JK. Metabolic indices in schizophrenia: Association of negative symptoms with higher HDL cholesterol in female patients. World J Biol Psychiatry. 2021;22(7):552-556. doi: 10.1080/15622975.2020.1849796. Epub 2020 Dec 1. PMID: 33176545.	No drug
1165	Umbricht D, Cheng WY, Lipsmeier F, Bamdadian A, Lindemann M. Deep learning-based human activity recognition for continuous activity and gesture monitoring for schizophrenia patients with negative symptoms. Front Psychiatry. 2020;11:574375. doi: 10.3389/fpsyt.2020.574375 PMID: 33192706; PMCID: PMC7525025.	No drug
1166	Okada H, Hirano D, Taniguchi T. Single versus dual pathways to functional outcomes in schizophrenia: Role of negative symptoms and cognitive function. Schizophr Res Cogn. 2020;23:100191. doi: 10.1016/j.scog.2020.100191. PMID: 33204652; PMCID: PMC7648174.	No drug
1167	Pelizza L, Azzali S, Paterlini F, Garlassi S, Scazza I, Chiri LR, Poletti M, Pupo S, Raballo A. Negative symptom dimensions in first episode psychosis: Is there a difference between schizophrenia and non-schizophrenia spectrum disorders? Early Interv Psychiatry. 2021;15(6):1513-1521. doi: 10.1111/eip.13088. Epub 2020 Nov 25. PMID: 33238327.	No drug
1168	Kirschner M, Schmidt A, Hodzic-Santor B, Burrer A, Manoliu A, Zeighami Y, Yau Y, Abbasi N, Maatz A, Habermeyer B, Abivardi A, Avram M, Brandl F, Sorg C, Homan P, Riecher-Rössler A, Borgwardt S, Seifritz E, Dagher A, Kaiser S. Orbitofrontal-striatal structural alterations linked to negative symptoms at different stages of the schizophrenia spectrum. Schizophr Bull. 2021;47(3):849-863. doi: 10.1093/schbul/sbaa169. PMID: 33257954; PMCID: PMC8084448.	No drug
1169	Granholm E, Holden J, Dwyer K, Mikhael T, Link P, Depp C. Mobile-assisted cognitive behavioral therapy for negative symptoms: open single-arm trial with schizophrenia patients. JMIR Ment Health. 2020;7(12):e24406. doi: 10.2196/24406. PMID: 33258792; PMCID: PMC7738249.	No drug
1170	Haller N, Hasan A, Padberg F, da Costa Lane Valiengo L, Brunelin J, Palm U. Gamma transcranial alternating current stimulation for treatment of negative symptoms in schizophrenia: Report of two cases. Asian J Psychiatr. 2020;54:102423. doi: 10.1016/j.ajp.2020.102423. Epub 2020 Sep 15. PMID: 33271707.	Case
1171	Mahmood Z, Van Patten R, Keller AV, Lykins HC, Perivoliotis D, Granholm E, Twamley EW. Reducing negative symptoms in schizophrenia: Feasibility and acceptability of a combined cognitive-behavioral social skills training and compensatory cognitive training intervention. Psychiatry Res. 2021;295:113620. doi: 10.1016/j.psychres.2020.113620. Epub 2020 Dec 1. PMID: 33290939; PMCID: PMC7779756.	No drug
1172	Yolland COB, Carruthers SP, Toh WL, Neill E, Sumner PJ, Thomas EHX, Tan EJ, Gurvich C, Phillipou A, Van Rheenen TE, Rossell SL. The relationship between negative symptoms and both emotion management and non-social cognition in schizophrenia spectrum disorders. J Int Neuropsychol Soc. 2021;27(9):916-928. doi: 10.1017/S1355617720001290. Epub 2020 Dec 21. PMID: 33342446.	No drug
1173	Hosseininasab M, Zarghami M, Mazhari S, Salehifar E, Moosazadeh M, Fariborzifar A, Babaeirad S, Hendouei N. Nanocurcumin as an add-on to antipsychotic drugs for treatment of negative symptoms in patients with chronic schizophrenia: A randomized, double-blind, placebo-controlled study. J Clin Psychopharmacol. 2021;41(1):25-30. doi: 10.1097/JCP.0000000000001324. PMID: 33347019.	**Included**
1174	Samaei A, Moradi K, Bagheri S, Ashraf-Ganjouei A, Alikhani R, Mousavi SB, Rezaei F, Akhondzadeh S. Resveratrol adjunct therapy for negative symptoms in patients with stable schizophrenia: A double-blind, randomized placebo- controlled trial. Int J Neuropsychopharmacol. 2020;23(12):775-782. doi: 10.1093/ijnp/pyaa006. PMID: 33372679; PMCID: PMC7770519.	**Included**
1175	Malashenkova IK, Ushakov VL, Zakharova NV, Krynskiy SA, Ogurtsov DP, Hailov NA, Chekulaeva EI, Ratushnyy AY, Kartashov SI, Kostyuk GP, Didkovsky NA. Neuro-immune aspects of schizophrenia with severe negative symptoms: new diagnostic markers of disease phenotype. Sovrem Tekhnologii Med. 2021;13(6):24-33. doi: 10.17691/stm2021.13.6.03. Epub 2021 Dec 28. PMID: 35265356; PMCID: PMC8858398.	No drug
1176	Xu H, Wang J, Zhou Y, Chen D, Xiu M, Wang L, Zhang X. BDNF affects the mediating effect of negative symptoms on the relationship between age of onset and cognition in patients with chronic schizophrenia. Psychoneuroendocrinology. 2021;125:105121. doi: 10.1016/j.psyneuen.2020.105121. Epub 2020 Dec 26. PMID: 33387927.	No drug
1177	Rekhi G, Ang MS, Lee J. Association between negative symptom domains and happiness in schizophrenia. Gen Hosp Psychiatry. 2021;68:83-89. doi: 10.1016/j.genhosppsych.2020.12.016. Epub 2020 Dec 28. PMID: 33412469.	No drug
1178	Smulevich AB, Ivanov SV, Yakhin KK, Voronova EI, Kharkova GS, Skurygina EI, Konohova MV, Beybalaeva TZ, Katok AA. Карипразин в терапии шизофрении с преобладанием негативных симптомов: начальные эффекты лечения – Kariprazin v terapii shizofrenii s preobladaniem negativnykh simptomov: initsial'nye effekty lecheniya (observatsionnoe issledovanie) [Cariprazine in schizophrenia with predominantly negative symptoms: early effects of therapy (observational study)]. Zh Nevrol Psikhiatr Im S S Korsakova. 2020;120(12):67-72. Russian. doi: 10.17116/jnevro202012012167. PMID: 33459543.	Open
1179	Li R, Hei G, Yang Y, Wu R, Zhao J. Research advances in add-on treatment for negative symptoms and cognitive dysfunction in schizophrenia Zhong Nan Da Xue Xue Bao Yi Xue Ban. 2020;45(12):1457-1463. English, Chinese. doi: 10.11817/j.issn.1672-7347.2020.190556. PMID: 33473003. 李然然，黑钢瑞，杨叶，吴仁容，赵靖平. 精神分裂症阴性症状及认知障碍增效治疗的研究进展. 中南大学学报（医学版）2020;45(12):1457-63.	Review
1180	Kousar T, Riaz MN. Effect of positive and negative symptoms on schizophrenia related quality of life of hospitalized schizophrenic patients at Sargodha and Lahore: Moderating role of neuropsychiatric symptoms. J Pak Med Assoc. 2021;71(1(A)):4-7. doi: 10.47391/JPMA.1105. PMID 33484508.	No drug
1181	Zahid A, Best MW. Stigma towards individuals with schizophrenia: Examining the effects of negative symptoms and diagnosis awareness on preference for social distance. Psychiatry Res. 2021;297:113724. doi: 10.1016/j.psychres.2021.113724. Epub 2021 Jan 15. PMID: 33486270.	No drug
1182	Haller N, Hasan A, Padberg F, Strube W, da Costa Lane Valiengo L, Brunoni AR, Brunelin J, Palm U. Transkranielle elektrische Hirnstimulationsverfahren zur Behandlung der Negativsymptomatik bei Schizophrenie [Transcranial electrical brain stimulation methods for treatment of negative symptoms in schizophrenia]. Nervenarzt. 2022;93(1):41-50. German. doi: 10.1007/s00115-021-01065-5. Epub 2021 Jan 25. PMID: 33492411; PMCID: PMC8763819.	No drug

1183	Tuominen L, DeCross SN, Boeke E, Cassidy CM, Freudenreich O, Shinn AK, Tootell RBH, Holt DJ. Neural abnormalities in fear generalization in schizophrenia and associations with negative symptoms. Biol Psychiatry Cogn Neurosci Neuroimaging. 2021;6(12):1165-1175. doi: 10.1016/j.bpsc.2021.01.006. Epub 2021 Jan 29. PMID: 33524600.	No drug
1184	Rancans E, Dombi ZB, Mátrai P, Barabássy Á, Sebe B, Skrivele I, Németh G. The effectiveness and safety of cariprazine in schizophrenia patients with negative symptoms and insufficient effectiveness of previous antipsychotic therapy: an observational study. Int Clin Psychopharmacol. 2021;36(3):154-161. doi: 10.1097/YIC.0000000000000351. PMID: 33560040; PMCID: PMC8011502.	Open
1185	Bation R, Magnin C, Poulet E, Mondino M, Brunelin J. Intermittent theta burst stimulation for negative symptoms of schizophrenia-A double-blind, sham- controlled pilot study. NPJ Schizophr. 2021;7(1):10. doi: 10.1038/s41537-021-00138-3. PMID: 33580032; PMCID: PMC7880987.	No drug
1186	Galderisi S, Mucci A, Dollfus S, Nordentoft M, Falkai P, Kaiser S, Giordano GM, Vandevelde A, Nielsen MØ, Glenthøj LB, Sabé M, Pezzella P, Bitter I, Gaebel W. EPA guidance on assessment of negative symptoms in schizophrenia. Eur Psychiatry. 2021;64(1):e23. doi: 10.1192/j.eurpsy.2021.11. PMID: 33597064; PMCID: PMC8080207.	No drug
1187	Sehatpour P, Avissar M, Kantrowitz JT, Corcoran CM, De Baun HM, Patel GH, Girgis RR, Brucato G, Lopez-Calderon J, Silipo G, Dias E, Martinez A, Javitt DC. Deficits in pre-attentive processing of spatial location and negative symptoms in subjects at clinical high risk for schizophrenia. Front Psychiatry. 2021;11:629144. doi: 10.3389/fpsyt.2020.629144. PMID: 33603682; PMCID: PMC7884473.	No drug
1188	Căpățînă OO, Micluția IV, Fadgyas-Stănculete M. Current perspectives in treating negative symptoms of schizophrenia: A narrative review (Review). Exp Ther Med. 2021;21(3):276. doi: 10.3892/etm.2021.9707. Epub 2021 Jan 25. PMID: 33603883; PMCID: PMC7851661.	Review
1189	Strauss GP, Bartolomeo LA, Harvey PD. Avolition as the core negative symptom in schizophrenia: relevance to pharmacological treatment development. NPJ Schizophr. 2021;7(1):16. doi: 10.1038/s41537-021-00145-4. PMID: 33637748; PMCID: PMC7910596.	No drug
1190	Ulrich S, Messer T. Review and meta-analysis of add-on tranylcypromine with antipsychotic drugs for the treatment of schizophrenia with predominant negative symptoms: a restoration of evidence. Curr Med Res Opin. 2021;37(7):1233-1248. doi: 10.1080/03007995.2021.1895095. Epub 2021 May 20. PMID: 33651656.	Review
1191	Dharani R, Goyal N, Mukherjee A, Umesh S. Adjuvant High-Definition Transcranial Direct Current Stimulation for Negative Symptoms in Schizophrenia: A Pilot Study. J ECT. 2021;37(3):195-201. doi: 10.1097/YCT.0000000000000756. PMID: 33661184.	No drug
1192	Okada H, Hirano D, Taniguchi T. Impact of Negative Symptom Domains and Other Clinical Characteristics on Functional Outcomes in Patients with Schizophrenia. Schizophr Res Treatment. 2021;2021:8864352. doi: 10.1155/2021/8864352. PMID: 33688435; PMCID: PMC7914085.	No drug
1193	Umbricht D, Abt M, Tamburri P, Chatham C, Holiga Š, Frank MJ, Collins AGE, Walling DP, Mofsen R, Gruener D, Gertsik L, Sevigny J, Keswani S, Dukart J. Proof-of-mechanism study of the phosphodiesterase 10 inhibitor RG7203 in patients with schizophrenia and negative symptoms. Biol Psychiatry Glob Open Sci. 2021;1(1):70-77. doi: 10.1016/j.bpsgos.2021.03.001. PMID: 36324430; PMCID: PMC9616307.	Unfocused
1194	Galderisi S, Kaiser S, Bitter I, Nordentoft M, Mucci A, Sabé M, Giordano GM, Nielsen MØ, Glenthøj LB, Pezzella P, Falkai P, Dollfus S, Gaebel W. EPA guidance on treatment of negative symptoms in schizophrenia. Eur Psychiatry. 2021;64(1):e21. doi: 10.1192/j.eurpsy.2021.13. PMID: 33726883; PMCID: PMC8057437.	No drug
1195	Gan H, Zhu J, Zhuo K, Zhang J, Tang Y, Qian Z, Xiang Q, Li X, Zhu Y, Wang J, Wang J, Liu D. High frequency repetitive transcranial magnetic stimulation of dorsomedial prefrontal cortex for negative symptoms in patients with schizophrenia: A double-blind, randomized controlled trial. Psychiatry Res. 2021;299:113876. doi: 10.1016/j.psychres.2021.113876. Epub 2021 Mar 16. PMID: 33770710.	No drug
1196	Yang Z, Lee SH, Abdul Rashid NA, See YM, Dauwels J, Tan BL, Lee J. Predicting real-world functioning in schizophrenia: The relative contributions of neurocognition, functional capacity, and negative symptoms. Front Psychiatry. 2021;12:639536. doi: 10.3389/fpsyt.2021.639536. PMID: 33815171; PMCID: PMC8017150.	No drug
1197	Rao NP, Ramachandran P, Jacob A, Joseph A, Thonse U, Nagendra B, Chako DM, Shiri S, Hassan H, Sreenivas V, Maran S, Durgam D, Nandakumar K, Varambally S, Gangadhar BN. Add on yoga treatment for negative symptoms of schizophrenia: A multi-centric, randomized controlled trial. Schizophr Res. 2021;231:90-97. doi: 10.1016/j.schres.2021.03.021. Epub 2021 Apr 5. PMID: 33831770.	No drug
1198	Strauss GP. A bioecosystem theory of negative symptoms in schizophrenia. Front Psychiatry. 2021;12:655471. doi: 10.3389/fpsyt.2021.655471. PMID: 33841217; PMCID: PMC8026872.	No drug
1199	Hajj A, Hallit S, Chamoun K, Sacre H, Obeid S, Haddad C, Dollfus S, Khabbaz LR. Negative symptoms in schizophrenia: correlation with clinical and genetic factors. Pharmacogenomics. 2021;22(7):389-399. doi: 10.2217/pgs-2020-0171. Epub 2021 Apr 16. PMID: 33858192.	No drug
1200	Jeganathan J, Breakspear M. An active inference perspective on the negative symptoms of schizophrenia. Lancet Psychiatry. 2021;8(8):732-738. doi: 10.1016/S2215-0366(20)30527-7. Epub 2021 Apr 15. PMID: 33865502.	No drug
1201	Rampino A, Torretta S, Gelao B, Veneziani F, Iacoviello M, Marakhovskaya A, Masellis R, Andriola I, Sportelli L, Pergola G, Minelli A, Magri C, Gennarelli M, Vita A, Beaulieu JM, Bertolino A, Blasi G. Evidence of an interaction between FXR1 and GSK3β polymorphisms on levels of Negative Symptoms of Schizophrenia and their response to antipsychotics. Eur Psychiatry. 2021;64(1):e39. doi: 10.1192/j.eurpsy.2021.26. PMID: 33866994; PMCID: PMC8260562.	Unfocused
1202	Zhu R, Wang D, Wei G, Wang J, Zhou H, Xu H, Wang W, Wei S, Chen D, Xiu M, Wang L, Zhang XY. Association of negative symptoms with cognitive impairment in Chinese Han patients with chronic schizophrenia. Int J Psychiatry Clin Pract. 2021;25(3):292-298. doi: 10.1080/13651501.2021.1912357. Epub 2021 Apr 20. PMID: 33879034.	No drug
1203	Strauss GP, Macdonald KI, Ruiz I, Raugh IM, Bartolomeo LA, James SH. The impact of the COVID-19 pandemic on negative symptoms in individuals at clinical high-risk for psychosis and outpatients with chronic schizophrenia. Eur Arch Psychiatry Clin Neurosci. 2022;272(1):17-27. doi: 10.1007/s00406-021-01260-0. Epub 2021 Apr 21. PMID: 33881621; PMCID: PMC8057945.	No drug
1204	Recio-Barbero M, Segarra R, Zabala A, González-Fraile E, González-Pinto A, Ballesteros J. Cognitive enhancers in schizophrenia: A systematic review and meta-analysis of alpha-7 nicotinic acetylcholine receptor agonists for cognitive deficits and negative symptoms. Front Psychiatry. 2021;12:631589. doi: 10.3389/fpsyt.2021.631589. PMID: 33889097; PMCID: PMC8055861.	Review
1205	Sabe M, Zhao N, Crippa A, Strauss GP, Kaiser S. Intranasal oxytocin for negative symptoms of schizophrenia: Systematic review, meta-analysis, and dose- response meta-analysis of randomized controlled trials. Int J Neuropsychopharmacol. 2021;24(8):601-614. doi: 10.1093/ijnp/pyab020. PMID: 33890987; PMCID: PMC8378078.	Review
1206	Tam MHW, Ling-Ling W, Cheng KM, Wong JOY, Cheung EFC, Lui SSY, Chan RCK. Latent structure of self-report negative symptoms in patients with schizophrenia: A preliminary study. Asian J Psychiatr. 2021;61:102680. doi: 10.1016/j.ajp.2021.102680. Epub 2021 May 11. PMID: 34000499.	No drug
1207	Rekhi G, Ang MS, Chan YH, Fernandez-Egea E, Kirkpatrick B, Lee J. Defining negative symptoms remission in schizophrenia using the Brief Negative Symptom Scale. Rev Psiquiatr Salud Ment (Engl Ed). 2021:S1888-9891(21)00060-4. English, Spanish. doi: 10.1016/j.rpsm.2021.05.003. Epub ahead of print 2021 May 29. PMID: 34058418.	No drug
1208	Căpățînă O, Stănculete MF, Micluția I. Behavioral outputs of negative symptom domains of schizophrenia. Exp Ther Med. 2021;22(2):805. doi: 10.3892/etm.2021.10237. Epub 2021 May 26. PMID: 34093761; PMCID: PMC8170643.	No drug
1209	Kantrowitz JT. How do we address treating the negative symptoms of schizophrenia pharmacologically? Expert Opin Pharmacother. 2021;22(14):1811-1813. doi: 10.1080/14656566.2021.1939677. Epub 2021 Jun 16. PMID: 34130578.	Review
1210	Herniman SE, Phillips LJ, Wood SJ, Cotton SM, Liemburg EJ, Allott KA. Interrelationships between depressive symptoms and positive and negative symptoms of recent onset schizophrenia spectrum disorders: A network analytical approach. J Psychiatr Res. 2021;140:373-380. doi: 10.1016/j.jpsychires.2021.05.038. Epub 2021 Jun 4. PMID: 34144441.	No drug

1211	Alizadeh M, Delborde Y, Ahmadpanah M, Seifrabiee MA, Jahangard L, Bazzazi N, Brand S. Non-linear associations between retinal nerve fibre layer (RNFL) and positive and negative symptoms among men with acute and chronic schizophrenia spectrum disorder. J Psychiatr Res. 2021;141:81-91. doi: 10.1016/j.jpsychires.2021.06.007. Epub 2021 Jun 7. PMID: 34182380.	No drug
1212	Fan YS, Li H, Guo J, Pang Y, Li L, Hu M, Li M, Wang C, Sheng W, Liu H, Gao Q, Chen X, Zong X, Chen H. Tracking positive and negative symptom improvement in first-episode schizophrenia treated with risperidone using individual-level functional connectivity. Brain Connect. 2022;12(5):454-464. doi: 10.1089/brain.2021.0061. Epub 2021 Sep 13. PMID: 34210149.	Unfocused
1213	Bonfils KA, Luther L, Lysaker PH. Metacognition & social cognition differentially influence experiential & expressive negative symptoms in schizophrenia. Schizophr Res. 2021;233:13-15. doi: 10.1016/j.schres.2021.06.020. Epub 2021 Jun 30. PMID: 34216940.	No drug
1214	Wu Q, Wang X, Wang Y, Long YJ, Zhao JP, Wu RR. Developments in biological mechanisms and treatments for negative symptoms and cognitive dysfunction of schizophrenia. Neurosci Bull. 2021;37(11):1609-1624. doi: 10.1007/s12264-021-00740-6. Epub 2021 Jul 5. PMID: 34227057; PMCID: PMC8566616.	No drug
1215	Pioch A, Spreen M, Bokern H. Muziektherapie voor negatieve symptomen bij schizofrenie: vijf n=1-studies in een tbs-kliniek [Music therapy for negative symptoms in patients with schizophrenia: five systemic N-of-1 trials in a high- security psychiatric hospital]. Tijdschr Psychiatr. 2021;63(6):412-418. Dutch. PMID: 34231859.	No drug
1216	Reichard HA, Schiffer HH, Monenschein H, Atienza JM, Corbett G, Skaggs AW, Collia DR, Ray WJ, Serrats J, Bliesath J, Kaushal N, Lam BP, Amador-Arjona A, Rahbaek L, McConn DJ, Mulligan VJ, Brice N, Gaskin PLR, Cilia J, Hitchcock S. Discovery of TAK-041: a potent and selective GPR139 agonist explored for the treatment of negative symptoms associated with schizophrenia. J Med Chem. 2021;64(15):11527-11542. doi: 10.1021/acs.jmedchem.1c00820. Epub 2021 Jul 14. PMID: 34260228.	Animal
1217	Madzarac Z, Tudor L, Sagud M, Nedic Erjavec G, Mihaljevic Peles A, Pivac N. The associations between COMT and MAO-B genetic variants with negative symptoms in patients with schizophrenia. Curr Issues Mol Biol. 2021;43(2):618-636. doi: 10.3390/cimb43020045. PMID: 34287249; PMCID: PMC8928957.	Review
1218	García-Portilla MP, García-Álvarez L, de la Fuente-Tomás L, Dal Santo F, Velasco A, González-Blanco L, Zurrón-Madera P, Fonseca-Pedrero E, Bobes-Bascarán MT, Sáiz PA, Bobes J. Spanish Validation of the MAP-SR: Two heads better than one for the assessment of negative symptoms of schizophrenia. Psicothema. 2021;33(3):473-480. doi: 10.7334/psicothema2020.457. PMID: 34297678.	Opinion
1219	Bulubas L, Goerigk S, Gomes JS, Brem AK, Carvalho JB, Pinto BS, Elkis H, Gattaz WF, Padberg F, Brunoni AR, Valiengo L. Cognitive outcomes after tDCS in schizophrenia patients with prominent negative symptoms: Results from the placebo-controlled STARTS trial. Schizophr Res. 2021;235:44-51. doi: 10.1016/j.schres.2021.07.008. Epub 2021 Jul 22. PMID: 34304146.	No drug
1220	Shen H, Zhang L, Li Y, Zheng D, Du L, Xu F, Xu C, Liu Y, Shen J, Li Z, Cui D. Mindfulness-based intervention improves residual negative symptoms and cognitive impairment in schizophrenia: a randomized controlled follow-up study. Psychol Med. 2023;53(4):1390-1399. doi: 10.1017/S0033291721002944. Epub 2021 Aug 2. PMID: 36468948; PMCID: PMC10009398.	No drug
1221	Collo G, Mucci A, Giordano GM, Merlo Pich E, Galderisi S. Corrigendum: Negative symptoms of schizophrenia and dopaminergic transmission: Translational models and perspectives opened by iPSC techniques. Front Neurosci. 2021;15:729082. doi: 10.3389/fnins.2021.729082. Erratum for: Front Neurosci. 2020 Jun 18;14:632. PMID: 34335179; PMCID: PMC8322975.	Duplicate= 1146
1222	Ruiz-Castañeda P, Daza-González MT, Santiago-Molina E. Negative symptoms and behavioral alterations associated with dorsolateral prefrontal syndrome in patients with schizophrenia. J Clin Med. 2021;10(15):3417. doi: 10.3390/jcm10153417. PMID: 34362200; PMCID: PMC8348852.	No drug
1223	Ebrahimi A, Poursharifi H, Dolatshahi B, Rezaee O, Hassanabadi HR, Naeem F. The cognitive model of negative symptoms in schizophrenia: A hierarchical component model with PLS-SEM. Front Psychiatry. 2021;12:707291. doi: 10.3389/fpsyt.2021.707291. PMID: 34366940; PMCID: PMC8339582.	No drug
1224	Quek YF, Yang Z, Dauwels J, Lee J. The impact of negative symptoms and neurocognition on functioning in MDD and schizophrenia. Front Psychiatry. 2021;12:648108. doi: 10.3389/fpsyt.2021.648108. PMID: 34381384; PMCID: PMC8350050.	No drug
1225	Zhu C, Zheng M, Ali U, Xia Q, Wang Z, Chenlong, Yao L, Chen Y, Yan J, Wang K, Chen J, Zhang X. Association between abundance of Haemophilus in the gut microbiota and negative symptoms of schizophrenia. Front Psychiatry. 2021;12:685910. doi: 10.3389/fpsyt.2021.685910. PMID: 34393849; PMCID: PMC8362742.	No drug
1226	Németh G, Dombi ZB, Laszlovszky I, Barabássy Á. Addressing negative symptoms of schizophrenia pharmacologically with cariprazine: evidence from clinical trials, a real-world study, and clinical cases. Expert Opin Pharmacother. 2022;23(12):1467-1468. doi: 10.1080/14656566.2021.1968827. Epub 2021 Aug 25. PMID: 34431422.	Review
1227	Morozova MA, Potanin SS, Rupchev GE, Burminskiy DS, Lepilkina TA, Beniashvili AG. Влияние блокады холинестеразы на негативные расстройства при шизофрении Vliyanie blokady kholinesterazy na negativnye rasstroistva pri shizofrenii [An effect of cholinesterase blockade on negative symptoms in schizophrenia]. Zh Nevrol Psikhiatr Im S S Korsakova. 2021;121(8):37-44. Russian. doi: 10.17116/jnevro202112108137. PMID: 34481434.	Open
1228	Kermit E, Yamada R, Hirakawa T, Kimura H. Therapeutic potential of TAK-071, a muscarinic M1 receptor positive allosteric modulator with low cooperativity, for the treatment of cognitive deficits and negative symptoms associated with schizophrenia. Neurosci Lett. 2021;764:136240. doi: 10.1016/j.neulet.2021.136240. Epub 2021 Sep 10. PMID: 34509568.	Animal
1229	Wen N, Chen L, Miao X, Zhang M, Zhang Y, Liu J, Xu Y, Tong S, Tang W, Wang M, Liu J, Zhou S, Fang X, Zhao K. Effects of high-frequency rTMS on negative symptoms and cognitive function in hospitalized patients with chronic schizophrenia: A double-blind, sham-controlled pilot trial. Front Psychiatry. 2021;12:736094. doi: 10.3389/fpsyt.2021.736094. PMID: 34539472; PMCID: PMC8446365.	No drug
1230	Zhu L, Zhang W, Zhu Y, Mu X, Zhang Q, Wang Y, Cai J, Xie B. Cerebellar theta burst stimulation for the treatment of negative symptoms of schizophrenia: A multicenter, double-blind, randomized controlled trial. Psychiatry Res. 2021;305:114204. doi: 10.1016/j.psychres.2021.114204. Epub 2021 Sep 5. PMID: 34587567.	No drug
1231	Demyttenaere K, Leenaerts N, Acsai K, Sebe B, Laszlovszky I, Barabássy Á, Fonticoli L, Szatmári B, Earley W, Németh G, Correll CU. Disentangling the symptoms of schizophrenia: Network analysis in acute phase patients and in patients with predominant negative symptoms. Eur Psychiatry. 2021;65(1):e18. doi: 10.1192/j.eurpsy.2021.2241. PMID: 34641986; PMCID: PMC8926909.	No drug
1232	Basavaraju R, Ithal D, Thanki MV, Ramalingaiah AH, Thirthalli J, Reddy RP, Brady RO Jr, Halko MA, Bolo NR, Keshavan MS, Pascual-Leone A, Mehta UM, Kesavan M. Intermittent theta burst stimulation of cerebellar vermis enhances fronto-cerebellar resting state functional connectivity in schizophrenia with predominant negative symptoms: A randomized controlled trial. Schizophr Res. 2021;238:108-120. doi: 10.1016/j.schres.2021.10.005. Epub 2021 Oct 12. PMID: 34653740; PMCID: PMC8662658.	No drug
1233	Raucher-Chéné D, Thibaudeau E, Sauvé G, Lavigne KM, Lepage M. Understanding others as a mediator between verbal memory and negative symptoms in schizophrenia-spectrum disorder. J Psychiatr Res. 2021;143:429-435. doi: 10.1016/j.jpsychires.2021.10.007. Epub 2021 Oct 11. PMID: 34656875.	No drug
1234	Goldsmith DR, Massa N, Miller BJ, Miller AH, Duncan E. The interaction of lipids and inflammatory markers predict negative symptom severity in patients with schizophrenia. NPJ Schizophr. 2021;7(1):50. doi: 10.1038/s41537-021-00179-8. PMID: 34671033; PMCID: PMC8528914.	No drug
1235	Khonsari NM, Badrfam R, Mohammdi MR, Rastad H, Etemadi F,Vafaei Z, Zandifar A. Effect of aerobic exercise as adjuncttherapy on the improvement of negative symptoms and cognitive impairment in patients with schizophrenia: A randomized, case-control clinical trial. J Psychosoc Nurs Ment Health Serv.2022;60(5):38-43. doi: 10.3928/02793695-20211014-03. Epub 2021Oct 25. PMID: 34677118.	No drug
1236	Kathiravan S, Shouan A, Kumari S, Harshit K, Singh SM. The utility of HD-tDCS as add on treatment for negative symptoms in schizophrenia: A case report. Psychiatr Danub. 2021;33(3):366-367. doi: 10.24869/psyd.2021.366. PMID: 34795182	Case

1237	Banazadeh M, Mehrabani M, Banazadeh N, Dabaghzadeh F,Shahabi F. Evaluating the effect of black myrobalan on cognitive,positive, and negative symptoms in patients with chronic schizophrenia: A randomized, double-blind, placebo-controlled trial. Phytother Res. 2022;36(1):543-550. doi: 10.1002/ptr.7340. Epub2021 Nov 23. PMID: 34814232.	**Included**
1238	Novo A, Fonsêca J, Barroso B, Guimarães M, Louro A, FernandesH, Lopes RP, Leitão P. Virtual reality rehabilitation's impact on negative symptoms and psychosocial rehabilitation in schizophrenia spectrum disorder: A systematic review. Healthcare (Basel). 2021;9(11):1429. doi: 10.3390/healthcare9111429. PMID:34828476; PMCID: PMC8621037.	Review
1239	Chang CC, Huang CC, Chung YA, Im JJ, Lin YY, Ma CC, TzengNS, Chang HA. Online left-hemispheric in-phase frontoparietal theta tACS for the treatment of negative symptoms of schizophrenia. J Pers Med. 2021;11(11):1114. doi:10.3390/jpm11111114. PMID: 34834466; PMCID: PMC8625275.	No drug
1240	Bugarski-Kirola D, Arango C, Fava M, Nasrallah H, Liu IY, AbbsB, Stankovic S. Pimavanserin for negative symptoms ofschizophrenia: results from the ADVANCE phase 2 randomised, placebo-controlled trial in North America and Europe. Lancet Psychiatry. 2022;9(1):46-58. doi: 10.1016/S2215-0366(21)00386-2.Epub 2021 Nov 30. PMID: 34861170.	**Included**
1241	Vasiliu O. Case report: Cariprazine efficacy in young patients diagnosed with schizophrenia with predominantly negative symptoms. Front Psychiatry. 2021;12:786171. doi:10.3389/fpsyt.2021.786171. PMID: 34880797; PMCID:PMC8645548.	Case
1242	Naguy A. Levomilnacipran for negative symptom domain schizophrenia. Prim Care Companion CNS Disord.2021;23(6):20l02873. doi: 10.4088/PCC.20l02873. PMID:34890499.	Case
1243	Navalón P, Sahuquillo-Leal R, Moreno-Giménez A, Salmerón L,Benavent P, Sierra P, Cañada Y, Cañada-Martínez A, Berk M, García-Blanco A. Attentional engagement and inhibitory control according to positive and negative symptoms in schizophrenia: An emotional antisaccade task. Schizophr Res. 2022;239:142-150. doi:10.1016/j.schres.2021.11.044. Epub 2021 Dec 7. PMID: 34891078.	No drug
1244	Haguiara B, Koga G, Diniz E, Fonseca L, Higuchi CH, Kagan S,Lacerda A, Correll CU, Gadelha A. What is the best latent structure of negative symptoms in schizophrenia? A systematic review. Schizophr Bull Open. 2021;2(1):sgab013. doi:10.1093/schizbullopen/sgab013. PMID: 34901862; PMCID:PMC8650068.	Review
1245	Cathomas F, Klaus F, Guetter K, Seifritz E, Hartmann-Riemer MN, Tobler PN, Kaiser S, Kaliuzhna M. Associations between negative symptoms and effort discounting in patients with schizophrenia and major depressive disorder. Schizophr Bull Open. 2021;2(1):sgab022. doi: 10.1093/schizbullopen/sgab022. PMID:34901865; PMCID: PMC8650075.	No drug
1246	Rajagopal L, Ryan C, Elzokaky A, Burstein ES, Meltzer HY.Pimavanserin augments the efficacy of atypical antipsychotic drugs in a mouse model of treatment-refractory negative symptoms of schizophrenia. Behav Brain Res. 2022;422:113710. doi:10.1016/j.bbr.2021.113710. Epub 2021 Dec 11. PMID: 34906610.	Animal
1247	Pelizza L, Leuci E, Maestri D, Quattrone E, Azzali S, Paulillo G, Pellegrini P. Longitudinal persistence of negative symptoms in young individuals with first episode schizophrenia: a 24-month multi-modal program follow-up. Nord J Psychiatry. 2022;76(7):530-538. doi: 10.1080/08039488.2021.2015431. Epub 2021 Dec 22.PMID: 34936855.	No drug
1248	Giordano GM, Perrottelli A, Mucci A, Di Lorenzo G, Altamura M, Bellomo A, Brugnoli R, Corrivetti G, Girardi P, Monteleone P, Niolu C, Galderisi S, Maj M, The Italian Network For Research On Psychoses. Investigating the relationships of P3b with negative symptoms and neurocognition in subjects with chronic schizophrenia. Brain Sci. 2021;11(12):1632. doi:10.3390/brainsci11121632. PMID: 34942934; PMCID:PMC8699055.	No drug
1249	Wu S, Gao C, Han F, Cheng H. Histamine H1 receptor in basal forebrain cholinergic circuit: A novel target for the negative symptoms of schizophrenia? Neurosci Bull. 2022;38(5):558-560.doi: 10.1007/s12264-021-00811-8. Epub 2021 Dec 27. PMID:34958430; PMCID: PMC9106766.	Opinion
1250	Russo M, Repisti S, Blazhevska Stoilkovska B, Jerotic S, Ristic I, Mesevic Smajic E, Uka F, Arenliu A, Bajraktarov S, Dzubur Kulenovic A, Injac Stevovic L, Priebe S, Jovanovic N. Structure of negative symptoms in schizophrenia: An unresolved issue. Front Psychiatry. 2021;12:785144. doi: 10.3389/fpsyt.2021.785144.Erratum in: Front Psychiatry. 2022;13:885883. PMID: 34970168;PMCID: PMC8712471.	No drug
1251	Giordano GM, Brando F, Perrottelli A, Di Lorenzo G, Siracusano A,Giuliani L, Pezzella P, Altamura M, Bellomo A, Cascino G, DelCasale A, Monteleone P, Pompili M, Galderisi S, Maj M; Italian Network for Research on Psychoses. Tracing links between early auditory information processing and negative symptoms in schizophrenia: An ERP study. Front Psychiatry. 2021;12:790745.doi: 10.3389/fpsyt.2021.790745. PMID: 34987433; PMCID:PMC8721527.	No drug
1252	Pedersen IN, Bonde LO, Hannibal NJ, Nielsen J, Aagaard J, Gold C, Rye Bertelsen L, Jensen SB, Nielsen RE. Music therapy vs. music listening for negative symptoms in schizophrenia: Randomized, controlled, assessor- and patient-blinded trial. Front Psychiatry. 2021;12:738810. doi: 10.3389/fpsyt.2021.738810. PMID:34992553; PMCID: PMC8724305.	No drug
1253	Samochowiec J, Szulc A, Bieńkowski P, Dudek D, Gałecki P,Heitzman J, Wojnar M, Wichniak A. Polish Psychiatric Association consensus statement on non-pharmacological methods in the treatment of negative symptoms of schizophrenia. Psychiatr Pol.2021;55(4):719-742. English, Polish. doi:10.12740/PP/OnlineFirst/135527. Epub 2021 Aug 31. PMID:34994733.	Review
1254	Wójciak P, Domowicz K, Zabłocka M, Michalak M, Rybakowski JK. Association of negative symptoms of schizophrenia assessed by the BNSS and SNS scales with neuropsychological performance: A gender effect. Front Psychiatry. 2021;12:797386. doi: 10.3389/fpsyt.2021.797386. PMID: 35002812; PMCID:PMC8738094.	No drug
1255	Priyamvada R, Ranjan R, Jha GK, Chaudhury S. Correlation of neurocognitive deficits with positive and negative symptoms in schizophrenia. Ind Psychiatry J. 2021;30(2):249-254. doi:10.4103/ipj.ipj_44_20. Epub 2021 Sep 8. PMID: 35017808;PMCID: PMC8709519.	No drug
1256	Bhat PS, Raj J, Chatterjee K, Srivastava K. Cognitive dysfunction in first-episode schizophrenia and its correlation with negative symptoms and insight. Ind Psychiatry J. 2021;30(2):310-315. doi:10.4103/ipj.ipj_107_20. Epub 2021 Oct 28. PMID: 35017817;PMCID: PMC8709515.	No drug
1257	Ahmed AO, Kirkpatrick B, Granholm E, Rowland LM, Barker PB,Gold JM, Buchanan RW, Outram T, Bernardo M, García-Portilla MP, Mane A, Fernandez-Egea E, Strauss GP. Two factors, fivefactors, or both? External validation studies of negative symptom dimensions in schizophrenia. Schizophr Bull. 2022;48(3):620-630.doi: 10.1093/schbul/sbab148. PMID: 35020936; PMCID:PMC9077418.	No drug
1258	Lezheiko TV, Kolesina NY, Golimbet VE. Dataset on negative symptoms factors in patients with schizophrenia. Data Brief. 2022;40:107790. doi: 10.1016/j.dib.2022.107790. PMID: 35036488;PMCID: PMC8749170.	No drug
1259	Li M, Qiu Y, Zhang J, Zhang Y, Liu Y, Zhao Y, Jia Q, Fan X, Li J. Improvement of adjunctive berberine treatment on negativesymptoms in patients with schizophrenia. Eur Arch Psychiatry Clin Neurosci. 2022;272(4):633-642. doi: 10.1007/s00406-021-01359-4.Epub 2022 Jan 17. PMID: 35037116.	**Included**
1260	Mosolov SN, Yaltonskaya PA. Primary and secondary negative symptoms in schizophrenia. Front Psychiatry. 2022;12:766692. doi:10.3389/fpsyt.2021.766692. PMID: 35046851; PMCID:PMC8761803.	No drug
1261	van der Meer L, Kaiser S, Castelein S. Negative symptoms in schizophrenia: reconsidering evidence and focus in clinical trials. Br J Psychiatry. 2021;219(1):359-360. doi: 10.1192/bjp.2021.66.PMID: 35048858.	No drug
1262	Pelizza L, Leuci E, Maestri D, Quattrone E, Azzali S, Paulillo G, Pellegrini P. Negative symptoms in first episode schizophrenia: treatment response across the 2-year follow-up of the "Parma Early Psychosis" program. Eur Arch Psychiatry Clin Neurosci.2022;272(4):621-632. doi: 10.1007/s00406-021-01374-5. Epub2022 Jan 28. PMID: 35088121.	No drug
1263	Hebel T, Langguth B, Schecklmann M, Schoisswohl S, StaudingerS, Schiller A, Ustohal L, Sverak T, Horky M, Kasparek T, Skront T,Hyza M, Poeppl TB, Riester ML, Schwemmer L, Zimmermann S,Sakreida K. Rationale and study design of a trial to assess rTMSadd-on value for the amelioration of negative symptoms of schizophrenia (RADOVAN). Contemp Clin Trials Commun. 2022;26:100891. doi: 10.1016/j.conctc.2022.100891. PMID:35128142; PMCID: PMC8804178.	Protocol

1264	Ivanov SV, Smulevich AB, Voronova EI, Yakhin KK, Beybalaeva TZ, Katok AA. Early clinical effects of novel partial D3/D2 agonist cariprazine in schizophrenia patients with predominantly negative symptoms (open-label, non-controlled study). FrontPsychiatry. 2022;12:770592. doi: 10.3389/fpsyt.2021.770592.PMID: 35140638; PMCID: PMC8818881.	Open
1265	Weber S, Scott JG, Chatterton ML. Healthcare costs and resource use associated with negative symptoms of schizophrenia: A systematic literature review. Schizophr Res. 2022;241:251-259. doi:10.1016/j.schres.2022.01.051. Epub 2022 Feb 15. PMID: 35180664.	Review
1266	Bitter I, Mohr P, Raspopova N, Szulc A, Samochowiec J, MicluiaIV, Skugarevsky O, Herold R, Mihaljevic-Peles A, Okribelashvili N, Dragašek J, Adomaitiene V, Rancans E, Chihai J, Maruta N, Marić NP, Milanova V, Tavčar R, Mosolov S. Assessment and treatment of negative symptoms in schizophrenia-A regional perspective. Front Psychiatry. 2022;12:820801. doi: 10.3389/fpsyt.2021.820801.PMID: 35185643; PMCID: PMC8855151.	No drug
1267	Demjaha A, Galderisi S, Glenthøj B, Arango C, Mucci A, LawrenceA, O'Daly O, Kempton M, Ciufolini S, Baandrup L, Ebdrup BH, Rodriguez-Jimenez R, Diaz-Marsa M, Díaz-Caneja CM, Winter van Rossum I, Kahn R, Dazzan P, McGuire P. Negative symptoms in First-Episode Schizophrenia related to morphometric alterations in orbitofrontal and superior temporal cortex: the OPTiMiSE study. Psychol Med. 2023;53(8):3471-3479. doi:10.1017/S0033291722000010. Epub 2022 Feb 24. PMID:35197142; PMCID: PMC10277764.	No drug
1268	García-Fernández L, Romero-Ferreiro V, Sánchez-Pastor L, Dompablo M, Martínez-Gras I, Espejo-Saavedra JM, Rentero D, Aparicio AI, Alvarez-Mon MA, Lahera G, Lee J, Santos JL, Rodriguez-Jimenez R. Impact of negative symptoms on functioning and quality of life in first psychotic episodes of schizophrenia. J Clin Med. 2022;11(4):983. doi:10.3390/jcm11040983. PMID: 35207256; PMCID: PMC8879613.	No drug
1269	Davidson M, Saoud J, Staner C, Noel N, Werner S, Luthringer E,Walling D, Weiser M, Harvey PD, Strauss GP, Luthringer R. Efficacy and safety of roluperidone for the treatment of negative symptoms of schizophrenia. Schizophr Bull. 2022;48(3):609-619. doi: 10.1093/schbul/sbac013. PMID: 35211743; PMCID:PMC9077422.	Non-marketed
1270	Wong SMY, Suen YN, Wong CWC, Chan SKW, Hui CLM, ChangWC, Lee EHM, Cheng CPW, Ho GCL, Lo GG, Leung EYL, Yeung PKMA, Chen S, Honer WG, Mak HKF, Sham PC, McKenna PJ, Pomarol-Clotet E, Veronese M, Howes OD, Chen EYH. Striatal dopamine synthesis capacity and its association with negative symptoms upon resolution of positive symptoms in first-episode schizophrenia and delusional disorder. Psychopharmacology (Berl). 2022;239(7):2133-2141. doi: 10.1007/s00213-022-06088-7. Epub2022 Feb 25. PMID: 35211769.	No drug
1271	Phahladira L, Asmal L, Lückhoff HK, du Plessis S, Scheffler F, SmitR, Chiliza B, Emsley R. The trajectories and correlates of two negative symptom subdomains in first-episode schizophrenia. Schizophr Res. 2022;243:17-23. doi: 10.1016/j.schres.2022.02.017.Epub 2022 Feb 25. PMID: 35228035.	No drug
1272	Borovcanin MM, Janicijevic SM, Mijailovic NR, Jovanovic IP, Arsenijevic NN, Vesic K. Uric acid potential role in systemic inflammation and negative symptoms after acute antipsychotic treatment in schizophrenia. Front Psychiatry. 2022;12:822579. doi:10.3389/fpsyt.2021.822579. PMID: 35237183; PMCID:PMC8882684.	Unfocused
1273	García-Álvarez L, Martínez-Cao C, Bobes-Bascarán T, Portilla A,Courtet P, de la Fuente-Tomás L, Velasco Á, González-Blanco L,Zurrón-Madera P, Fonseca- Pedrero E, Sáiz PA, García-Portilla MP, Bobes J. Validation of a European Spanish-version of the Self-Evaluation of Negative Symptoms (SNS) in patients with schizophrenia. Rev Psiquiatr Salud Ment (Engl Ed). 2022;15(1):14-21. doi: 10.1016/j.rpsmen.2022.01.005. PMID: 35256068.	No drug
1274	Rekhi G, Ang MS, Chan YH, Fernandez-Egea E, Kirkpatrick B, Lee J. Defining negative symptoms remission in schizophrenia using the Brief Negative Symptom Scale. Rev Psiquiatr Salud Ment (EnglEd). 2022;15(1):3-13. doi: 10.1016/j.rpsmen.2022.01.007. PMID:35256070.	No drug
1275	Du X, Fan HZ, Wang YH, Zhang J, Zhu XL, Zhao YL, Tan SP. Characteristics of facial muscle activity intensity in patients with schizophrenia and its relationship to negative symptoms. Front Psychiatry. 2022;13:829363. doi: 10.3389/fpsyt.2022.829363.PMID: 35264989; PMCID: PMC8900141.	No drug
1276	Pultsina KI, Alekhin AN, Petrova EV, Vorobieva NV. Effektivnost'setei vnimaniya i vyrazhennost' pozitivnoi i negativnoi simptomatikipri shizofrenii [Efficiency of the attention networks and severity of positive and negative symptoms in schizophrenia]. Zh Nevrol Psikhiatr Im S S Korsakova. 2022;122(2):88-96. Russian. doi:10.17116/jnevro202212202188. PMID: 35271242.	No drug
1277	Nestor PG, Levitt JJ, Ohtani T, Newell DT, Shenton ME, Niznikiewicz M. Loosening of associations in chronic schizophrenia: intersectionality of verbal learning, negative symptoms, and brain structure. Schizophr Bull Open.2022;3(1):sgac004. doi: 10.1093/schizbullopen/sgac004. PMID:35295655; PMCID: PMC8918213.	No drug
1278	Hayashi R, Kuroda K, Inadomi H. Jumping to conclusions correlates with negative symptoms, poor response inhibition, and impaired functioning in individuals diagnosed with schizophrenia. Asian J Psychiatr. 2022;71:103068. doi: 10.1016/j.ajp.2022.103068. Epub2022 Mar 12. PMID: 35311670.	No drug
1279	Macfie WG, Spilka MJ, Bartolomeo LA, Gonzalez CM, Strauss GP. Emotion regulation and social knowledge in youth at clinical high-risk for psychosis and outpatients with chronic schizophrenia: Associations with functional outcome and negative symptoms. Early Interv Psychiatry. 2023;17(1):21-28. doi: 10.1111/eip.13287. Epub2022 Apr 1. PMID: 35362242; PMCID: PMC10084209.	No drug
1280	Ping J, Zhang J, Wan J, Huang C, Luo J, Du B, Jiang T. A polymorphism in the BDNF gene (rs11030101) is associated with negative symptoms in Chinese Han patients with schizophrenia. Front Genet. 2022;13:849227. doi: 10.3389/fgene.2022.849227.PMID: 35368680; PMCID: PMC8974295.	No drug
1281	Jaiswal N, Kar SK, Gupta PK. Comments on "High frequencyrepetitive transcranial magnetic stimulation of dorsomedialprefrontal cortex for negative symptoms in patients withschizophrenia: A double-blind, randomized controlled trial".Psychiatry Res. 2022;312:114532. doi: 10.1016/j.psychres.2022.114532. Epub 2022 Mar 30. PMID:35417823.	No drug
1282	Russo M, Repisti S, Blazhevska Stoilkovska B, Jerotic S, Ristic I, Mesevic Smajic E, Uka F, Arenliu A, Bajraktarov S, Dzubur Kulenovic A, Injac Stevovic L, Priebe S, Jovanovic N. Corrigendum: Structure of negative symptoms in schizophrenia: An unresolved issue. Front Psychiatry. 2022;13:885883. doi: 10.3389/fpsyt.2022.885883. Erratum for: Front Psychiatry. 2021;12:785144. PMID: 35418890; PMCID: PMC8997331.	Duplicate= 1250
1283	Laraki Y, Lebrun C, Merenciano M, Eisenblaetter M, Attal J, Macgregor A, Decombe A, Capdevielle D, Raffard S. Validation of the French Clinical Assessment Interview for Negative Symptoms in a sample of stable French individuals with schizophrenia. Front Psychiatry. 2022;13:836600. doi: 10.3389/fpsyt.2022.836600. PMID: 35432043; PMCID: PMC9010618.	No drug
1284	Charernboon T. Interplay among positive and negative symptoms, neurocognition, social cognition, and functioning in clinically stable patients with schizophrenia: a network analysis. F1000Res. 2021;10:1258. doi: 10.12688/f1000research.74385.3. PMID: 35464178; PMCID: PMC9021676.	No drug
1285	Perera P, Gajaram G, Qureshi D, Gill M, Thanju A, Zaman A, Fouron P, Jolayemi A. Use of bupropion in the management of negative symptom schizophrenia: A case series. Cureus. 2022;14(3):e23518. doi: 10.7759/cureus.23518. PMID: 35494898; PMCID: PMC9038077.	Case
1286	Mahmood Z, Parrish EM, Keller AV, Lykins HC, Pickell D, Granholm E, Twamley EW. Modifiable predictors of self-reported and performance-based functioning in individuals with schizophrenia-spectrum disorders and high levels of negative symptoms. J Psychiatr Res. 2022;151:347-353. doi: 10.1016/j.jpsychires.2022.04.039. Epub 2022 May 2. PMID: 35533518.	No drug
1287	Czobor P, Sebe B, Acsai K, Barabássy Á, Laszlovszky I, Németh G, Furukawa TA, Leucht S. What is the minimum clinically important change in negative symptoms of schizophrenia? PANSS based post-hoc analyses of a Phase III clinical trial. Front Psychiatry. 2022;13:816339. doi: 10.3389/fpsyt.2022.816339. PMID: 35546918; PMCID: PMC9083222.	No drug
1288	Demyttenaere K, Anthonis E, Acsai K, Correll CU. Depressive Symptoms and PANSS symptom dimensions in patients with predominant negative symptom schizophrenia: A network analysis. Front Psychiatry. 2022;13:795866. doi: 10.3389/fpsyt.2022.795866. PMID: 35546936; PMCID: PMC9081724.	No drug

1289	Carrà G, Crocamo C, Bartoli F, Angermeyer M, Brugha T, Toumi M, Bebbington P. Influence of positive and negative symptoms on hedonic and eudaemonic well-being in people with schizophrenia: A longitudinal analysis from the EuroSc study. Schizophr Res. 2022;244:21-28. doi: 10.1016/j.schres.2022.04.009. Epub 2022 May 11. PMID: 35567870.	No drug
1290	Zierhut M, Böge K, Bergmann N, Hahne I, Braun A, Kraft J, Ta TMT, Ripke S, Bajbouj M, Hahn E. The relationship between the recognition of basic emotions and negative symptoms in individuals with schizophrenia spectrum disorders - An exploratory study. Front Psychiatry. 2022;13:865226. doi: 10.3389/fpsyt.2022.865226. PMID: 35573376; PMCID: PMC9091587.	No drug
1291	Sverak T, Mayerova M, Obdrzalkova M, Ustohal L. Accelerated repetitive transcranial magnetic stimulation in the treatment of negative symptoms of schizophrenia: An open-label study. J ECT. 2022;38(2):e24-e25. doi: 10.1097/YCT.0000000000000826. PMID: 35613013.	No drug
1292	Tan X, Martin D, Lee J, Tor PC. The impact of electroconvulsive therapy on negative symptoms in schizophrenia and their association with clinical outcomes. Brain Sci. 2022;12(5):545. doi: 10.3390/brainsci12050545. PMID: 35624932; PMCID: PMC9139352.	No drug
1293	Misir E, Ozbek MM, Halac E, Turan S, Alkas GE, Ciray RO, Ermis C. The effects of catechol-O-methyltransferase single nucleotide polymorphisms on positive and negative symptoms of schizophrenia: A systematic review and meta- analysis. Psych J. 2022;11(6):779-791. doi: 10.1002/pchj.562. Epub 2022 May 31. PMID: 35642295.	Review
1294	Bayrakçı A, Zorlu N, Karakılıç M, Gülyüksel F, Yalınçetin B, Oral E, Gelal F, Bora E. Negative symptoms are associated with modularity and thalamic connectivity in schizophrenia. Eur Arch Psychiatry Clin Neurosci. 2023;273(3):565-574. doi: 10.1007/s00406-022-01433-5. Epub 2022 Jun 3. PMID: 35661912.	No drug
1295	Meyer-Lindenberg A, Nielsen J, Such P, Lemming OM, Zambori J, Buller R, der Goltz CV. A double-blind, randomized, placebo-controlled proof of concept study of the efficacy and safety of Lu AF11167 for persistent negative symptoms in people with schizophrenia. Eur Neuropsychopharmacol. 2022;61:4-14. doi: 10.1016/j.euroneuro.2022.05.009. Epub 2022 Jun 12. PMID: 35704951.	Non-marketed
1296	Czobor P, Kakuszi B, Bitter I. Placebo response in trials of negative symptoms in schizophrenia: A critical reassessment of the evidence. Schizophr Bull. 2022;48(6):1228-1240. doi: 10.1093/schbul/sbac061. PMID: 35713342; PMCID: PMC9673255.	Review
1297	Tseng PT, Zeng BS, Hung CM, Liang CS, Stubbs B, Carvalho AF, Brunoni AR, Su KP, Tu YK, Wu YC, Chen TY, Li DJ, Lin PY, Hsu CW, Chen YW, Suen MW, Satogami K, Takahashi S, Wu CK, Yang WC, Shiue YL, Huang TL, Li CT. Assessment of noninvasive brain stimulation interventions for negative symptoms of schizophrenia: A systematic review and network meta-analysis. JAMA Psychiatry. 2022;79(8):770-779. doi: 10.1001/jamapsychiatry.2022.1513. PMID: 35731533; PMCID: PMC9218931.	No drug
1298	Arnovitz MD, Spitzberg AJ, Davani AJ, Vadhan NP, Holland J, Kane JM, Michaels TI. MDMA for the treatment of negative symptoms in schizophrenia. J Clin Med. 2022;11(12):3255. doi: 10.3390/jcm11123255. PMID: 35743326; PMCID: PMC9225098.	Review
1299	Giordano GM, Caporusso E, Pezzella P, Galderisi S. Updated perspectives on the clinical significance of negative symptoms in patients with schizophrenia. Expert Rev Neurother. 2022;22(7):541-555. doi: 10.1080/14737175.2022.2092402. Epub 2022 Jun 27. PMID: 35758871.	Review
1300	Becarevic N, Softic R, Osmanovic E. Does the duration of the illness affect the severity of negative symptoms of schizophrenia? Mater Sociomed. 2022;34(1):25-27. doi: 10.5455/msm.2022.33.25-27. PMID: 35801060; PMCID: PMC9229283.	No drug
1301	Lorentzen R, Nguyen TD, McGirr A, Hieronymus F, Østergaard SD. The efficacy of transcranial magnetic stimulation (TMS) for negative symptoms in schizophrenia: a systematic review and meta-analysis. Schizophrenia (Heidelb). 2022;8(1):35. doi: 10.1038/s41537-022-00248-6. PMID: 35853882; PMCID: PMC9261093.	Review
1302	Lee J, Oh JS, Park CI, Bang M, Sung G, Jung S, Lee SH. White matter microstructure of superior longitudinal fasciculus II is associated with intelligence and treatment response of negative symptoms in patients with schizophrenia. Schizophrenia (Heidelb). 2022;8(1):43. doi: 10.1038/s41537-022-00253-9. PMID: 35853887; PMCID: PMC9262917.	No drug
1303	Strauss GP, Raugh IM, Zhang L, Luther L, Chapman HC, Allen DN, Kirkpatrick B, Cohen AS. Validation of accelerometry as a digital phenotyping measure of negative symptoms in schizophrenia. Schizophrenia (Heidelb). 2022;8(1):37. doi: 10.1038/s41537-022-00241-z. PMID: 35853890; PMCID: PMC9261099.	No drug
1304	Deng M, Liu Z, Zhang W, Wu Z, Cao H, Yang J, Palaniyappan L. Associations between polygenic risk, negative symptoms, and functional connectome topology during a working memory task in early-onset schizophrenia. Schizophrenia (Heidelb). 2022;8(1):54. doi: 10.1038/s41537-022-00260-w. PMID: 35853905; PMCID: PMC9261080.	No drug
1305	Neill E, Rossell SL, Yolland C, Meyer D, Galletly C, Harris A, Siskind D, Berk M, Bozaoglu K, Dark F, Dean OM, Francis PS, Liu D, Phillipou A, Sarris J, Castle DJ. N-Acetylcysteine (NAC) in schizophrenia resistant to clozapine: A double-blind, randomized, placebo-controlled trial targeting negative symptoms. Schizophr Bull. 2022;48(6):1263-1272. doi: 10.1093/schbul/sbac065. PMID: 35857752; PMCID: PMC9673271.	**Included**
1306	Okada H. Association of negative symptom domains and other clinical characteristics of schizophrenia on long-term hospitalization. Indian J Psychiatry. 2022;64(3):277-283. doi: 10.4103/indianjpsychiatry.indianjpsychiatry_134_21. Epub 2022 Jun 8. PMID: 35859563; PMCID: PMC9290414.	No drug
1307	Brandl F, Knolle F, Avram M, Leucht C, Yakushev I, Priller J, Leucht S, Ziegler S, Wunderlich K, Sorg C. Negative symptoms, striatal dopamine and model- free reward decision-making in schizophrenia. Brain. 2023;146(2):767-777. doi: 10.1093/brain/awac268. PMID: 35875972.	No drug
1308	Tran T, Spilka MJ, Ruiz I, Strauss GP. Implicit cognitive effort monitoring impairments are associated with expressive negative symptoms in schizophrenia. Schizophr Res. 2022;248:14-20. doi: 10.1016/j.schres.2022.07.006. Epub 2022 Jul 27. PMID: 35907347.	No drug
1309	Salehi A, Namaei P, Taghavi Zanjani F, Bagheri S, Moradi K, Khodaei Ardakani MR, Akhondzadeh S. Adjuvant palmitoylethanolamide therapy with risperidone improves negative symptoms in patients with schizophrenia: A randomized, double-blinded, placebo-controlled trial. Psychiatry Res. 2022;316:114737. doi: 10.1016/j.psychres.2022.114737. Epub 2022 Jul 27. PMID: 35917650.	**Included**
1310	Maurus I, Röll L, Keeser D, Karali T, Papazov B, Hasan A, Schmitt A, Papazova I, Lembeck M, Hirjak D, Thieme CE, Sykorova E, Münz S, Seitz V, Greska D, Campana M, Wagner E, Löhrs L, Pömsl J, Roeh A, Malchow B, Keller-Varady K, Ertl-Wagner B, Stöcklein S, Meyer-Lindenberg A, Falkai P. Associations between aerobic fitness, negative symptoms, cognitive deficits and brain structure in schizophrenia-a cross-sectional study. Schizophrenia (Heidelb). 2022;8(1):63. doi: 10.1038/s41537-022-00269-1. PMID: 35918344; PMCID: PMC9345912.	No drug
1311	Cella M, Tomlin P, Robotham D, Green P, Griffiths H, Stahl D, Valmaggia L. Virtual Reality Therapy for the Negative Symptoms of Schizophrenia (V-NeST): A pilot randomised feasibility trial. Schizophr Res. 2022;248:50-57. doi: 10.1016/j.schres.2022.07.013. Epub 2022 Aug 5. PMID: 35939920.	No drug
1312	Wang LL, Tam MHW, Ho KKY, Hung KSY, Wong JOY, Lui SSY, Chan RCK. Bridge centrality network structure of negative symptoms in people with schizophrenia. Eur Arch Psychiatry Clin Neurosci. 2023;273(3):589-600. doi: 10.1007/s00406-022-01474-w. Epub 2022 Aug 16. PMID: 35972557.	No drug
1313	Abplanalp SJ, Braff DL, Light GA, Nuechterlein KH, Green MF; Consortium on the Genetics of Schizophrenia-2. Understanding connections and boundaries between positive symptoms, negative symptoms, and role functioning among individuals with schizophrenia: A network psychometric approach. JAMA Psychiatry. 2022;79(10):1014-1022. doi: 10.1001/jamapsychiatry.2022.2386. PMID: 35976655; PMCID: PMC9386606.	No drug
1314	Okruszek Ł, Chrustowicz M, Jarkiewicz M, Krawczyk M, Manera V, Piejka A, Schudy A, Wiśniewska M, Wysokiński A. Mentalizing abilities mediate the impact of the basic social perception on negative symptoms in patients with schizophrenia. J Psychiatr Res. 2022;155:85-89. doi: 10.1016/j.jpsychires.2022.07.069. Epub 2022 Aug 4. PMID: 35995018.	No drug
1315	Li SB, Liu C, Zhang JB, Wang LL, Hu HX, Chu MY, Wang Y, Lv QY, Lui SSY, Cheung EFC, Yi ZH, Chan RCK. Revisiting the latent structure of negative symptoms in schizophrenia: Evidence from two second-generation clinical assessments. Schizophr Res. 2022;248:131-139. doi: 10.1016/j.schres.2022.08.016. Epub 2022 Aug 29. Erratum in: Schizophr Res. 2023;252:206-207. PMID: 36037646.	No drug

1316	Ince Guliyev E, Guloksuz S, Ucok A. Impaired effort allocation in patients with recent-onset schizophrenia and its relevance to negative symptoms assessments and persistent negative symptoms. J Clin Med. 2022;11(17):5060. doi: 10.3390/jcm11175060. PMID: 36078990; PMCID: PMC9457458.	No drug
1317	Fattal J, Brascamp JW, Slate RE, Lehet M, Achtyes ED, Thakkar KN. Blunted pupil light reflex is associated with negative symptoms and working memory in individuals with schizophrenia. Schizophr Res. 2022;248:254-262. doi: 10.1016/j.schres.2022.09.019. Epub 2022 Sep 14. PMID: 36115190; PMCID: PMC9613610.	No drug
1318	James SH, Berglund A, Chang WC, Strauss GP. Discrepancies between ideal and actual affect in schizophrenia: Implications for understanding negative symptoms. J Psychiatr Res. 2022;155:313-319. doi: 10.1016/j.jpsychires.2022.09.024. Epub 2022 Sep 21. PMID: 36174366.	No drug
1319	Lisoni J, Baldacci G, Nibbio G, Zucchetti A, Butti Lemmi Gigli E, Savorelli A, Facchi M, Miotto P, Deste G, Barlati S, Vita A. Effects of bilateral, bipolar-nonbalanced, frontal transcranial Direct Current Stimulation (tDCS) on negative symptoms and neurocognition in a sample of patients living with schizophrenia: Results of a randomized double-blind sham-controlled trial. J Psychiatr Res. 2022;155:430-442. doi: 10.1016/j.jpsychires.2022.09.011. Epub 2022 Sep 21. PMID: 36182772.	No drug
1320	Bruhn D, Hwang S, Howarth A, Dubé S. The burden of illness for patients with schizophrenia and primary negative symptoms: A systematic literature review. Schizophr Res. 2022;248:341-344. doi: 10.1016/j.schres.2022.09.017. Epub 2022 Oct 3. PMID: 36202050.	Review
1321	Herwerth L, Prinz K, Brauner H, Müller K, Fleischhaker C. Psychosen aus dem schizophrenen Formenkreis im Jugendalter: Einflussfaktoren auf Neuropsychologie, Behandlungserfolg und Negativsymptomatik [Psychoses on the schizophrenia spectrum in adolescence: A cross-sectional study of factors influencing neuropsychology, treatment outcome, and negative symptoms]. Z Kinder Jugendpsychiatr Psychother. 2023;51(3):196-206. German. doi: 10.1024/1422-4917/a000903. Epub 2022 Oct 7. PMID: 36205021.	No drug
1322	Huang Z, Ruan D, Huang B, Zhou T, Shi C, Yu X, Chan RCK, Wang Y, Pu C. Negative symptoms correlate with altered brain structural asymmetry in amygdala and superior temporal region in schizophrenia patients. Front Psychiatry. 2022;13:1000560. doi: 10.3389/fpsyt.2022.1000560. PMID: 36226098; PMCID: PMC9548644.	No drug
1323	Zhu T, Wang Z, Zhou C, Fang X, Huang C, Xie C, Ge H, Yan Z, Zhang X, Chen J. Meta-analysis of structural and functional brain abnormalities in schizophrenia with persistent negative symptoms using activation likelihood estimation. Front Psychiatry. 2022;13:957685. doi: 10.3389/fpsyt.2022.957685. PMID: 36238945; PMCID: PMC9552970.	Review
1324	Barlati S, Nibbio G, Calzavara-Pinton I, Invernizzi E, Cadei L, Lisoni J, Valsecchi P, Deste G, Vita A. Primary and secondary negative symptoms severity and the use of psychiatric care resources in schizophrenia spectrum disorders: A 3-year follow-up longitudinal retrospective study. Schizophr Res. 2022;250:31-38. doi: 10.1016/j.schres.2022.10.002. Epub 2022 Oct 14. PMID: 36252294.	No drug
1325	Correction to: Predictors of placebo response in pharmacological clinical trials of negative symptoms in schizophrenia: A meta-regression analysis. Schizophr Bull. 2023;49(2):534-537. doi: 10.1093/schbul/sbac172. Erratum for: Schizophr Bull. 2019;45(1):57-68. PMID: 36315443; PMCID: PMC10016394.	Duplicate= 994
1326	Abdallah MS, Mosalam EM, Hassan A, Ramadan AN, Omara-Reda H, Zidan AA, Samman WA, El-Berri EI. Pentoxifylline as an adjunctive in treatment of negative symptoms in chronic schizophrenia: A double-blind, randomized, placebo- controlled trial. CNS Neurosci Ther. 2023;29(1):354-364. doi: 10.1111/cns.14010. Epub 2022 Nov 7. Retraction in: CNS Neurosci Ther. 2024;30(3):e14668. PMID: 36341700; PMCID: PMC9804082.	Retracted/ Removed
1327	Moya NA, Yun S, Fleps SW, Martin MM, Nadel JA, Beutler LR, Zweifel LS, Parker JG. The effect of selective nigrostriatal dopamine excess on behaviors linked to the cognitive and negative symptoms of schizophrenia. Neuropsychopharmacology. 2023;48(4):690-699. doi: 10.1038/s41386-022-01492-1. Epub 2022 Nov 15. PMID: 36380221; PMCID: PMC9938164.	Animal
1328	Maroney M. Management of cognitive and negative symptoms in schizophrenia. Ment Health Clin. 2022;12(5):282-299. doi: 10.9740/mhc.2022.10.282. PMID: 36405508; PMCID: PMC9645289.	Case
1329	Zhao Q, Wang WQ, Fan HZ, Li D, Li YJ, Zhao YL, Tian ZX, Wang ZR, Tan YL, Tan SP. Vocal acoustic features may be objective biomarkers of negative symptoms in schizophrenia: A cross-sectional study. Schizophr Res. 2022;250:180-185. doi: 10.1016/j.schres.2022.11.013. Epub 2022 Nov 21. PMID: 36423443.	No drug
1330	Petrescu C, Papacocea IR, Vilciu C, Mihalache OA, Vlad DM, Marian G, Focseneanu BE, Sima CT, Ciobanu CA, Riga S, Ciobanu AM. The impact of antipsychotic treatment on neurological soft signs in patients with predominantly negative symptoms of schizophrenia. Biomedicines. 2022;10(11):2939. doi: 10.3390/biomedicines10112939. PMID: 36428507; PMCID: PMC9687986.	Lumping
1331	Ahmadkhaniha H, Ayazi N, Alavi K, Najjarzadehgan M, Hadi F. The comparison between positive and negative symptoms severity in prolonged methamphetamine-induced psychotic disorder and schizophrenia. Basic Clin Neurosci. 2022;13(3):325-333. doi: 10.32598/bcn.2021.2837.1. Epub 2022 May 1. PMID: 36457876; PMCID: PMC9706289.	No drug
1332	Golimbet V, Lezheiko T, Mikhailova V, Korovaitseva G, Kolesina N, Plakunova V, Kostyuk G. A study of the association between polymorphisms in the genes for interleukins IL-6 and IL-10 and negative symptoms subdomains in schizophrenia. Indian J Psychiatry. 2022;64(5):484-488. doi: 10.4103/indianjpsychiatry.indianjpsychiatry_212_22. Epub 2022 Oct 12. PMID: 36458089; PMCID: PMC9707661.	No drug
1333	Sabe M, Chen C, Perez N, Solmi M, Mucci A, Galderisi S, Strauss GP, Kaiser S. Thirty years of research on negative symptoms of schizophrenia: A scientometric analysis of hotspots, bursts, and research trends. Neurosci Biobehav Rev. 2023;144:104979. doi: 10.1016/j.neubiorev.2022.104979. Epub 2022 Dec 1. PMID: 36463972.	No drug
1334	Kim S, Kim SW, Bui MAT, Kim Y, Kim M, Park JC, Kim NH, Pyeon GH, Jo YS, Jang J, Koh HY, Jeong CH, Kang M, Kang HJ, Lee YW, Stockmeier CA, Seong JK, Woo DH, Han JS, Kim YS. SELENBP1 overexpression in the prefrontal cortex underlies negative symptoms of schizophrenia. Proc Natl Acad Sci U S A. 2022;119(51):e2203711119. doi: 10.1073/pnas.2203711119. Epub 2022 Dec 13. PMID: 36512497; PMCID: PMC9907074.	*Post-mortem*
1335	Gao T, Huang Z, Huang B, Zhou T, Shi C, Yu X, Pu C. Negative symptom dimensions and social functioning in Chinese patients with schizophrenia. Front Psychiatry. 2022;13:1033166. doi: 10.3389/fpsyt.2022.1033166. PMID: 36561640; PMCID: PMC9763280.	No drug
1336	Jin Y, Tong J, Huang Y, Shi D, Zhu N, Zhu M, Liu M, Liu H, Sun X. Effectiveness of accelerated intermittent theta burst stimulation for social cognition and negative symptoms among individuals with schizophrenia: A randomized controlled trial. Psychiatry Res. 2023;320:115033. doi: 10.1016/j.psychres.2022.115033. Epub 2022 Dec 25. PMID: 36603383.	No drug
1337	Galderisi S. Negative symptoms of schizophrenia: Trying to answer unanswered research questions. Psychiatry Res. 2023;320:115043. doi: 10.1016/j.psychres.2022.115043. Epub 2022 Dec 28. PMID: 36623425.	Opinion
1338	Paul NB, Strauss GP, Gates-Woodyatt JJ, Barchard KA, Allen DN. Two and five-factor models of negative symptoms in schizophrenia are differentially associated with trait affect, defeatist performance beliefs, and psychosocial functioning. Eur Arch Psychiatry Clin Neurosci. 2023;273(8):1715-1724. doi: 10.1007/s00406-022-01507-4. Epub 2023 Jan 12. PMID: 36633673.	No drug
1339	Oh J, Lee E, Cha EJ, Seo HJ, Choi KH. Community-based multi-site randomized controlled trial of behavioral activation for patients with negative symptoms of schizophrenia. Schizophr Res. 2023;252:118-126. doi: 10.1016/j.schres.2022.12.051. Epub 2023 Jan 12. PMID: 36640745.	No drug
1340	Chan SKW, Liao Y, Hui CLM, Wong TY, Suen Y, Chang WC, Lee EHM, Chen EYH. Longitudinal changes of cognitive function and its relationship with subdomains of negative symptoms in patients with adult-onset first-episode schizophrenia: A 4-year follow up study. Schizophr Res. 2023;252:181-188. doi: 10.1016/j.schres.2023.01.004. Epub 2023 Jan 17. PMID: 36657362.	No drug
1341	Li SB, Liu C, Zhang JB, Wang LL, Hu HX, Chu MY, Wang Y, Lv QY, Lui SSY, Cheung EFC, Yi ZH, Chan RCK. Corrigendum to "Revisiting the latent structure of negative symptoms in schizophrenia: Evidence from two second-generation clinical assessments" [Schizophr. Res. 248 (2022) 131-139]. Schizophr Res. 2023;252:206-207. doi: 10.1016/j.schres.2022.12.024. Epub 2023 Jan 17. Erratum for: Schizophr Res. 2022;248:131-139. PMID: 36657365.	Duplicate= 1315

1342	Lui SSY, Wang LL, Lau WYS, Shing E, Yeung HKH, Tsang KCM, Zhan EN, Cheung ESL, Ho KKY, Hung KSY, Cheung EFC, Chan RCK. Emotion-behaviour decoupling and experiential pleasure deficits predict negative symptoms and functional outcome in first-episode schizophrenia patients. Asian J Psychiatr. 2023;81:103467. doi: 10.1016/j.ajp.2023.103467. Epub 2023 Jan 16. PMID: 36669292.	No drug
1343	Giordano GM, Pezzella P, Giuliani L, Fazio L, Mucci A, Perrottelli A, Blasi G, Amore M, Rocca P, Rossi A, Bertolino A, Galderisi S, Italian Network For Research On Psychoses. Resting-state brain activity dysfunctions in schizophrenia and their associations with negative symptom domains: An fMRI study. Brain Sci. 2023;13(1):83. doi: 10.3390/brainsci13010083. PMID: 36672064; PMCID: PMC9856573.	No drug
1344	Cheon EJ, Male AG, Gao B, Adhikari BM, Edmond JT, Hare SM, Belger A, Potkin SG, Bustillo JR, Mathalon DH, Ford JM, Lim KO, Mueller BA, Preda A, O'Leary D, Strauss GP, Ahmed AO, Thompson PM, Jahanshad N, Kochunov P, Calhoun VD, Turner JA, van Erp TGM. Five negative symptom domains are differentially associated with resting state amplitude of low frequency fluctuations in Schizophrenia. Psychiatry Res Neuroimaging. 2023;329:111597. doi: 10.1016/j.pscychresns.2023.111597. Epub 2023 Jan 16. PMID: 36680843.	No drug
1345	Montvidas J, Adomaitienė V, Leskauskas D, Dollfus S. Correlation of health-related quality of life with negative symptoms assessed with the self- evaluation of Negative Symptoms Scale (SNS) and cognitive deficits in schizophrenia: A cross-sectional study in routine psychiatric care. J Clin Med. 2023;12(3):901. doi: 10.3390/jcm12030901. PMID: 36769548; PMCID: PMC9917914.	No drug
1346	Romeo B, Willaime L, Rari E, Benyamina A, Martelli C. Efficacy of 5-HT2A antagonists on negative symptoms in patients with schizophrenia: A meta-analysis. Psychiatry Res. 2023;321:115104. doi: 10.1016/j.psychres.2023.115104. Epub 2023 Feb 8. PMID: 36774749.	Review
1347	Isabelinha B, Cruz-Ferreira A, Maximiano J, Almeida G. Effects of body- oriented therapies on the negative symptoms in people with schizophrenia: A systematic review. J Bodyw Mov Ther. 2023;33:189-201. doi: 10.1016/j.jbmt.2022.09.009. Epub 2022 Sep 23. PMID: 36775518.	No drug
1348	Berglund AM, James SH, Raugh IM, Strauss GP. Beliefs about the uncontrollability and usefulness of emotion in the schizophrenia-spectrum: links to emotion regulation and negative symptoms. Cognit Ther Res. 2023;47(2):282-294. doi: 10.1007/s10608-023-10357-w. Epub 2023 Feb 3. PMID: 36779179; PMCID: PMC9894745.	No drug
1349	Šagud M, Madžarac Z, Nedic Erjavec G, Šimunović Filipčić I, Mikulić FL, Rogić D, Bradaš Z, Bajs Janović M, Pivac N. The associations of neutrophil- lymphocyte, platelet-lymphocyte, monocyte-lymphocyte ratios and immune- inflammation index with negative symptoms in patients with schizophrenia. Biomolecules. 2023;13(2):297. doi: 10.3390/biom13020297. PMID: 36830666; PMCID: PMC9952992.	No drug
1350	Wang Z, Ling Y, Wang Y, Zhu T, Gao J, Tang X, Yu M, Zhou C, Xu Y, Zhang X, Zhang X, Fang X. The role of two factors of negative symptoms and cognition on social functioning in male patients with schizophrenia: A mediator model. Brain Sci. 2023;13(2):187. doi: 10.3390/brainsci13020187. PMID: 36831730; PMCID: PMC9953813.	No drug
1351	Kim M, Lee Y, Kang H. Effects of exercise on positive symptoms, negative symptoms, and depression in patients with schizophrenia: A systematic review and meta-analysis. Int J Environ Res Public Health. 2023;20(4):3719. doi: 10.3390/ijerph20043719. PMID: 36834415; PMCID: PMC9967614.	Review
1352	Samochowiec J, Jabłoński M, Plichta P, Piotrowski P, Stańczykiewicz B, Bielawski T, Misiak B. The self-evaluation of negative symptoms in differentiating deficit schizophrenia: The comparison of sensitivity and specificity with other tools. Psychopathology. 2023;56(6):453-461. doi: 10.1159/000529244. Epub 2023 Mar 6. PMID: 36878191.	No drug
1353	Reddy LF, Glynn SM, McGovern JE, Sugar CA, Reavis EA, Green MF. A novel psychosocial intervention for motivational negative symptoms in schizophrenia: Combined motivational interviewing and CBT. Am J Psychiatry. 2023;180(5):367-376. doi: 10.1176/appi.ajp.20220243. Epub 2023 Mar 9. PMID: 36891649.	No drug
1354	Luther L, Raugh IM, Collins DE, Knippenberg AR, Strauss GP. Negative symptoms in schizophrenia differ across environmental contexts in daily life. J Psychiatr Res. 2023;161:10-18. doi: 10.1016/j.jpsychires.2023.02.037. Epub 2023 Mar 1. PMID: 36893666; PMCID: PMC10149609.	No drug
1355	Witkowski G, Januszko P, Skalski M, Mach A, Wawrzyniak ZM, Poleszak E, Ciszek B, Radziwoń-Zaleska M. Factors contributing to risk of persistence of positive and negative symptoms in schizophrenia during hospitalization. Int J Environ Res Public Health. 2023;20(5):4592. doi: 10.3390/ijerph20054592. PMID: 36901603; PMCID: PMC10001938.	No drug
1356	Cella M, Roberts S, Pillny M, Riehle M, O'Donoghue B, Lyne J, Tomlin P, Valmaggia L, Preti A. Psychosocial and behavioural interventions for the negative symptoms of schizophrenia: a systematic review of efficacy meta- analyses. Br J Psychiatry. 2023;223(1):321-331. doi: 10.1192/bjp.2023.21. PMID: 36919340; PMCID: PMC10331321.	Review
1357	Wang X, Chang Z, Wang R. Opposite effects of positive and negative symptoms on resting-state brain networks in schizophrenia. Commun Biol. 2023;6(1):279. doi: 10.1038/s42003-023-04637-0. PMID: 36932140; PMCID: PMC10023794.	No drug
1358	Rabinowitz J, Staner C, Saoud J, Weiser M, Kuchibhatla R, Davidson M, Harvey PD, Luthringer R. Long-term effects of Roluperidone on negative symptoms of schizophrenia. Schizophr Res. 2023;255:9-13. doi: 10.1016/j.schres.2023.03.028. Epub 2023 Mar 16. PMID: 36933291.	Overlap= 966,1087,1269
1359	Tharoor H, Maran S, Chandan AK, Pari M, Rao S, Durairaj J. Cognitive and negative symptoms in schizophrenia with L-Carnosine adjuvant therapy – A randomized double-blind placebo-controlled study. Pharmacol Res Perspect. 2023;11(2):e01074. doi: 10.1002/prp2.1074. PMID: 36946070; PMCID: PMC10031293.	**Included**
1360	Gao Z, Xiu M, Liu J, Wu F, Zhang XY. Obesity, antioxidants and negative symptom improvement in first-episode schizophrenia patients treated with risperidone. Schizophrenia (Heidelb). 2023;9(1):17. doi: 10.1038/s41537-023-00346-z. PMID: 36949120; PMCID: PMC10033648.	Open
1361	Vrublevska J. Case report: Successful administration of cariprazine in a young, severely ill patient with recurrent relapses of schizophrenia and persistent negative symptoms. Front Psychiatry. 2023;14:1134692. doi: 10.3389/fpsyt.2023.1134692. PMID: 36970274; PMCID: PMC10034963.	Case
1362	Schmitt A, Maurus I, Falkai P. Treatment of negative symptoms in schizophrenia: a challenge for clinical research. Eur Arch Psychiatry Clin Neurosci. 2023;273(3):525-526. doi: 10.1007/s00406-023-01595-w. PMID: 36977788; PMCID: PMC10085889.	Opinion
1363	Brasso C, Colli G, Sgro R, Bellino S, Bozzatello P, Montemagni C, Villari V, Rocca P. Efficacy of serotonin and dopamine activity modulators in the treatment of negative symptoms in schizophrenia: A rapid review. Biomedicines. 2023;11(3):921. doi: 10.3390/biomedicines11030921. PMID: 36979900; PMCID: PMC10046337.	Review
1364	Fuentes-Claramonte P, Garcia-Leon MA, Salgado-Pineda P, Ramiro N, Soler- Vidal J, Torres ML, Cano R, Argila-Plaza I, Panicali F, Sarri C, Jaurrieta N, Sánchez M, Boix-Quintana E, Albacete A, Maristany T, Sarró S, Raduà J, McKenna PJ, Salvador R, Pomarol-Clotet E. Do the negative symptoms of schizophrenia reflect reduced responsiveness to reward? Examination using a reward prediction error (RPE) task. Psychol Med. 2023;53(15):7106-7115. doi: 10.1017/S0033291723000521. Epub 2023 Mar 29. PMID: 36987680; PMCID: PMC10719670.	No drug
1365	Suen YN, Pang SWT, Cheung C, Wong TY, Hui LMC, Lee HME, Chang WC, Chen YHE, Chan SKW. Impact of early negative symptom patterns on the long-term outcomes of patients with first-episode schizophrenia-spectrum disorders: A 12-year follow up study. Psychiatry Res. 2023;323:115180. doi: 10.1016/j.psychres.2023.115180. Epub 2023 Mar 24. PMID: 36989910.	No drug
1366	Mayeli A, LaGoy AD, Smagula SF, Wilson JD, Zarbo C, Rocchetti M, Starace F, Zamparini M, Casiraghi L, Calza S, Rota M, D'Agostino A, de Girolamo G; DiAPAson Consortium; Ferrarelli F. Shared and distinct abnormalities in sleep-wake patterns and their relationship with the negative symptoms of Schizophrenia Spectrum Disorder patients. Mol Psychiatry. 2023;28(5):2049-2057. doi: 10.1038/s41380-023-02050-x. Epub 2023 Apr 14. PMID: 37055512.	No drug
1367	Han Almis B, Tekin A, Hakyemez Geylani G, Baltaci E, Egeli A. Is there a Relationship Between Caffeine Intake and Smoking and Positive and Negative Symptom Severity in Schizophrenia? Psychiatr Danub. 2023;35(1):56-61. doi: 10.24869/psyd.2023.56. PMID: 37060593.	No drug

1368	Pu Z, Wen H, Jiang H, Hou Q, Yan H. Berberine improves negative symptoms and cognitive function in patients with chronic schizophrenia via anti- inflammatory effect: a randomized clinical trial. Chin Med. 2023;18(1):41. doi: 10.1186/s13020-023-00746-4. PMID: 37069570; PMCID: PMC10108529.	Open
1369	Zhai Z, Ren L, Song Z, Xiang Q, Zhuo K, Zhang S, Li X, Zhang Y, Jiao X, Tong S, Sun J, Liu D. The efficacy of low-intensity transcranial ultrasound stimulation on negative symptoms in schizophrenia: A double-blind, randomized sham-controlled study. Brain Stimul. 2023;16(3):790-792. doi: 10.1016/j.brs.2023.04.021. Epub 2023 Apr 29. PMID: 37121354.	No drug
1370	Chai J, Liu F, Liu L, Hu N, Huang W, Wang H, Cui Y, Liu H, Li X, Li Y. The efficacy of homestyle rehabilitation on negative symptoms in chronic schizophrenia: A randomized controlled trial. Front Psychiatry. 2023;14:1138794. doi: 10.3389/fpsyt.2023.1138794. PMID: 37139315; PMCID: PMC10149672.	No drug
1371	Zhou Q, Pu CC, Huang BJ, Miao Q, Zhou TH, Cheng Z, Gao TQ, Shi C, Yu X. Optimal cutoff scores of the Chinese version of 15-item negative symptom assessment that indicate prominent negative symptoms of schizophrenia. Front Psychiatry. 2023;14:1154459. doi: 10.3389/fpsyt.2023.1154459. PMID: 37139322; PMCID: PMC10149848.	No drug
1372	Penadés R, Wykes T. Use of cognitive remediation to treat negative symptoms in schizophrenia: is it time yet? Br J Psychiatry. 2023;223(1):319-320. doi: 10.1192/bjp.2023.50. PMID: 37194957.	No drug
1373	Galderisi S, Kaiser S. The pathophysiology of negative symptoms of schizophrenia: main hypotheses and open challenges. Br J Psychiatry. 2023;223(1):298-300. doi: 10.1192/bjp.2023.63. PMID: 37232136; PMCID: PMC10331317.	No drug
1374	Compton MT, Ku BS, Covington MA, Metzger C, Hogoboom A. Lexical Diversity and Other Linguistic Measures in Schizophrenia: Associations With Negative Symptoms and Neurocognitive Performance. J Nerv Ment Dis. 2023;211(8):613-620. doi: 10.1097/NMD.0000000000001672. Epub 2023 May 31. PMID: 37256631.	No drug
1375	Sulejmanpasic G, Memic-Serdarevic A, Sabanagic-Hajric S, Bajramagic N. The Correlation of Positive and Negative Symptoms (PANSS Scores) in Patients with Schizophrenia According to Gender. Med Arch. 2023;77(2):123-126. doi: 10.5455/medarh.2023.77.123-126. PMID: 37260804; PMCID: PMC10227848.	No drug
1376	Howes O, Fusar-Poli P, Osugo M. Treating negative symptoms of schizophrenia: current approaches and future perspectives. Br J Psychiatry. 2023;223(1):332-335. doi: 10.1192/bjp.2023.57. PMID: 37272623.	Review
1377	Racher M, Carpenter W, Kane JM. A Patient-Clinician Discussion of Current Challenges in Schizophrenia Part 2: Negative Symptoms in Schizophrenia [Podcast]. Neuropsychiatr Dis Treat. 2023;19:1339-1345. doi: 10.2147/NDT.S419397. PMID: 37292182; PMCID: PMC10244616.	Opinion
1378	Habtewold TD, Tiles-Sar N, Liemburg EJ, Sandhu AK, Islam MA, Boezen HM; GROUP Investigators; Bruggeman R, Alizadeh BZ. Six-year trajectories and associated factors of positive and negative symptoms in schizophrenia patients, siblings, and controls: Genetic Risk and Outcome of Psychosis (GROUP) study. Sci Rep. 2023;13(1):9391. doi: 10.1038/s41598-023-36235-9. PMID: 37296301; PMCID: PMC10256804.	No drug
1379	Fernandez-Egea E, Mucci A, Lee J, Kirkpatrick B. A new era for the negative symptoms of schizophrenia. Br J Psychiatry. 2023;223(1):269-270. doi: 10.1192/bjp.2023.69. Epub 2023 Jul 10. PMID: 37424202.	Opinion
1380	Galderisi S, Mucci A. A new approach to negative symptoms of schizophrenia. Eur Neuropsychopharmacol. 2023;75:62-64. doi: 10.1016/j.euroneuro.2023.06.008. Epub 2023 Jul 14. PMID: 37454626.	Opinion
1381	Kalisova L, Michalec J, Dechterenko F, Silhan P, Hyza M, Chlebovcova M, Brenova M, Bezdicek O. Impact of cognitive performance and negative symptoms on psychosocial functioning in Czech schizophrenia patients. Schizophrenia (Heidelb). 2023;9(1):43. doi: 10.1038/s41537-023-00374-9. PMID: 37460587; PMCID: PMC10352309.	No drug
1382	Li M, Luo G, Qiu Y, Zhang X, Sun X, Li Y, Zhao Y, Sun W, Yang S, Li J. Negative symptoms and neurocognition in drug-naïve schizophrenia: moderating role of plasma neutrophil gelatinase-associated lipocalin (NGAL) and interferon- gamma (INF-γ). Eur Arch Psychiatry Clin Neurosci. 2023. doi: 10.1007/s00406-023-01650-6. Epub ahead of print 2023 Jul 25. PMID: 37490111.	No drug
1383	Shatalina E, Ashok AH, Wall MB, Nour MM, Myers J, Reis Marques T, Rabiner EA, Howes OD. Reward processing in schizophrenia and its relation to Mu opioid receptor availability and negative symptoms: A [11C]-carfentanil PET and fMRI study. Neuroimage Clin. 2023;39:103481. doi: 10.1016/j.nicl.2023.103481. Epub 2023 Jul 24. PMID: 37517175; PMCID: PMC10400918.	No drug
1384	Marder SR, Umbricht D. Negative symptoms in schizophrenia: Newly emerging measurements, pathways, and treatments. Schizophr Res. 2023;258:71-77. doi: 10.1016/j.schres.2023.07.010. Epub 2023 Jul 28. PMID: 37517366.	Review
1385	Vöckel J, Thiemann U, Weisbrod M, Schröder J, Resch F, Klein C, Bender S. Movement initiation and preparation in subjects with schizophrenia - The amplitude of the readiness potential as a biological marker for negative symptom severity. Schizophr Res. 2023;260:3-11. doi: 10.1016/j.schres.2023.07.012. Epub 2023 Aug 3. PMID: 37543008.	No drug
1386	Das M, Saxena A, Samantaray S, Kumar S, Raj Pc J, Goyal N. Intermittent theta burst stimulation using H7 coil for management of negative symptoms and cognitive dysfunction in chronic schizophrenia: A case report. J ECT. 2024;40(1):61-62. doi: 10.1097/YCT.0000000000000958. Epub 2023 Aug 3. PMID: 37561931.	Case
1387	Emami M, Kheirabadi G, Fallahi M. The effect of Lieberman Community Return Program on reducing positive and negative symptoms and improving social skills in patients with schizophrenia. Adv Biomed Res. 2023;12:146. doi: 10.4103/abr.abr_21_21. PMID: 37564452; PMCID: PMC10410411.	No drug
1388	Rajagopal L, Mahjour S, Huang M, Ryan CA, Elzokaky A, Csakai AJ, Orr MJ, Scheidt K, Meltzer HY. NU-1223, a simplified analog of alstonine, with 5-HT2cR agonist-like activity, rescues memory deficit and positive and negative symptoms in subchronic phencyclidine mouse model of schizophrenia. Behav Brain Res. 2023;454:114614. doi: 10.1016/j.bbr.2023.114614. Epub 2023 Aug 10. PMID: 37572758.	Animal
1389	Fekih-Romdhane F, Hajje R, Haddad C, Hallit S, Azar J. Exploring negative symptoms heterogeneity in patients diagnosed with schizophrenia and schizoaffective disorder using cluster analysis. BMC Psychiatry. 2023;23(1):595. doi: 10.1186/s12888-023-05101-3. PMID: 37582728; PMCID: PMC10428523.	No drug
1390	Strauss GP. Environmental factors contributing to negative symptoms in youth at clinical high risk for psychosis and outpatients with schizophrenia. Soc Psychiatry Psychiatr Epidemiol. 2023. doi: 10.1007/s00127-023-02556-3. Epub ahead of print 2023 Aug 25. PMID: 37624464.	No drug
1391	Saleh Y, Jarratt-Barnham I, Petitet P, Fernandez-Egea E, Manohar SG, Husain M. Negative symptoms and cognitive impairment are associated with distinct motivational deficits in treatment resistant schizophrenia. Mol Psychiatry. 2023;28(11):4831-4841. doi: 10.1038/s41380-023-02232-7. Epub 2023 Aug 25. PMID: 37626135; PMCID: PMC10914595.	No drug
1392	Cengisiz C, Misir E. Dimensional characteristics of persistent negative symptoms in schizophrenia and their relationships with schizotypy in first- degree relatives. Nord J Psychiatry. 2023;77(8):737-746. doi: 10.1080/08039488.2023.2250777. Epub 2023 Aug 30. PMID: 37646862.	No drug
1393	Hu N, Li W, Deng H, Song J, Yang H, Chai J, Huang W, Wang H, Zhou X, Zhang P, He S, Cui Y, Fan T, Li Y. The mediating role of negative symptoms in "secondary factors" determining social functioning in chronic schizophrenia. Front Psychiatry. 2023;14:1196760. doi: 10.3389/fpsyt.2023.1196760. PMID: 37649558; PMCID: PMC10464835.	No drug
1394	Cella M, Tomlin P, Robotham D, Green P, Griffiths H, Stahl D, Valmaggia L. Virtual reality supported therapy for the negative symptoms of schizophrenia: the V-NeST feasibility RCT. Southampton (UK): National Institute for Health and Care Research; 2023 Sep. PMID: 37851846.	No drug
1395	Rucci P, Caporusso E, Sanmarchi F, Giordano GM, Mucci A, Giuliani L, Pezzella P, Perrottelli A, Bucci P, Rocca P, Rossi A, Bertolino A, Galderisi S, Maj M; Italian Network for Research on Psychoses. The structure stability of negative symptoms: longitudinal network analysis of the Brief Negative Symptom Scale in people with schizophrenia. BJPsych Open. 2023;9(5):e168. doi: 10.1192/bjo.2023.541. PMID: 37674282 PMCID: PMC10594087.	No drug

1396	Xiu M, Zhao L, Sun Q, Lang X. Efficacy of low-dose olanzapine in combination with sertraline on negative symptoms and psychosocial functioning in schizophrenia: A randomized controlled trial. Curr Neuropharmacol. 2023. doi: 10.2174/1570159X21666230913152344. Epub ahead of print 2023 Sep 13. PMID: 37711125.	Open
1397	Starzer M, Hansen HG, Hjorthøj C, Albert N, Nordentoft M, Madsen T. 20-year trajectories of positive and negative symptoms after the first psychotic episode in patients with schizophrenia spectrum disorder: results from the OPUS study. World Psychiatry. 2023;22(3):424-432. doi: 10.1002/wps.21121. PMID: 37713547; PMCID: PMC10503930.	No drug
1398	Zhang L, James SH, Strauss GP. Environmental resource reductions predict greater severity of negative symptoms in schizophrenia. Schizophr Res. 2023;261:94-99. doi: 10.1016/j.schres.2023.09.013. Epub 2023 Sep 14. PMID: 37716206; PMCID: PMC10840833.	No drug
1399	Mezquida G, Amoretti S, Bioque M, García-Rizo C, Sánchez-Torres AM, Pina- Camacho L, Lopez-Pena P, Mané A, Rodriguez-Jimenez R, Corripio I, Sarró S, Ibañez A, Usall J, García-Portilla MP, Vieta E, Mas S, Cuesta MJ, Parellada M, González-Pinto A, Berrocoso E, Bernardo M; 2EPs Group. Identifying risk factors for predominant negative symptoms from early stages in schizophrenia: A longitudinal and sex-specific study in first-episode schizophrenia patients. Span J Psychiatry Ment Health. 2023;16(3):159-168. doi: 10.1016/j.rpsm.2023.01.004. Epub 2023 Feb 1. PMID: 37716849.	No drug
1400	Zeng J, Zhang W, Lu X, Zhou H, Huang J, Xu Z, Liao H, Liang J, Liang M, Ye C, Sun T, Hu Y, She Q, Chen H, Guo Q, Yan L, Wu R, Li Z. The association of SOD and HsCRP with the efficacy of sulforaphane in schizophrenia patients with residual negative symptoms. Eur Arch Psychiatry Clin Neurosci. 2023. doi: 10.1007/s00406-023-01679-7. Epub ahead of print 2023 Sep 20. PMID: 37728803.	Open
1401	D'Anna G, Zarbo C, Cardamone G, Zamparini M, Calza S, Rota M, Correll CU, Rocchetti M, Starace F, de Girolamo G; DIAPASON collaborators. Interplay between negative symptoms, time spent doing nothing, and negative emotions in patients with schizophrenia spectrum disorders: results from a 37-site study. Schizophrenia (Heidelb). 2023;9(1):63. doi: 10.1038/s41537-023-00372-x. PMID: 37735175; PMCID: PMC10514038.	No drug
1402	Li Y, Rekhi G, Ang MS, Lee J. Impact of negative symptoms on health- related quality of life in schizophrenia. Front Psychiatry. 2023;14:1252354. doi: 10.3389/fpsyt.2023.1252354. PMID: 37744001; PMCID: PMC10512711.	No drug
1403	Pierce B. Transcranial Magnetic Stimulation-A Patient's First-Person Account on the Treatment of Schizophrenia's Negative Symptoms (Avolition). Schizophr Bull. 2023:sbad141. doi: 10.1093/schbul/sbad141. Epub ahead of print 2023 Sep 29. PMID: 37774403.	No drug
1404	Franza F, Soddu A, Fiorentino N, Calabrese L, Zarrella A, Solomita B, Tavormina G. The interference of negative symptoms of schizophrenia on cognitive domains: a long-term observational study on the role of clozapine. Psychiatr Danub. 2023;35(Suppl 2):132-135. PMID: 37800215.	Open
1405	Ghosh S, Deka B. Relation of Vitamin D levels with positive and negative symptoms of schizophrenia - A hospital based cross-sectional comparative study. Indian J Psychiatry. 2023;65(9):955-960. doi: 10.4103/indianjpsychiatry.indianjpsychiatry_355_23. Epub 2023 Sep 5. PMID: 37841540; PMCID: PMC10569326.	No drug
1406	Huo L, Qu D, Pei C, Wu W, Ning Y, Zhou Y, Zhang XY. Alexithymia in chronic schizophrenia and its mediating effect between cognitive deficits and negative symptoms. Schizophr Res. 2023 Nov;261:275-280. doi: 10.1016/j.schres.2023.10.006. Epub 2023 Oct 20. PMID: 37866075.	No drug
1407	Zhu T, Wang Z, Wu W, Ling Y, Wang Z, Zhou C, Fang X, Huang C, Xie C, Chen J, Zhang X. Altered brain functional networks in schizophrenia with persistent negative symptoms: an activation likelihood estimation meta-analysis. Front Hum Neurosci. 2023;17:1204632. doi: 10.3389/fnhum.2023.1204632. PMID: 37954938; PMCID: PMC10637389.	Review
1408	Levin Y, Bachem R, Brafman D, Ben-Ezra M. The association between dissociative symptoms and schizophrenia-related negative symptoms: A transdiagnostic approach. J Psychiatr Res. 2024;169:81-83. doi: 10.1016/j.jpsychires.2023.11.016. Epub 2023 Nov 18. PMID: 38006822.	No drug
1409	Wolpe N, Vituri A, Jones PB, Shahar M, Fernandez-Egea E. The longitudinal structure of negative symptoms in treatment resistant schizophrenia. Compr Psychiatry. 2024;128:152440. doi: 10.1016/j.comppsych.2023.152440. Epub 2023 Nov 27. PMID: 38039918.	No drug
1410	Ma CC, Lin YY, Chung YA, Park SY, Huang CC, Chang WC, Chang HA. The two- back task leads to activity in the left dorsolateral prefrontal cortex in schizophrenia patients with predominant negative symptoms: a fNIRS study and its implication for tDCS. Exp Brain Res. 2024;242(3):585-597. doi: 10.1007/s00221-023-06769-5. Epub 2024 Jan 16. PMID: 38227007.	No drug
1411	Yi S, Wang Q, Wang W, Hong C, Ren Z. Efficacy of repetitive transcranial magnetic stimulation (rTMS) on negative symptoms and cognitive functioning in schizophrenia: An umbrella review of systematic reviews and meta-analyses. Psychiatry Res. 2024;333:115728. doi: 10.1016/j.psychres.2024.115728. Epub 2024 Jan 12. PMID: 38232567.	Review
1412	Kong L, Zhang Y, Wu XM, Wang XX, Wu HS, Li SB, Chu MY, Wang Y, Lui SSY, Lv QY, Yi ZH, Chan RCK. The network characteristics in schizophrenia with prominent negative symptoms: a multimodal fusion study. Schizophrenia (Heidelb). 2024;10(1):10. doi: 10.1038/s41537-023-00408-2. PMID: 38233433; PMCID: PMC10851703.	No drug
1413	Tan X, Goh SE, Lee JJ, Vanniasingham SD, Brunelin J, Lee J, Tor PC. Efficacy of using intermittent theta burst stimulation to treat negative symptoms in patients with schizophrenia-A systematic review and meta-analysis. Brain Sci. 2023;14(1):18. doi: 10.3390/brainsci14010018.	Review
1414	Zedan SA, Zahid A, Best MW. Examining the effects of diagnostic awareness, positive symptoms, and negative symptoms on stigmatizing attitudes and social exclusion towards schizophrenia. Schizophr Res. 2024;264:482-490. doi: 10.1016/j.schres.2024.01.023. Epub 2024 Jan 25.	No drug
1415	Du N, Meng X, Li J, Shi L, Zhang X. Decline in working memory in stable schizophrenia may be related to attentional impairment: Mediating effects of negative symptoms, a cross-sectional study. Neuropsychiatr Dis Treat. 2024;20:149-158. doi: 10.2147/NDT.S447965.	No drug
1416	Zierhut M, Bergmann N, Hahne I, Wohlthan J, Kraft J, Braun A, Tam Ta TM, Hellmann-Regen J, Ripke S, Bajbouj M, Hahn E, Böge K. The combination of oxytocin and mindfulness-based group therapy for empathy and negative symptoms in schizophrenia spectrum disorders – A double-blinded, randomized, placebo-controlled pilot study. J Psychiatr Res. 2024;171:222-229. doi: 10.1016/j.jpsychires.2024.01.014. Epub 2024 Jan 9. PMID: 38309212.	**Included**
1417	Giordano GM, Pezzella P, Mucci A, Austin SF, Erfurth A, Glenthøj B, Hofer A, Hubenak J, Libiger J, Melle I, Nielsen MØ, Rybakowski JK, Wojciak P, Galderisi S, Sachs G. Negative symptoms and social cognition as mediators of the relationship between neurocognition and functional outcome in schizophrenia. Front Psychiatry. 2024;15:1333711. doi: 10.3389/fpsyt.2024.1333711. PMID: 38356912; PMCID: PMC10864497.	No drug
1418	Hart XM, Mitsukura Y, Bies RR, Uchida H. Unraveling the influence of age, IQ, education, and negative symptoms on neurocognitive performance in schizophrenia: A conditional inference tree analysis. Pharmacopsychiatry. 2024;57(2):53-60. doi: 10.1055/a-2258-0379. Epub 2024 Feb 22. PMID: 38387603.	No drug
1419	Chen Y, Li Z, Yan C, Zou L. Is it more effective for anhedonia and avolition? A systematic review and meta-analysis of non-invasive brain stimulation interventions for negative symptoms in schizophrenia. CNS Neurosci Ther. 2024;30(3):e14645. doi: 10.1111/cns.14645. PMID: 38432851; PMCID: PMC10909625.	Review
1420	Retraction: Abdallah MS, Mosalam EM, Hassan A, et al. Pentoxifylline as an adjunctive in treatment of negative symptoms in chronic schizophrenia: A double- blind, randomized, placebo-controlled trial. CNS Neurosci. Ther. 2023;29:354-364 (https://doi.org/10.1111/cns.14010). CNS Neurosci Ther. 2024;30(3):e14667. doi: 10.1111/cns.14667. PMID: 38465461; PMCID: PMC10926050.	Retracted/ Removed
1421	Retraction: Abdallah MS, Mosalam EM, Hassan A, et al. Pentoxifylline as an adjunctive in treatment of negative symptoms in chronic schizophrenia: A double- blind, randomized, placebo-controlled trial. CNS Neurosci. Ther. 2023;29:354-364 (https://doi.org/10.1111/cns.14010). CNS Neurosci Ther. 2024;30(3):e14668. doi: 10.1111/cns.14668. PMID: 38465469; PMCID: PMC10926048.	Duplicate= 1420

1422	Sun X, He R, Xiao Y, Xiu M, Sun M, Wu F, Zhang XY. Interaction between baseline BMI and baseline disease severity predicts greater improvement in negative symptoms in first-episode schizophrenia. Eur Arch Psychiatry Clin Neurosci. 2024. doi: 10.1007/s00406-024-01763-6. Epub ahead of print 2024 Mar 27. PMID: 38536473.	No drug
1423	Cohen-Laroque J, Grangier I, Perez N, Kirschner M, Kaiser S, Sabé M. Positive and negative symptoms in methamphetamine-induced psychosis compared to schizophrenia: A systematic review and meta-analysis. Schizophr Res. 2024;267:182-190. doi: 10.1016/j.schres.2024.03.037. Epub ahead of print. PMID: 38554698.	Review
1424	Aydın A, Ersoy Özcan B, Kaya Y. The effect of an emotion recognition and expression program on the alexithymia, emotion expression skills and positive and negative symptoms of patients with schizophrenia in a community mental health center. Issues Ment Health Nurs. 2024:1-9. doi: 10.1080/01612840.2024.2326951. Epub ahead of print 2024 Apr 2. PMID: 38563973.	No drug
1425	Zhai W, Li M, Su Z, Ji Q, Xiong Z, Zhao Y, Yang Y, Liao D, Li C, Wang C. The effect of repetitive transcranial magnetic stimulation on the negative symptoms of chronic schizophrenia and serum brain-derived neurotrophic factor. Psychiatr Pol. 2023;57(6):1293-1303. English, Polish. doi: 10.12740/PP/153375. Epub 2023 Dec 31. PMID: 38564527.	No drug
1426	Kong L, Zhang Y, Wu XM, Wang XX, Wu HS, Li SB, Chu MY, Wang Y, Lui SSY, Lv QY, Yi ZH, Chan RCK. Author Correction: The network characteristics in schizophrenia with prominent negative symptoms: a multimodal fusion study. Schizophrenia (Heidelb). 2024;10(1):43. doi: 10.1038/s41537-024-00467-z. Erratum for: Schizophrenia (Heidelb). 2024;10(1):10. PMID: 38589443.	Duplicate= 1412
1427	Amoretti S, Mezquida G, Verdolini N, Bioque M, Sánchez-Torres AM, Pina-Camacho L, Zorrilla I, Trabsa A, Rodriguez-Jimenez R, Corripio I, Sarró S, Ibañez A, Usall J, Segarra R, Vieta E, Roberto N, Ramos-Quiroga JA, Tortorella A, Menculini G, Cuesta MJ, Parellada M, González-Pinto A, Berrocoso E, Bernardo M; 2EPs Group; Annex. Members of the 2EPs group. Negative symptoms and sex differences in first episode schizophrenia: What's their role in the functional outcome? A longitudinal study. Span J Psychiatry Ment Health. 2023:S2950-2853(23)00010-8. doi: 10.1016/j.sjpmh.2023.04.001. Epub ahead of print 2023 Sep 14. PMID: 38591832.	No drug
1428	Herizchi S, Shafiee-Kandjani AR, Farahbakhsh M, Jahangiri Z, Ghanbarzadeh Javid S, Azizi H. Efficacy of rivastigmine augmentation on positive and negative symptoms, general psychopathology, and quality of life in patients with chronic schizophrenia: A randomized controlled trial. Psychopharmacol Bull. 2024;54(2):15-27. PMID: 38601834; PMCID: PMC11003261.	Lumping
1429	Isıklı S, Bektaş AB, Temel Ş, Atabay M, Arkalı BD, Bağcı B, Bayrakcı A, Sebold M, Zorlu N. Effort-cost decision-making associated with negative symptoms in schizophrenia and bipolar disorder. Behav Brain Res. 2024:114996. doi: 10.1016/j.bbr.2024.114996. Epub ahead of print 2024 Apr 10. PMID: 38609021.	No drug
1430	Carruzzo F, Kaliuzhna M, Kuenzi N, Geffen T, Katthagen T, Schlagenhauf F, Kaiser S. Striatal response to reward anticipation as a biomarker for schizophrenia and negative symptoms: Effects, test-retest reliability, and stability across sites. Schizophr Bull.2024:sbae046. doi: 10.1093/schbul/sbae046. Epub ahead of print 2024 Apr 19. PMID: 38641344.	No drug
1431	Nishimura Y, Yotsumoto K, Hashimoto T. Impacts of a self-directed social resources study program on negative symptoms and quality of life in schizophrenia outpatients: A randomized controlled trial. Kobe J Med Sci. 2024;70(1):E15-E21. doi: 10.24546/0100488377. PMID: 38644296.	No drug
1432	Geffen T, Hardikar S, Smallwood J, Kaliuzhna M, Carruzzo F, Böge K, Zierhut MM, Gutwinski S, Katthagen T, Kaiser S, Schlagenhauf F. Striatal functional hypoconnectivity in patients with schizophrenia suffering from negative symptoms, longitudinal findings. Schizophr Bull. 2024:sbae052. doi: 10.1093/schbul/sbae052. Epub ahead of print 2024 Apr 30.	No drug
1433	Selvan P, Devkare P, Shetty A, Dharmadhikari S, Khandhedia C, Mane A, Mehta S, Andrade C. A review on the pharmacology of cariprazine and its role in the treatment of negative symptoms of schizophrenia. Front Psychiatry. 2024;15:1385925. doi: 10.3389/fpsyt.2024.1385925. PMID: 38711874; PMCID: PMC11071166.	Review
1434	Muyambi K, Walsh S, Dettwiller P, Tan KL, Dennis S, Bressington D, Gray RJ, McCall A, Jones M. Australian trial of behavioural activation for people with schizophrenia experiencing negative symptoms: a feasibility randomised controlled trial protocol. BMJ Open. 2024;14(5):e080245. doi: 10.1136/bmjopen-2023-080245. PMID: 38719282.	Protocol
1435	Muyambi K, Walsh S, Dan Bressington, Gray R, Dennis S, Brown E, Grimshaw M, Drummond J, Jones M. Efficacy of behavioural activation in the treatment of negative symptoms in people with schizophrenia spectrum disorders: A systematic review. Int J Nurs Stud Adv. 2023;5:100132. doi: 10.1016/j.ijnsa.2023.100132. Online 2024 May 15. PMID: 38746587; PMCID: PMC11080335.	Review
1436	Hou WP, Qin XQ, Hou WW, Han YY, Bo QJ, Dong F, Zhou FC, Li XB, Wang CY. Interaction between catechol-O-methyltransferase Val/Met polymorphism and cognitive reserve for negative symptoms in schizophrenia. World J Psychiatry. 2024;14(5):695-703. doi: 10.5498/wjp.v14.i5.695. PMID: 38808087; PMCID: PMC11129152.	No drug
1437	Hua JPY, Abram SV, Loewy RL, Stuart B, Fryer SL, Vinogradov S, Mathalon DH. Brain age gap in early illness schizophrenia and the clinical high-risk syndrome: Associations with experiential negative symptoms and conversion to psychosis. Schizophr Bull. 2024:sbae074. doi: 10.1093/schbul/sbae074. Epub ahead of print 2024 May 30. PMID: 38815987.	No drug
1438	Chen YH, Yu H, Xue F, Bai J, Guo L, Peng ZW. 16S rRNA gene sequencing reveals altered gut microbiota in young adults with schizophrenia and prominent negative symptoms. Brain Behav. 2024;14(6):e3579. doi: 10.1002/brb3.3579.	No drug
1439	Wang M, Lu S, Hao L, Xia Y, Shi Z, Su L. Placebo effects of repetitive transcranial magnetic stimulation on negative symptoms and cognition in patients with schizophrenia spectrum disorders: a systematic review and meta-analysis. Front Psychiatry. 2024 May 28;15:1377257. doi: 10.3389/fpsyt.2024.1377257.	No drug
1440	Guha A, Popov T, Bartholomew ME, Reed AC, Diehl CK, Subotnik KL, Ventura J, Nuechterlein KH, Miller GA, Yee CM. Task-based default mode network connectivity predicts cognitive impairment and negative symptoms in first-episode schizophrenia. Psychophysiology. 2024:e14627. doi: 10.1111/psyp.14627. Epub ahead of print Jun 24 2024. PMID: 38924105.	No drug
1441	Filip TF, Hellemann GS, Ventura J, Subotnik KL, Green MF, Nuechterlein KH, McCleery A. Defeatist performance beliefs in individuals with recent-onset schizophrenia: Relationships with cognition and negative symptoms. Schizophr Res. 2024;270:212-219. doi: 10.1016/j.schres.2024.06.021. Epub ahead of print Jun 25 2024. PMID: 38924939.	No drug
1442	Du SC, Li CY, Lo YY, Hu YH, Hsu CW, Cheng CY, Chen TT, Hung PH, Lin PY, Chen CR. Effects of visual art therapy on positive symptoms, negative symptoms, and emotions in individuals with schizophrenia: A systematic review and meta-analysis. Healthcare (Basel). 2024;12(11):1156. doi: 10.3390/healthcare12111156.	Review
1443	Hadzi Boskovic D, Smith-Palmer J, Pöhlmann J, Pollock RF, Hwang S, Bruhn D. Systematic literature review of studies reporting measures of functional outcome or quality of life in people with negative symptoms of schizophrenia. Patient Relat Outcome Meas. 2024;15:199-217. doi: 10.2147/PROM.S454845.	Review
1444	Guha A, Popov T, Bartholomew ME, Reed AC, Diehl CK, Subotnik KL, Ventura J, Nuechterlein KH, Miller GA, Yee CM. Task-based default mode network connectivity predicts cognitive impairment and negative symptoms in first-episode schizophrenia. Psychophysiology. 2024:e14627. doi: 10.1111/psyp.14627. Epub ahead of print 2024 Jun 24.	Duplicate= 1440
1445	Filip TF, Hellemann GS, Ventura J, Subotnik KL, Green MF, Nuechterlein KH, McCleery A. Defeatist performance beliefs in individuals with recent-onset schizophrenia: Relationships with cognition and negative symptoms. Schizophr Res. 2024;270:212-219. doi: 10.1016/j.schres.2024.06.021. Epub ahead of print 2024 Jun 25.	No drug
1446	Fulford D, Marsch LA, Pratap A. Prescription digital therapeutics: An emerging treatment option for negative symptoms in schizophrenia. Biol Psychiatry. 2024:S0006-3223(24)01430-6. doi: 10.1016/j.biopsych.2024.06.026. Epub ahead of print 2024 Jul 1. PMID: 38960019.	Review
1447	Chen P, Yang HD, Wang JJ, Zhu ZH, Zhao HM, Yin XY, Cai Y, Zhu HL, Fu JL, Zhang XZ, Sun WX, Hui L, Zhang XB. Association of serum interleukin-6 with negative symptoms in stable early-onset schizophrenia. World J Psychiatry. 2024;14(6):794-803. doi: 10.5498/wjp.v14.i6.794.	No drug

1448	Lui SSY, Lam EHY, Wang LL, Leung PBM, Cheung ESL, Wong CHY, Zhan N, Wong RWK, Siu BWM, Tang DYY, Liu ACY, Chan RCK. Negative symptoms in treatment-resistant schizophrenia and its relationship with functioning. Schizophr Res. 2024;270:459-464. doi: 10.1016/j.schres.2024.07.008. Epub ahead of print 2024 Jul 11. PMID: 38996523.	No drug
1449	Fang J, Cai R, Hu Y, Wang Y, Ling Y, Lv Y, Fang X, Zhang X, Zhou C. Aberrant brain functional connectivity mediates the effects of negative symptoms on cognitive function in schizophrenia: A structural equation model. J Psychiatr Res. 2024;177:109-117. doi: 10.1016/j.jpsychires.2024.07.006. Epub ahead of print 2024 Jul 4.	No drug
1450	Reznik AM, Arbuzov AL, Murin SP, Pavlichenko AV. Negative symptoms of schizophrenia: New prospects of cariprazine treatment. Consort Psychiatr. 2020;1(2):43-51. doi: 10.17650/2712-7672-2020-1-2-43-51. PMID: 39006904; PMCID: PMC11240126. (Резник АМ, Арбузов АЛ, Мурин СП, Павличенко АВ. Негативные симптомы шизофрении: новые перспективы лечения карипразином. Consort Psychiatr. 2020;1(2):43-51. doi: 10.17650/2712-7672-2020-1-2-43-51).	Review
1451	Kushnir YA, Kozhyna HM, Abdryakhimova TB, Panko TV. Assessment of socio-demographic characteristics and social status of patients with negative symptoms in schizophrenia at different stages of the disease. Wiad Lek. 2024;77(5):943-949. doi: 10.36740/WLek202405110. PMID: 39008581.	No drug
1452	James SH, Ahmed AO, Harvey PD, Saoud JB, Davidson M, Kuchibhatla R, Luthringer R, Strauss GP. Network intervention analysis indicates that roluperidone achieves its effect on negative symptoms of schizophrenia by targeting avolition. Eur Neuropsychopharmacol. 2024;87:18-23. doi: 10.1016/j.euroneuro.2024.07.005. Epub ahead of print 2024 Jul 17. PMID: 39024856.	Non-marketed
1453	Donati FL, Mayeli A, Nascimento Couto BA, Sharma K, Janssen S, Krafty RJ, Casali AG, Ferrarelli F. Prefrontal oscillatory slowing in early-course schizophrenia is associated with worse cognitive performance and negative symptoms: a TMS-EEG study. Biol Psychiatry Cogn Neurosci Neuroimaging. 2024:S2451-9022(24)00201-5. doi: 10.1016/j.bpsc.2024.07.013. Epub ahead of print 2024 Jul 24.	No drug
1454	Vergallito A, Gesi C, Torriero S. Intermittent theta burst stimulation combined with cognitive training to improve negative symptoms and cognitive impairment in schizophrenia: A pilot study. Brain Sci. 2024;14(7):683. doi: 10.3390/brainsci14070683.	No drug
1455	Luther L, Raugh IM, Grant PM, Beck AT, Strauss GP. The role of defeatist performance beliefs in state fluctuations of negative symptoms in schizophrenia measured in daily life via ecological momentary assessment. Schizophr Bull. 2024:sbae128. doi: 10.1093/schbul/sbae128. Epub ahead of print 2024 Jul 27.	No drug
1456	Eack SM. Defeatist beliefs about performance are important treatment targets for negative symptoms in the early course of schizophrenia. Schizophr Res. 2024;271:332-333. doi: 10.1016/j.schres.2024.07.037. Epub ahead of print 2024 Jul 31.	No drug
1457	Patel R, Dembek C, Won Y, Kadakia A, Huang X, Zeni C, Pikalov A. A real-world data analysis of electronic health records to investigate the associations of predominant negative symptoms with healthcare resource utilisation, costs and treatment patterns among patients with schizophrenia. BMJ Open. 2024;14(7):e084613. doi: 10.1136/bmjopen-2024-084613.	No drug
1458	Tsui HKH, Wong TY, Sum MY, Chu ST, Hui CLM, Chang WC, Lee EHM, Suen Y, Chen EYH, Chan SKW. Comparison of Negative Symptom Network Structures Between Patients With Early and Chronic Schizophrenia: A Network and Exploratory Graph Analysis. Schizophr Bull. 2024:sbae135. doi: 10.1093/schbul/sbae135. Epub ahead of print 2024 Aug 2.	No drug
1459	Ware K, Misiak B, Hamza EA, Nalla S, Moustafa AA. The impact of childhood trauma on the negative symptoms of schizophrenia. J Nerv Ment Dis. 2024 Aug 9. doi: 10.1097/NMD.0000000000001788. Epub ahead of print 2024 Aug 9.	No drug
1460	Lee KH, Yu CH. Reexamination of the relationships among neurocognition, self-defeatist beliefs, experiential negative symptoms, and social functioning in a sample of patients diagnosed with chronic schizophrenia and schizoaffective disorder. BMC Psychiatry. 2024;24(1):559. doi: 10.1186/s12888-024-06003-8.	No drug
1461	Targum SD, Ge T, Asgharnejad M, Reksoprodjo P, Singh JB, Murthy V. Use of video-recordings of site-based interviews for quality assurance in a study of subjects with schizophrenia and persistent negative symptoms. Schizophr Res. 2024;272:61-68. doi: 10.1016/j.schres.2024.08.014. Epub ahead of print 2024 Aug 26.	No drug
1462	Correll CU, Xiang P, Sarikonda K, Bhagvandas N, Gitlin M. The economic impact of cognitive impairment and negative symptoms in schizophrenia: A targeted literature review with a focus on outcomes relevant to health care decision-makers in the United States. J Clin Psychiatry. 2024 Aug 21;85(3):24r15316. doi: 10.4088/JCP.24r15316.	Review
1463	Xu F, Xu S. Cognitive-behavioral therapy for negative symptoms of schizophrenia: A systematic review and meta-analysis. Medicine (Baltimore). 2024 Sep 6;103(36):e39572. doi: 10.1097/MD.0000000000039572.	Review
1464	Horan WP, Targum SD, Claxton A, Kaul I, Yohn SE, Marder SR, Miller AC, Brannan SK. Efficacy of KarXT on negative symptoms in acute schizophrenia: A post hoc analysis of pooled data from 3 trials. Schizophr Res. 2024;274:57-65. doi: 10.1016/j.schres.2024.08.001. Epub ahead of print 2024 Sep 10.	*Post hoc*
1465	Zhou Q, Zheng Y, Guo X, Wang Y, Pu C, Shi C, Yu X. Abnormal hedonic process in patients with stable schizophrenia: Relationships to negative symptoms and social functioning. Schizophr Res Cogn. 2024 Aug 24;38:100325. doi: 10.1016/j.scog.2024.100325.	No drug
1466	Li Y, Ang MS, Yee JY, See YM, Lee J. Predictors of functioning in treatment-resistant schizophrenia: the role of negative symptoms and neurocognition. Front Psychiatry. 2024;15:1444843. doi: 10.3389/fpsyt.2024.1444843.	No drug
1467	Yeh TC, Lin YY, Tzeng NS, Kao YC, Chung YA, Chang CC, Fang HW, Chang HA. Effects of online high-definition transcranial direct current stimulation over left dorsolateral prefrontal cortex on predominant negative symptoms and EEG functional connectivity in patients with schizophrenia: a randomized, double-blind, controlled trial. Psychiatry Clin Neurosci. 2024. doi: 10.1111/pcn.13745. Epub ahead of print 2024 Sep 24.	No drug
1468	Tsapakis EM, Treiber M, Mitkani C, Drakaki Z, Cholevas A, Spanaki C, Fountoulakis KN. pharmacological treatments of negative symptoms in schizophrenia-An update. J Clin Med. 2024;13(18):5637. doi: 10.3390/jcm13185637. Epub ahead of print 2024 Sep 23.	Review
1469	Fan Y, Tao Y, Wang J, Gao Y, Wei W, Zheng C, Zhang X, Song XM, Northoff G. Irregularity of visual motion perception and negative symptoms in schizophrenia. Schizophrenia (Heidelb). 2024;10(1):82. doi: 10.1038/s41537-024-00496-8. Epub ahead of print 2024 Sep 30.	No drug
1470	Tan XW, Gulwant Singh HK, Koh JZJ, Tan RSY, Tor PC. Personalised transcranial magnetic stimulation for treatment-resistant depression, depression with comorbid anxiety and negative symptoms of schizophrenia: a narrative review. Singapore Med J. 2024;65(10):544-551. doi: 10.4103/singaporemedj.SMJ-2024-133. Epub 2024 Oct 4.	No drug
1471	De Pieri M, Berg X, Georgiadis F, Brakowski J, Burrer A, Sabé M, Kaliuzhna M, Vetter S, Seifritz E, Homan P, Kaiser S, Kirschner M. Negative symptoms and their associations with other clinical variables and working memory across the schizophrenia spectrum and bipolar disorder. Schizophr Bull Open. 2024;5(1):sgae024. doi: 10.1093/schizbullopen/sgae024. Epub 2024 Sep 18.	No drug
1472	Melville G, Preisig C, Zheng M, Kurtz MM. The Effects of Cognitive Remediation on Negative Symptoms in Schizophrenia-Spectrum Illness: A Meta-analytic Investigation of Efficacy. Schizophr Bull. 2024:sbae185. doi: 10.1093/schbul/sbae185. Epub ahead of print 2024 Nov 1.	Review
1473	Moga CI, Pavăl D, Micluția IV. Outlining the absence: From inflammation to a distinct endophenotype for the negative symptoms of schizophrenia. Psychiatr Danub. 2024;36(2):161-173. doi: 10.24869/psyd.2024.161.	No drug
1474	Weidenauer A, Dajic I, Praschak-Rieder N, Willeit M. The dopaminergic basis of negative symptoms in schizophrenia: an addendum. Mol Psychiatry. 2024. doi: 10.1038/s41380-024-02828-7. Epub ahead of print 2024 Nov 20.	No drug
1475	Fekih-Romdhane F, Kerbage G, Hachem N, El Murr M, Haddad G, Loch AA, Abou Khalil R, El Hayek E, Hallit S. The moderating role of COMT gene rs4680 polymorphism between maladaptive metacognitive beliefs and negative symptoms in patients with schizophrenia. BMC Psychiatry. 2024 Nov 20;24(1):831. doi: 10.1186/s12888-024-06275-0.	No drug
1476	Zhang L, James SH, Standridge J, Condray R, Allen DN, Strauss GP. Social network reductions are associated with negative symptoms in schizophrenia. Soc Psychiatry Psychiatr Epidemiol. 2024. doi: 10.1007/s00127-024-02804-0. Epub ahead of print 2024 Dec 11.	No drug

1477	Mazhar S, Shamabadi A, Kazemzadeh K, Farahvash MA, Heidari Dalfard A, Fallahpour B, Khodaei Ardakani MR, Akhondzadeh S. *Crocus sativus* (saffron) adjunct to risperidone for negative symptoms of schizophrenia: a randomized, double-blind, placebo-controlled trial. Int Clin Psychopharmacol. 2024. doi: 10.1097/YIC.0000000000000575. Epub ahead of print 2024 Dec 12.	**Included**
1478	Gao C, Li G, Wang Z, Jiang Q, He R, Sun J, You Y, Zhu Y, Zhao J, Zhang X, Zhou C. The therapeutic effects of theta burst stimulation on negative symptoms in chronic schizophrenia using functional near-infrared spectroscopy. J Psychiatr Res. 2024;181:484-491. doi: 10.1016/j.jpsychires.2024.12.010. Epub ahead of print 2024 Dec 7.	No drug
1479	Wang J, Wei Y, Hu Q, Tang Y, Zhu H, Wang J. The efficacy and safety of dual-target rTMS over dorsolateral prefrontal cortex (DLPFC) and cerebellum in the treatment of negative symptoms in first-episode schizophrenia: Protocol for a multicenter, randomized, double-blind, sham-controlled study. Schizophr Res Cogn. 2024 Nov 29;39:100339. doi: 10.1016/j.scog.2024.100339.	No drug
1480	Moritz AA, Terebova PS, Ivanov MV. Роль иммуновоспалительных факторов в развитии негативных симптомов при шизофрении [The role of immuno-inflammatory factors in the development of negative symptoms in schizophrenia]. Zh Nevrol Psikhiatr Im S S Korsakova. 2024;124(11):42-48. Russian. doi: 10.17116/jnevro202412411142.	Review
1481	Shamabadi A, Rafiei-Tabatabaei ES, Kazemzadeh K, Farahmand K, Fallahpour B, Khodaei Ardakani MR, Akhondzadeh S. Pentoxifylline adjunct to risperidone for negative symptoms of stable schizophrenia: A randomized, double-blind, placebo-controlled trial. Int J Neuropsychopharmacol. 2024:pyae051. doi: 10.1093/ijnp/pyae051. Epub ahead of print 2024 Dec 24.	**Included**
1482	Zhou C, Zhang R, Ding M, Duan W, Fang J, Tang X, Qu Q, Zhang X. Progressive structural alterations associated with negative symptoms in schizophrenia: A causal structural covariance network analysis. Prog Neuropsychopharmacol Biol Psychiatry. 2024;136:111236. doi: 10.1016/j.pnpbp.2024.111236. Epub ahead of print 2024 Dec 26.	No drug
1483	Onitsuka T. New treatment options for negative symptoms in schizophrenia. Psychiatry Clin Neurosci. 2025;79(1):1. doi: 10.1111/pcn.13763.	Opinion
1484	Kamoi R, Mifune Y, Soriano K, Tanioka R, Yamanaka R, Ito H, Osaka K, Umehara H, Shimomoto R, Bollos LA, Kwan RYC, Endo I, Palijo SS, Noguchi K, Mifune K, Tanioka T. Association between dynapenia/sarcopenia, extrapyramidal symptoms, negative symptoms, body composition, and nutritional status in patients with chronic schizophrenia. Healthcare (Basel). 2024;13(1):48. doi: 10.3390/healthcare13010048	No drug
1485	Govil P, Kantrowitz JT. Negative Symptoms in Schizophrenia: An Update on Research Assessment and the Current and Upcoming Treatment Landscape. CNS Drugs. 2025 Jan 12. doi: 10.1007/s40263-024-01151-7. Epub ahead of print.	Review
1486	Gao Z, Xiao Y, Zhu F, Tao B, Zhao Q, Yu W, Bishop JR, Gong Q, Lui S. Neurobiological fingerprints of negative symptoms in schizophrenia identified by connectome-based modeling. Psychiatry Clin Neurosci. 2025. doi: 10.1111/pcn.13782. Epub ahead of print 2025 Jan 15.	No drug
1487	Li J, Mo X, Jiang D, Huang X, Wang X, Xia T, Zhang W. Intermittent theta burst stimulation for negative symptoms in schizophrenia patients with mild cognitive impairment: a randomized controlled trail. Front Psychiatry. 2025 Jan 3;15:1500113. doi: 10.3389/fpsyt.2024.1500113.	No drug
1488	Huang J, Chen A, Jin H, Liu F, Hei G, Teng Z, Xiao J, Wu R, Zhao J, Davis JM, Shao P, Smith RC. Efficacy and safety of sulforaphane added to antipsychotics for the treatment of negative symptoms of schizophrenia: A randomized controlled trial. J Clin Psychiatry. 2025;86(1):24m15272. doi: 10.4088/JCP.24m15272. Published online on January 20, 2025.	**Included**
1489	Mucci A, Leucht S, Giordano GM, Giuliani L, Wehr S, Weigel L, Galderisi S. Assessment of negative symptoms in schizophrenia: From the consensus conference-derived scales to remote digital phenotyping. Brain Sci. 2025;15(1):83. doi: 10.3390/brainsci15010083. Epub 2025 Jan 17.	Review
1490	Snipes C, Dorner-Ciossek C, Hare BD, Besedina O, Campellone T, Petrova M, Lakhan SE, Pratap A. Establishment and maintenance of a digital therapeutic alliance in people living with negative symptoms of schizophrenia: Two exploratory single-arm studies. JMIR Ment Health. 2025;12:e64959. doi: 10.2196/64959. Epub 2025 Jan 27.	No drug
1491	Zhang P, Chen L, Qin Q, Liu C, Zhu H, Hu W, He X, Tang K, Yan Q, Shen H. Enhanced computerized cognitive remediation therapy improved cognitive function, negative symptoms, and GDNF in male long-term inpatients with schizophrenia. Front Psychiatry. 2025;15:1477285. doi: 10.3389/fpsyt.2024.1477285.	No drug
1492	Li J, Jiang D, Huang X, Wang X, Xia T, Zhang W. Intermittent theta burst stimulation for negative symptoms in schizophrenia patients with moderate to severe cognitive impairment: A randomized controlled trial. Psychiatry Clin Neurosci. 2025. doi: 10.1111/pcn.13779. Epub ahead of print 2025 Jan 30.	No drug
1493	Delay C, Link P, Holden J, Granholm E. Using pupillometry to predict outcome in cognitive behavioral therapy for negative symptoms of schizophrenia. Schizophr Res. 2025;276:135-142. doi: 10.1016/j.schres.2025.01.014. Epub ahead of print 2025 Jan 30.	No drug
1494	Saperia S, Plahouras J, Best M, Kidd S, Zakzanis K, Foussias G. The cognitive model of negative symptoms: a systematic review and meta-analysis of the dysfunctional belief systems associated with negative symptoms in schizophrenia spectrum disorders. Psychol Med. 2025 Feb 5;55:e11. doi: 10.1017/S0033291724003325.	Review
1495	Corsi-Zuelli F, Donohoe G, Griffiths SL, Del-Ben CM, Watson AJ, Burke T, Lalousis PA, McKernan D, Morris D, Kelly J, McDonald C, Patlola SR, Pariante C, Barnes NM, Khandaker GM, Suckling J, Deakin B, Upthegrove R, Dauvermann MR. Depressive and negative symptoms in the early and established stages of schizophrenia: Integrating structural brain alterations, cognitive performance, and plasma interleukin 6 levels. Biol Psychiatry Glob Open Sci. 2024;5(2):100429. doi: 10.1016/j.bpsgos.2024.100429.	No drug
1496	Marder SR, Meibach RC. Risperidone in the treatment of schizophrenia. Am J Psychiatry. 1994;151(6):825-35. doi: 10.1176/ajp.151.6.825.	**Included**
1497	Danion JM, Rein W, Fleurot O. Improvement of schizophrenic patients with primary negative symptoms treated with amisulpride. Amisulpride Study Group. Am J Psychiatry. 1999;156(4):610-6. doi: 10.1176/ajp.156.4.610.	**Included**
1498	Lang X, Zang X, Yu F, Xiu M. Effects of low-dose combined olanzapine and sertraline on negative and depressive symptoms in treatment-resistant outpatients with acute exacerbated schizophrenia. Front Pharmacol. 2023;14:1166507. doi: 10.3389/fphar.2023.1166507.	Open

Included	96	
Excluded	1402	
No drug		918
Review		185
Opinion		58
Open-label		54
Case report/series		49
Animal		43
Unfocused		35
Non-marketed drug		12
Overlapping samples		10
Post hoc		5
Protocol		4
No placebo comparator		3
Lumping (diagnoses/drugs)		3
Post-mortem		3
Inadequate design		2
Unrelated		2
In vitro		0
Retracted/Removed		3
Duplicates		13

Supplementary Table S2. Drugs used in eligible studies of drug treatment of the negative symptoms of schizophrenia.

Antidepressant compounds

Selective Serotonin Reuptake Inhibitors (SSRIs):

Fluvoxamine

Paroxetine

Escitalopram

Citalopram

Serotonin-Noradrenaline Reuptake Inhibitors (SNRIs)

Duloxetine

Noradrenaline Reuptake Inhibitors (NaRIs)

Reboxetine

Mixed Noradrenaline-Dopamine Reuptake Inhibitors (Na-DaRIs)

Bupropion

Noradrenaline-Serotonin Specific Antagonists (NaSSA)

Mirtazapine: Presynaptic α_2 adrenoceptor/5-HT_{2A} serotonin receptor antagonist

Tetracyclics

Maprotiline: Mainly noradrenaline transporter inhibitor

Serotonin receptor antagonists and reuptake inhibitors (SARIs)

Trazodone (Combined SERT/5-HT_{2A}/α_1 receptor inhibitor/partial 5-HT_{1A} agonist)

Drugs interfering with serotonergic function

Tropisetron: 5-HT_3 serotonin receptor antagonist and α7-nicotinic receptor partial agonist, weak 5-HT_4 serotonin receptor antagonist, an indole derivative used as an antiemetic

Granisetron: 5-HT_3 serotonin receptor antagonist, an indazole derivative used as an antiemetic, with minimal cholinergic effects and no activity on other than 5-HT_3 serotonin receptors

Pimavanserin: 5-HT_{2A} serotonin receptor inverse agonist and 5-HT_{2C} serotonin receptor antagonist with no known dopaminergic effects, bland σ_1 site antagonist, primarily used to treat Parkinson's disease-associated psychosis

Ritanserin: 5-$HT_{2A/2B/2C}$ serotonin receptor antagonist and Diacylglycerol kinase α (DGKA) inhibitor

Glutamatergic modulators active at the NMDA receptors

Glycine: NMDA glutamatergic receptor co-agonist

DCS (D-cycloserine): Partial NMDA glutamatergic receptor agonist

Memantine: Non-competitive NMDA receptor antagonist

Cholinergic compounds

Galantamine: Acetylcholinesterase inhibitor, used in the dementias

Citicoline (INN), cytidine diphosphate-choline (CDP-choline) or cytidine 5'-diphosphocholine: Phosphatidylcholine precursor, makes part of biological membranes and may have neuroprotective effects (stimulates the sulfoadenosin-methionine shuttle and glutathione synthesis and preserves other phospholipids, like phosphatidylethanolamine, phosphatidylcholine, sphingomyelin, and cardiolipin by decreasing phospholipase A2 action). The brain uses it to synthesise acetylcholine. Used to recover from stroke and enhance cognition

MAO-B Inhibitors

Selegiline (L-Deprenyl): MAO-B inhibitor, used in Parkinson's disease, since it increases synaptic dopamine levels

Rasagiline: MAO-B inhibitor, used in Parkinson's disease, since it increases synaptic dopamine levels; differs from selegiline in that it has no amphetamine-like metabolites

Central Nervous System stimulants

Modafinil: Mild inhibitor of the dopamine transporter (DAT), used in excessive daytime sleepiness secondary to night shifts, obstructive sleep apnoea and narcolepsy; psychoactive and euphorigenic like its R-enantiomer armodafinil. It induces CYP3A4 and CYP1A2, while inhibiting CYP2C19

Armodafinil: Modafinil's R-enantiomer; like the racemic compound, it increases dopamine in synapses; used for the same purposes as modafinil and its precursor, adrafinil; similarly to racemic modafinil, it moderately induces CYP3A4 and moderately inhibits CYP2C19, but differently from modafinil, it does not induce CYP1A2

Peptide mixtures and peptide analogues

Oxytocin: Nonapeptide (Cys–Tyr–Ile–Gln–Asn–Cys–Pro–Leu–Gly–NH_2) neurohormone and neuromodulator. Increases empathy, attachment and affiliation. Binds to OT1 and OT2 receptors. Modulates monoaminergic activities, namely dopaminergic activity in the n. accumbens. Used as a spray or intravenously in autism spectrum disorder

1-Deamino 8-D-arginine vasopressin (DDAVP, Desmopressin): Arginine-vasopressin analogue, binds to V_2 arginin-vasopressin receptors and is used to control water-electrolyte balance

Cerebrolysin (FPF-1070): A mixture of enzymatically-treated peptides derived from pig brain containing several growth factors (Brain-Derived Neurotrophic Factor [BDNF], Glial cell line-Derived Neurotrophic Factor [GDNF], Nerve Growth Factor [NGF], and Ciliary NeuroTrophic Factor [CNTF]). Used without sound evidence for vascular dementia and Alzheimer's disease, against negative evidence for stroke, and also for a variety of neurodegenerative diseases

Steroids and steroid receptor-active drugs

Pregnenolone (pregn-5-en-3β-ol-20-one): Neurosteroid, $GABA_A$ receptor-negative allosteric modulator, NMDA glutamate receptor positive allosteric modulator, CB_1 cannabinoid negative allosteric modulator, binds brain microtubule-associated protein 2 (MAP2) and affects neurite growth

Raloxifene: Selective oestrogen receptor modulator (SERM); inhibits oestrogen receptors. Benzothiophene derivative. Used in osteoporosis of post-menopausal women

- **Sarsasapogenin ((25S)-5β-Spirostan-3β-ol)**: Plant steroid, a glycoside that can be converted to pregnane-3,20-diol, a progesterone analogue, and testosterone. Isolated from *Sarsapailla* root, extracted mainly from the rhizome of *Anemarrhena asphodeloides*. Used in type II diabetes and Alzheimer's disease (it slows the loss of muscarinic cholinergic receptors)

Anti-inflammatory/Antioxidant and multipurpose drugs

- **Minocycline**: Tetracycline antibiotic; indirectly inhibits inducible nitric oxide synthase (NO-s); possesses neuroprotective and antinflammatory properties (halts degenerative disease progression). Used in treatment-resistant depression (not different from placebo) and negative symptoms of schizophrenia (consistent effect in meta-analyses as an add-on)
- ***N*-Acetylcysteine (NAC)**: Antioxidant, prodrug to L-cysteine and precursor to glutathione, eventual glutamatergic modulators (NMDA, mGluR2/3 and at higher doses mGluR5)
- **Resveratrol (3,5,4′-trihydroxy-trans-stilbene)**: A stilbenoid natural plant product with purported antioxidant properties used in various conditions (diabetes, dementia, ageing, cancer, dysmetabolism, cardiovascular disorders, neuroinflammation, endothelial disorders) with weak or no evidence; it acts on several targets like NAD(P)H dehydrogenase quinone 2 (NQO2), AKT1, glutathione-S transferase phosphate 1 (GSTP1), oestrogen receptor beta (ERβ), carbonyl reductase-1 (CBR1), and integrin αVβ, of which the role in resveratrol's purported clinical actions is unclear
- **Palmitoylethanolamide (PEA)**: Antinfiammatory and analgesic endogenous fatty acid amide and lipid modulator, proposed to act on the peroxisome proliferator-activated receptor alpha (PPAR-α) and on cannabinoid-like G-coupled receptors GPR55 and GPR119, behaving like anandamide, but lacking affinity for cannabinoid CB1 and CB2 receptors
- **Pioglitazone**: Thiazolidinedione, stimulates peroxisome proliferator-activated receptor gamma (PPAR-$\gamma_{1/2}$) (reduces insulin resistance) and, to a lesser extent, PPAR-α (reduces free fatty acids), used as an antidiabetic for limited times (<1 year, due to increased risk for bladder cancer)
- **Simvastatin**: Statin, selective and competitive inhibitor HMG-CoA reductase (3-hydroxy-3-methylglutaryl coenzyme A reductase), which converts HMG-CoA in mevalonate, a rate limiting step in the biosynthesis of cholesterol. Used in hypercholesterolaemias, obesity, liver and gastrointestinal disorders, but also in insomnia and depression as an adjuvant
- **L-Carnosine (β-Alanylhistidine)**: Dipeptide, the product of condensation of beta-alanine and histidine; antioxidant, reactive oxygen species (ROS) and α-β unsaturated aldehyde scavenger; it reduces the rate of formation of advanced glycation end-products. Used to counter ageing
- **Nanocurcumin**: Nanoparticle-encapsulated curcumin, which is a diketone tautomer, in enolic form in organic solvents and keto form in water. Pigment from turmeric rhizome, polyphenolic, diarylheptanoid, mixture of resin and turmeric oil, used as a food supplement; purported antimicrobial activity for nanoparticles. Curcumin was at the centre of fraudulent studies on its anticancer properties. Antioxidant and "neuroprotective" properties in the rat
- **Black Myrobalan (Choti Harad, *Terminalia chebula* Retz.)**: Medicinal plant of the *Combretaceae* family, rich in antioxidants, with antioxidant, antiacetylcholinesterase, and anti-inflammatory effects; it protects from free radicals. Traditionally used in Ayurvedic medicine for a variety of conditions
- **Berberine**: Quaternary ammonium salt from the protoberberine group of benzylisoquinoline alkaloids; natural secondary metabolite of some plants, including species of *Berberis*. It increases the removal from plasma of low-density lipoprotein C (LDL-C) by enhancing LDLR mRNA stability and increasing hepatic LDLR density. It is antioxidant through direct scavenging and upregulation of the P13K/AKT/Bcl-2 and Nrf2/HO-1-pathway. Used without evidence to regulate weight, blood lipids and blood sugar
- **Saffron**: Spice derived from the flower of *Crocus sativus*, containing safranal, 4-ketoisophorone, dihydrooxophorone, nonvolatile phytochemicals (the carotenoids zeaxanthin, lycopene, various α- and β-carotenes), crocetin and its glycoside crocein, the glycoside picrocrocin, and α-crocin. It has anti-inflammatory and antioxidant actions. In particular, it inhibits the activity and transfer to the nuclear factor kappa-light-chain-enhancer of activated B cells (NF-κB) nucleus and inhibits TNF-α transcription

Channel blockers

- **Verapamil**: Phenylalkylamine calcium channel blocker, mainly used for cardiovascular problems (hypertension, angina pectoris, supraventricular tachycardia), cluster headache, and migraine prevention
- **Carbamazepine**: Voltage-gated sodium channel inhibitor (in its inactive conformation); may also act on calcium channels and enhance serotonin release (or block its reuptake). Used as a mood stabiliser and antiepileptic agent
- **Riluzole**: Benzothiazole, tetrodotoxin-sensitive sodium channel blocker with controversial glutamate modulating acitivities (NMDA and kainate binding, allosteric $GABA_A$ potentiator; used in amyotrophic lateral sclerosis with doubtful efficacy

Phosphodiesterase inhibitors

- **Sildenafil**: cGMP-specific phosphodiesterase type 5 (PDE_5) inhibitor, used for erectile dysfunction and pulmonary hypertension with poor evidence (unclear effects on alpha and beta-adrenoceptors)
- **Cilostazol**: Selective inhibitor of phosphodiesterase type 3 (PDE_3) with consequent increase in intracellular cAMP levels, in turn increasing the active form of protein kinase A (PKA), which then inhibits platelet aggregation and prevents myosin light-chain kinase activation, thus relaxing smooth muscles and inducing vasodilation. Used to treat intermittent claudication in peripheral vascular disease

Opioid receptor ligands

- **Naltrexone (*N*-cyclopropylmethylnoroxymorphone)**: An oxymorphone derivative; binds the opioid receptor (OR), strongly at the ORμ, less at the delta ORδ and much less at the ORκ. It is inverse agonist or plain antagonist of ORμ and weak antagonist or bland partial agonist of ORδ and ORκ. Used to treat substance use disorders and compulsions and the adverse events of interferone therapy

Xanthine derivatives

- **Pentoxifylline (3,7-Dimethyl-1-(5-oxohexyl)purine-2,6-dione)**: Purinergic receptor inhibitor (inhibits adenosine A2 receptors); non-selective phosphodiesterase inhibitor (but mostly PDE3 and PDE4), increases intracellular cyclic AMP, activates Protein Kinase A, inhibits the synthesis of TNF and leukotriene, reduces inflammation-innate immunity, increases red blood cell membrane deformability, allowing erythrocytes to cross narrow spaces, it reduces blood viscosity, platelet aggregation and blood clot formation. Used to treat intermittent claudication; possibly useful in cardiovascular and cerebrovascular disorders

Table S3. PRISMA 2020 Checklist.

Section and Topic	Item #	Checklist item	Location where item is reported
**TITLE**			1
Title	1	Identify the report as a systematic review.	1
**ABSTRACT**			2
Abstract	2	See the PRISMA 2020 for Abstracts checklist.	2
**INTRODUCTION**			2-4
Rationale	3	Describe the rationale for the review in the context of existing knowledge.	
Objectives	4	Provide an explicit statement of the objective(s) or question(s) the review addresses.	
**METHODS**			4-5
Eligibility criteria	5	Specify the inclusion and exclusion criteria for the review and how studies were grouped for the syntheses.	4
Information sources	6	Specify all databases, registers, websites, organisations, reference lists and other sources searched or consulted to identify studies. Specify the date when each source was last searched or consulted.	4
Search strategy	7	Present the full search strategies for all databases, registers and websites, including any filters and limits used.	4
Selection process	8	Specify the methods used to decide whether a study met the inclusion criteria of the review, including how many reviewers screened each record and each report retrieved, whether they worked independently, and if applicable, details of automation tools used in the process.	4
Data collection process	9	Specify the methods used to collect data from reports, including how many reviewers collected data from each report, whether they worked independently, any processes for obtaining or confirming data from study investigators, and if applicable, details of automation tools used in the process.	4
Data items	10a	List and define all outcomes for which data were sought. Specify whether all results that were compatible with each outcome domain in each study were sought (e.g. for all measures, time points, analyses), and if not, the methods used to decide which results to collect.	4
	10b	List and define all other variables for which data were sought (e.g. participant and intervention characteristics, funding sources). Describe any assumptions made about any missing or unclear information.	N/A
Study risk of bias assessment	11	Specify the methods used to assess risk of bias in the included studies, including details of the tool(s) used, how many reviewers assessed each study and whether they worked independently, and if applicable, details of automation tools used in the process.	4-5
Effect measures	12	Specify for each outcome the effect measure(s) (e.g. risk ratio, mean difference) used in the synthesis or presentation of results.	N/A
Synthesis methods	13a	Describe the processes used to decide which studies were eligible for each synthesis (e.g. tabulating the study intervention characteristics and comparing against the planned groups for each synthesis (item #5)).	N/A
	13b	Describe any methods required to prepare the data for presentation or synthesis, such as handling of missing summary statistics, or data conversions.	N/A
	13c	Describe any methods used to tabulate or visually display results of individual studies and syntheses.	4-5, Table 1
	13d	Describe any methods used to synthesize results and provide a rationale for the choice(s). If meta-analysis was performed, describe the model(s), method(s) to identify the presence and extent of statistical heterogeneity, and software package(s) used.	N/A
	13e	Describe any methods used to explore possible causes of heterogeneity among study results (e.g. subgroup analysis, meta-regression).	N/A
	13f	Describe any sensitivity analyses conducted to assess robustness of the synthesized results.	N/A
Reporting bias assessment	14	Describe any methods used to assess risk of bias due to missing results in a synthesis (arising from reporting biases).	4-5
Certainty assessment	15	Describe any methods used to assess certainty (or confidence) in the body of evidence for an outcome.	N/A
**RESULTS**			5-27
Study selection	16a	Describe the results of the search and selection process, from the number of records identified in the search to the number of studies included in the review, ideally using a flow diagram.	5-7

Section and Topic	Item #	Checklist item	Location where item is reported
	16b	Cite studies that might appear to meet the inclusion criteria, but which were excluded, and explain why they were excluded.	Suppl.
Study characteristics	17	Cite each included study and present its characteristics.	8-27
Risk of bias in studies	18	Present assessments of risk of bias for each included study.	8-27
Results of individual studies	19	For all outcomes, present, for each study: (a) summary statistics for each group (where appropriate) and (b) an effect estimate and its precision (e.g. confidence/credible interval), ideally using structured tables or plots.	N/A
Results of syntheses	20a	For each synthesis, briefly summarise the characteristics and risk of bias among contributing studies.	N/A
	20b	Present results of all statistical syntheses conducted. If meta-analysis was done, present for each the summary estimate and its precision (e.g. confidence/credible interval) and measures of statistical heterogeneity. If comparing groups, describe the direction of the effect.	N/A
	20c	Present results of all investigations of possible causes of heterogeneity among study results.	8-27
	20d	Present results of all sensitivity analyses conducted to assess the robustness of the synthesized results.	27
Reporting biases	21	Present assessments of risk of bias due to missing results (arising from reporting biases) for each synthesis assessed.	N/A
Certainty of evidence	22	Present assessments of certainty (or confidence) in the body of evidence for each outcome assessed.	N/A
**DISCUSSION**			28-35
Discussion	23a	Provide a general interpretation of the results in the context of other evidence.	28-29, 32-35
	23b	Discuss any limitations of the evidence included in the review.	34
	23c	Discuss any limitations of the review processes used.	34
	23d	Discuss implications of the results for practice, policy, and future research.	33-34
**OTHER INFORMATION**			34-35
Registration and protocol	24a	Provide registration information for the review, including register name and registration number, or state that the review was not registered.	4,5,34
	24b	Indicate where the review protocol can be accessed, or state that a protocol was not prepared.	N/A
	24c	Describe and explain any amendments to information provided at registration or in the protocol.	N/A
Support	25	Describe sources of financial or non-financial support for the review, and the role of the funders or sponsors in the review.	34
Competing interests	26	Declare any competing interests of review authors.	34-35
Availability of data, code and other materials	27	Report which of the following are publicly available and where they can be found: template data collection forms; data extracted from included studies; data used for all analyses; analytic code; any other materials used in the review.	N/A, Suppl.

From: Page MJ, McKenzie JE, Bossuyt PM, Boutron I, Hoffmann TC, Mulrow CD, et al. The PRISMA 2020 statement: an updated guideline for reporting systematic reviews. BMJ 2021;372:n71. doi: 10.1136/bmj.n71
